# Ti-catalyzed ring-opening oxidative amination of methylenecyclopropanes with diazenes†

**DOI:** 10.1039/d0sc01998d

**Published:** 2020-06-23

**Authors:** Evan P. Beaumier, Amy A. Ott, Xuelan Wen, Zachary W. Davis-Gilbert, T. Alexander Wheeler, Joseph J. Topczewski, Jason D. Goodpaster, Ian A. Tonks

**Affiliations:** Department of Chemistry, University of Minnesota – Twin Cities 207 Pleasant St SE Minneapolis MN 55455 USA jgoodpas@umn.edu itonks@umn.edu

## Abstract

The ring-opening oxidative amination of methylenecyclopropanes (MCPs) with diazenes catalyzed by py_3_TiCl_2_(NR) complexes is reported. This reaction selectively generates branched α-methylene imines as opposed to linear α,β-unsaturated imines, which are difficult to access *via* other methods. Products can be isolated as the imine or hydrolyzed to the corresponding ketone in good yields. Mechanistic investigation *via* density functional theory suggests that the regioselectivity of these products results from a Curtin–Hammett kinetic scenario, where reversible β-carbon elimination of a spirocyclic [2 + 2] azatitanacyclobutene intermediate is followed by selectivity-determining β-hydrogen elimination of the resulting metallacycle. Further functionalizations of these branched α-methylene imine products are explored, demonstrating their utility as building blocks.

## Abstract 2

The ring-opening oxidative amination of methylenecyclopropanes (MCPs) with diazenes catalyzed by py_3_TiCl_2_(NR) complexes is reported.

## Graphical abstract

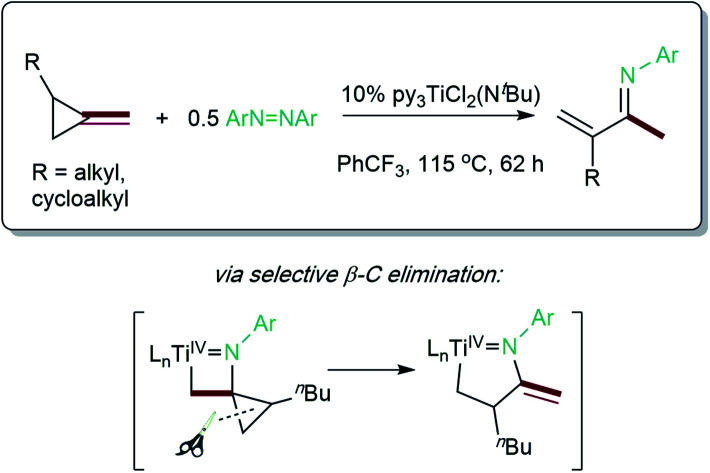

## Introduction

Metal-catalyzed oxidative amination reactions are increasingly important methods of forming new carbon–nitrogen bonds.^1^ There are now many examples of metal-catalyzed oxidative C–H amination and alkene aziridination reactions, most commonly with late transition metals such as Fe, Rh, and Ag. In contrast, there are few examples with early transition metals such as Ti. Typically, Ti-catalyzed C–N bond forming reactions are redox neutral hydrofunctionalization reactions;^2^ the thermodynamic stability of Ti^IV^ makes catalytic transformations involving redox at the metal center more challenging.

Recently, we found that it is possible to exploit the [2 + 2] cycloaddition reaction between Ti imidos (Ti≡NR) and alkynes to perform oxidative amination reactions.^3^ Further insertion of unsaturated substrates into the azatitanacyclobutene resulting from [2 + 2] cycloaddition gave expanded metallacycles that underwent reductive processes to liberate aminated products. The resulting transient Ti^II^ species can be reoxidized to Ti^IV^ with azobenzene. Using this approach, we have designed new routes to pyrroles, α,β-unsaturated imines, α-iminocyclopropanes, carbodiimides, and pyrazoles.

**Fig. 1:**
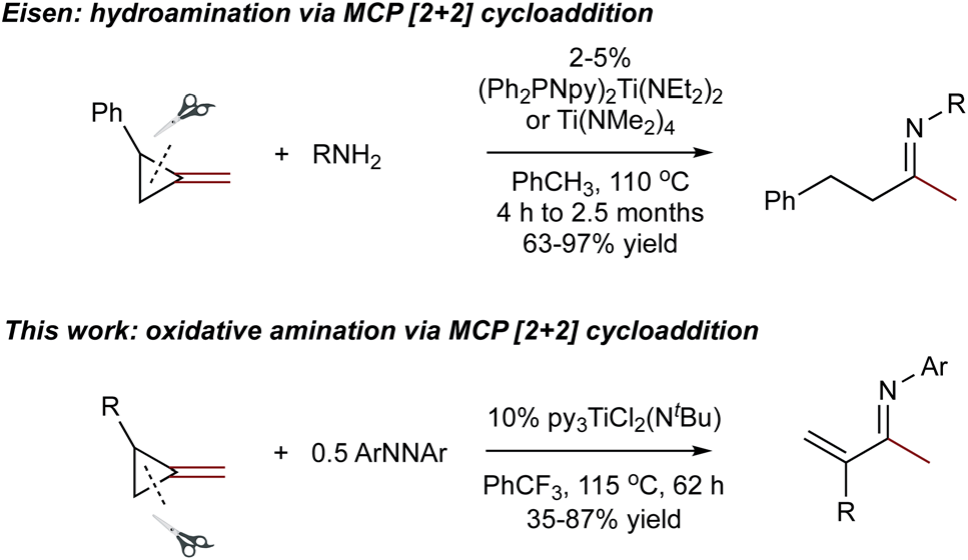
Top: Eisen's report of Ti-catalyzed ring-opening hydroamination of MCPs. Bottom: TI-catalyzed oxidative amination of MCPs (this work).

In light of these discoveries, we sought to expand on this oxidative amination beyond alkynes to other substrates capable of [2 + 2] cycloaddition with Ti imidos. In this regard, methylenecyclopropanes (MCPs) were targeted. Eisen demonstrated that Ti and Zr precatalysts can add primary amines across MCPs through a hydroamination mechanism that included a [2 + 2] cycloaddition with a group IV imido and subsequent β-carbon elimination of the corresponding spirocyclic azatitanacyclobutane, eventually providing linear or branched imines upon protonolysis (Fig. 1, top).^4^ Eisen suggested that the highly strained β-carbon in MCPs provides significant “sp-like” character which allows for accessible [2 + 2] cycloadditions with group IV imidos.^4*a*,*b*^ Furthermore, the β-carbon elimination in MCPs can occur due to the release of ring strain from the cyclopropyl moiety.^5^ Based on this strategy, we report that Ti imido catalysts are capable of the ring-opening oxidative amination of terminal MCPs to yield unusual α-methylene substituted imines, which are challenging to access *via* other methods (Fig. 1, bottom).^6^ A full computational analysis of the regioselectivity toward forming the α-methylene imine product is presented, as well several examples of further functionalization of these useful building blocks.

## Results and discussion

Reaction of 2.2 equiv. of 1-butyl-2-methylenecyclopropane (**1a**) with azobenzene and 5 mol% [py_2_TiCl_2_(NPh)]_2_ at 115 °C in PhCF_3_ for 62 hours cleanly provided the α-methylene imine product 3-methylene-*N*-phenylheptan-2-imine (**2a**) which was isolated in 68% yield on a >1 g scale (eqn (1)).1
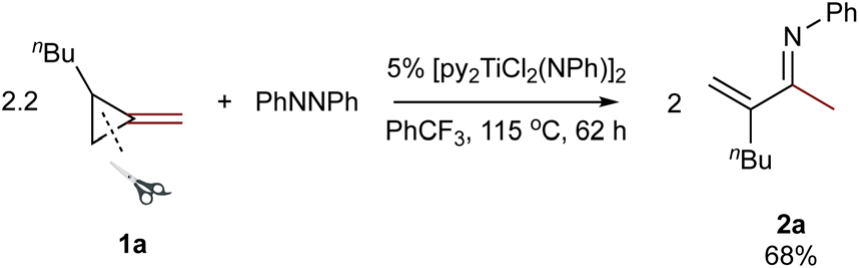


**Fig. 2:**
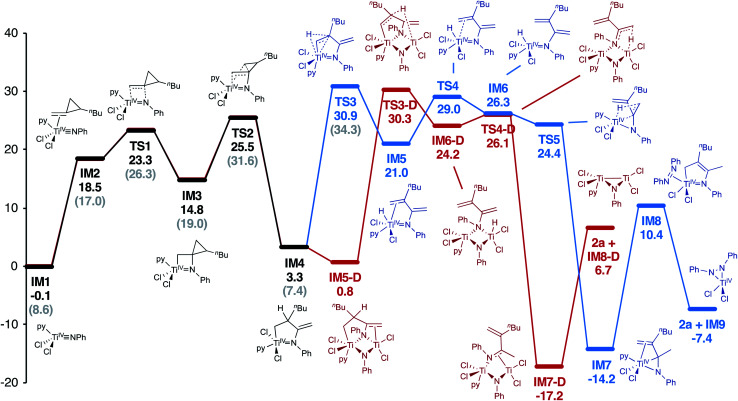
Computational analysis (M06/6-311G(d,p), SMD PhCF_3_, 388.15 K) of Ti-catalyzed oxidative amination of **1a**. Free energies reported in kcal mol^−1^. Both a monometallic pathway (blue) and bimetallic pathway (red) are energetically feasible. Similarly, catalyst species with a ligated pyridine (black) or without ligated pyridine (grey) also possible, although the ligated pyridine pathway is lower in energy.

**Fig. 3:**
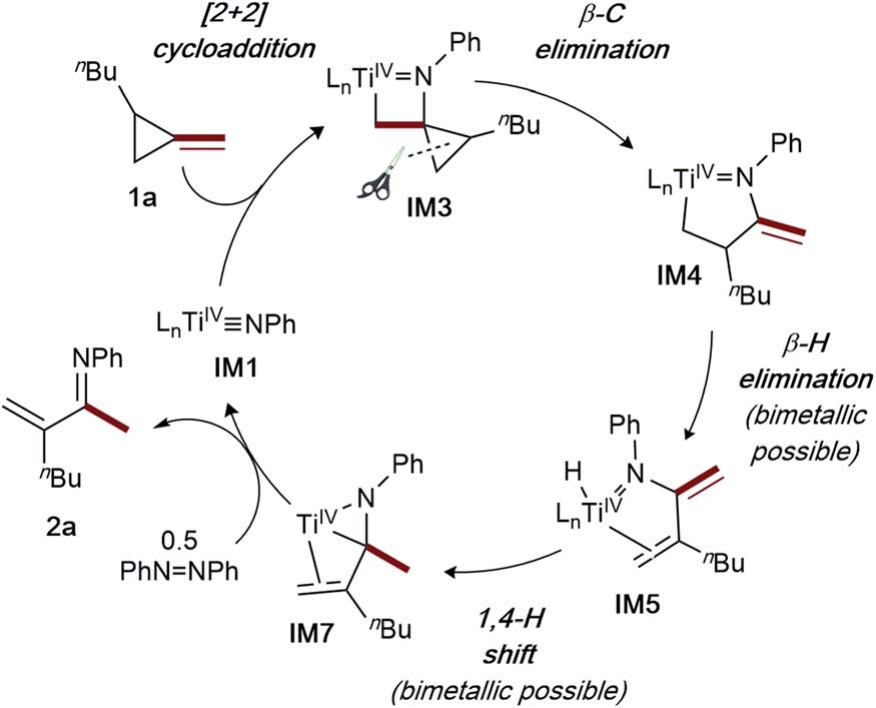
Simplified catalytic cycle for α-methylene imine formation *via* ring-opening oxidative amination of MCPs.

A full computational analysis of the catalytic reaction coordinate is presented in Fig. 2, as well as a simplified catalytic cycle in Fig. 3. As previously observed in [2 + 2 + 1] pyrrole synthesis,^3*d*^ dative ligand speciation can impact the energetics of the overall catalytic cycle, and both mono-pyridine (Fig. 2, black) and no-pyridine (Fig. 2, grey) bound manifolds are possible, although the mono-pyridine bound Ti manifold is generally lower in energy. First, Markovnikov-like^2*a*,4*a*,*b*^ [2 + 2] cycloaddition of a Ti imido (**IM1**) with **1a** yields the spirocyclic azatitanacyclobutane **IM3**. Next, selective β-C elimination of the unsubstituted β-C-C bond generates an azatitana-methylenecyclopentane (**IM4**). The origin of selectivity will be discussed later (*vide infra*).

From **IM4**, two feasible pathways diverge. First, a monometallic pathway in which **IM4** undergoes rate-determining (**TS3**, 30.9 kcal mol^−1^) β-H elimination to form a Ti hydride (**IM5**). Next, ligand rearrangement (**IM5** to **IM6**) and a 1,4-H migration^7*a*^ yields the α,β-unsaturated imine adduct **IM7**. An N–H reductive elimination pathway was also considered from **IM5**, but it is significantly higher in energy (Fig. S58†). Associative displacement^7*a*^ of the α,β-unsaturated imine by PhNNPh (**IM8**) results in charge transfer^3*g*^ from the κ^2^-α,β-unsaturated imine to azobenzene, liberating the product and a Ti^IV^ η^2^-hydrazide (**IM9**) which can undergo disproportionation to close the catalytic cycle.^3*d*^ Similar to a report on isocyanide oxidative amination,^3*g*^ PhNNPh coordination is required for product release to avoid high-energy free Ti^II^ species.^7^

Alternately, **IM4** can undergo roughly thermoneutral ligand exchange to form a bimetallic **IM5-D**, which similarly undergoes rate-determining (**TS3-D**, 30.3 kcal mol^−1^) β-H elimination to form **IM6-D**. Next, a 1,4-H migration generates **IM7-D**, which can undergo product release prior to PhNNPh reoxidation to close the catalytic cycle. Both pathways are very similar in overall energy; complex/intractable reaction kinetics are observed, indicating that both are likely contributing to the overall rate.

**Fig. 4:**
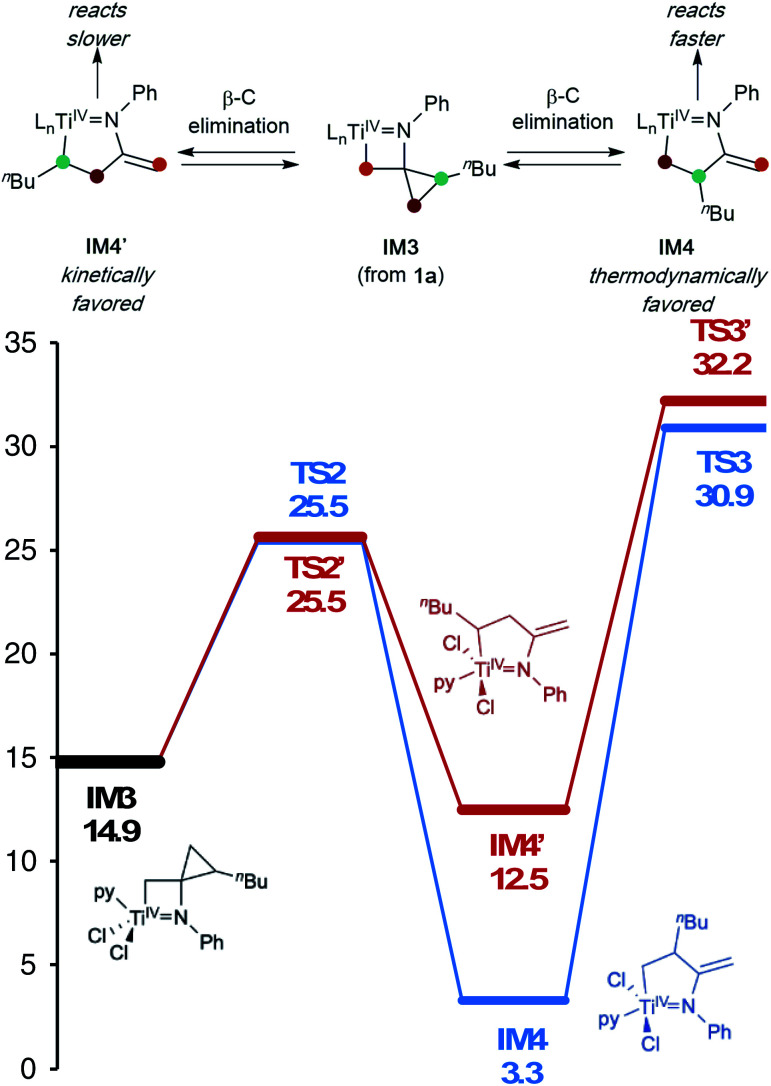
β-Carbon elimination from **1a** thermodynamically favors formation of **IM4** over **IM4′**, and kinetic formation of **IM4′** is reversible (M06/6-311G(d,p), SMD PhCF_3_, 388.15 K). L_n_ = pyCl_2_.

**Fig. 5:**
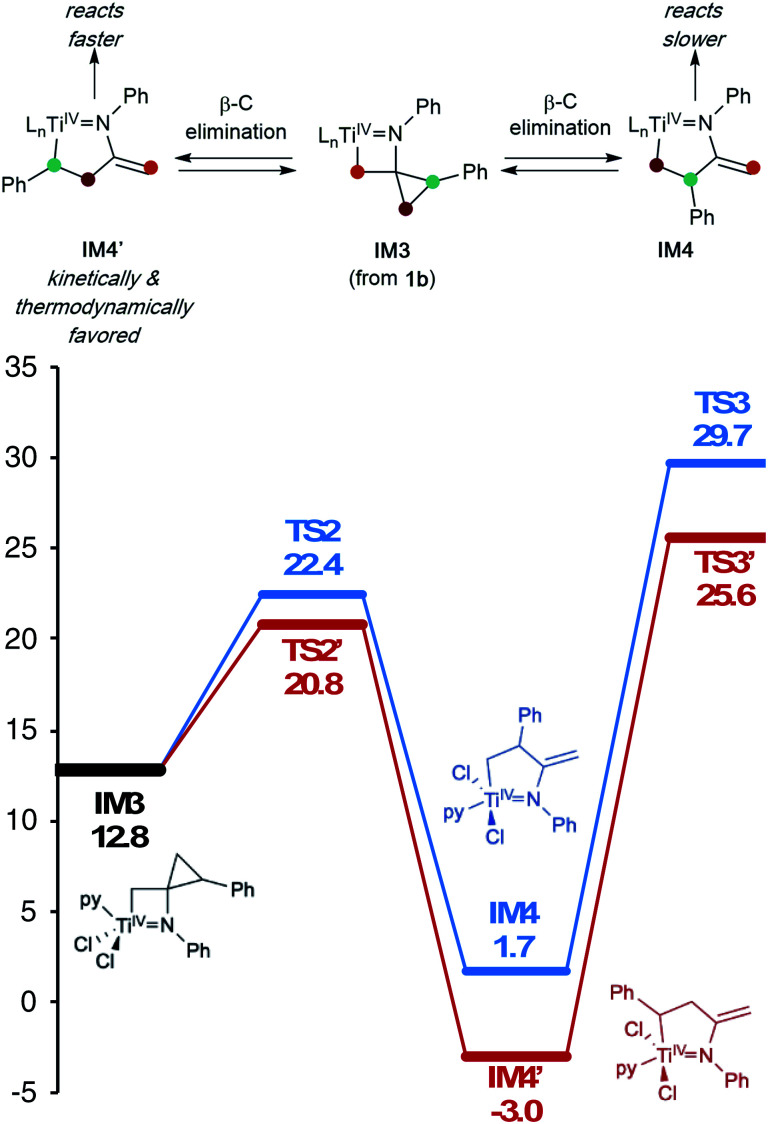
β-Carbon elimination from **1b** both kinetically and thermodynamically favors formation of **IM4′** over **IM4** (M06/6-311G(d,p), SMD PhCF_3_, 388.15 K). L_n_ = pyCl_2_.

In this oxidative amination of **1a**, β-C elimination occurs with opposite regioselectivity than in the previously-reported Ti-catalyzed hydroamination of 1-phenyl-2-methylenecyclopropane (**1b**) (Fig. 1, top). As a result, the regioselectivity of C–C ring-opening was investigated computationally (Fig. 4 and 5). For **1a**, ring-opening of the substituted C–C bond to form **IM4** or **IM4′** is identical (**TS2′** = **TS2** = 25.5 kcal mol^−1^), but ultimately, **IM4** (3.3 kcal mol^−1^) is significantly more stable than **IM4′** (12.5 kcal mol^−1^) (Fig. 4).^8^ Since the subsequent β-H elimination (**TS3**/**TS3′**) is rate-determining, the C–C ring opening *via* β-C elimination is proposed to be reversible, leading to Curtin–Hammett control of the regioselectivity favoring reaction through **TS3** instead of higher-energy **TS3′**. In contrast, for **1b** ring-opening of the substituted benzylic C–C bond to form **IM4′** is both kinetically and thermodynamically favored, and **TS3′** is lower in energy than **TS3**, leading to the opposite regioselectivity compared to **1a** (Fig. 5).^9^ Additional computations were performed considering initial pyridine dissociation before ring-opening (Fig. S59 and S60†) and lead to the same conclusion. Computational analysis of Eisen's hydroamination of **1b** with Ti(NMe_2_)_4_ leads to the same conclusion (Fig. S61†).

**Fig. 6:**
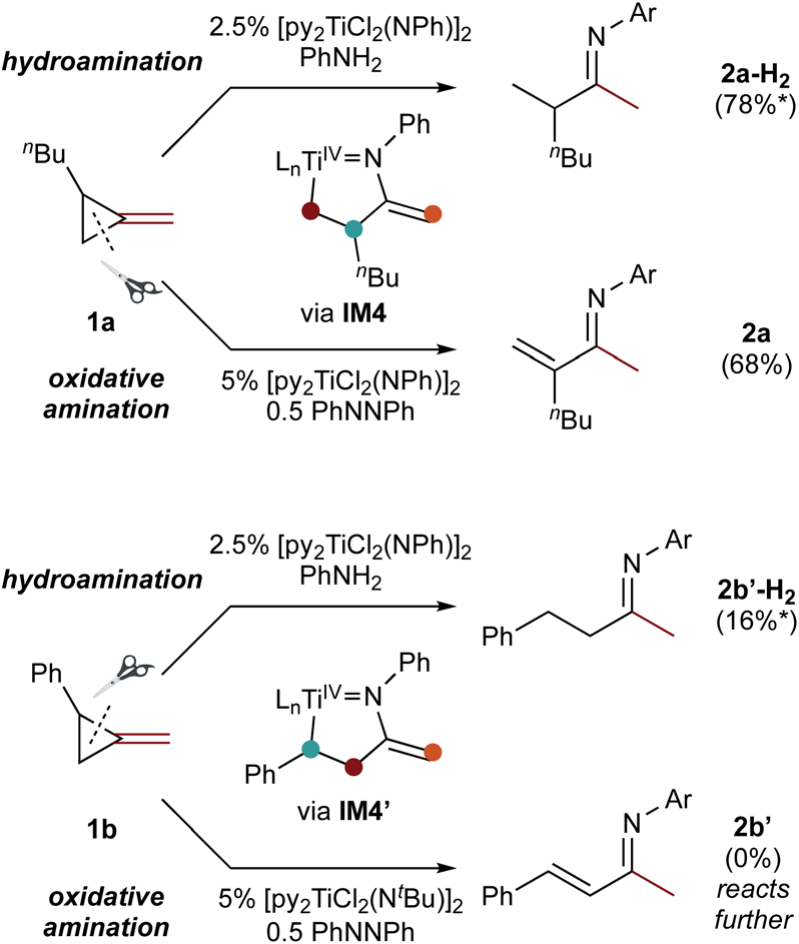
Regioselectivity of hydroamination and oxidative amination of **1a** and **1b** follow the same substrate control mechanisms. Conditions: 115 °C, 62 h (ox. amination) 20 h (hydroamination). *Yield determined *via* No-D ^1^H NMR spectroscopy.

Since protonolysis is often rate-determining in alkyne hydroamination,^2*a*^ we hypothesized that similar manifolds of regiocontrol would also impact MCP hydroamination, and that hydroamination of **1a** would lead to the formation of branched products, whereas **1b** would lead to linear products (Fig. 6). In fact, reaction of **1a** with PhNH_2_ catalyzed by [py_2_TiCl_2_(NPh)]_2_ under standard conditions leads to the formation of the branched product **2a-H2**, while reaction of **1b** leads to the linear product **2b′-H2**. Attempts at oxidative amination of **1b** led to an intractable mixture, although GC-MS analysis indicated the possible formation of a quinoline. Quinoline formation indicates that **2b′** had formed initially, as Mindiola had previously shown that α,β-unsaturated β-aryl imines underwent autoxidation to quinolines under similar reaction conditions (see ESI† for details).^10^ Nonetheless, these studies indicate that across multiple systems the regioselectivity of β-C elimination is under Curtin–Hammett control for alkyl and aryl-substituted cyclopropanes.

Table 1 depicts the initial scope of the MCP oxidative amination reaction. Monosubstituted aliphatic (**1a**, **1c**), benzylic (**1d**), and bicyclic aliphatic (**1e**, **1f**) MCPs all give good yields of the branched (or cyclic) α,β-unsaturated imines. Azobenzenes with electron donating and withdrawing groups also give good yields of **2a-tol** and **2a-OCF3**, respectively. Internally-substituted cyclopropylidene **1g** and methylenecyclobutane **1h** both did not react, presumably due to sterics (**1g**) or lack of sufficient ring strain (**1h**).^11^ Currently, the reaction scope is limited to monosubstituted or bicyclic aliphatic or benzylic MCPs. The substrate scope and practicality of this process are limited in large part by the difficulty in synthesizing MCPs *via* traditional routes.^12^ However, recent work from Uyeda on alkylidenecyclopropane synthesis indicates that more efficient access of these substrates may be possible in the future.^13^ Overall, this method represents an efficient route to generate α-methylene ketimines, which are often difficult to access *via* condensation of α,β-unsaturated ketones with primary amines because of competitive Michael addition.^14^

**Table 1:** Scope of Ti-catalyzed oxidative amination of MCPs^a^

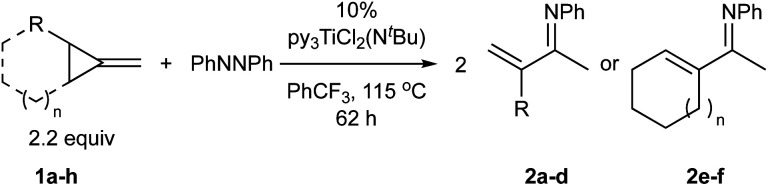			
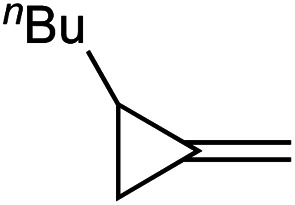	**1a** e	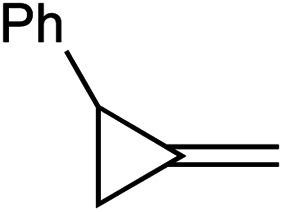	**1b**
	68%		0% (98%)^b^
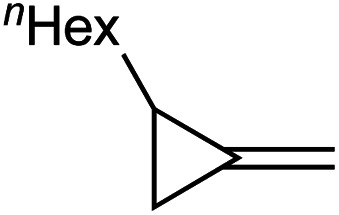	**1c** c	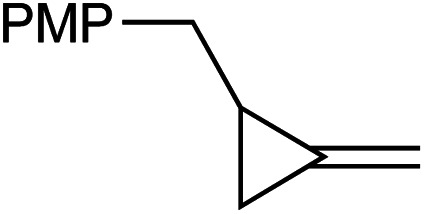	**1d** c ^,^ d
	77%		59%
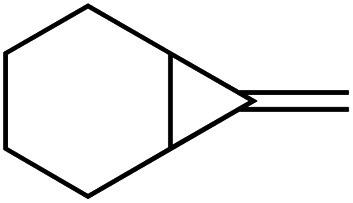	**1e** e	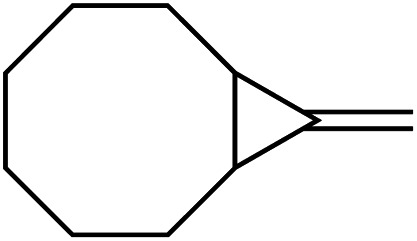	**1f** c ^,^ f
	67%		35%
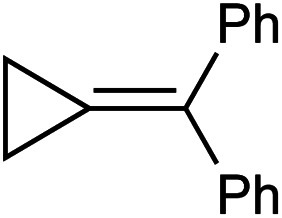	**1g**	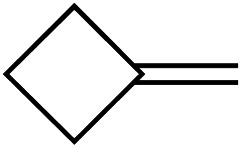	**1h**
	N.R.		N.R.
	—		—
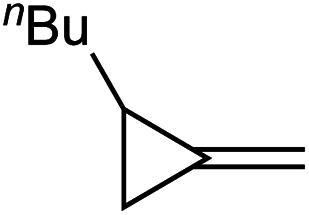	**1a** g	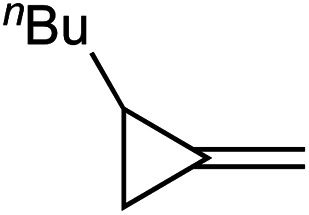	**1a** g
	Ph = *p*-tol (**2a-tol**)		Ph = *p*-CF_3_OPh (**2a-OCF3**)
	60%		59%

a Isolated yields.

b PhNNPh conversion.

c Isolated as ketone after hydrolysis (2 M HCl, THF, 25 °C, 16 h).

d 5% [py_2_TiCl_2_(N^*t*^Bu)]_2_ as catalyst.

e 5% [py_2_TiCl_2_(NPh)]_2_ as catalyst.

f 145 °C, 110 h, 4 equiv. **1f**.

g 4 equiv. **1a**.

**Fig. 7:**
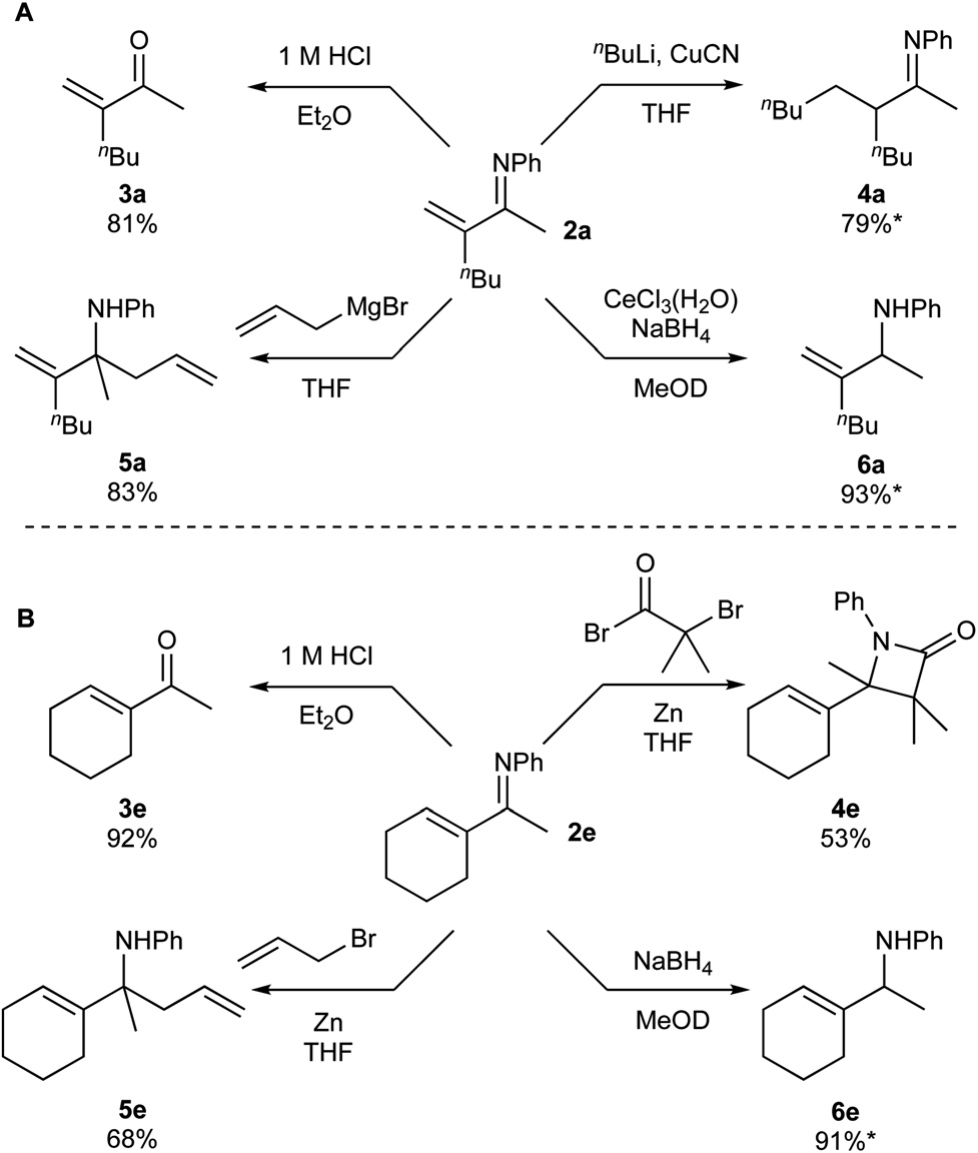
Further functionalizations of products **2a** (**A**) and **2e** (**B**). *Denotes yields that were determined *via*^1^H NMR spectroscopy.

Given that there are few reports on the synthesis of α-methylene ketimines,^6^ we sought to explore the reactivity of products **2a** and **2e** (Fig. 7). Acidic hydrolysis of **2a** and **2e** generated ketones **3a** and **3e**, respectively. Intriguingly, the α-methylene moiety does not undergo acid-catalyzed isomerization to the internal alkene during the formation of **3a**. Conjugate addition product **4a** can be synthesized *via* reaction of CuCN and ^*n*^BuLi with **2a**, however isolation was complicated by enamine–imine tautomerization (see ESI† for details). β-Lactam **4e** can be generated in modest yield from **2d** using an *in situ* ketene formation, based on a procedure adapted from the flow system reported by Haftner and Ley.^15^ β-lactams are a key pharmacophore in antibiotics.^16^

1,2-Allylations can also be accomplished *via* reaction of **2a** with allyl magnesium bromide to form homoallylic amine **5a**, or reaction of **2e** with zinc and allyl bromide to form homoallylic amine **5e**.^17^ Finally, both substrates can be reduced to allyl amines selectively, either *via* Luche reduction of **2a** to form **6a** or NaBH_4_ reduction of **2e** to form **6e**. Allyl amines are common precursors for nitrogen containing pharmaceuticals and natural products,^18^ and are functional groups present in some bioactive compounds, including naftifine and flunarizine.^19^ Thus, the reactivity of these unsaturated imines can be controlled in terms of selective 1,2 or 1,4-additions, depending on the reaction conditions. Additionally, cyclization reactions of these products present the potential for entry into heterocyclic moieties. With this reactivity in hand, we hope that these compounds can be incorporated toward the synthesis of useful nitrogen-containing compounds, including pharmaceuticals and natural products.

## Conclusions

In conclusion, simple Ti imido halide complexes catalyze the ring-opening oxidative amination reaction of MCPs and diazenes to generate α-methylene imines in good yields. Importantly, this reaction further expands on the library of unsaturated substrates capable of undergoing Ti-catalyzed oxidative amination, indicating that aminations of more challenging substrates may be possible. The regioselectivity of ring-opening is dictated by the substituents on the cyclopropane ring, as determined by DFT calculations. For simple alkyl-substituted methylenecyclopropanes, branched products are obtained because rate-determining β-H elimination allows for equilibration to the more stable titanacycle that will ultimately yield the branched product. Reactivity of these α-methylene imine products has shown that diverse post-functionalizations can be achieved and hopefully may allow for access to important nitrogen-containing compounds.

## Conflicts of interest

There are no conflicts to declare.

## Supplementary Material

SC-011-D0SC01998D-s001

## References

[cit1] Park Y., Kim Y., Chang S. (2017). Chem. Rev..

[cit2] Müller T. E., Hultzsch K. C., Yus M., Foubelo F., Tada M. (2008). Chem. Rev..

[cit3] Gilbert Z. W., Hue R. J., Tonks I. A. (2016). Nat. Chem..

[cit4] Smolensky E., Kapon M., Eisen M. S. (2005). Organometallics.

[cit5] O'Reilly M. E., Dutta S., Veige A. S. (2016). Chem. Rev..

[cit6] De Kimpe N., Tehrani K. A., Fonck G. (1996). J. Org. Chem..

[cit7] Guo J., Lu Y., Zhao R., Liu Z., Menberu W., Wang Z.-X. (2018). Chem. –Eur. J..

[cit8] Hartwig J. F., in Organotransition Metal Chemistry: From Bonding to Catalysis, University Science Books, Sausalito, 2010, ch. 3, p. 90

[cit9] Jiao Y., Evans M. E., Morris J., Brennessel W. W., Jones W. D. (2013). J. Am. Chem. Soc..

[cit10] Basuli F., Aneetha H., Huffman J. C., Mindiola D. J. (2005). J. Am. Chem. Soc..

[cit11] Wiberg K. B. (1986). Angew. Chem., Int. Ed. Engl..

[cit12] Kitatani K., Hiyama T., Nozaki H. (1977). Bull. Chem. Soc. Jpn..

[cit13] Pal S., Zhou Y.-Y., Uyeda C. (2017). J. Am. Chem. Soc..

[cit14] Odom A. L. (2006). Dalton Trans..

[cit15] Hafnter A., Ley S. V. (2015). Synlett.

[cit16] Kong K.-F., Schneper L., Mathee K. (2010). APMIS.

[cit17] Zhao L.-M., Zhang S.-Q., Jin H.-S., Wan L.-J., Dou F. (2012). Org. Lett..

[cit18] Johannsen M., Jørgensen K. A. (1998). Chem. Rev..

[cit19] Petranyi G., Ryder N. S., Stütz A. (1984). Science.

