# Supplementary material for: Ti-catalyzed ring-opening oxidative amination of methylenecyclopropanes with diazenes
Source: Chem Sci. 2020 Jun 23;11(27):7204–9. doi: 10.1039/d0sc01998d (PMC8159277; doi:10.1039/d0sc01998d)
Supplement: SC-011-D0SC01998D-s001 [file SC-011-D0SC01998D-s001.pdf]

Electronic Supplementary Material (ESI) for Chemical Science.
This journal is © The Royal Society of Chemistry 2020

Supporting Information:

Ring-opening oxidative amination of methylenecyclopropanes with diazenes via Ti^{IV}/Ti^{II} redox catalysis

Evan P. Beaumier, Amy A. Ott, Xuelan Wen, Zachary W. Davis-Gilbert, T. Alex Wheeler, Joseph J. Topczewski, Jason, D. Goodpaster* and Ian A. Tonks*

Contribution from the Department of Chemistry, University of Minnesota-Twin Cities, 207 Pleasant St. SE, Minneapolis MN 55455. E-mail: itonks@umn.edu

General Considerations **S3**

Synthesis of 1-methoxy-4-((2-methylenecyclopropyl)methyl)benzene (1c) S4

Synthesis of 1-butyl-2-methylenecyclopropane (1a) **S12**

Synthesis of 9-methylenebicyclo[6.1.0]nonane (1e) **S14**

Synthesis of 3-methylene-*N*-phenylheptan-2-imine (2a) **S16**

Synthesis of 3-methylene-*N*-(*p*-tolyl)heptan-2-imine (2a-tol) **S21**

Synthesis of 3-methylene-*N*-(4-(trifluoromethoxy)phenyl)heptan-2-imine (2a-OCF_3) **S26**

Synthesis of 3-methylenenonan-2-one (2c) **S31**

Synthesis of 3-(4-methoxybenzyl)but-3-en-2-one (2d) **S35**

Synthesis of 1-(cyclohex-1-en-1-yl)-*N*-phenylethan-1-imine (2e) **S37**

Synthesis of 1-(cyclooct-1-en-1-yl)ethan-1-one (2f) **S40**

General Procedure and NMR Reaction Data For Failed Substrates **S43**

NMR Reaction of 1b and Azobenzene **S45**

General Procedure For Hydroamination Reactions **S48**

NMR Reaction of 1a and Aniline Yielding 2a-H_2 .... **S49**

NMR Reaction of 1b and Aniline Yielding 2b'-H_2 .... **S52**

Synthesis of 3-methyleneheptan-2-one (3a) **S55**

Synthesis of 3-butyl-*N*-phenyloctan-2-imine (4a) **S57**

Synthesis of *N*-(4-methyl-5-methylenenon-1-en-4-yl)aniline (5a) **S60**

Synthesis of *N*-(3-methyleneheptan-2-yl)aniline (6a) **S63**

Synthesis of 1-(cyclohex-1-en-1-yl)ethan-1-one (3e) **S66**

Synthesis of 4-(cyclohex-1-en-1-yl)-3,3,4-trimethyl-1-phenylazetidin-2-one (4e) **S67**

Synthesis of *N*-(2-(cyclohex-1-en-1-yl)pent-4-en-2-yl)aniline (5e) S70
Synthesis of *N*-(1-(cyclohex-1-en-1-yl)ethyl)aniline (6e) S73
Computational Details... S74
References S103

General Considerations

All air- and moisture-sensitive compounds were manipulated in a glovebox under a nitrogen atmosphere. Solvents for air- and moisture-sensitive reactions were purified on a Pure Process Technologies Solvent Purification System (THF, hexanes, CH_2Cl_2, $PhCF_3$). Complexes $py_3TiCl_2(N^tBu)$ and $[py_2TiCl_2(N^tBu)]_2$ were prepared according to literature procedures.[1] Complex $[py_2TiCl_2(NPh)]_2$ was prepared according to literature procedure,[2] then purified by recrystallization from a CH_2Cl_2/pyridine mixture layered with hexanes at -35 °C then dried in vacuo at 110 °C overnight. Azobenzene was purchased from TCI America, purified via column chromatography (silica, hexanes), crushed in a mortar and pestle, and then dried in vacuo overnight. Substituted diazenes 1,2-di-*p*-tolyldiazene and 1,2-bis(4-(trifluoromethoxy)phenyl)diazene were prepared according to literature procedures,[3] crushed in a mortar and pestle, and then dried in vacuo overnight. Substrates **1b**,[4] **1c**,[4] **1e**,[4] **1g**,[5] and **1h**,[6] were prepared according to literature procedures. All liquid methylenecyclopropanes (MCPs) and **1h** were freeze-pump-thawed three times, brought into the glovebox and passed through activated basic alumina before being stored at -35 °C. C_6D_5Br was synthesized according to the literature procedure,[7] then vacuum distilled from CaH_2, freeze-pump-thawed three times, brought into the glove box, and passed through activated basic alumina before use. Reagents were purchased from Sigma Aldrich, TCI America, Oakwood Chemicals, Alfa Aesar, or Acros Organics and used without further purification. ^1H, ^{13}C, ^{15}N, $^1H^{15}N$-HMBC, $^1H^{13}C$-HSQC, and DEPT-135 NMR spectra were recorded on Bruker Avance 400 MHz, Bruker Avance III 500 MHz, or Bruker Avance III HD 500 MHz spectrometers. Chemical shifts are reported with respect to residual protio-solvent impurities for ^1H (s, 7.26 ppm for $CHCl_3$; s, 7.30 ppm for the most deshielded shift for $C_6D_4\mathit{H}Br$), solvent carbons for ^{13}C (t, 77.16 ppm for $CDCl_3$), and trifluoroacetic acid reference for ^{19}F (s, -76.6 ppm[2] for trifluoroacetic acid in $CDCl_3$). No-D reactions in $PhCF_3$ were referenced to 1,3,5-$(OMe)_3C_6H_3$ (s, 6.13 ppm for $C_6\mathit{H}_3$). All titanium-catalyzed reactions are highly air- and water-sensitive and all reagents and solvents should be thoroughly dried before use.

Synthesis of Substrates

Synthesis of 1-((2,2-dibromocyclopropyl)methyl)-4-methoxybenzene:

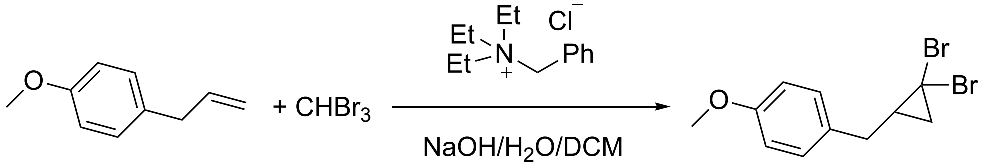

Benzyltriethylammonium chloride (801 mg., 3.53 mmol, 0.02 equiv), 4-allyl anisole (26.0 g, 0.175 mol, 1 equiv), and bromoform (63.9 g, 0.253 mol, 1.5 equiv) were added to a round-bottom flask containing CH_2Cl_2 (50 mL), and a NaOH solution (30 mL, 1:1 w/v) was added dropwise over 1 h by addition funnel. After the addition was complete, the reaction mixture was stirred for 3 days at room temperature. The reaction mixture was then poured into a separatory funnel containing hexanes (100 mL) and water (100 mL). The aqueous layer was extracted with hexanes (3 x 100 mL). The combined organic phases were washed with water (100 mL), dried over $MgSO_4$, filtered and concentrated in vacuo. The crude dark red liquid was then dried in vacuo on a Schlenk line at 80 °C to remove excess bromoform and 4-allyl anisole. The residue was filtered through a short plug of silica using pentane and was concentrated in vacuo to yield 1-((2,2-dibromocyclopropyl)methyl)-4-methoxybenzene (23.0 g, 42%) as a yellow oil.

^{1}H NMR (400 MHz, $CDCl_3$, 25 °C, δ, ppm): 7.23 (d, *J* = 8.5 Hz, 2H, Ar-C-*H*), 6.89 (d, *J* = 8.6 Hz, 2H, Ar-C-*H*), 3.81 (s, 3H, OCH_3), 2.97 (dd, *J* = 15.5, 7.0 Hz, 1H, Ar-CH-*H*), 2.72 (dd, *J* = 15.4, 6.2 Hz, 1H, Ar-CH-*H*), 2.19 – 1.69 (m, 2H), 1.50 – 1.11 (m, 1H).

^{13}C NMR (101 MHz, $CDCl_3$, 25 °C, δ, ppm): 158.4, 131.4, 129.4, 114.2, 55.4, 37.5, 32.3, 29.2, 28.6.

GC-HRMS: Calc. for $C_{11}H_{12}Br_2O$ [M^+] 319.9234; found 319.9201.

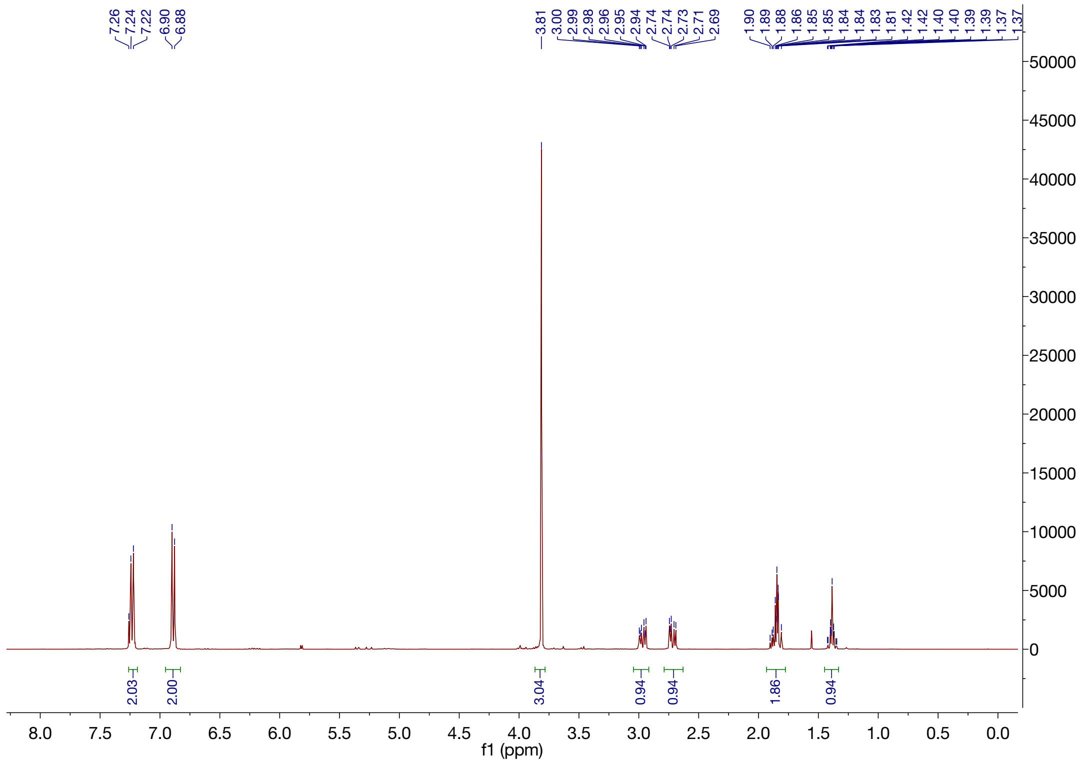

Figure S1. ¹H NMR of 1-((2,2-dibromocyclopropyl)methyl)-4-methoxybenzene in $CDCl_3$.

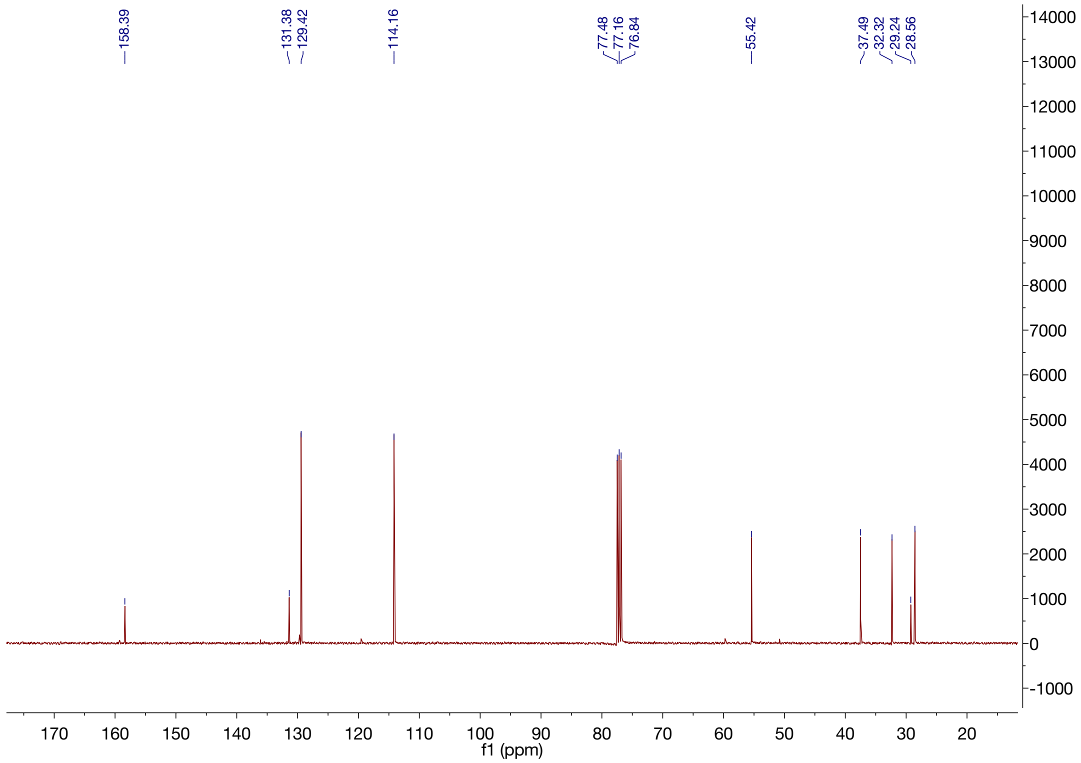

Figure S2. ^{13}C NMR of 1-((2,2-dibromocyclopropyl)methyl)-4-methoxybenzene in $CDCl_3$.

Synthesis of 1-((2-bromo-2-methylcyclopropyl)methyl)-4-methoxybenzene:

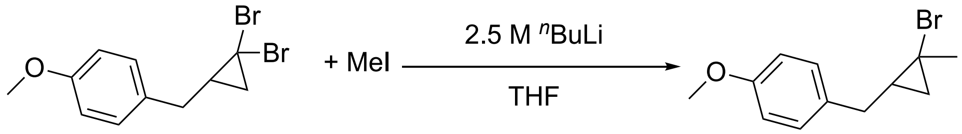

An oven-dried 250 mL 3-neck round bottom flask containing a stir bar was equipped with a nitrogen gas inlet, a thermometer, and a dry pressure equalizing addition funnel. Anhydrous THF (60 mL), MeI (22.8 g, 161 mmol, 2.6 equiv), and 1-((2,2-dibromocyclopropyl)methyl)-4-methoxybenzene (20.0 g, 62.4 mmol, 1 equiv) were then added to the flask. The reaction flask was then placed in a dewer and cooled in an EtOH/liquid nitrogen cold bath until the internal temperature was below -95 °C. Next, ^{n}BuLi (28.0 mL, 2.5 M in hexanes, 70.0 mmol, 1.1 equiv) was added slowly to the dried addition funnel via cannula transfer. Then, ^{n}BuLi was added slowly from the addition funnel to the reaction flask, while maintaining an internal temperature below -95 °C. After complete addition of the ^{n}BuLi, the reaction mixture was kept at -95 °C for an additional 2 h before slowly warming to room temperature. The reaction mixture was stirred overnight at room temperature, and then quenched with water (20 mL). The mixture was then poured into a separatory funnel containing hexanes (100 mL) and water (100 mL). The aqueous layer was then extracted with hexanes (3 x 100 mL). The combined organic phases were washed with water (100 mL), dried over $MgSO_4$, filtered and concentrated in vacuo. The crude oil was then passed through a short plug of silica using pentane. Concentration of the pentane fractions gave a yellow oil of crude 1-((2-bromo-2-methylcyclopropyl)methyl)-4-methoxybenzene as a mixture of diastereomers (12.3 g, 77%).

^{1}H NMR (400 MHz, $CDCl_3$, 25 °C, δ, ppm): 7.20 – 7.16 (m, 2H), 6.89 – 6.85 (m, 2H), 3.81 (s, 3H, OCH_3), 3.01 – 2.49 (m, 2H), 1.81 (s, 3H, -CH_3), 1.78 – 1.70 (m, 1H), 1.36 (dd, *J* = 10.0, 6.3 Hz, 1H, cyclopropyl C-*H*), 0.59 (t, *J* = 6.5 Hz, 1H, cyclopropyl C-*H*).

^{13}C NMR (101 MHz, $CDCl_3$, 25 °C, δ, ppm): 158.2, 158.1, 133.3, 132.7, 129.4, 129.1, 114.1, 114.0, 55.4, 38.0, 34.3, 33.6, 31.0, 28.7, 27.0, 24.9, 23.0.

GC-HRMS: Calc. for $C_{12}H_{15}BrO$ [M^+] 254.0306; found 254.0279.

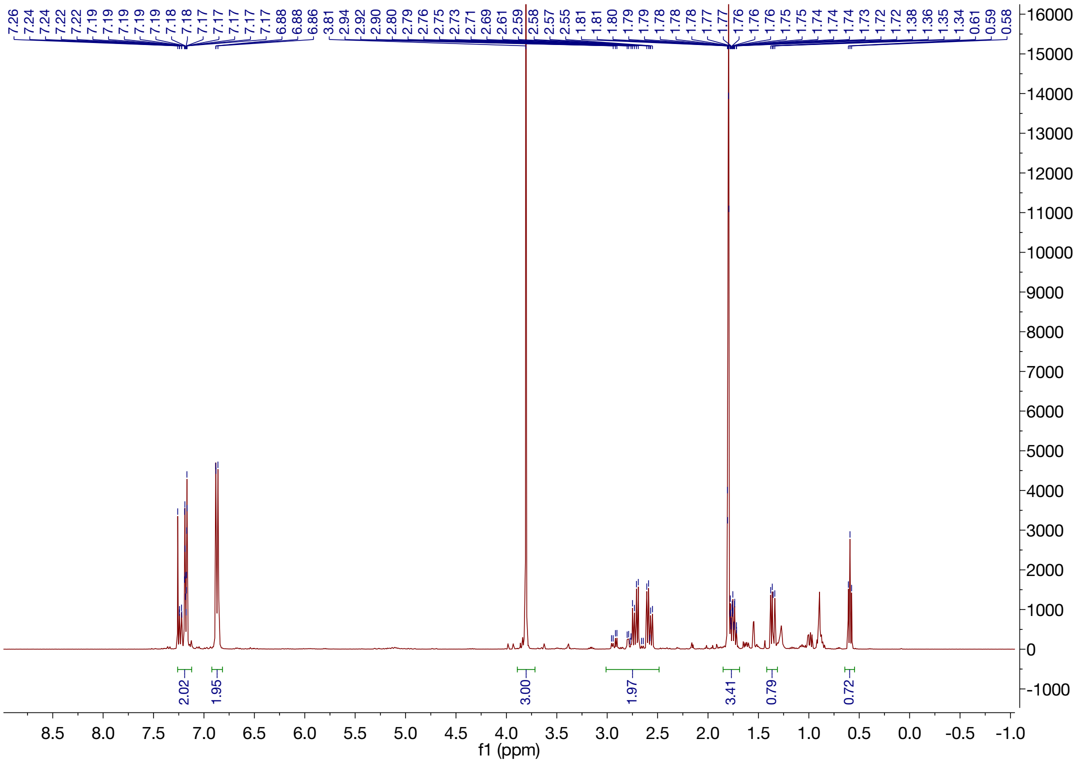

Figure S3. ^1H NMR of crude 1-((2-bromo-2-methylcyclopropyl)methyl)-4-methoxybenzene in $CDCl_3$.

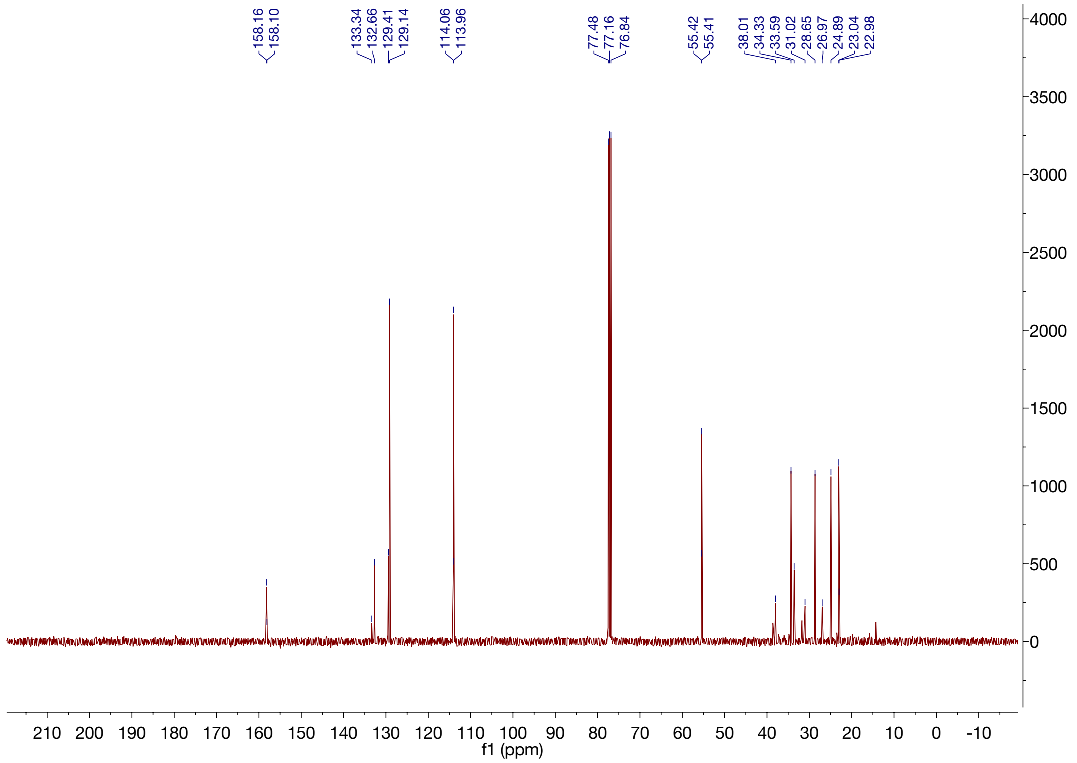

Figure S4. ^{13}C NMR of crude 1-((2-bromo-2-methylcyclopropyl)methyl)-4-methoxybenzene in $CDCl_3$.

Synthesis of 1-methoxy-4-((2-methylenecyclopropyl)methyl)benzene (1d):

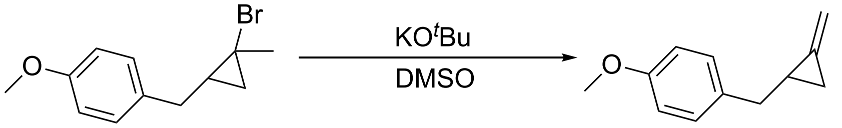

1d

DMSO (50 mL) was added to a 100 mL Schlenk flask containing a stir bar under a nitrogen atmosphere, and KO^{t}Bu (5.27 g, 47.0 mmol, 1.2 equiv) was added portion-wise. The reaction mixture was warmed to 60 °C to allow for full dissolution of the solid KO^{t}Bu. The reaction flask was then cooled to room temperature and 1-((2-bromo-2-methylcyclopropyl)methyl)-4-methoxybenzene (10.0 g, 39.2 mmol, 1 equiv) was added dropwise over 5 minutes. The reaction mixture was left to stir overnight. The reaction mixture was then poured into water (100 mL) and hexanes (100 mL). The aqueous layer was then extracted with hexanes (3 x 100 mL). The combined organic phases were washed with brine, dried over $MgSO_4$, filtered and concentrated in vacuo. The light yellow oil was then purified by column chromatography (2% EtOAc/hexanes) to yield **1d** (4.85 g, 71%) as a clear, colorless oil.

^{1}H NMR (400 MHz, $CDCl_3$, 25 °C, δ, ppm): 7.20 – 7.17 (m, 2H), 6.88 – 6.84 (m, 2H), 5.45 (m, 1H, =CH-*H*), 5.40 (m, 1H, =CH-*H*), 3.81 (s, 3H, OC*H*$_3$), 2.65 (dd, *J* = 8.0, 2.1 Hz, 1H, Ar-CH-*H*), 2.63 (dd, *J* = 8.0, 2.1 Hz, 1H, Ar-CH-*H*), 1.74 – 1.61 (m, 1H), 1.31 (tdd, *J* = 9.0, 2.5, 1.8 Hz, 1H, cyclopropyl C-*H*), 1.03 – 0.85 (m, 1H).

^{13}C NMR (101 MHz, $CDCl_3$, 25 °C, δ, ppm): 158.1, 136.4, 133.6, 129.4, 113.9, 103.4, 55.4, 38.2, 16.9, 9.7.

GC-HRMS: Calc. for $C_{12}H_{14}O$ [M^+] 174.1045; found 174.1066.

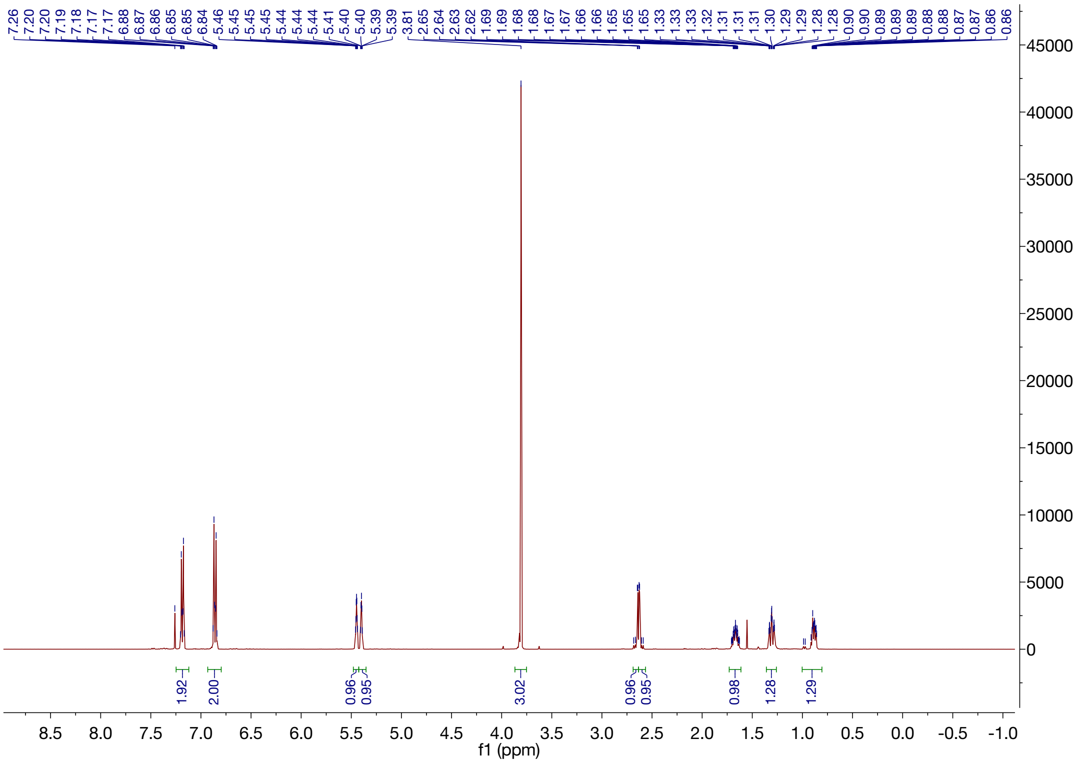

Figure S5. ^{1}H NMR of **1d** in $CDCl_3$.

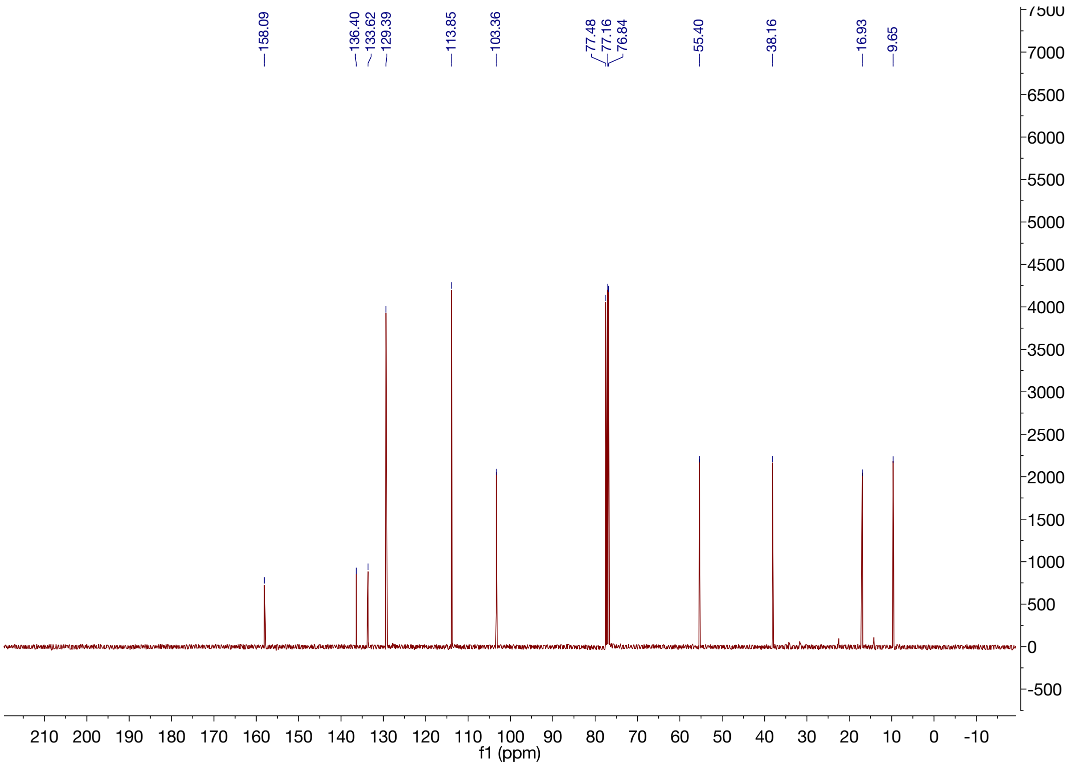

Figure S6. ^{13}C NMR of **1d** in $CDCl_3$.

Methylenecyclopropanes **1a** and **1f** were synthesized in similar fashion to **1d**, and have been synthesized previously.[8,9] In our hands, after methylation of these aliphatic 1,1-dibromocyclopropanes to their corresponding 1-bromo-1-methylcyclopropane derivatives, removal of the 1-iodobutane byproduct in vacuo on the Schlenk line (30 °C) significantly simplified isolation of the final MCP products. Performing the subsequent KO^{t}Bu elimination step with these 1-bromo-1-methylcyclopropane derivatives (after removal of 1-iodobutane) allowed for isolation of the pure corresponding MCP after via a simple pentane or hexanes extraction upon aqueous workup. NMR data for **1a** and **1e** is depicted below, and matched that of the literature.[8,9]

1-butyl-2-methylenecyclopropane (1a)

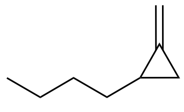

^{1}H NMR (400 MHz, $CDCl_3$, 25 °C, δ, ppm): 5.39 (s, 1H, =CH-*H*), 5.33 (s, 1H, =CH-*H*), 1.46-1.16 (m, 8H), 0.90 (t, *J* = 7.0 Hz, 3H), 0.74-0.68 (m, 1H).

^{13}C NMR (101 MHz, $CDCl_3$, 25 °C, δ, ppm): 137.5, 102.4, 33.0, 31.8, 22.6, 16.0, 14.2, 9.5.

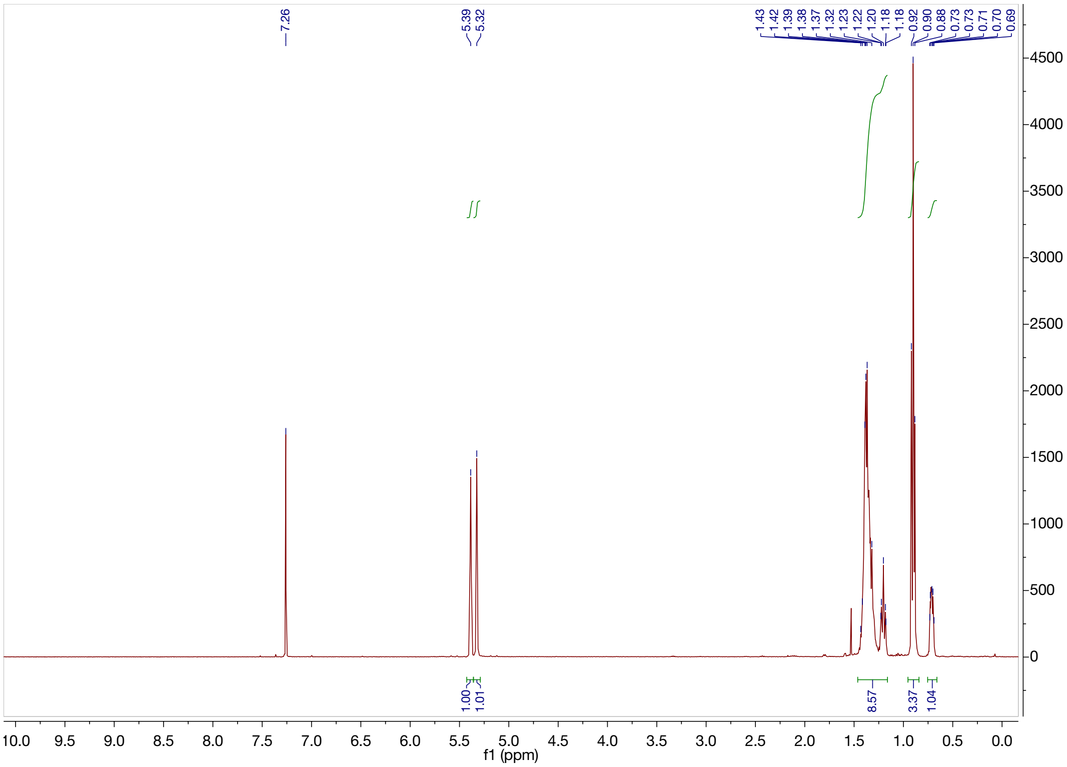

Figure S7. ^{1}H NMR of **1a** in $CDCl_3$.

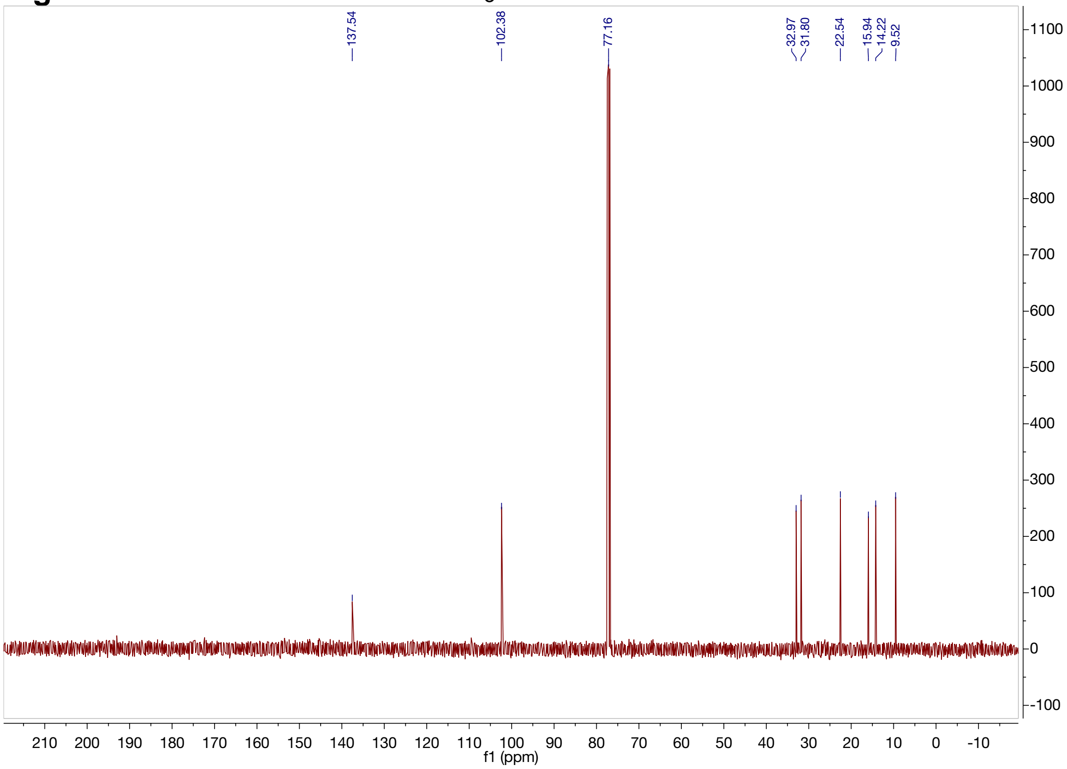

Figure S8. ^{13}C NMR of **1a** in $CDCl_3$.

9-methylenebicyclo[6.1.0]nonane (1f)

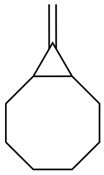

^{1}H NMR (400 MHz, $CDCl_3$, 25 °C, δ, ppm): 5.30 (s, 2H, =CH-*H*), 2.01-1.95 (m, 2H), 1.76-1.51 (m, 4H), 1.47-1.30 (m, 6H), 1.09-0.99 (m, 2H).

^{13}C NMR (101 MHz, $CDCl_3$, 25 °C, δ, ppm): 142.2, 101.3, 29.5, 26.6, 25.8, 19.5.

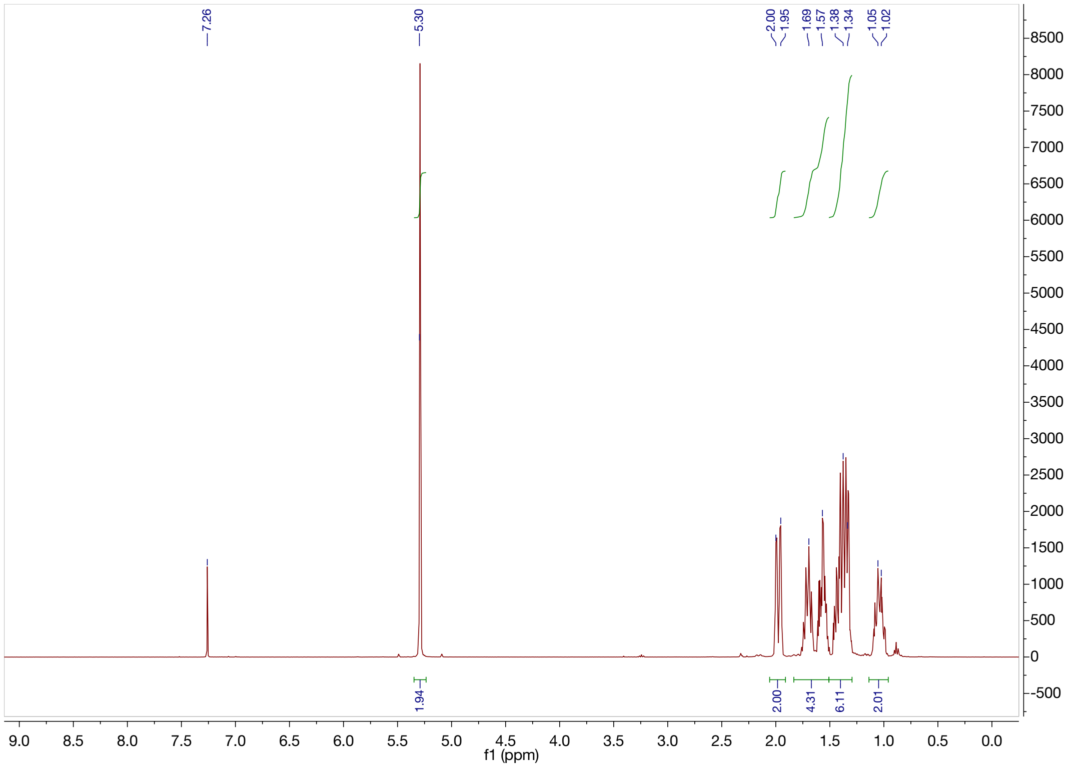

Figure S9. ^{1}H NMR of **1f** in $CDCl_3$.

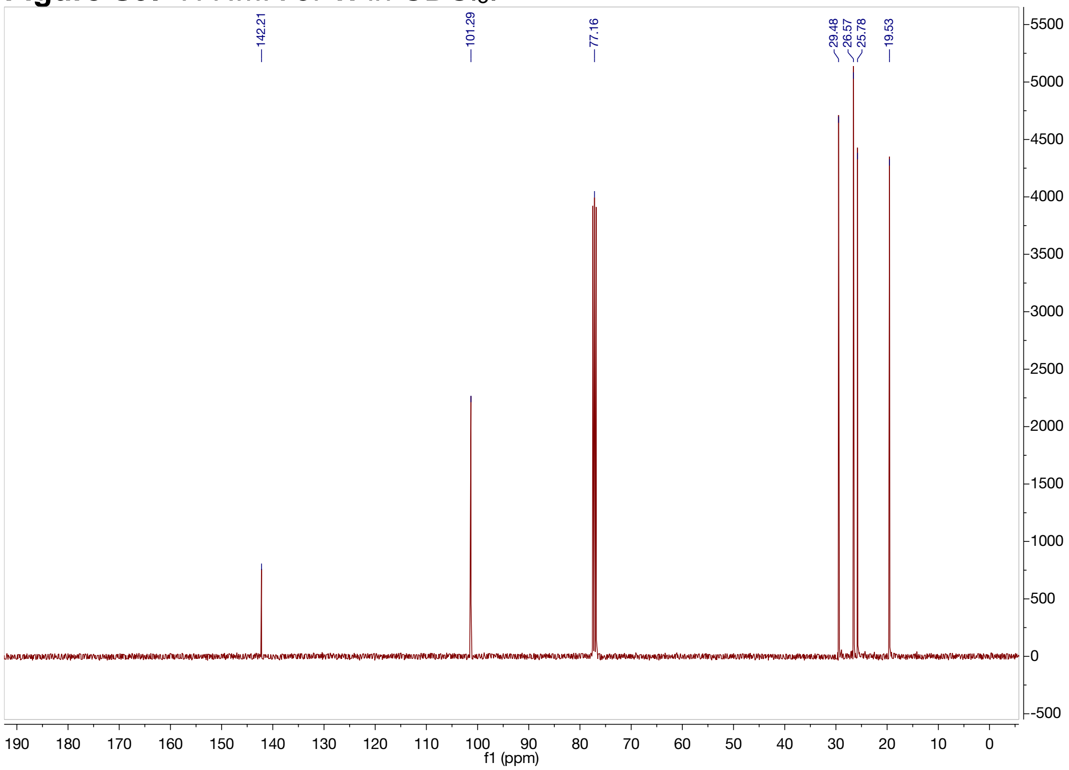

Figure S10. ^{13}C NMR of **1f** in $CDCl_3$.

Catalytic Synthesis of Unsaturated Imine Products

Synthesis of (*E*)-3-Methylene-*N*-phenylheptan-2-imine (2a):

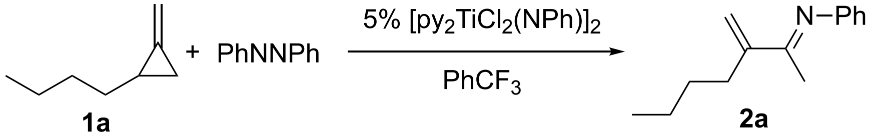

In a nitrogen glovebox, $[(py)_2TiCl_2(NPh)]_2$ (276 mg, 0.375 mmol, 0.05 equiv, 0.0250 M in Ti), azobenzene (1.37 g, 7.50 mmol, 1 equiv, 0.251 M), **1a** (1.82 g, 16.5 mmol, 2.2 equiv, 0.551 M), and $PhCF_3$ (30 mL) were added to a dried 50 mL Schlenk flask containing a stir bar. The flask was equipped with a reflux condenser and sealed with a rubber septum. The reaction flask was then removed from the glovebox, placed under nitrogen, and heated to 115 °C in an oil bath. After 62 h, the reaction mixture was cooled to room temperature, and was filtered through a pad of basic alumina using CH_2Cl_2 as eluent. The filtrate was concentrated in vacuo, and the residual oil was purified via short-path distillation under dynamic vacuum, providing **2a** as an orange oil (2.32 g, 77% in 88% purity; impurities include 10% of the corresponding hydroamination byproduct and 2% azobenzene, 68% corrected yield). Azobenzene and the hydroamination byproduct codistilled with **2a**.

^{1}H NMR (400 MHz, $CDCl_3$, 25 °C, δ, ppm): 7.30 (t, *J* = 7.6 Hz, 2H, Ar-C-*H*), 7.04 (t, *J* = 7.4 Hz, 1H, Ar-C-*H*), 6.68 (d, *J* = 8.4 Hz, 2H, Ar-C-*H*), 5.66 (s, 1H, =CH-*H*), 5.50 (s, 1H, =CH-*H*), 2.54-2.46 (m, 2H, $C(=CH_2)CH_2$), 1.96 (s, 3H, $C(NAr)CH_3$), 1.56-1.49 (m, 2H, $C(=CH_2)CH_2CH_2$), 1.42-1.33 (m, 2H, $CH_2CH_2CH_3$), 0.93 (t, *J* = 7.3 Hz, 3H, CH_2CH_3).

^{13}C NMR (101 MHz, $CDCl_3$, 25 °C, δ, ppm): 166.3, 152.0, 150.3, 129.0, 123.0, 119.2, 118.8, 32.0, 31.2, 22.8, 16.8, 14.2.

GC-HRMS: Calc. for $C_{14}H_{19}N$ [M^+] 201.1517; found 201.1521.

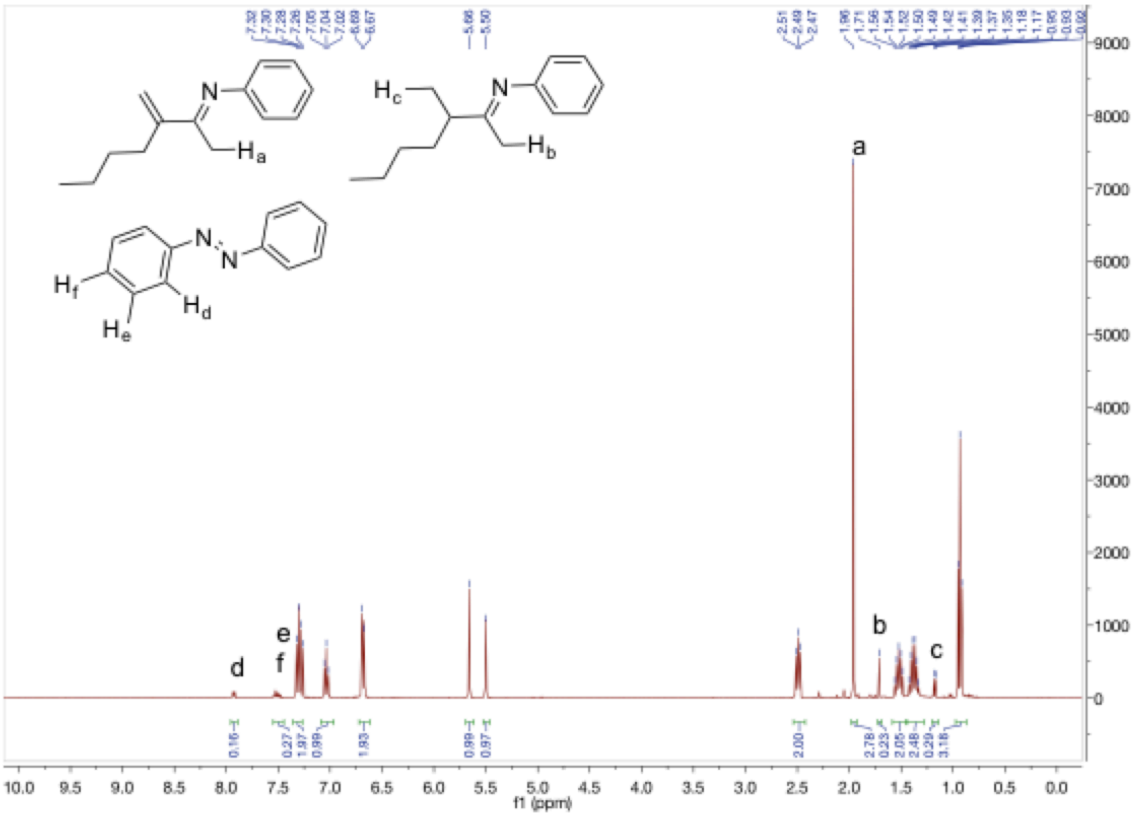

Figure S11. ^{1}H NMR of **2a** in $CDCl_3$.

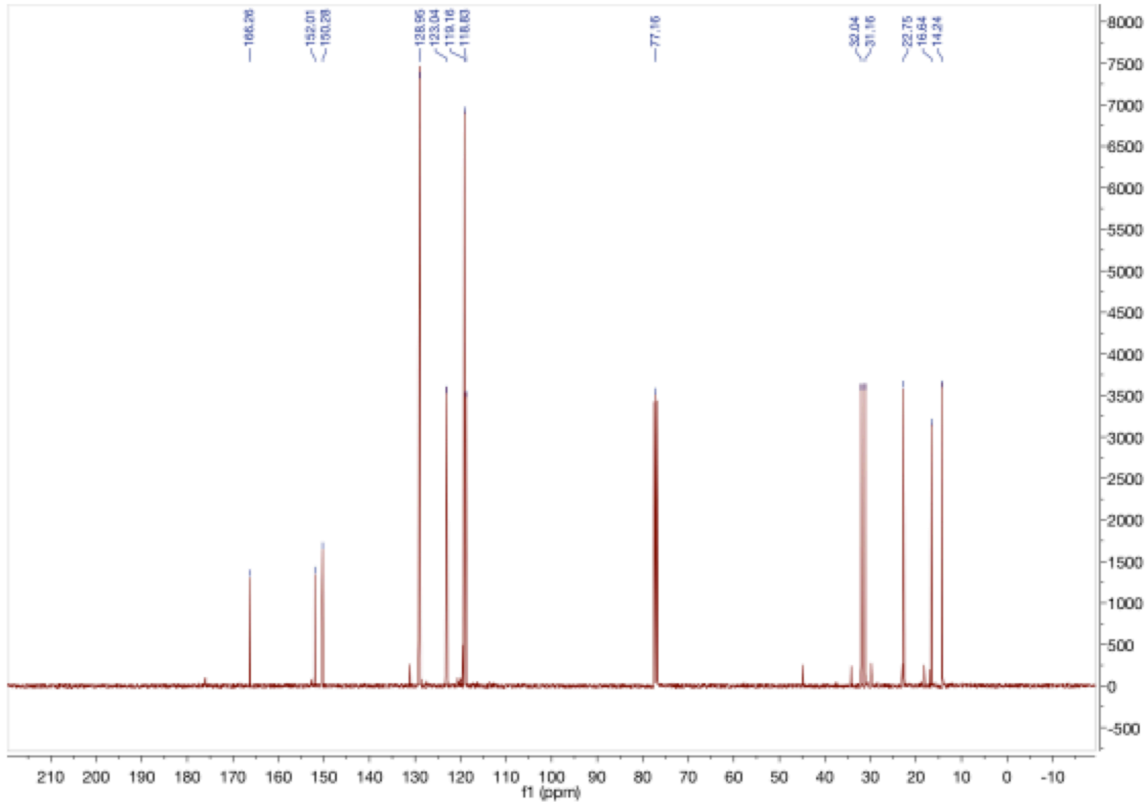

Figure S12. ^{13}C NMR of **2a** in $CDCl_3$.

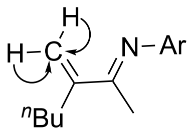

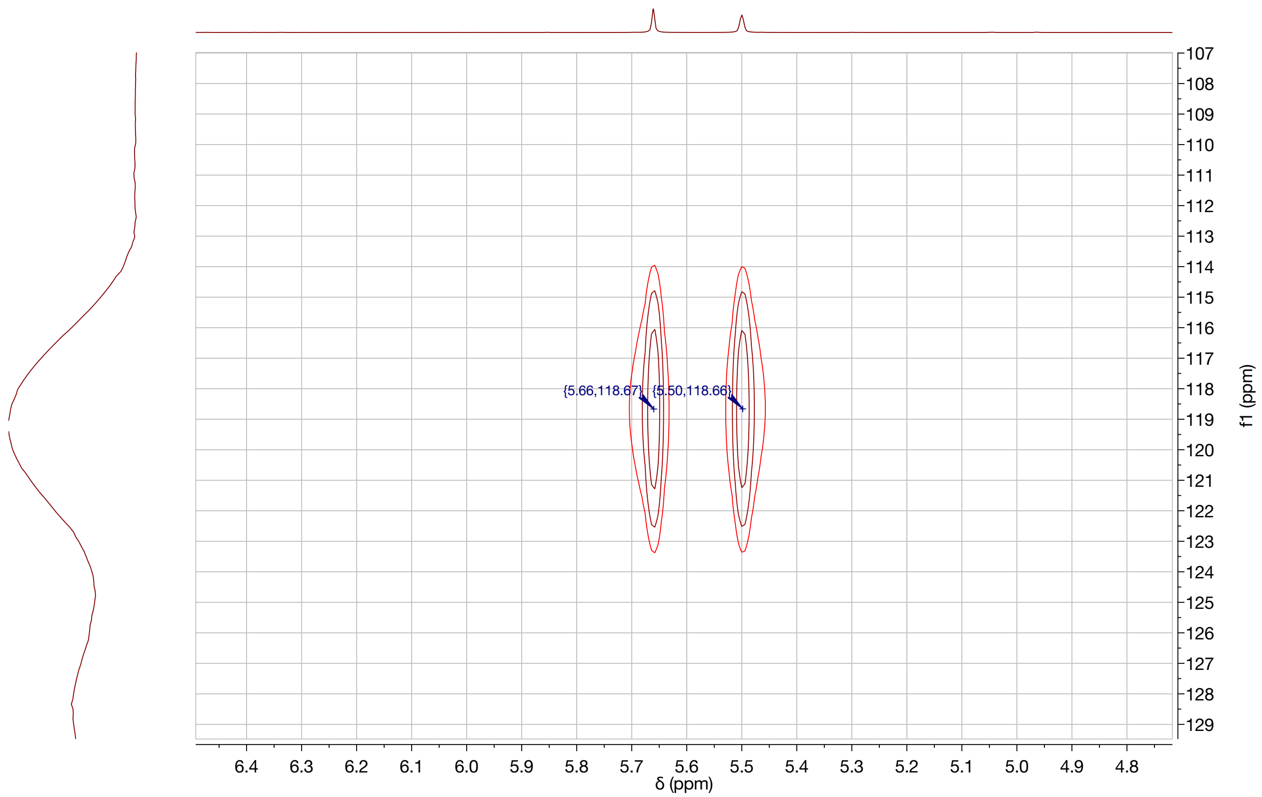

Figure S13. ^{1}H-^{13}C HSQC of **2a** in $CDCl_3$ zoomed in on the $=CH_2$ region (4.8-6.4 ppm).

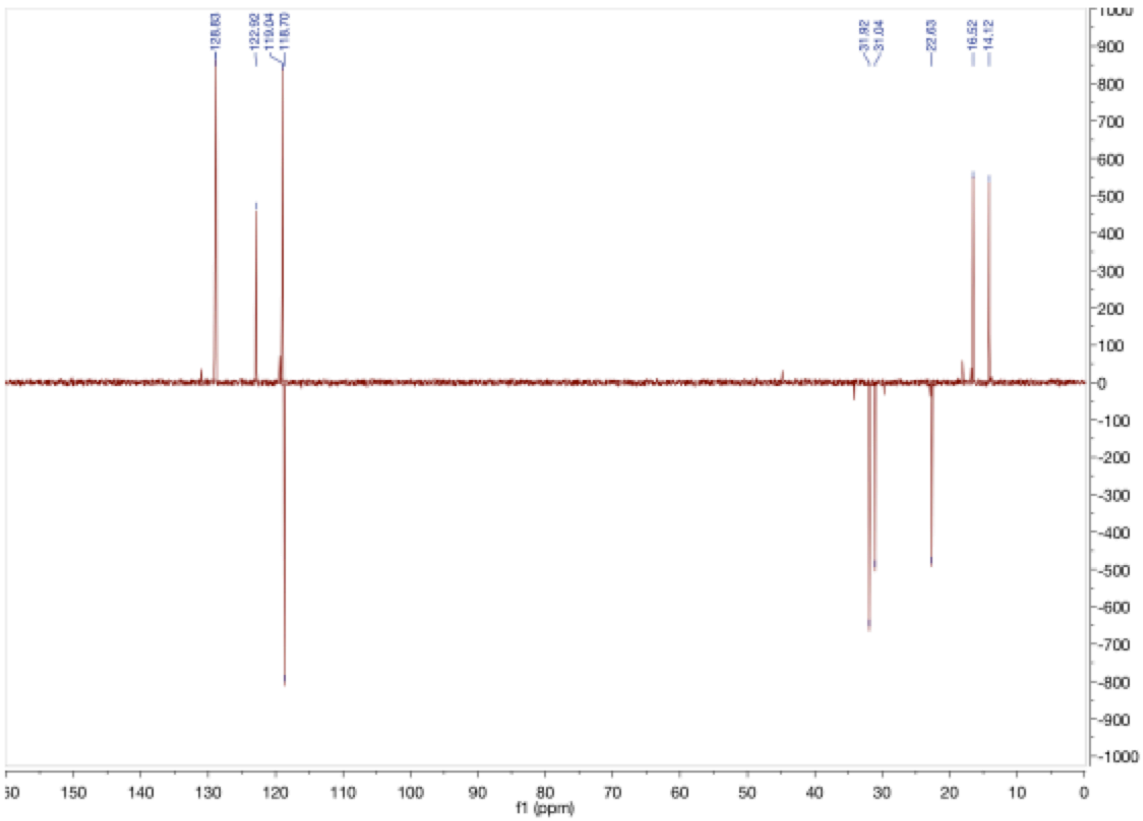

Figure S14. DEPT-135 NMR of **2a** in $CDCl_3$.

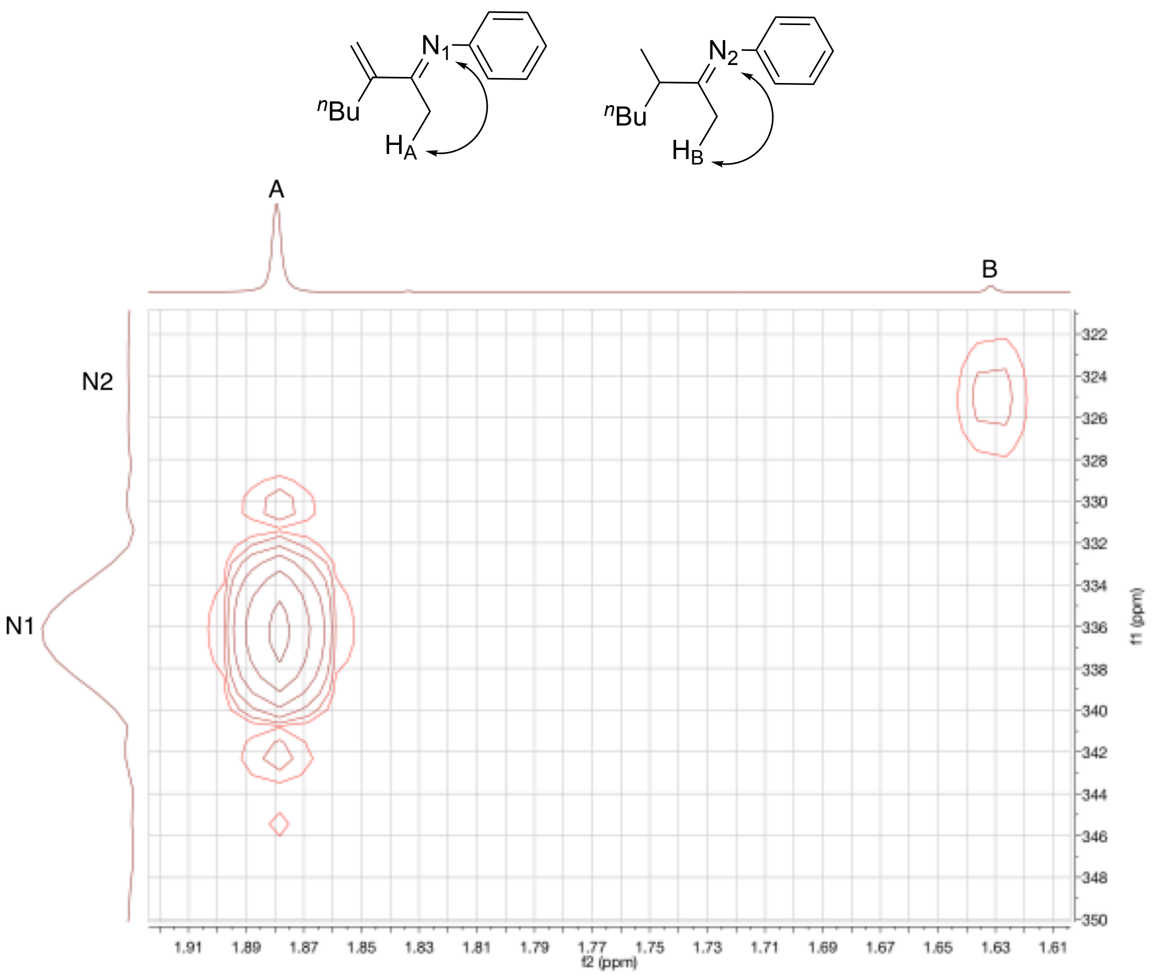

Figure S15. ^{1}H-^{15}N HMBC of **2a** and the hydroamination byproduct in $CDCl_3$ (1.6-1.9 ppm).

Synthesis of (*E*)-3-methylene-*N*-(*p*-tolyl)heptan-2-imine (2a-tol):

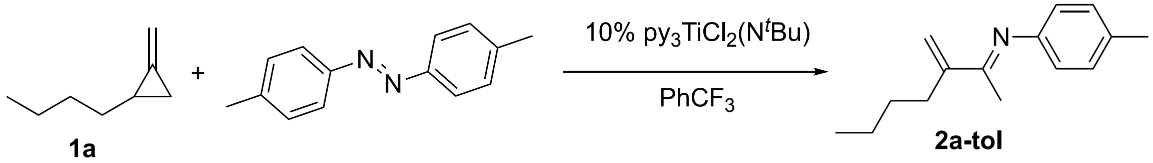

In a nitrogen glovebox, $(py)_3TiCl_2(N^tBu)$ (132 mg, 0.310 mmol, 0.1 equiv, 0.0618 M), 1,2-di-*p*-tolyldiazene (650 mg, 3.10 mmol, 1 equiv, 0.0618 M), **1a** (1.36 g, 12.4 mmol, 4 equiv, 2.47 M), and $PhCF_3$ (5 mL) were added to a 20 mL scintillation vial containing a stir bar. The vial was sealed with a Teflon cap, removed from the glove box and then heated to 115 °C in an oil bath. After 62 h, the reaction mixture was cooled to room temperature, and was filtered through a pad of alumina using CH_2Cl_2 as eluent. The filtrate was concentrated in vacuo, and the residual oil was purified via short-path distillation under a dynamic vacuum, providing **2a-tol** as an orange oil (860 mg, 65% in 92% purity; impurities include 8% of the corresponding hydroamination byproduct, 60% corrected yield). The hydroamination byproduct codistilled with **2a-tol**.

^{1}H NMR (400 MHz, $CDCl_3$, 25 °C, δ, ppm): 7.11 (d, *J* = 7.9 Hz, 2H, Ar-C-*H*), 6.59 (d, *J* = 8.2 Hz, 2H, Ar-C-*H*), 5.64 (s, 1H, =CH-*H*), 5.48 (s, 1H, =CH-*H*), 2.51 – 2.47 (m, 2H, $C(=CH_2)CH_2$), 2.32 (s, 3H, *p*-CH_3Ph), 1.96 (s, 3H, C(NAr)CH_3), 1.58 – 1.48 (m, 2H, $C(=CH_2)CH_2CH_2$), 1.42-1.33 (m, 2H, $CH_2CH_2CH_3$), 0.93 (t, *J* = 7.3 Hz, 3H, CH_2CH_3).

^{13}C NMR (101 MHz, $CDCl_3$, 25 °C, δ, ppm): 166.3, 150.4, 149.4, 132.4, 129.5, 119.2, 118.6, 77.5, 77.2, 76.8, 32.1, 31.2, 22.8, 21.0, 16.6, 14.3.

GC-HRMS: Calc. for $C_{15}H_{21}N$ [M^+] 215.1674; found 215.1663.

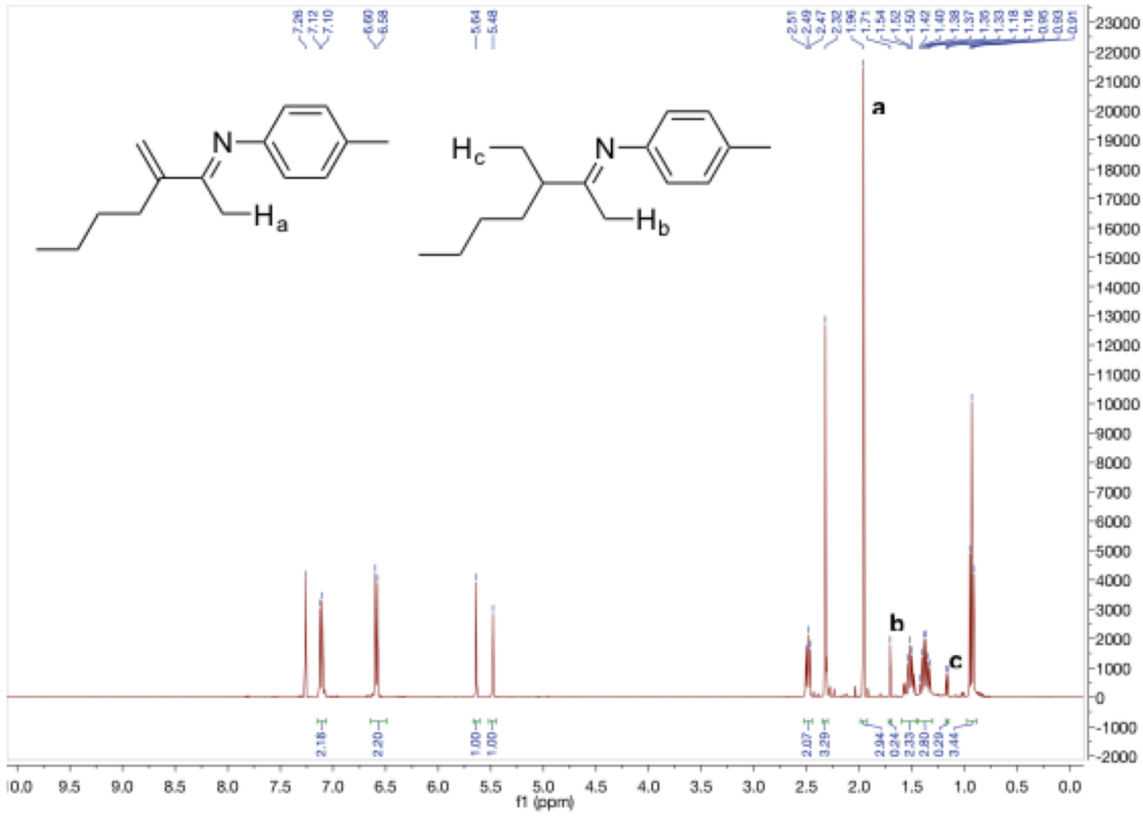

Figure S16. ^{1}H NMR of **2a-tol** in $CDCl_3$.

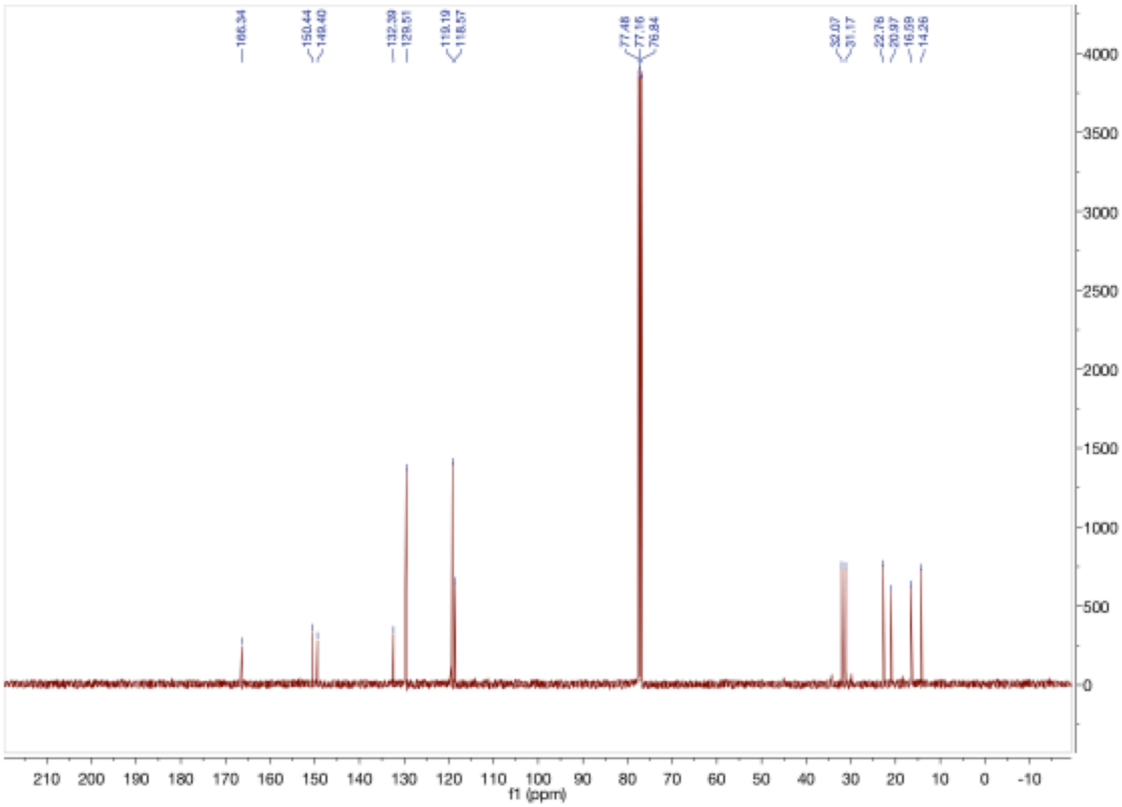

Figure S17. ^{13}C NMR of **2a-tol** in $CDCl_3$.

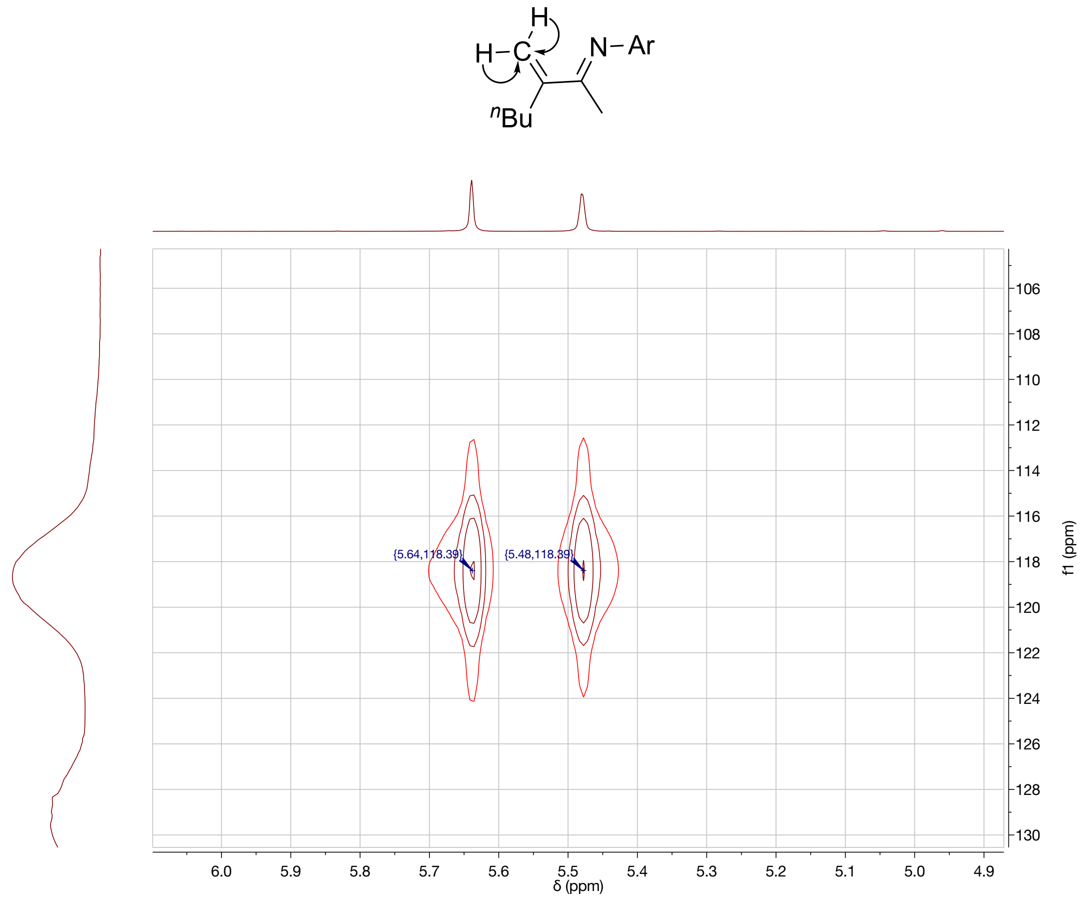

Figure S18. ^{1}H-^{13}C HSQC of **2a-tol** in $CDCl_3$ zoomed in on the $=CH_2$ region (4.9-6.0 ppm).

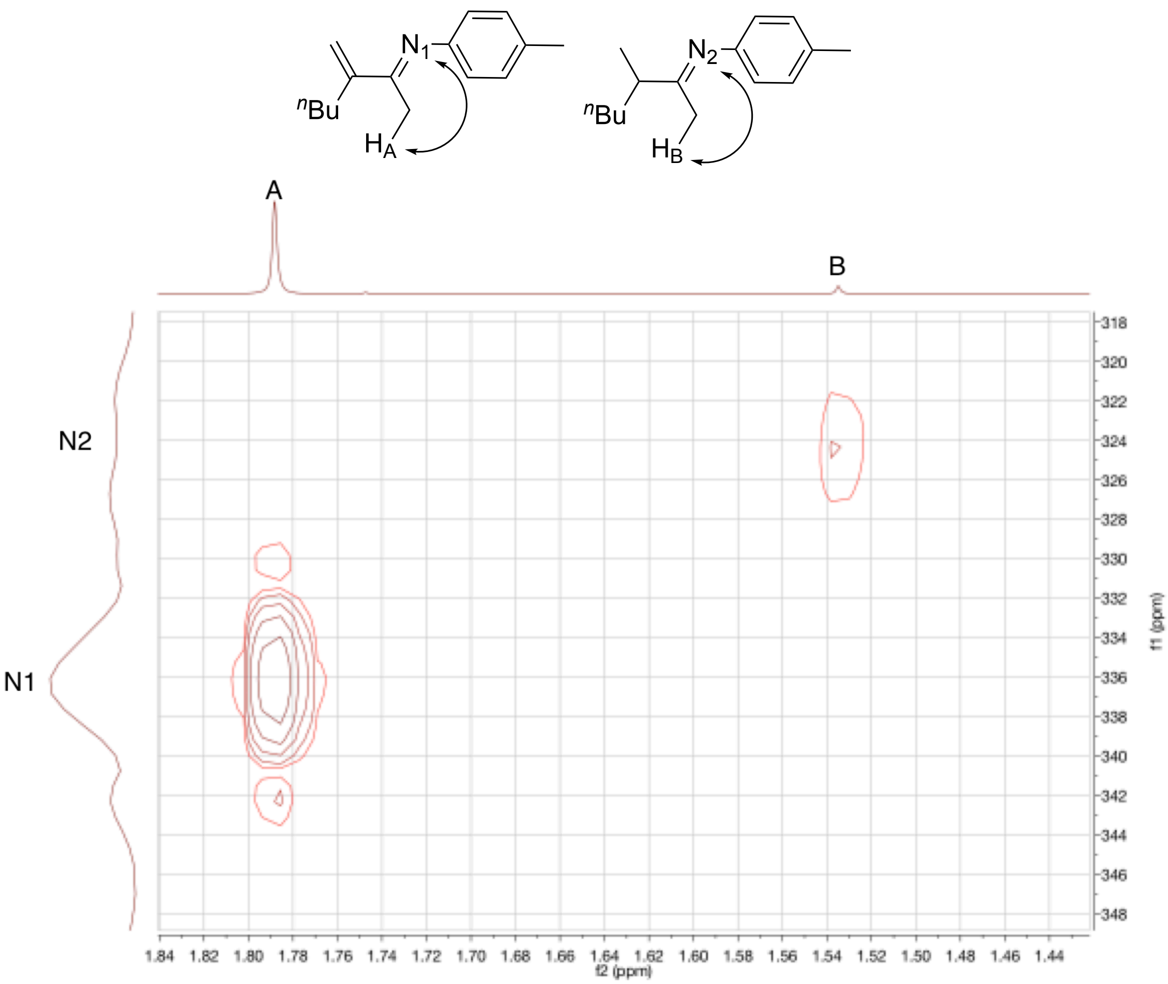

Figure S19. ^{1}H-^{15}N HMBC of **2a-tol** and the hydroamination byproduct in $CDCl_3$ (1.4-1.8 ppm).

Synthesis of (*E*)-3-methylene-*N*-(4-(trifluoromethoxy)phenyl)heptan-2-imine (2a-OCF$_3$):

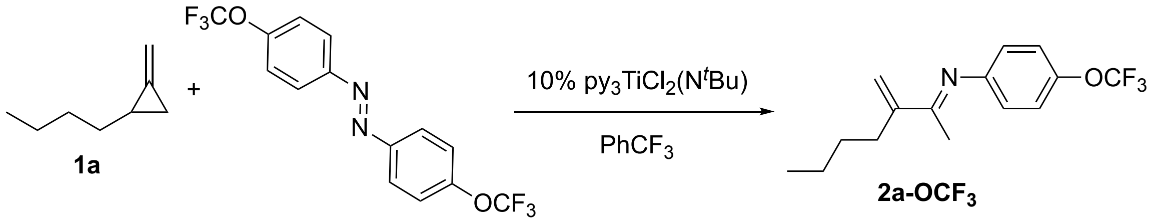

1a **2a-OCF$_3$**

In a nitrogen glovebox, $(py)_3TiCl_2(N^tBu)$ (86 mg, 0.20 mmol, 0.1 equiv, 0.0402 M), 1,2-bis(4-(trifluoromethoxy)phenyl)diazene (700 mg, 2.00 mmol, 1 equiv, 0.400 M), **1a** (881 mg, 8.00 mmol, 4 equiv, 1.60 M), and $PhCF_3$ (5 mL) were added to a 20 mL scintillation vial containing a stir bar. The vial was sealed with a Teflon cap, removed from the glove box and heated to 115 °C in an oil bath. After 62 h, the reaction mixture was cooled to room temperature, and was filtered through a pad of basic alumina using CH_2Cl_2 as eluent. The filtrate was concentrated in vacuo, and the residual oil was purified via short-path distillation under a dynamic vacuum, providing **2a-OCF$_3$** as a yellow oil (730 mg, 64% in 93% purity; impurities include 7% of the corresponding hydroamination byproduct, 59% corrected yield). The hydroamination byproduct codistilled with **2a-OCF$_3$**.

^{1}H NMR (400 MHz, $CDCl_3$, 25 °C, δ, ppm): 7.16 (d, *J* = 8.0 Hz, 2H, Ar-C-*H*), 6.68 (d, *J* = 8.9 Hz, 2H, Ar-C-*H*), 5.68 (s, 1H, =CH-*H*), 5.53 (s, 1H, =CH-*H*), 2.49 – 2.45 (m, 2H, C(=CH$_2$)C*H*$_2$), 1.97 (s, 3H, C(NAr)C*H*$_3$), 1.55 – 1.47 (m, 2H, C(=CH$_2$)CH$_2$C*H*$_2$), 1.42 – 1.33 (m, 2H, CH$_2$C*H*$_2$CH$_3$), 0.93 (t, *J* = 7.3 Hz, 3H, -CH$_2$C*H*$_3$).

^{13}C NMR (101 MHz, $CDCl_3$, 25 °C, δ, ppm): 167.2, 150.7, 150.1, 145.1, 121.9, 121.3 (q, $J_{C\text{-}F}$ = 151.5 Hz), 120.2, 119.5, 31.0, 31.2, 22.8, 16.7, 14.2.

^{19}F{^{1}H} NMR (376 MHz, $CDCl_3$, 25 °C, δ, ppm): -57.1.

GC-HRMS: Calc. for $C_{15}H_{18}F_3NO$ [M$^+$] 285.1340; found 285.1336.

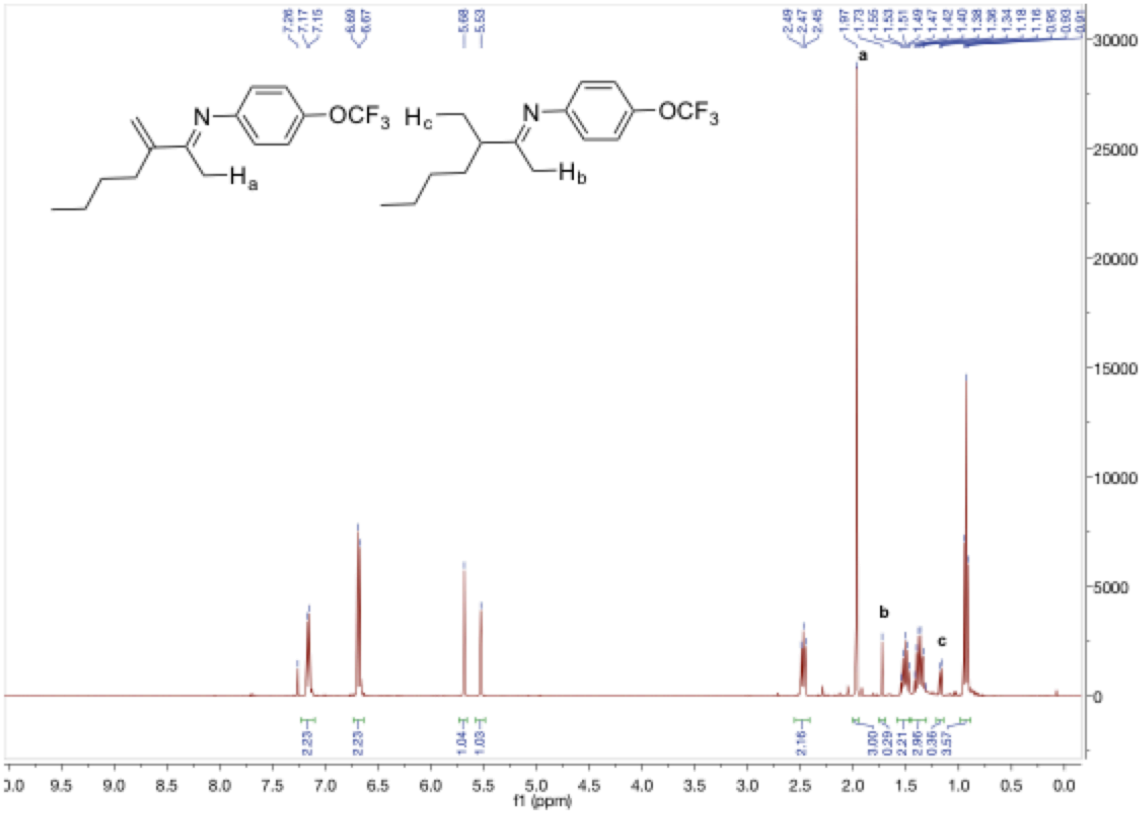

Figure S20. ^{1}H NMR of **2a-OCF$_3$** in $CDCl_3$.

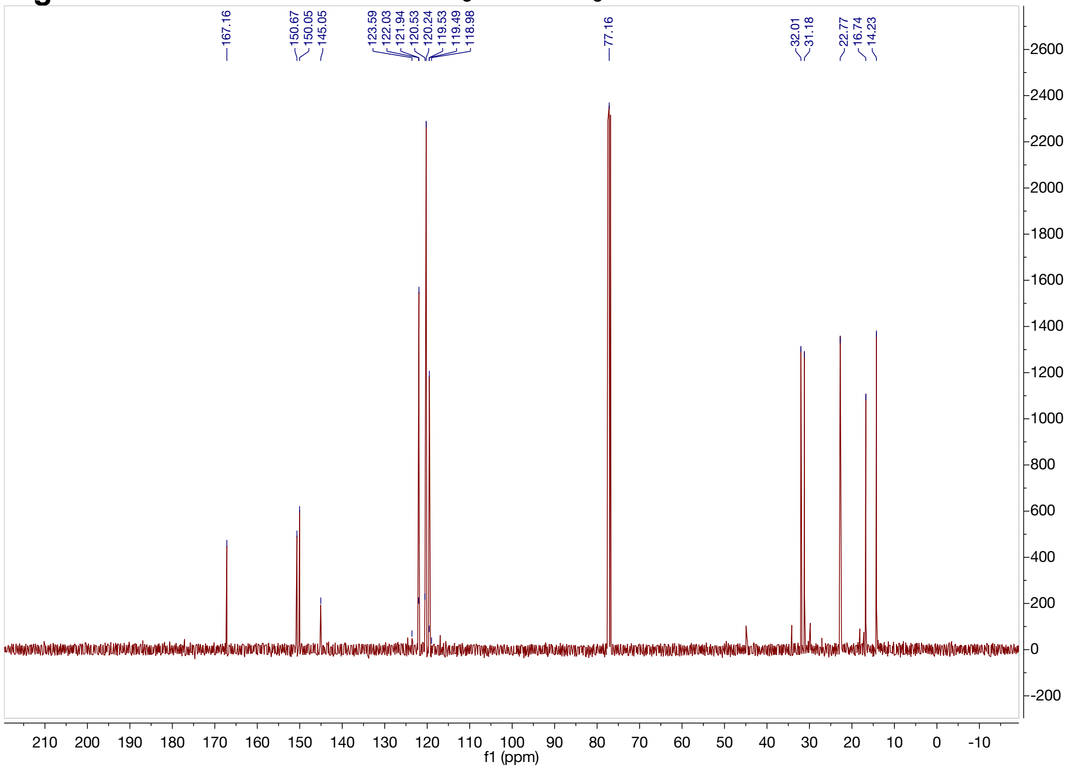

Figure S21. ^{13}C NMR of **2a-OCF$_3$** in $CDCl_3$.

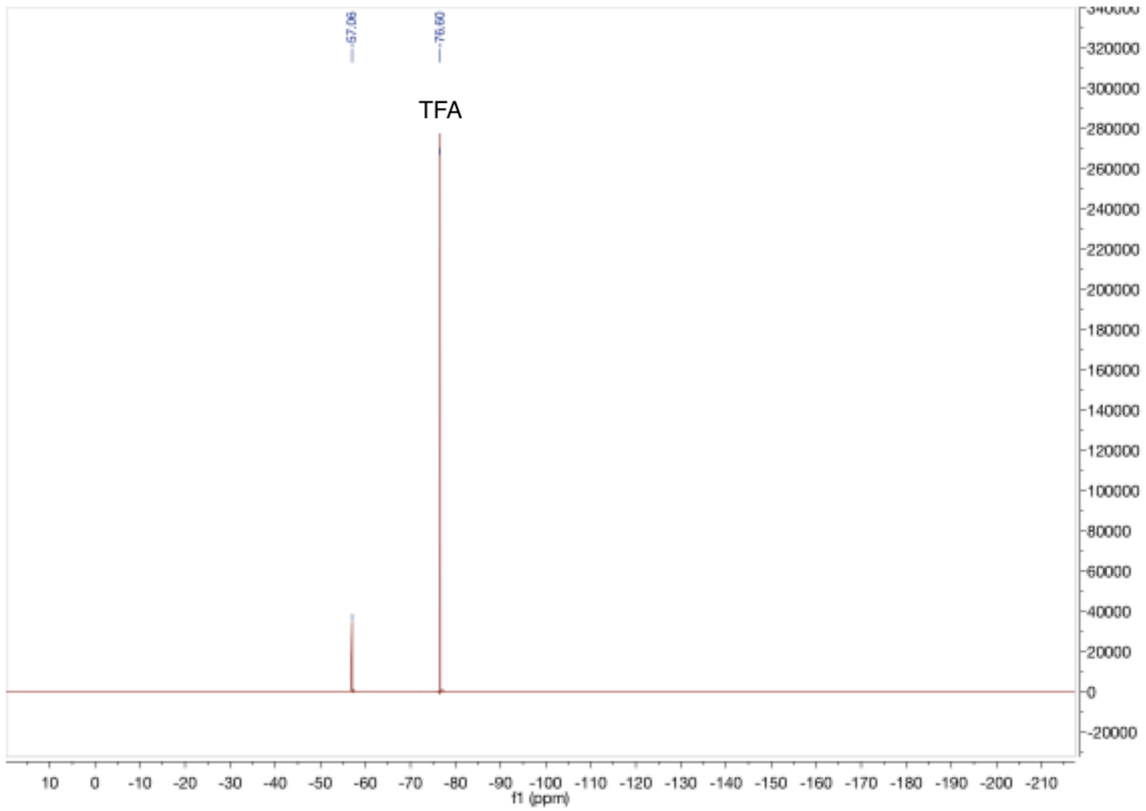

Figure S22. $^{19}F\{^{1}H\}$ NMR of **2a-OCF$_3$** in $CDCl_3$ (TFA used as internal reference).

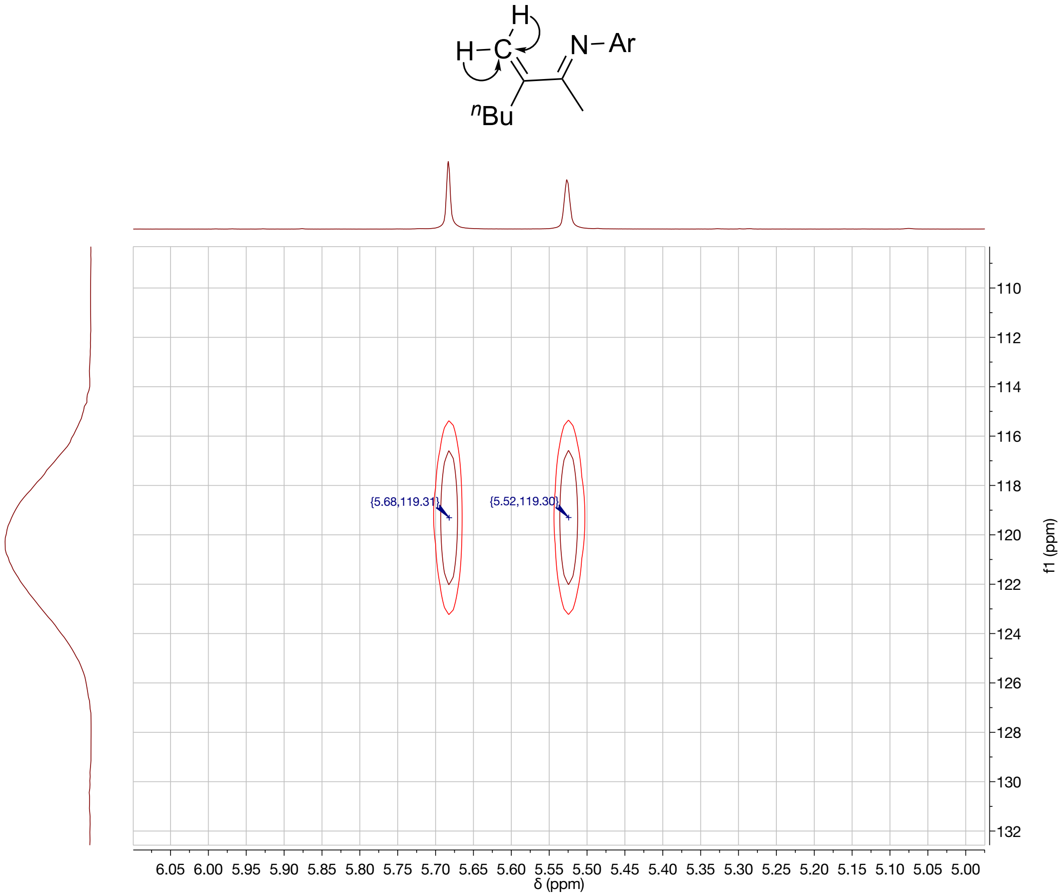

Figure S23. ^{1}H-^{13}C HSQC of **2a-OCF$_3$** in $CDCl_3$ zoomed in on the $=CH_2$ region (5.0-6.0 ppm).

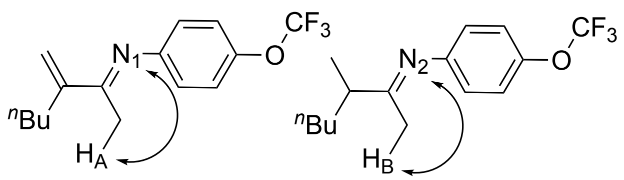

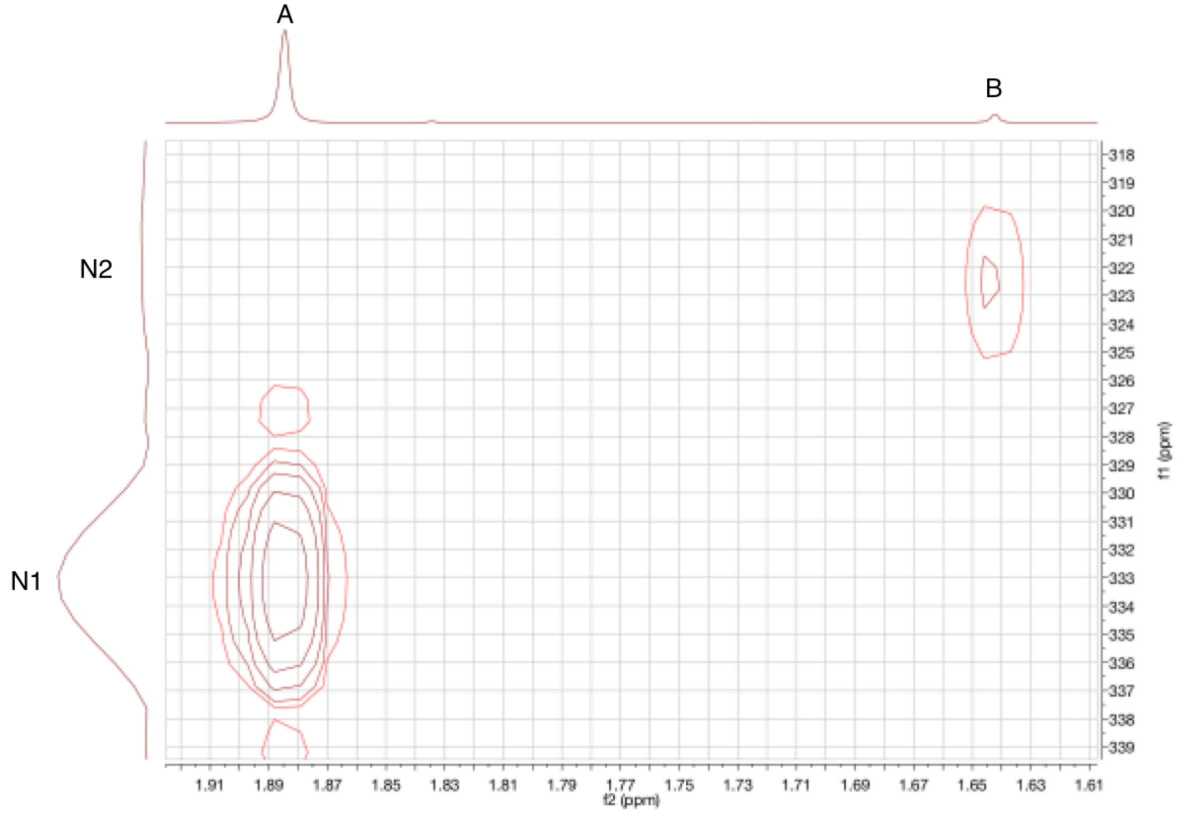

Figure S24. ^{1}H-^{15}N HMBC of **2a-OCF₃** and the hydroamination byproduct in $CDCl_3$ (1.6-1.9 ppm).

Synthesis of 3-methylenenonan-2-one (2c):

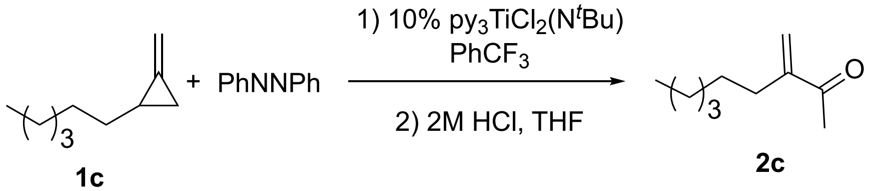

In a nitrogen glovebox, $(py)_3TiCl_2(N^tBu)$ (47 mg, 0.11 mmol, 0.1 equiv, 0.0220 M), azobenzene (200 mg, 1.10 mmol, 1 equiv, 0.220 M), **1c** (334 mg, 2.41 mmol, 2.2 equiv, 0.483 M), and $PhCF_3$ (5 mL) were added to a 20 mL scintillation vial containing a stir bar. The vial was sealed with a Teflon cap, then removed from the glove box and heated to 115 °C in an oil bath. After 62 h, the reaction mixture was cooled to room temperature, then THF (5 mL) and aq. HCl (5 mL, 2 M) were added to the reaction mixture. After stirring for 16 h at room temperature, the reaction was diluted with water (50 mL), and extracted with Et_2O (3 x 30 mL). The combined organic phases were washed with water (1 x 50 mL) and brine (1 x 30 mL), and then dried over $MgSO_4$. The solution was filtered and then concentrated in vacuo. Final purification via column chromatography (silica, 5% EtOAc/hexanes) afforded **2c** (260 mg, 77%) as an orange oil. The spectral data matched that of the reference.[10]

^1H NMR (400 MHz, $CDCl_3$, 25 °C, δ, ppm): 5.99 (s, 1H, =CH-*H*), 5.74 (s, 1H, =CH-*H*), 2.33 (s, 3H, C(O)C*H*$_3$), 2.29-2.20 (m, 2H, C(=CH$_2$)C*H*$_2$), 1.46-1.20 (m, 8H), 0.88 (t, J = 7.2 Hz, 3H, -CH$_2$C*H*$_3$).

^{13}C NMR (101 MHz, $CDCl_3$, 25 °C, δ, ppm): 200.1, 149.6, 124.8, 31.8, 30.7, 29.2, 28.5, 26.1, 22.8, 14.2.

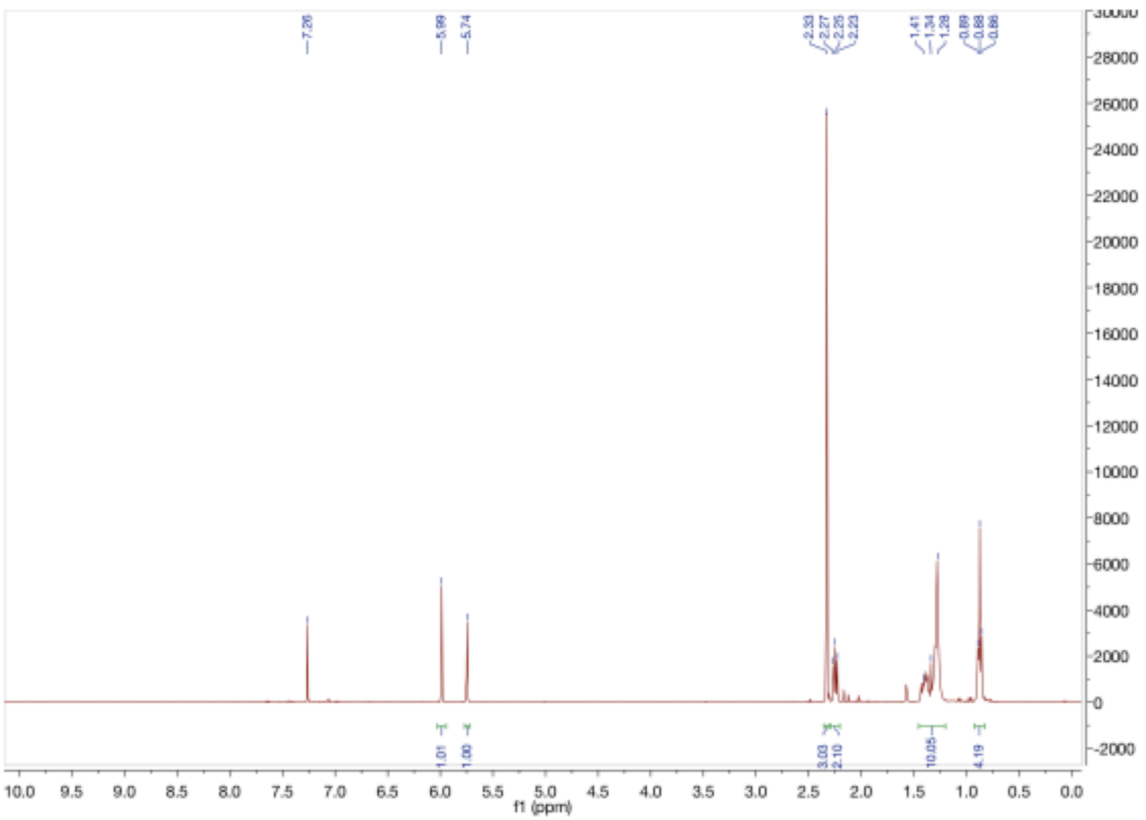

Figure S25. ^{1}H NMR of **2c** in $CDCl_3$.

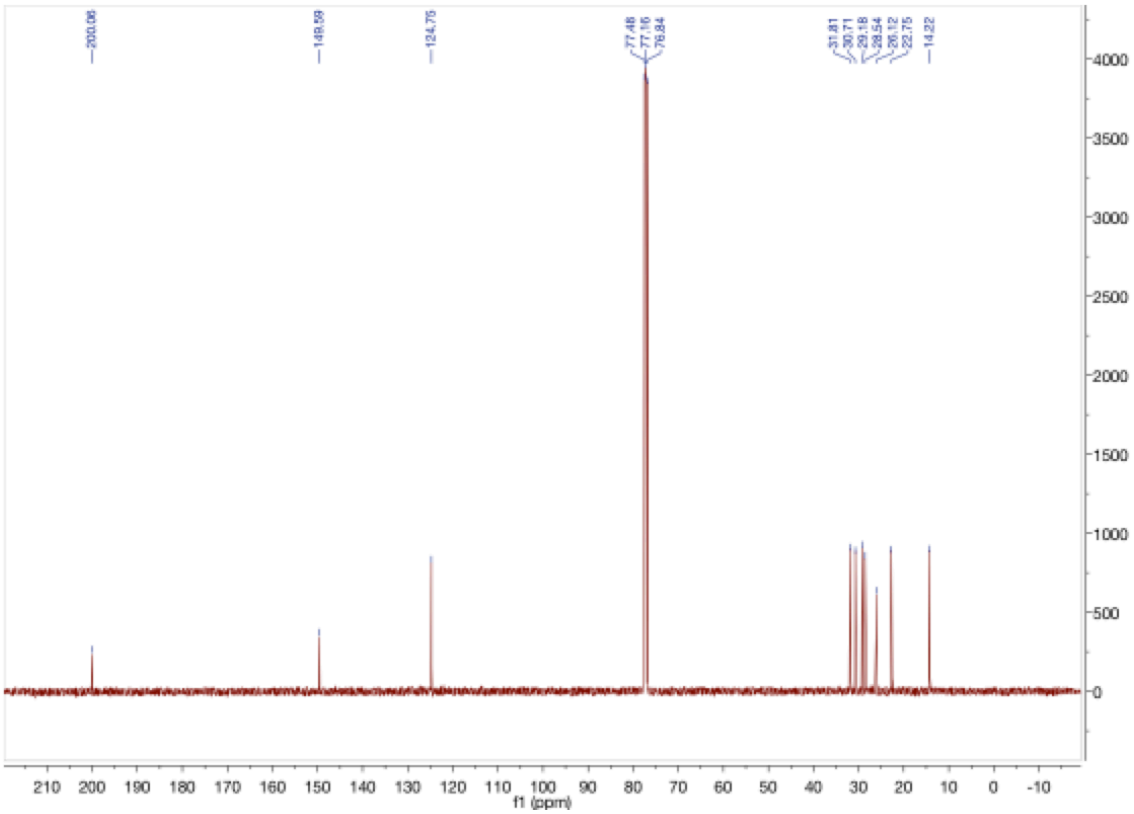

Figure S26. ^{13}C NMR of **2c** in $CDCl_3$.

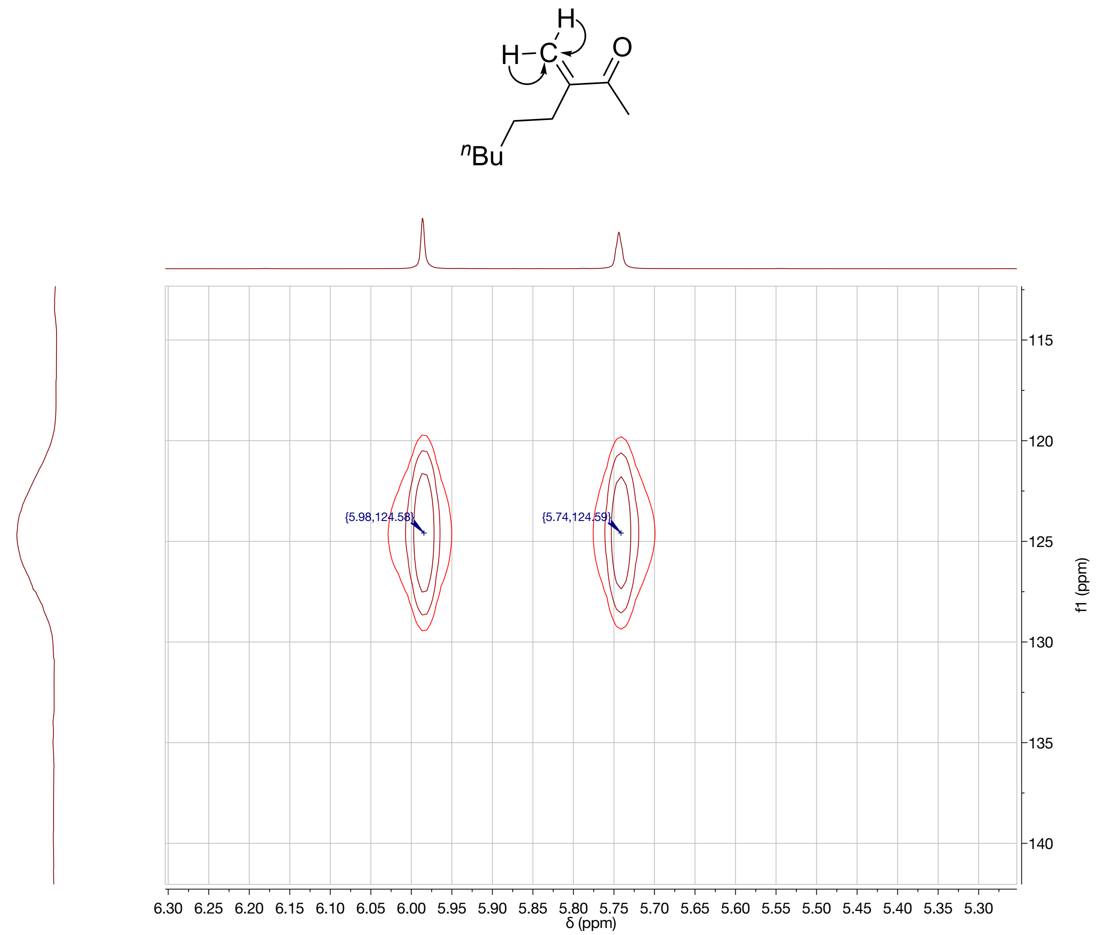

Figure S27. ^{1}H-^{13}C HSQC of **2c** in $CDCl_3$ zoomed in on the $=CH_2$ region (5.3-6.3 ppm).

Synthesis of 3-(4-methoxybenzyl)but-3-en-2-one (2d):

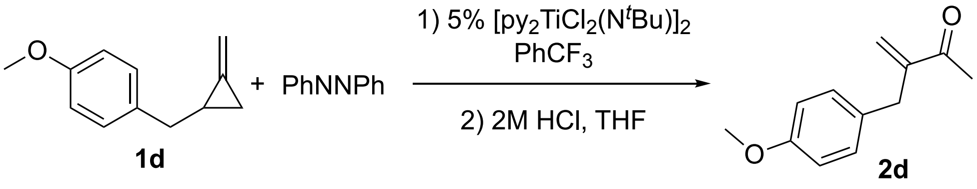

In a nitrogen glovebox, $[py_2TiCl_2(N^tBu)]_2$ (40 mg, 0.057 mmol, 0.05 equiv, 0.0192 M), azobenzene (200 mg, 1.09 mmol, 1 equiv, 0.183 M), **1d** (435 mg, 2.49 mmol, 2.3 equiv, 0.416 M), and $PhCF_3$ (6 mL) were added to a 20 mL scintillation vial containing a stir bar. The vial was sealed with a Teflon cap, then removed from the glove box and heated to 115 °C in an oil bath. After 62 h, the reaction mixture was cooled to room temperature, then THF (5 mL) and aq. HCl (5 mL, 2 M) were added to the reaction mixture. After stirring for 16 h at room temperature, the reaction was diluted with water (50 mL), and extracted with Et_2O (3 x 20 mL). The combined organic phases were washed with sat. Na_2CO_3 (1 x 50 mL), and brine (1 x 30 mL), then dried over $MgSO_4$. The solution was filtered and concentrated in vacuo. Final purification via column chromatography (silica, 5% Et_2O/hexanes), afforded **2d** (290 mg, 69% in 86% purity; impurities include 14% of the corresponding hydrolyzed hydroamination byproduct, 59% corrected yield) as a yellow oil. The hydrolyzed hydroamination byproduct coeluted with **2d**.

^1H NMR (400 MHz, $CDCl_3$, 25 °C, δ, ppm): 7.09 (d, J = 8.5 Hz, 2H, Ar-C-H), 6.83 (d, J = 8.6 Hz, 2H, Ar-C-H), 6.06 (s, 1H, =CH-H), 5.63 (s, 1H, =CH-H), 3.79 (s, 3H, OCH_3), 3.53 (s, 2H, Ph-CH_2-C=CH_2), 2.34 (s, 3H, C(O)CH_3).

^{13}C NMR (101 MHz, $CDCl_3$, 25 °C, δ, ppm): 199.4, 158.2, 149.2, 131.2, 130.2, 126.2, 114.0, 55.4, 36.0, 26.2.

GC-HRMS: Calc. for $C_{12}H_{14}O_2$ [M^+] 190.0994; found 190.0998.

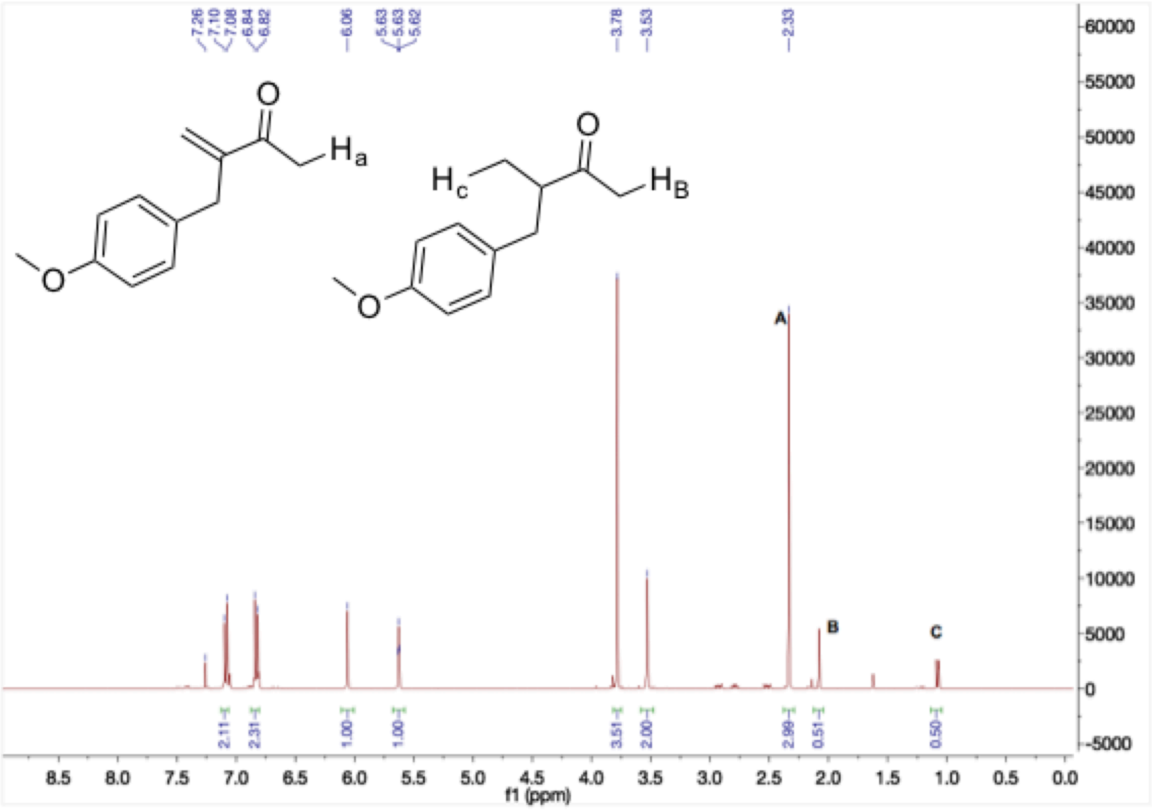

Figure S28. ^{1}H NMR of **2d** in $CDCl_3$.

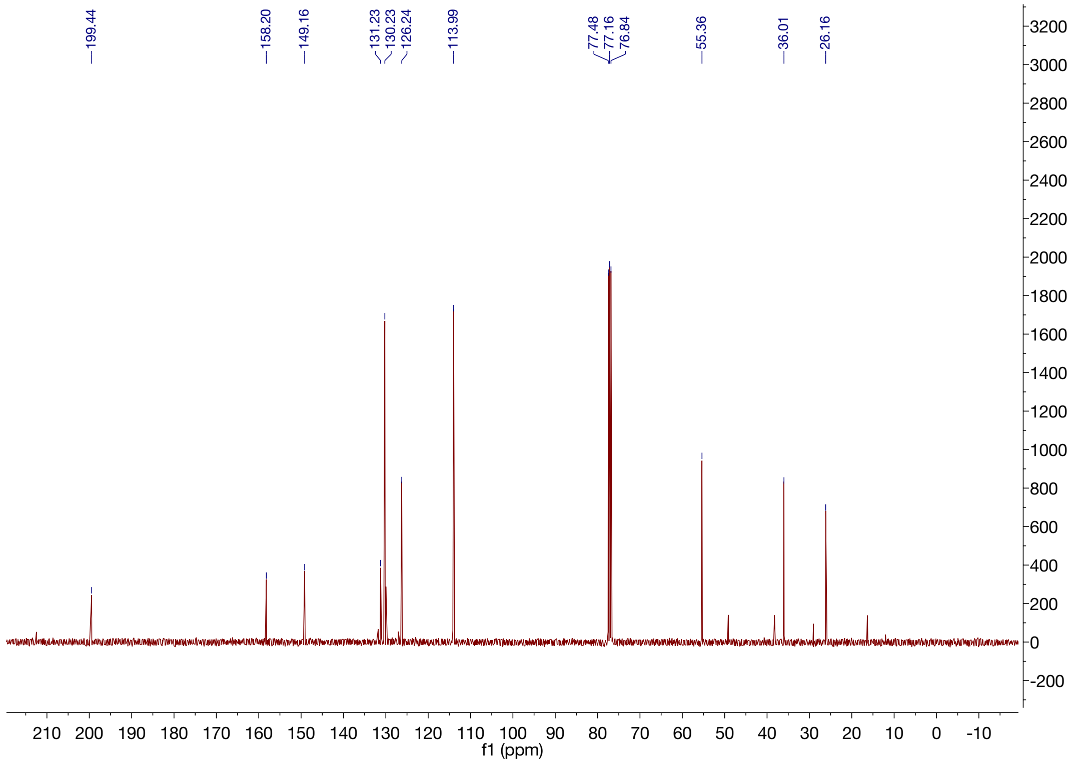

Figure S29. ^{13}C NMR of **2d** in $CDCl_3$.

Synthesis of (*E*)-1-(cyclohex-1-en-1-yl)-*N*-phenylethan-1-imine (2e):

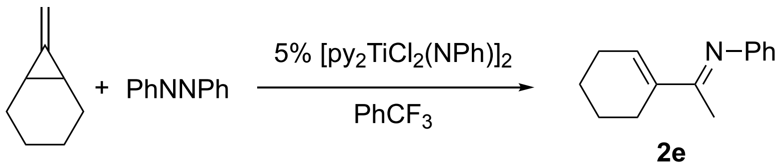

1e **2e**

In a nitrogen glovebox, $[py_2TiCl_2(NPh)]_2$ (276 mg, 0.375 mmol, 0.05 equiv, 0.0250 M in Ti), azobenzene (1.37 g, 7.50 mmol, 1 equiv, 0.251 M), **1e** (1.78 g, 16.5 mmol, 2.2 equiv, 0.548 M), and $PhCF_3$ (30 mL) were added to a 50 mL Schlenk flask containing a stir bar. The flask was equipped with a reflux condenser, and sealed with a rubber septum. The reaction flask was then removed from the glove box, placed under a flow of nitrogen, and heated to 115 °C in an oil bath. After 62 h, the reaction mixture was cooled to room temperature, and was filtered through a pad of basic alumina using CH_2Cl_2 as the eluent. The filtrate was concentrated in vacuo, and the residual oil was purified via short-path distillation under a dynamic vacuum, providing **2e** (2.01 g, 67%) as a light orange oil. Spectral data matched that of the reference.[11]

^{1}H NMR (400 MHz, $CDCl_3$, 25 °C, δ, ppm): 7.29 (t, *J* = 6.8 Hz, 2H, Ar-C-*H*), 7.02 (t, *J* = 7.5 Hz, 1H, Ar-C-*H*), 6.68 (d, *J* = 6.8 Hz, 2H, Ar-C-*H*), 6.54-6.52 (m, 1H, C=C-*H*), 2.45-2.43 (m, 2H, C=C-CH_2), 2.28-2.24 (m, 2H, C=C-CH_2), 1.93 (s, 3H, C(NAr)CH_3), 1.73-1.63 (m, 4H).

^{13}C NMR (101 MHz, $CDCl_3$, 25 °C, δ, ppm): 166.6, 152.3, 139.5, 133.8, 128.9, 122.8, 119.5, 26.4, 24.8, 22.7, 22.2, 15.9.

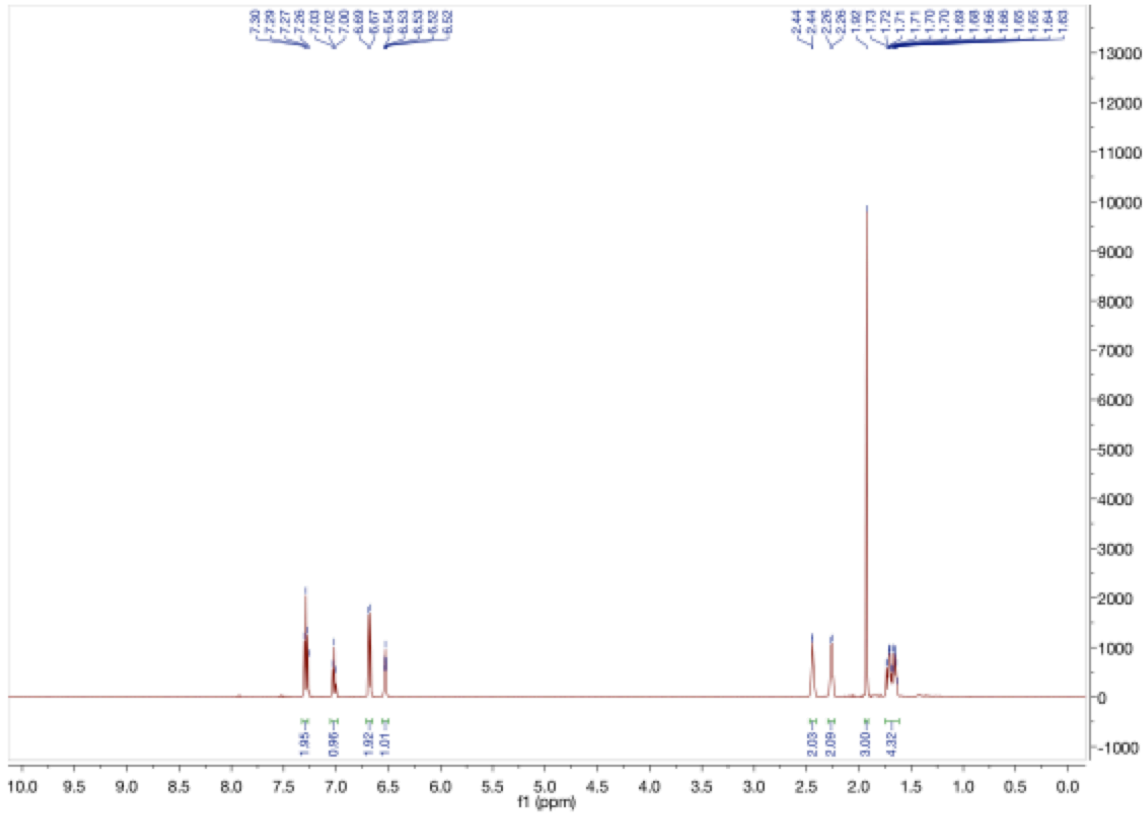

Figure S30. ^{1}H NMR of **2e** in $CDCl_3$.

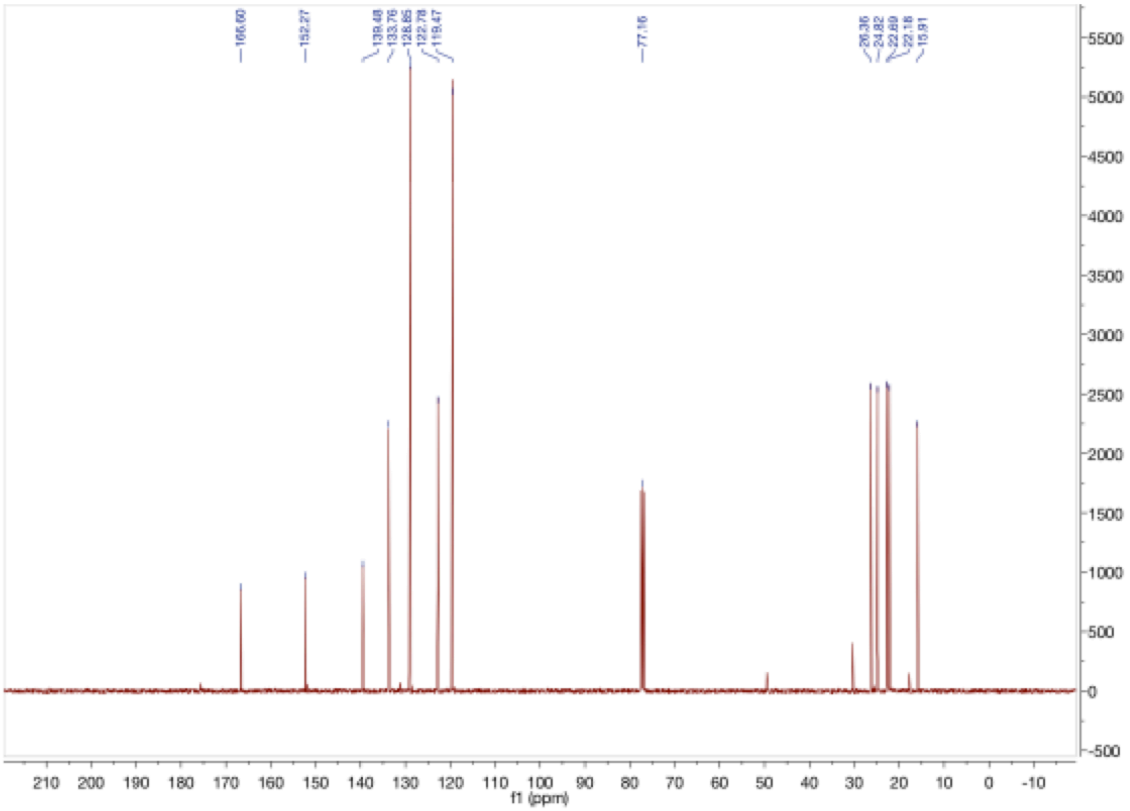

Figure S31. ^{13}C NMR of **2e** in $CDCl_3$.

Synthesis of 1-(cyclooct-1-en-1-yl)ethan-1-one (2f):

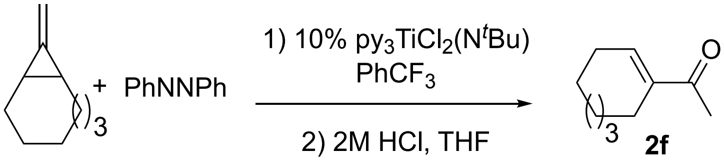

In a nitrogen glovebox, $py_3TiCl_2(N^tBu)$ (47 mg, 0.11 mmol, 0.1 equiv, 0.0192 M), azobenzene (200 mg, 1.10 mmol, 1 equiv, 0.220 M), **1f** (599 mg, 4.40 mmol, 4 equiv, 0.416 M), and $PhCF_3$ (5 mL) were added to a 20 mL scintillation vial containing a stir bar. The flask was sealed with a Teflon cap, removed from the glove box and heated to 145 °C in an oil bath. After 110 h, the reaction mixture was cooled to room temperature, then THF (5 mL) and aq. HCl (5 mL, 2M) were added to the reaction mixture. After stirring for 16 h at room temperature, the reaction was diluted with water (50 mL), and extracted with Et_2O (3 x 30 mL). The combined organic phases were washed with water (1 x 50 mL) and brine (1 x 30 mL), and then dried over $MgSO_4$. The solution was filtered and then concentrated in vacuo. Final purification by column chromatography (silica, 5% Et_2O/hexanes), afforded **2f** (130 mg, 39% in 91% purity; impurities include 9% of the corresponding hydrolyzed hydroamination byproduct, 35% corrected yield) as a yellow oil. The hydrolyzed hydroamination byproduct coeluted with **2f**. Spectral data matched that of the reference.[12]

^1H NMR (400 MHz, $CDCl_3$, 25 °C, δ, ppm): 6.86 (t, J = 8.3 Hz, 1H, C=C-*H*), 2.47-2.38 (m, 2H, C=C-CH_2), 2.38-2.24 (m, 2H, C=C-CH_2), 2.29 (s, 3H, C(O)CH_3), 1.66-1.34 (m, 8H).

^{13}C NMR (101 MHz, $CDCl_3$, 25 °C, δ, ppm): 199.2, 143.6, 143.2, 29.3, 29.2, 27.7, 26.2, 25.3, 23.5.

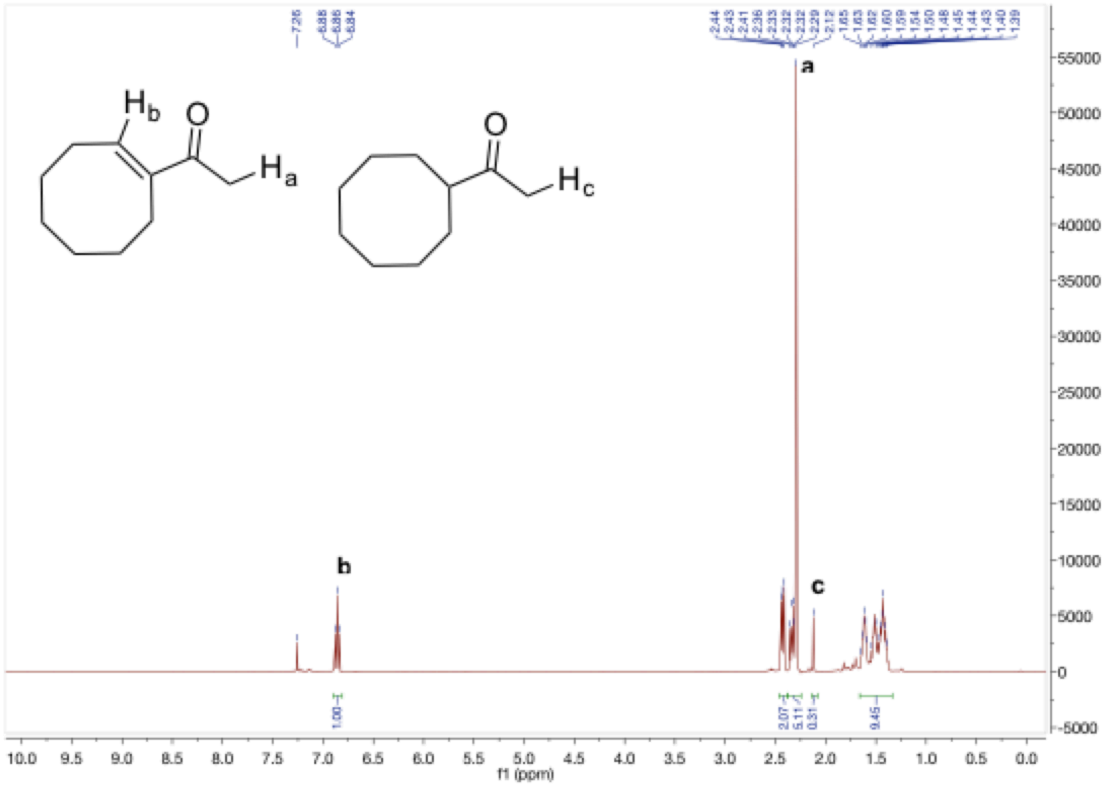

Figure S32. ^{1}H NMR of **2f** in $CDCl_3$.

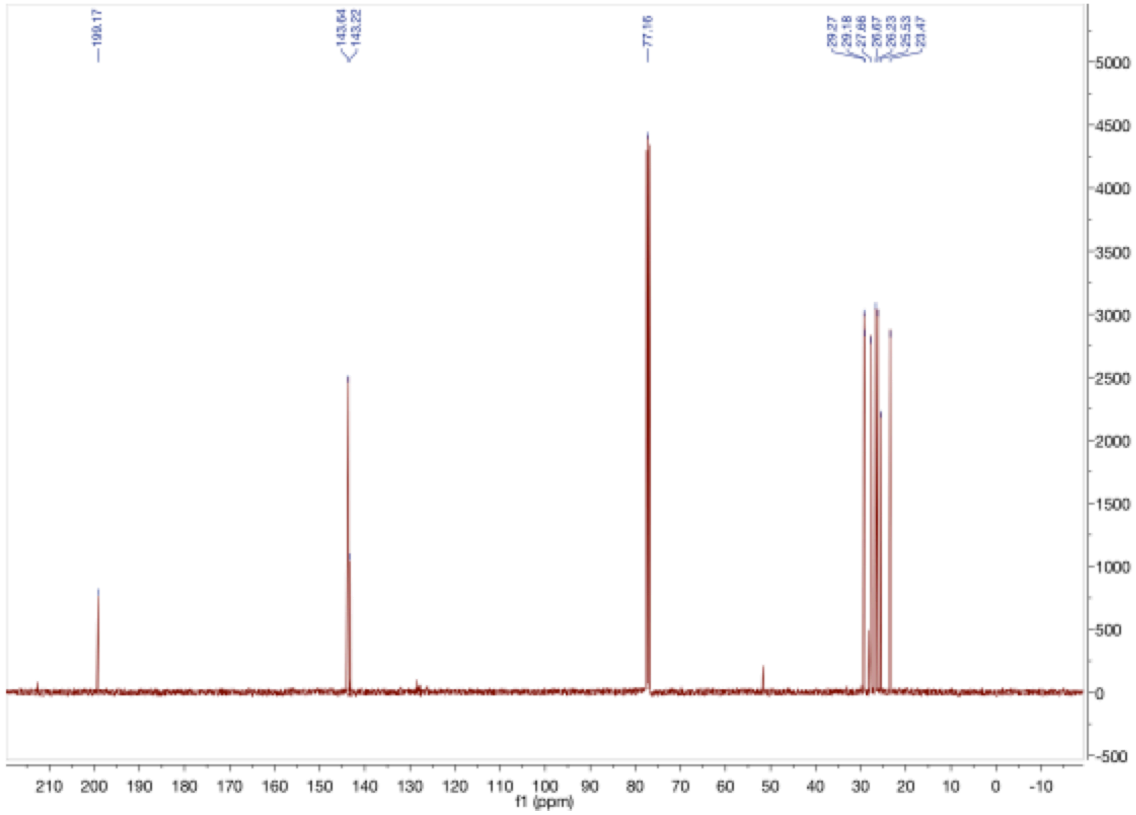

Figure S33. ^{13}C NMR of **2f** in $CDCl_3$.

NMR Tube Reaction Data of Unsuccessful Ti-Catalyzed Reactions

In a nitrogen glovebox, the requisite strained olefin substrate (0.43 mmol, 2.2 equiv, 0.86 M), and 0.5 mL of a stock solution containing azobenzene (0.19 mmol, 1.0 equiv, 0.38 M), $[py_2TiCl_2(N^tBu)]_2$ (0.0095 mmol, 0.05 equiv, 0.019 M), and 1,3,5-$(OMe)_3C_6H_3$ as an internal standard (0.019 mmol, 0.038 M, TMB) in C_6D_5Br was added to an NMR tube. The tube was sealed, wrapped with electric tape, and a time = 0 h ^{1}H NMR spectrum was taken. The reaction was then heated in an oil bath for 62 h at 115 °C. Afterward, the reaction was cooled to room temperature and a time = 62 h ^{1}H NMR spectrum was taken. ^{1}H NMR spectra were taken using acquisition parameters d1 = 30, aq = 5, and ns = 8 on a Bruker Avance 400 MHz spectrometer. Yields were determined by comparison with 1,3,5-$(OMe)_3C_6H_3$, and azobenzene conversions were determined by comparing the initial and final azobenzene concentrations. Spectra for initial and final timepoints for each substrate (**1b**, **1g**, and **1h**) are displayed below.

Table S1. NMR Yields and Azobenzene Conversions of Failed Reactions

Substrate	% Yield of imine	% Azobenzene Conversion[a]
1b[b]	-	98.5
1g	-	0.0
1h	-	0.0

[a]Azobenzene conversion was determined from initial and final time points of the NMR tube reactions using the integration values from the ortho C-H proton of azobenzene around 8 ppm. [b]Following this run, a GC-MS of the final reaction mixture was taken. An m/z of 219 (2 less m/z units of the coupling of MCP **1b** and a phenylnitrene fragment) and 93 (aniline) were observed. The resulting ^{1}H NMR spectrum is attached, but does not display the clean formation of any isolable products.

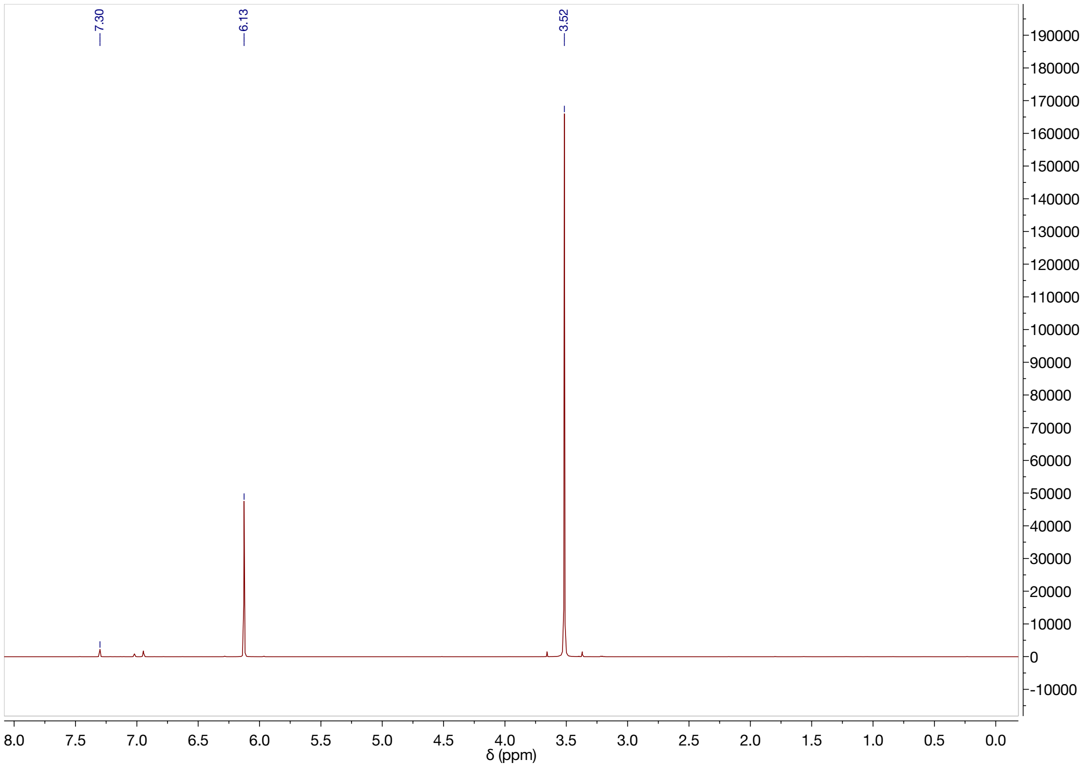

Figure S34. ^{1}H NMR spectrum of 1,3,5-$(OMe)_3C_6H_3$ in C_6D_5Br.

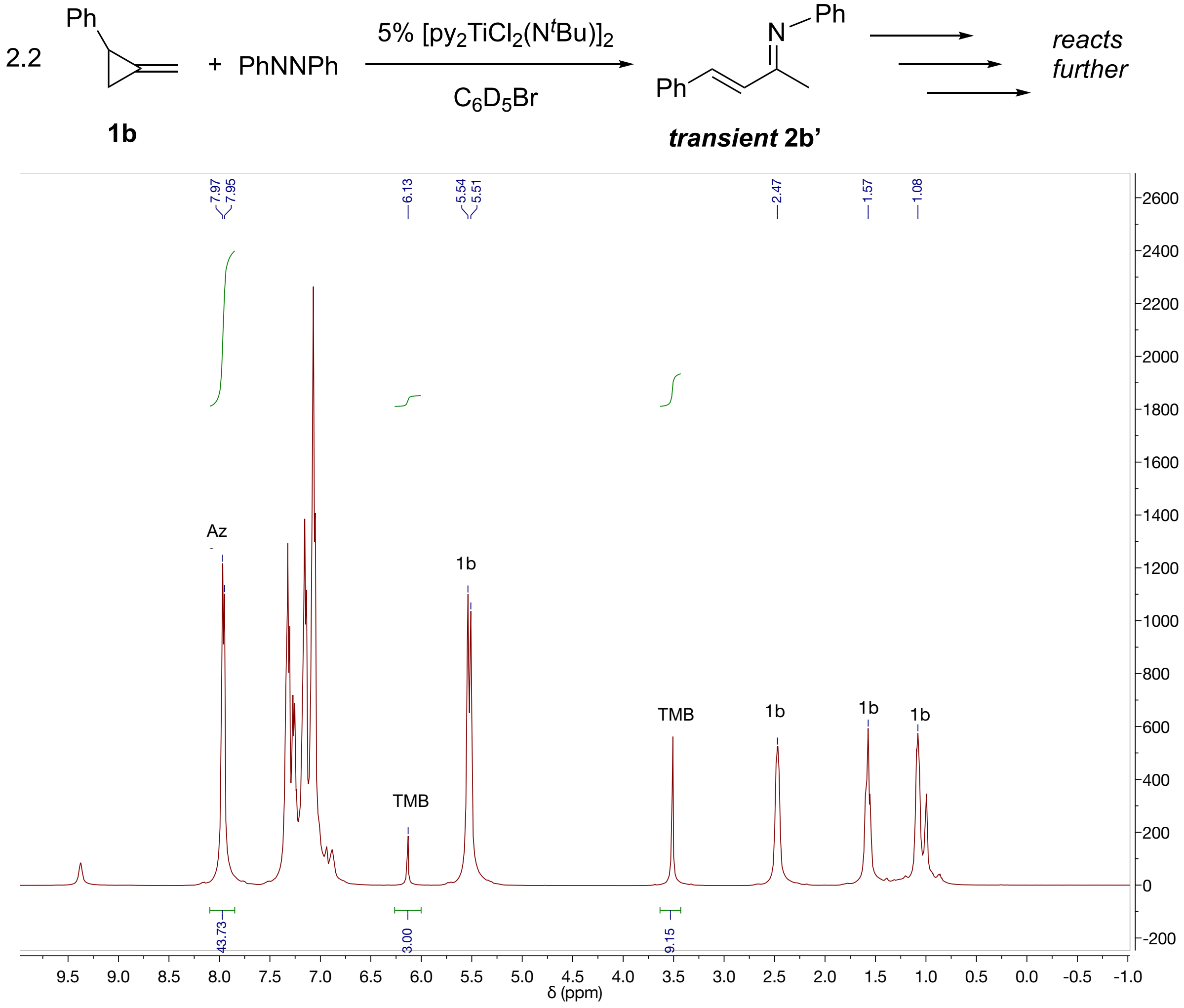

Figure S35. ^{1}H NMR spectrum of the reaction of azobenzene and **1b** at t = 0 h in C_6D_5Br.

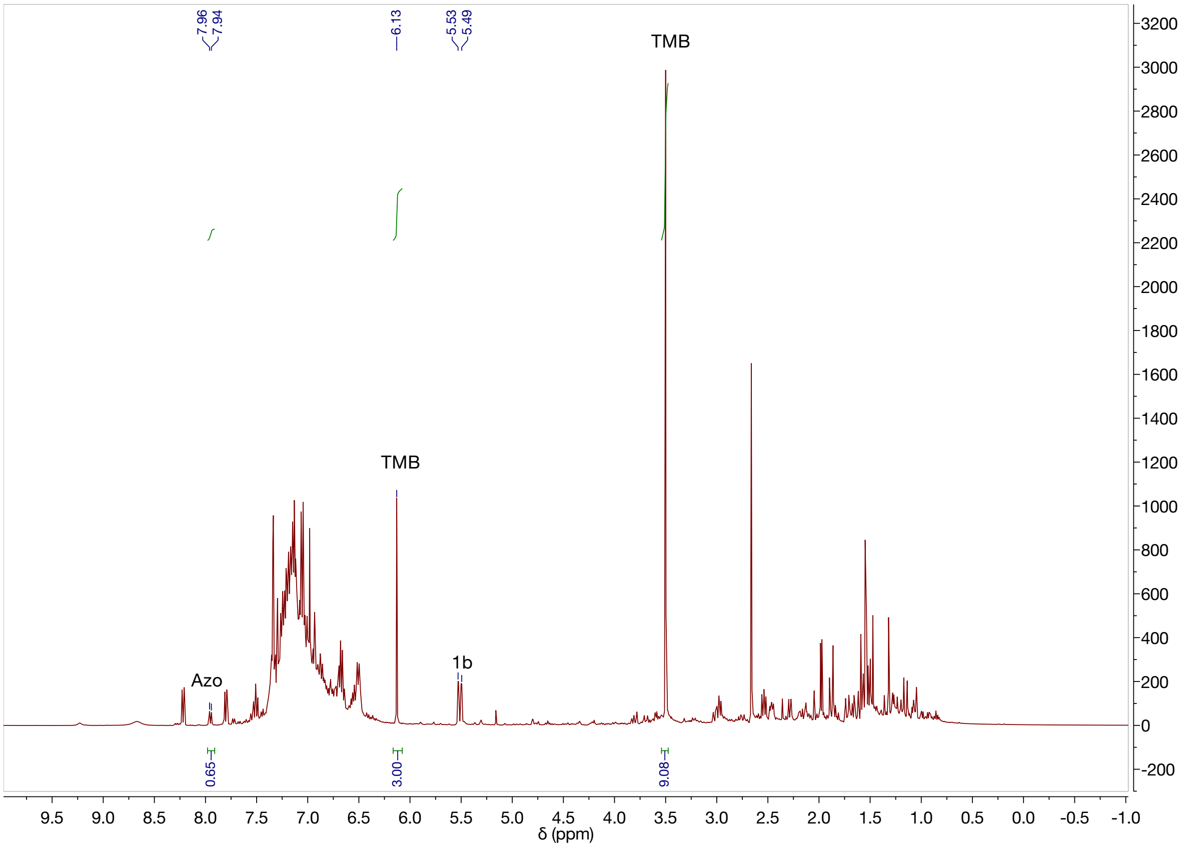

Figure S36. ^{1}H NMR spectrum of the reaction of azobenzene and **1b** at t = 62 h in C_6D_5Br showing the conversion of azobenzene, however with a lack of tractable reaction products.

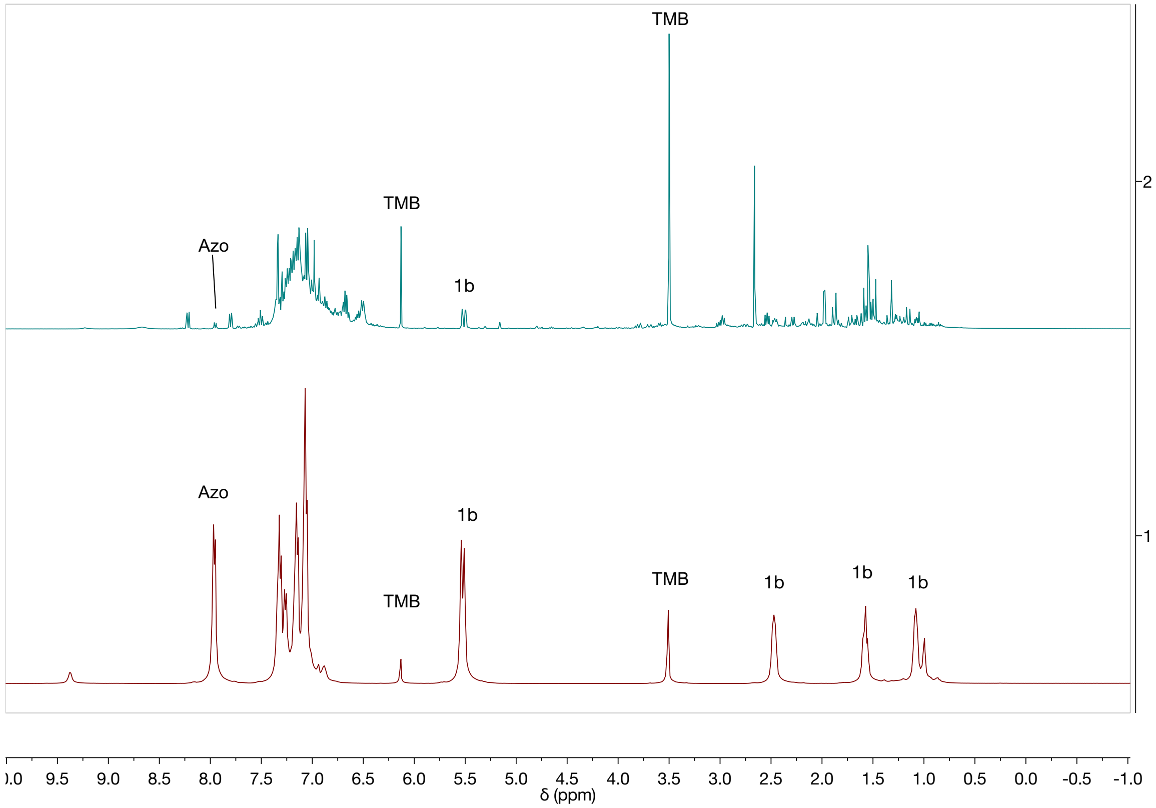

Figure S37. Stacked ^{1}H NMR spectrum of the reaction of azobenzene and **1b** in C_6D_5Br at t = 0 h (bottom) and t = 62 h (top) showing the conversion of azobenzene, however with a lack of tractable reaction products.

Ti-Catalyzed Hydroamination Reactions of 1a and 2a

In a nitrogen glovebox, the requisite MCP (0.363 mmol, 1.1 equiv, 0.726 M), $[py_2TiCl_2(NPh)]_2$ (0.00825 mmol, 0.025 equiv, 0.0165 M) and 0.5 mL of a stock solution containing aniline (0.0.33 mmol, 1.0 equiv, 0.66 M), and 1,3,5-$(OMe)_3C_6H_3$ as an internal standard (0.033 mmol, 0.066 M, TMB) in $PhCF_3$ was added to an NMR tube. The tube was then sealed, wrapped in electric tape, and a time = 0 h No-D ^1H NMR spectrum was taken. The reaction was then heated in an oil bath for 20 h at 115 °C. Afterward, the reaction was cooled to room temperature and a time = 20 h No-D ^1H NMR spectrum was taken. No-D ^1H NMR spectra were taken using acquisition parameters d1 = 30, aq = 5, and ns = 4 on a Bruker Avance III 500 MHz spectrometer. Yields were determined by comparison with 1,3,5-$(OMe)_3C_6H_3$ with respect to aniline loading. Spectra for initial and final timepoints for each substrate (**1a** and **1b**) are displayed below.

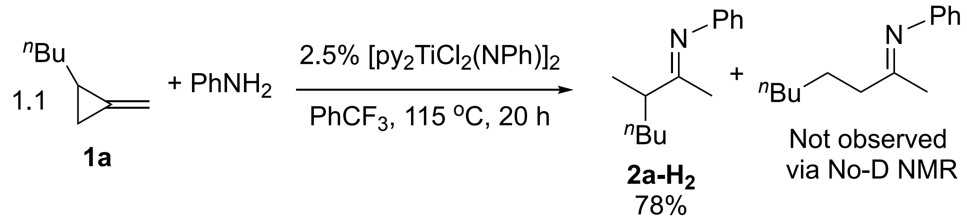

3-methyl-*N*-phenylheptan-2-imine (partial line list):
^1H NMR (500 MHz, $PhCF_3$, 27 °C, δ, ppm): 1.59 (s, 3H, -C(NPh)CH_3), 1.13 (d, 3H, J = 6.9 Hz, -CH(CH_3)C(NPh)CH_3).

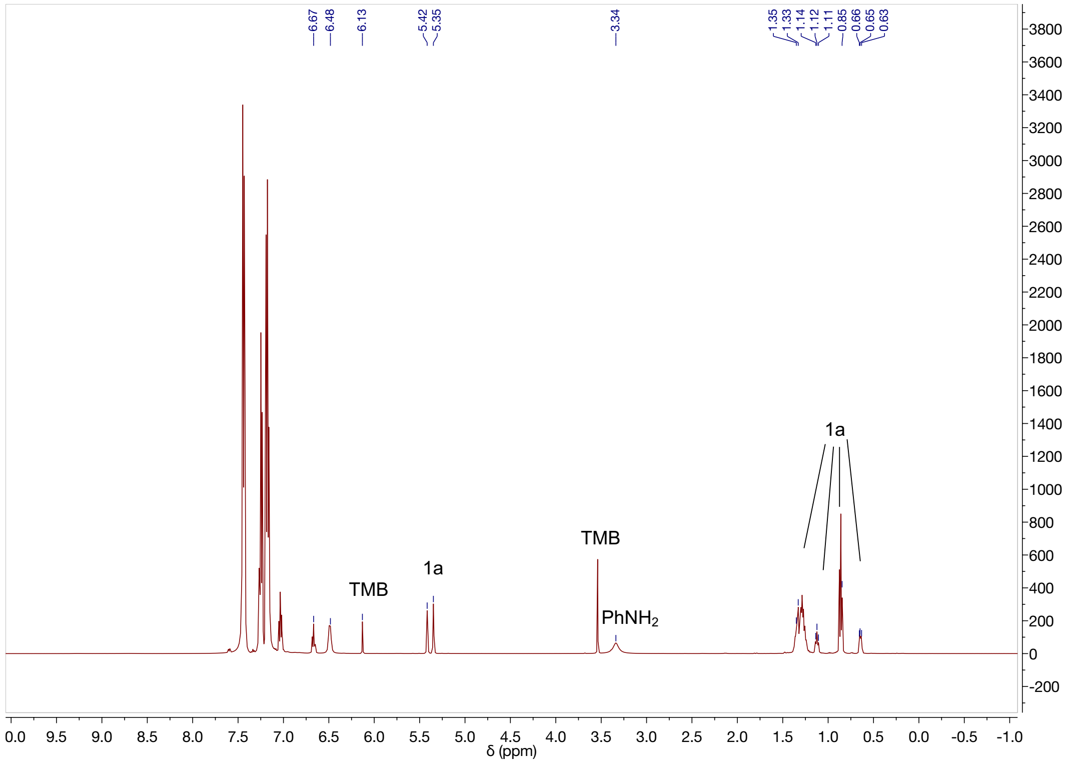

Figure S38. No-D NMR spectrum of the reaction of aniline and **1a** at t = 0 in $PhCF_3$.

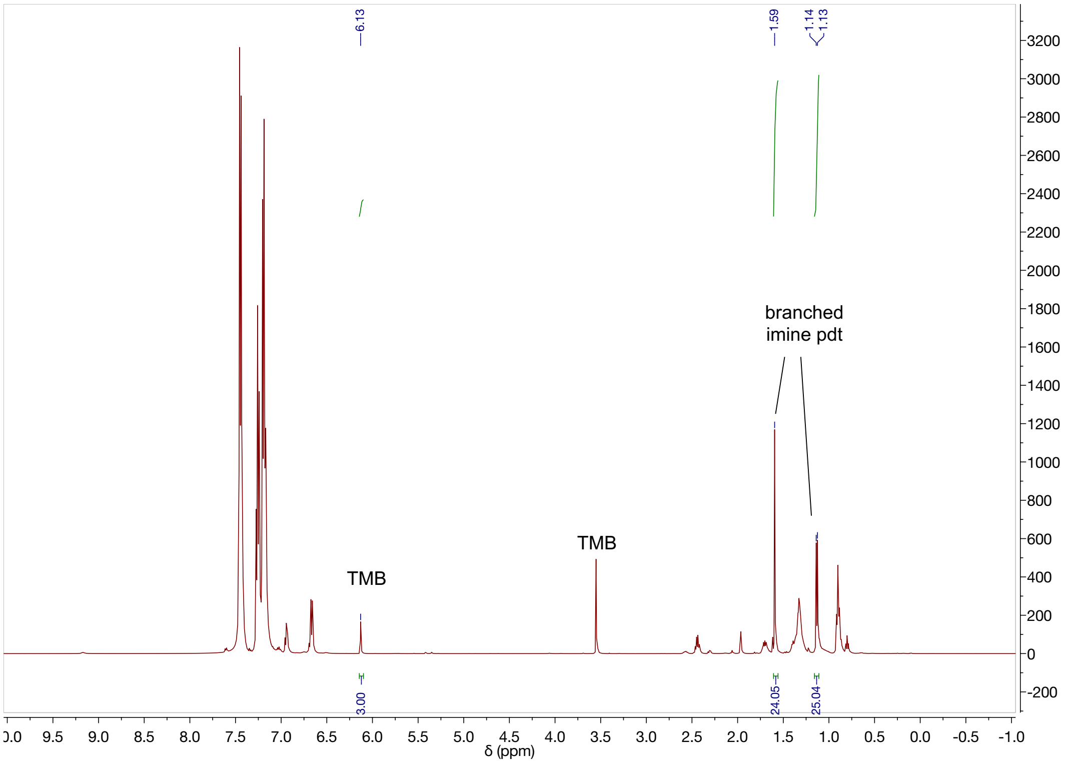

Figure S39. No-D NMR spectrum of the reaction of aniline and **1a** catalyzed by $[py_2TiCl_2(NPh)]_2$ at t = 20 in $PhCF_3$ showing the formation of 3-methyl-*N*-phenylheptan-2-imine (**2a-H₂**). *N*-phenyloctan-2-imine was not observed via No-D NMR spectroscopy.

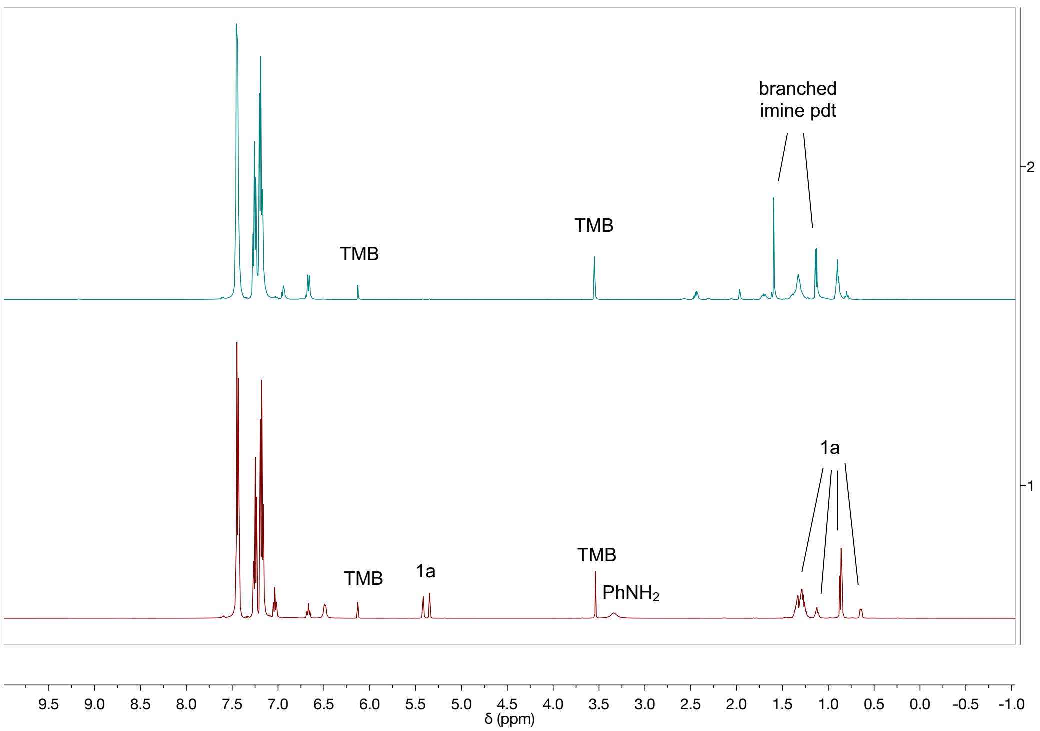

Figure S40. Stacked No-D NMR spectrum of the reaction of aniline and **1a** at t = 0 (bottom) and t = 20 h (top) catalyzed by $[py_2TiCl_2(NPh)]_2$ in $PhCF_3$ showing the formation of 3-methyl-*N*-phenylheptan-2-imine (**2a-H$_2$**). *N*-phenyloctan-2-imine was not observed via No-D NMR spectroscopy.

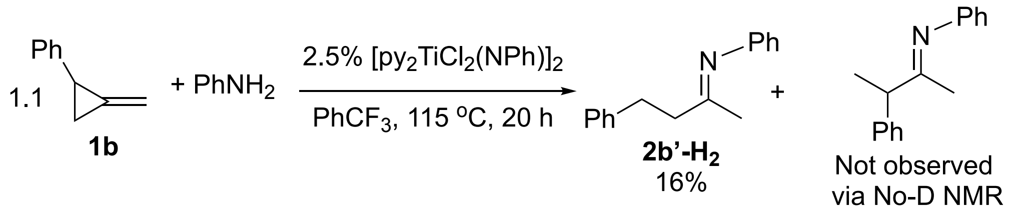

N,3-diphenylbutan-2-imine (partial line list):
^{1}H NMR (500 MHz, $PhCF_3$, 27 °C, δ, ppm): 2.96 (t, 2H, *J* =7.7 Hz, PhC*H*$_2$-), 2.54 (d, 2H, *J* = 7.7 Hz, $PhCH_2$C*H*$_2$-), 1.55 (s, 3H, -C(NPh)C*H*$_3$).

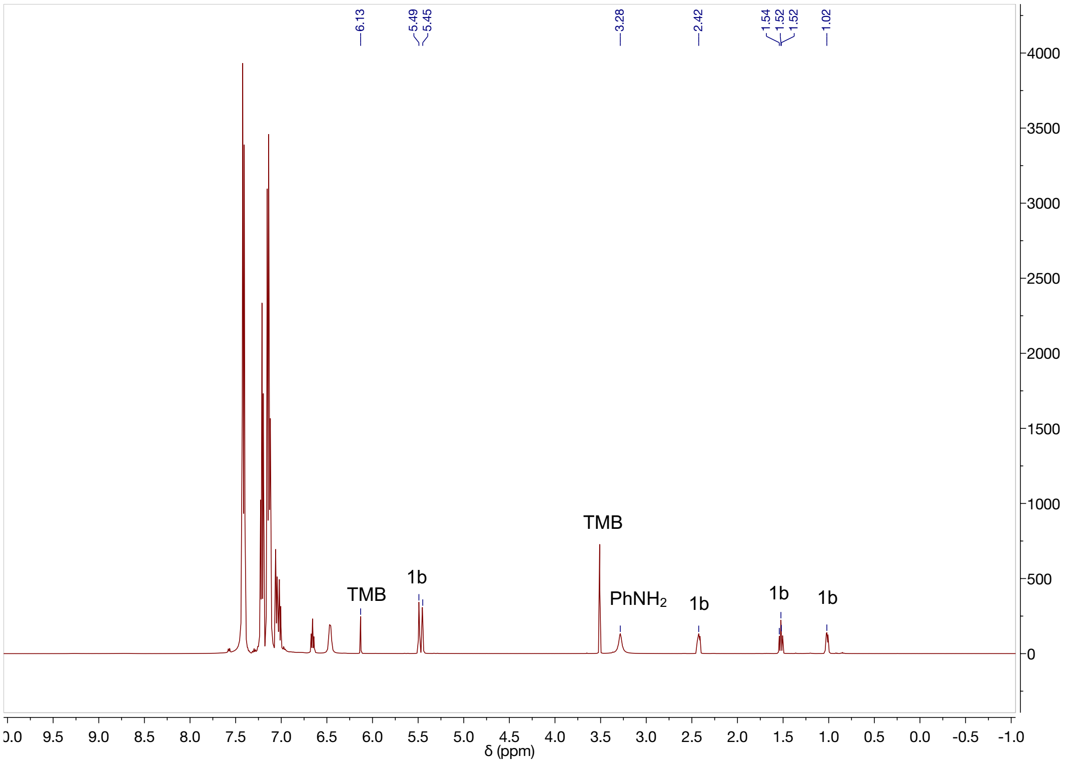

Figure S41. No-D NMR spectrum of the reaction of aniline and **1b** at t = 0 in $PhCF_3$.

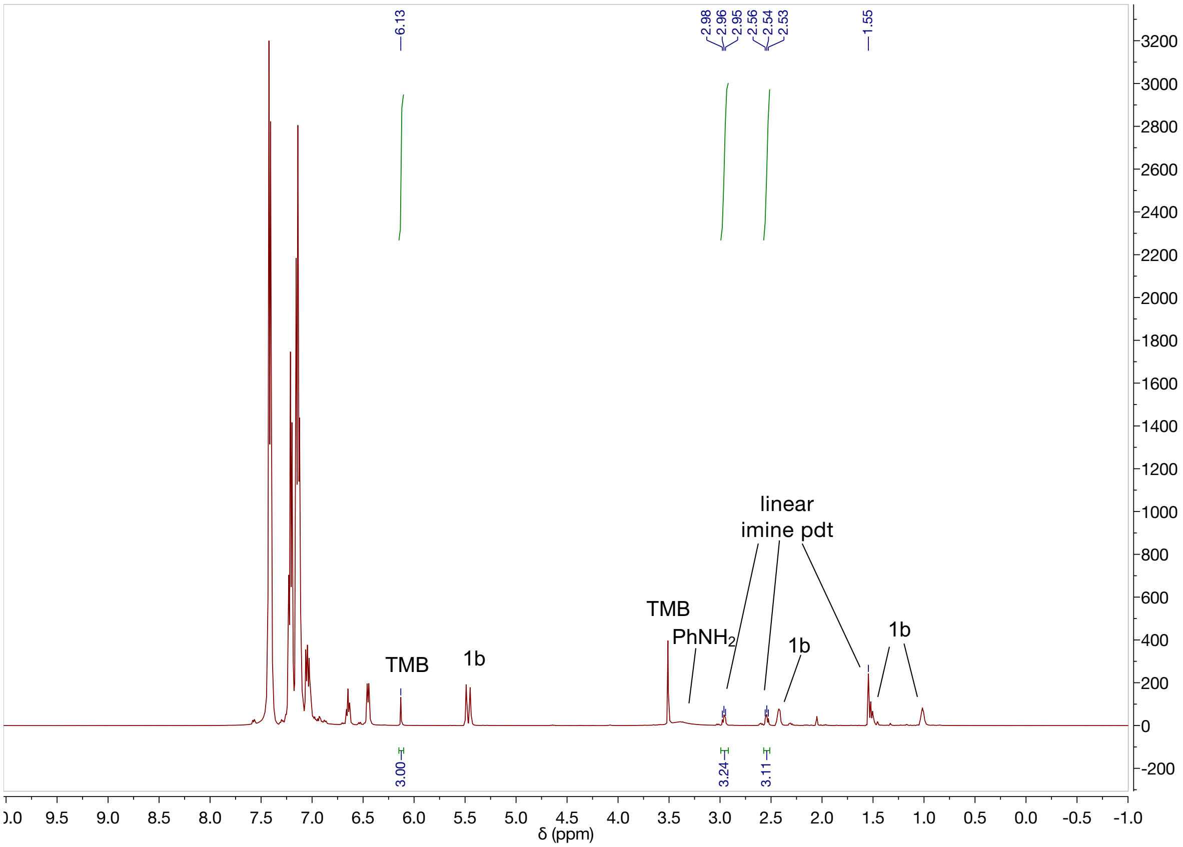

Figure S42. No-D NMR spectrum of the reaction of aniline and **1b** catalyzed by $[py_2TiCl_2(NPh)]_2$ at t = 20 in $PhCF_3$ showing the formation of *N*,3-diphenylbutan-2-imine (**2b'-H$_2$**). *N*,4-diphenylbutan-2-imine was not observed via No-D NMR spectroscopy.

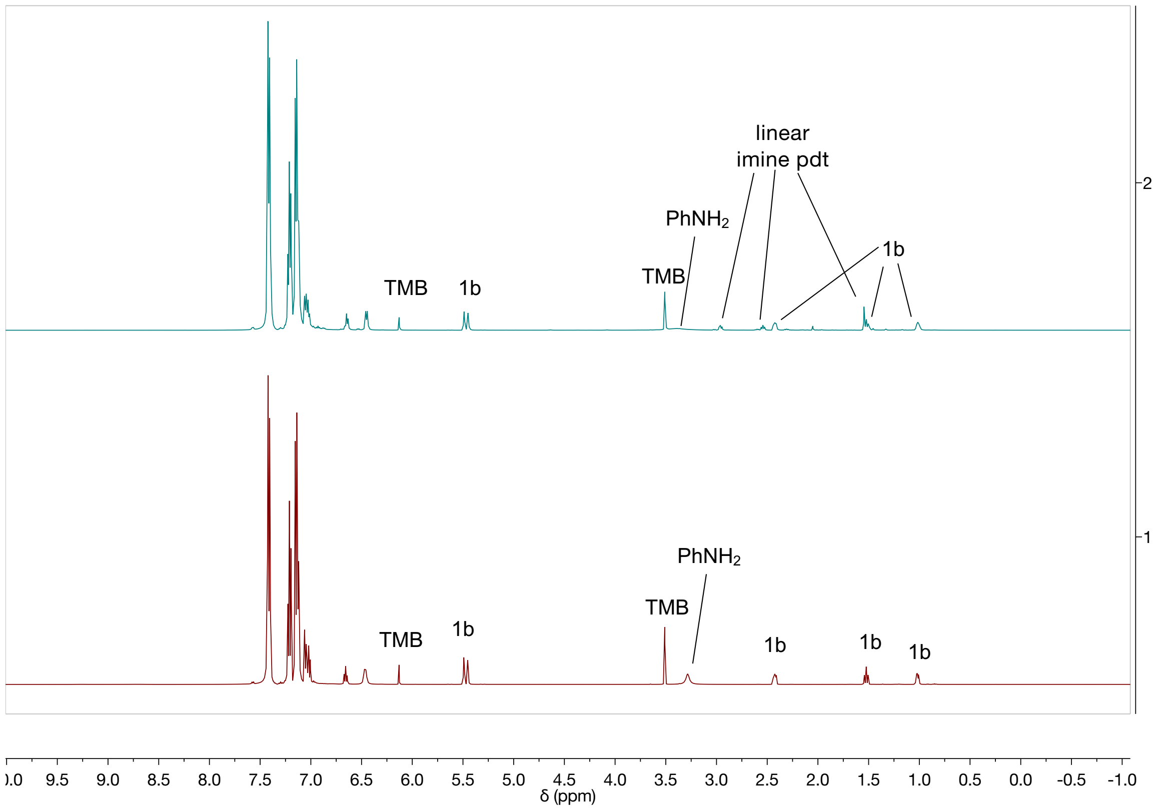

Figure S43. Stacked No-D NMR spectrum of the reaction of aniline and **1b** at t = 0 (top) and t = 20 h (bottom) catalyzed by $[py_2TiCl_2(NPh)]_2$ in $PhCF_3$ showing the formation of *N*,3-diphenylbutan-2-imine (**2b'-H$_2$**). *N*,4-diphenylbutan-2-imine was not observed via No-D NMR spectroscopy.

Functionalization of Unsaturated Imines

Synthesis of 3-methyleneheptan-2-one (3a):

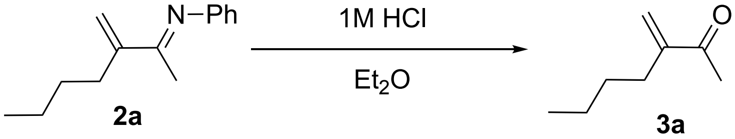

Triphenylmethane (8.0 mg, 0.03 mmol) was added to a solution of compound **2a** (24 mg, 0.12 mmol) in Et_2O (0.36 mL) as an internal standard. A small portion of this solution was concentrated in vacuo to obtain a ^{1}H NMR spectrum of the initial solution. HCl (0.36 mL, 1 M in H_2O, 0.36 mmol) was added to the remaining solution. After 30 min, the reaction was diluted with water, and the resulting mixture was extracted with CH_2Cl_2. The combined organic phases were washed with brine, dried over Na_2SO_4, and concentrated in vacuo. The crude oil was dissolved in $CDCl_3$. The crude yield was determined by ^{1}H NMR integration relative to triphenylmethane using the peak at 5.49 ppm from the standard and 5.93 ppm from the product. Product **3a** was generated in 81% yield.[13,14]

^{1}H NMR (400 MHz, $CDCl_3$, 25 °C, δ, ppm): 6.00 (s, 1H, C=C-*H*), 5.76 (s, 1H, C=C-*H*), 2.34 (s, 3H, C(O)CH_3), 2.27 (t, *J* = 6.7 Hz, 2H, C=C-CH_2), 1.42 – 1.29 (m, 4H), 0.92 (t, *J* = 7.1 Hz, 3H, CH_2-CH_3).

IR (cm^{-1}, NaCl): 2957, 1228, 1678, 1454.

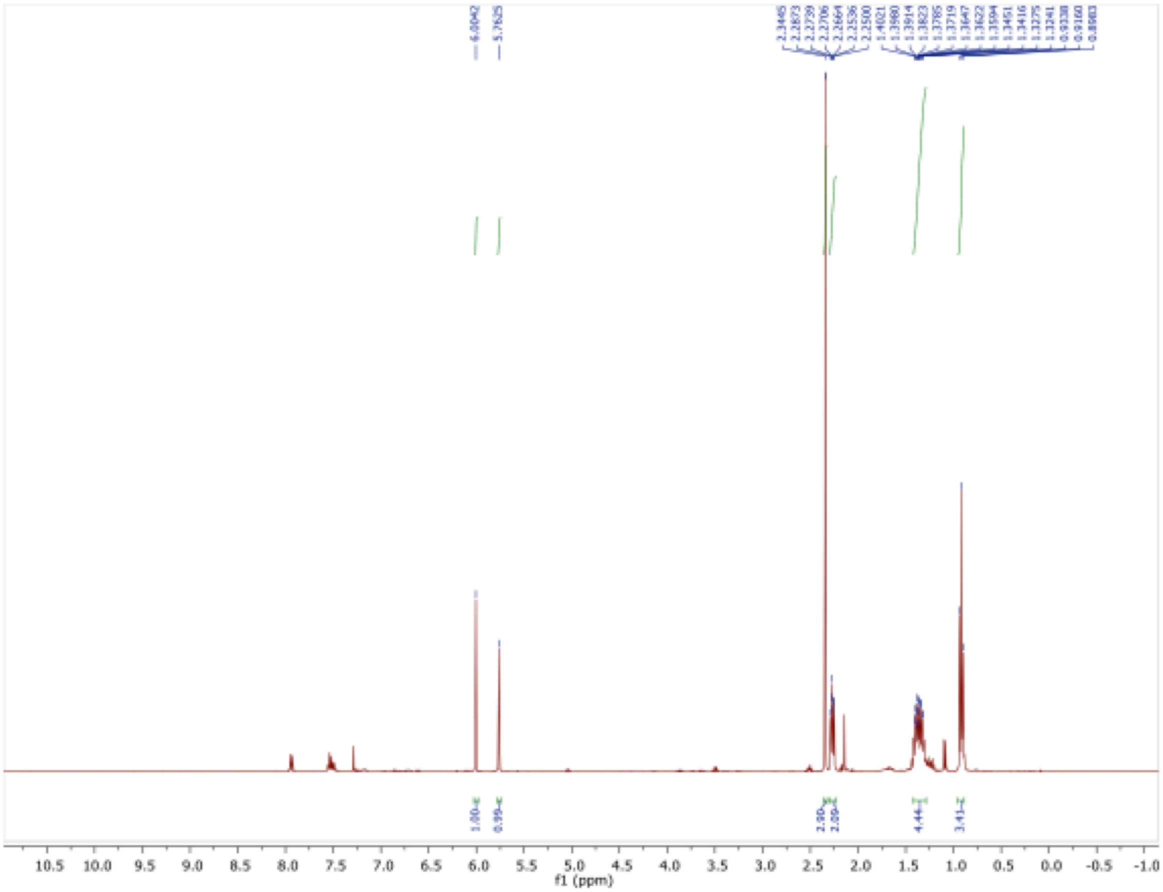

Figure S44. ^1H NMR of **3a** in $CDCl_3$.

Synthesis of (*E*)-3-butyl-*N*-phenyloctan-2-imine (4a):

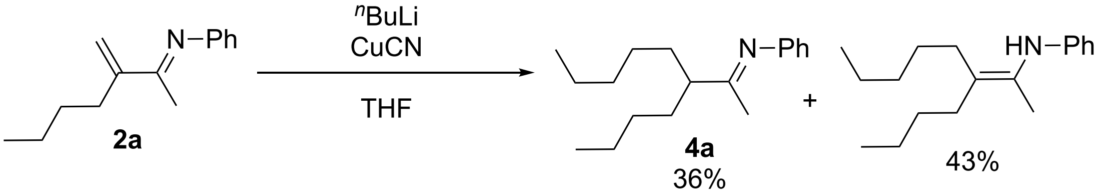

CuCN (79 mg, 0.88 mmol, 1.7 equiv) was added to a three neck round bottom flask containing a stir bar. The flask was fitted with two septa and a Schlenk line adaptor. The flask was connected to a Schlenk line and placed under an atmosphere of argon using three evacuate and back-fill cycles. The flask was then partially submerged into a brine bath. THF (10 mL) and ^{n}BuLi (0.75 mL, 2.5 M in hexanes, 1.8 mmol, 3.5 equiv) were then added to the flask. A separate vial was charged with imine **2a** (105 mg, 0.52 mmol, 1 equiv), 1,3,5-$(OMe)_3C_6H_3$ (20 mg, 0.12 mmol), and THF (2.5 mL). The solution of **2a** was added dropwise to the round bottom flask over 5 min. After 2 h, the reaction was quenched by the addition of ^{i}PrOH (0.2 mL). The solution was diluted with EtOAc in hexanes (10% EtOAc, 20 mL), which resulted in the precipitation of copper salts. The resulting slurry was passed through a short path of silica gel that was pre-equilibrated with triethylamine. The pad was rinsed with EtOAc in hexanes (10% EtOAc, 80 mL) and the filtrate was concentrated in vacuo. The residue was dissolved in $CDCl_3$ and analyzed by ^{1}H NMR spectroscopy. The crude yield was determined by integration relative to 1,3,5-$(OMe)_3C_6H_3$ using the peaks at 6.12 and 3.80 ppm from the standard, 2.06 ppm from the remaining starting material and 1.98 and 1.71 from the enamine and imine products, respectively. The crude mixture contained 36% of imine **4a** and 43% of its enamine tautomer. Attempts at purifying this mixture by silica gel chromatography resulted in a poor recovery of the enamine (~10%). The solution of enamine in $CDCl_3$ was unstable at room temperature and gradually converted to imine **4a**. A ^{13}C NMR spectrum was obtained of the crude mixture with imine **4a** as the major product.

Imine **4a**:

^{13}C NMR (101 MHz, $CDCl_3$, 25 °C, δ, ppm): 175.2, 151.7, 128.9, 122.8, 119.4, 50.7, 33.1, 32.9, 32.0, 29.8, 27.2, 22.8, 22.6, 16.7, 14.1 (2C).

MS (ESI): Calculated for $C_{18}H_{30}N+$, $(M + H)^+$ 260.2373, observed 260.2384.

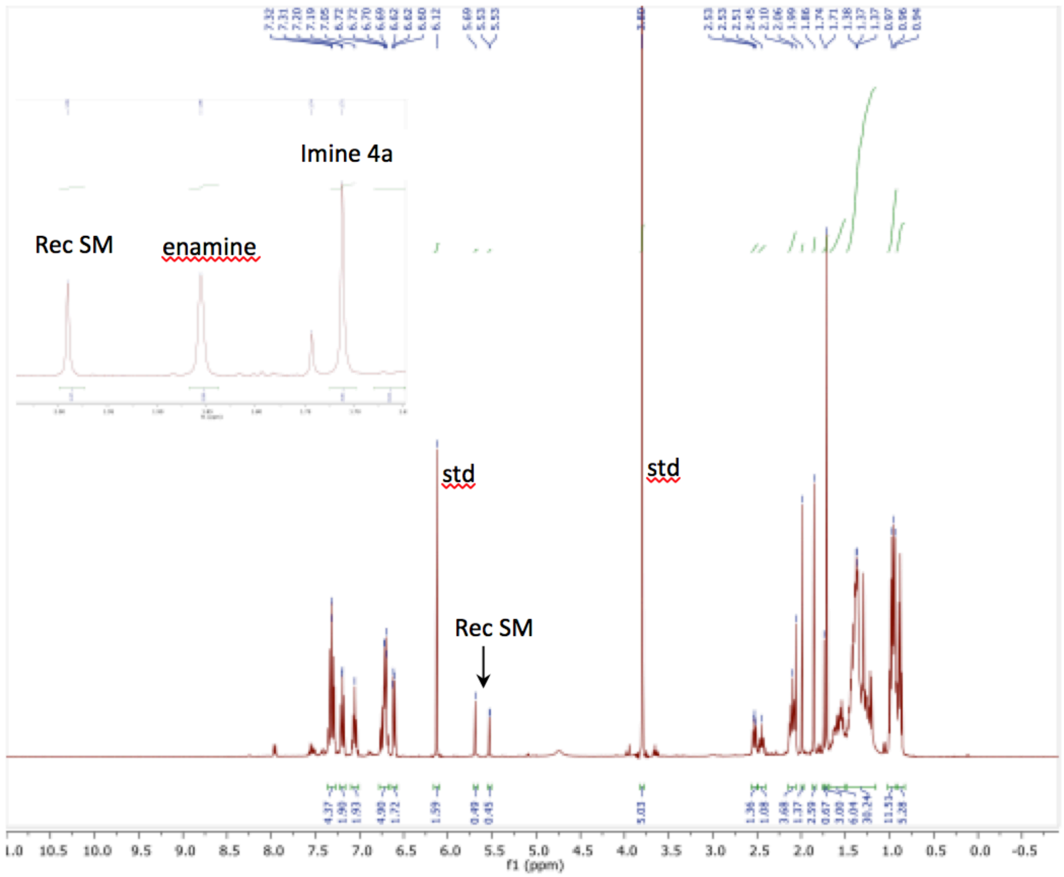

Figure S45. ^{1}H NMR of crude **4a** and its enamine tautomer in $CDCl_3$ with TMB as an internal standard.

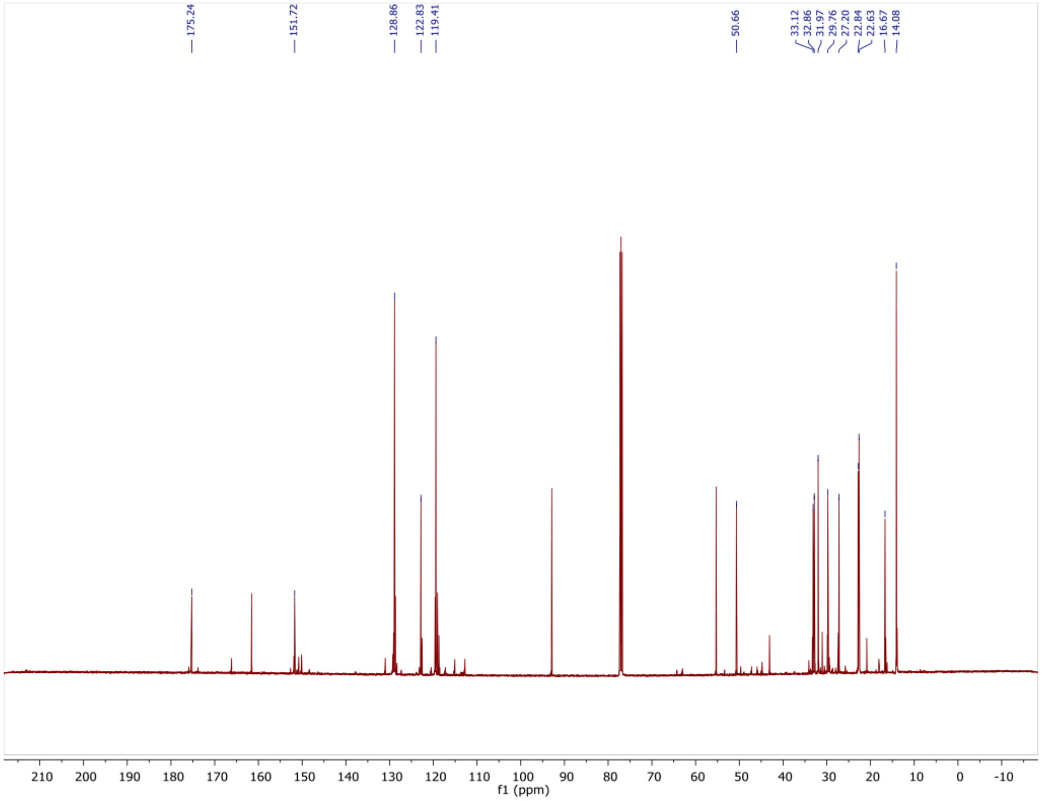

Figure S46. ^{13}C NMR of crude **4a** and its enamine tautomer with **4a** as the major product in $CDCl_3$.

Synthesis of *N*-(4-methyl-5-methylenenon-1-en-4-yl)aniline (5a):

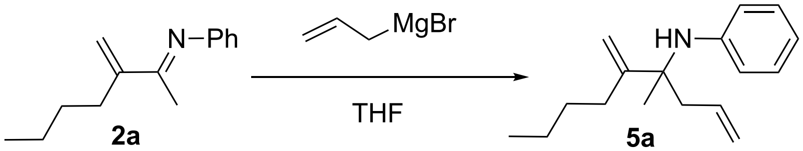

Imine **2a** (32 mg, 0.16 mmol, 1 equiv) was dissolved in THF (1 mL) and cooled in an ice bath. Allyl magnesium bromide (0.21 mL, 1 M in THF, 0.21 mmol, 1.3 equiv) was added dropwise to the solution of **2a**. After 2 h, the reaction was quenched by addition of water. The resulting solution was extracted with CH_2Cl_2. The combined organic phases were washed with brine, dried over Na_2SO_4, and concentrated in vacuo. Final purification by column chromatography (0-30% EtOAc in hexanes) afforded compound **5a** (33 mg, 83%) as an oil.

^{1}H NMR (500 MHz, $CDCl_3$, 25 °C, δ, ppm): 7.13 (t, *J* = 8.6 Hz, 2H, Ar-C-*H*), 6.72 – 6.63 (m, 3H, Ar-C-*H*), 5.86 – 5.73 (m, 1H, C=C-*H*), 5.20 – 5.10 (m, 3H, C=C-*H*), 5.04 (s, 1H), 3.76 (s, 1H), 2.57 (dd, *J* = 13.5, 7.7 Hz, 1H, C=C-CH_2), 2.39 (dd, *J* = 13.5, 7.1 Hz, 1H, C=C-CH_2), 2.15 (t, *J* = 7.8 Hz, 2H, CH_2-CH_2), 1.51 (quintet, *J* = 7.7 Hz, 2H, CH_2-CH_2-CH_2), 1.44 (s, 3H, C(NH)CH_3), 1.36 (sextet, *J* = 7.5 Hz, 2H, CH_2-CH_2-CH_3), 0.94 (t, *J* = 7.4 Hz, 3H, CH_2-CH_3).

^{13}C NMR (101 MHz, $CDCl_3$, 25 °C, δ, ppm): 154.5, 146.7, 133.9, 128.8, 118.7, 117.0, 114.9, 109.4, 58.7, 44.7, 30.5, 30.2, 24.3, 22.8, 14.2.

MS (ESI): Calculated for $C_{17}H_{25}N^+$ (M + H)$^+$ 244.2060, observed 244.2071.

IR (cm^{-1}, NaCl): 3411, 2956, 2929, 1602, 1505.

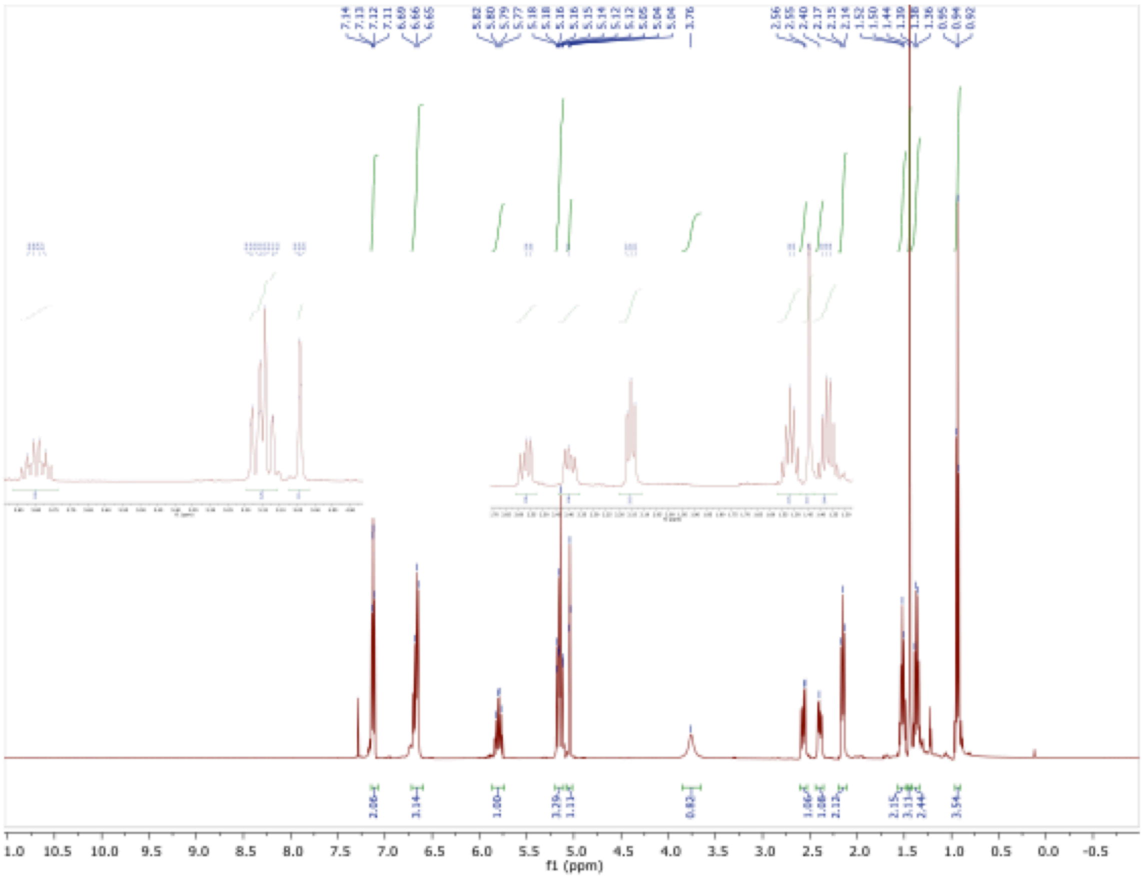

Figure S47. ^{1}H NMR of **5a** in $CDCl_3$.

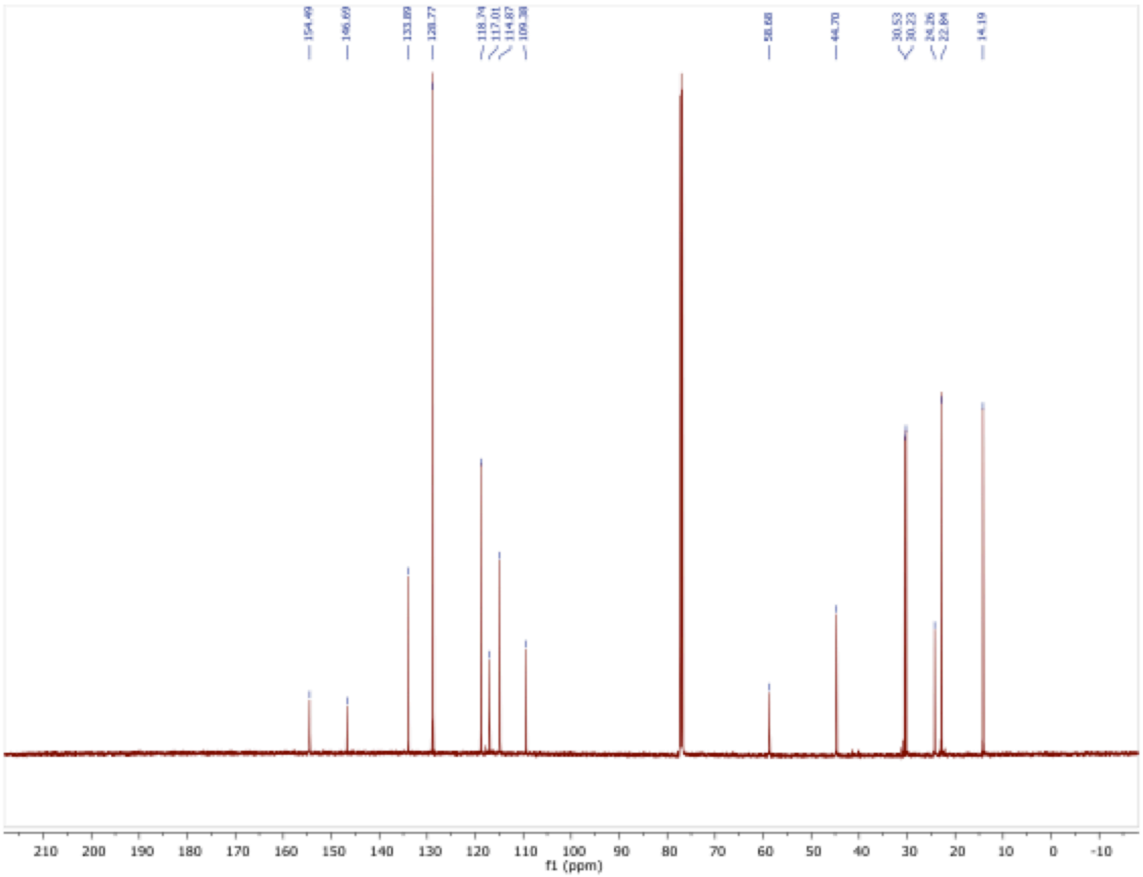

Figure S48. ^{13}C NMR of **5a** in $CDCl_3$.

Synthesis of *N*-(3-methyleneheptan-2-yl)aniline (6a):

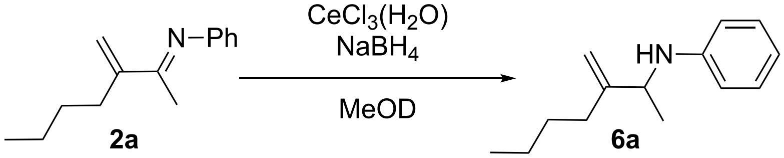

Compound **2a** (26 mg, 0.13 mmol, 1 equiv) was dissolved in MeOD (0.55 mL), and 1,3,5-$(OMe)_3C_6H_3$ (14 mg, 0.084 mmol) was added as an internal standard. After acquiring an NMR spectrum of the initial solution, $CeCl_3 \cdot H_2O$ (53 mg, 0.14 mmol, 1.1 equiv) and $NaBH_4$ (14 mg, 0.37 mmol, 2.8 equiv) were sequentially added. Once the evolution of H_2 had stopped, the reaction mixture was quenched by the addition of water. The resulting solution was extracted with CH_2Cl_2. The combined organic phases were washed with brine, dried over Na_2SO_4, and concentrated in vacuo. The crude oil was dissolved in $CDCl_3$ and the yield was determined by ^1H NMR integration relative to 1,3,5-$(OMe)_3C_6H_3$ using the peak at 6.13 ppm from the standard and 5.07 ppm from the product. Compound **6a** was generated in 93% yield.

^1H NMR (500 MHz, $CDCl_3$, 25 °C, δ, ppm): 7.18 (t, *J* = 7.8 Hz, 2H, Ar-C-*H*), 6.70 (d, *J* = 7.4 Hz, 1H, Ar-C-*H*), 6.59 (d, *J* = 8.1, 2H, Ar-C-*H*), 5.07 (s, 1H, C=C-*H*), 4.87 (s, 1H, C=C-*H*), 3.92 (q, *J* = 6.7 Hz, 1H, CH_3-*CH*), 3.72 (s, 1H, N-*H*), 2.08 (t, *J* = 7.8 Hz, 2H, C=C-C*H*$_2$), 1.57 – 1.45 (m, 2H, CH_2-*CH*$_2$-CH_2), 1.41 – 1.34 (m, 2H, CH_2-*CH*$_2$-CH_3), 1.36 (d, *J* = 6.6 Hz, 3H, CH-C*H*$_3$), 0.95 (t, *J* = 7.3 Hz, 3H, CH_2-*CH*$_3$).

^{13}C NMR (101 MHz, $CDCl_3$, 25 °C, δ, ppm): 151.6, 147.6, 129.1, 117.0, 113.1, 108.7, 54.0, 31.8, 30.2, 22.7, 21.6, 14.0.

MS (ESI): Calculated for $C_{14}H_{24}N^+$ $(M + H)^+$ 204.1747, observed 204.1756

IR (cm^{-1}, NaCl): 3411, 2958, 2928, 2871, 1602, 1505.

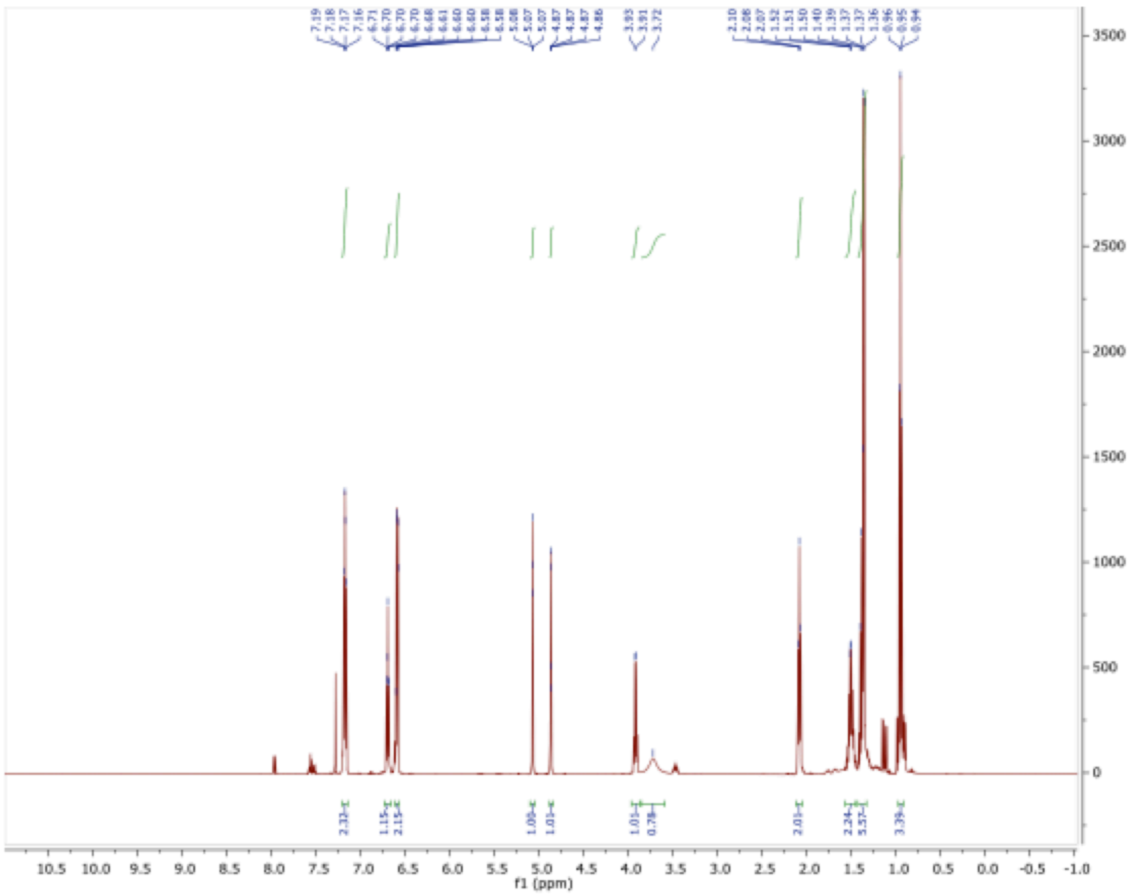

Figure S49. ^{1}H NMR of **6a** in $CDCl_3$.

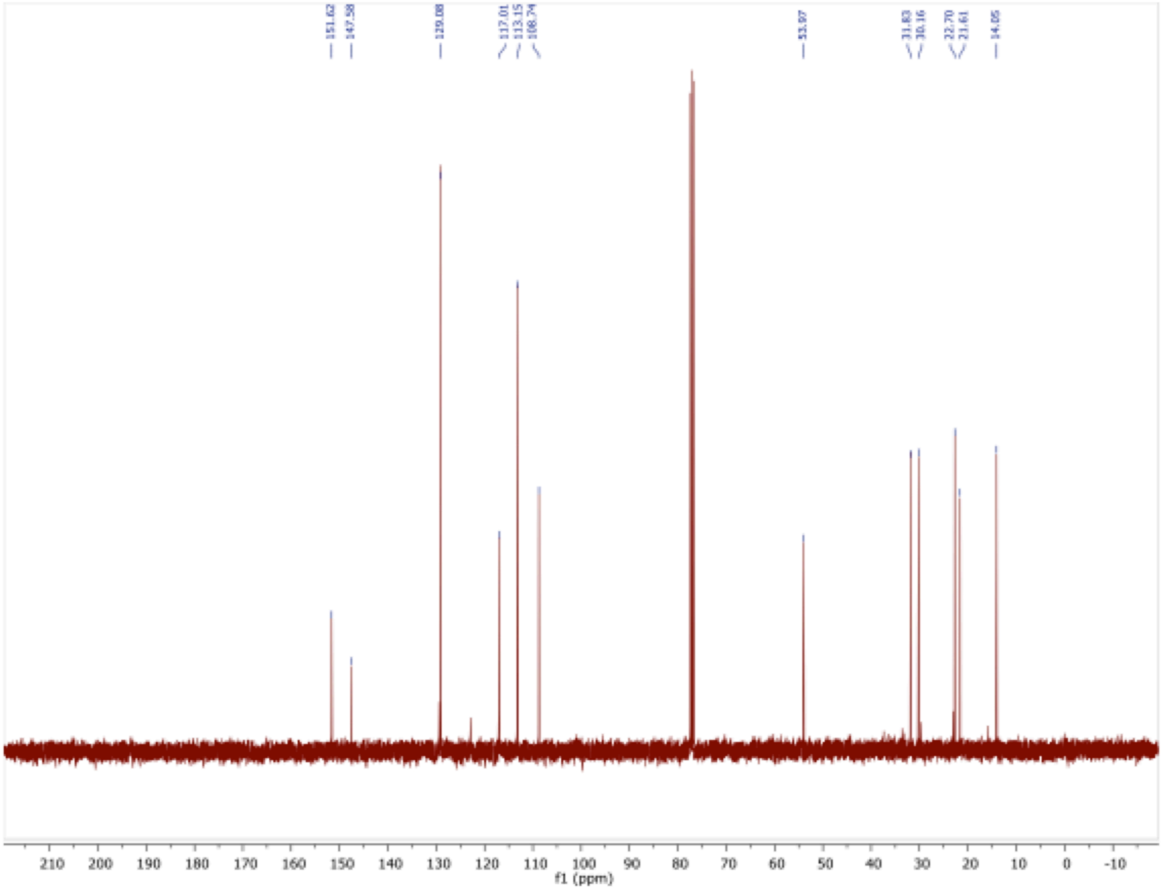

Figure S50. ^{13}C NMR of **6a** $CDCl_3$.

Synthesis of 1-(cyclohex-1-en-1-yl)ethan-1-one (3e):

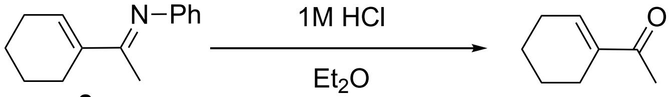

2e **3e**

Compound **2e** (43 mg, 0.21 mmol) was dissolved in Et_2O (0.64 mL) at room temperature and aq. HCl (0.64 mL, 1 M, 0.64 mmol) was added. After 2 h, the reaction was diluted with water. The resulting solution was extracted with CH_2Cl_2. The combined organic phases were washed with brine, dried over Na_2SO_4, and concentrated in vacuo to afford compound **3e** (25 mg, 92%) as an oil. The ^1H NMR spectrum of the pure compound is identical to the commercial product and is provided below.

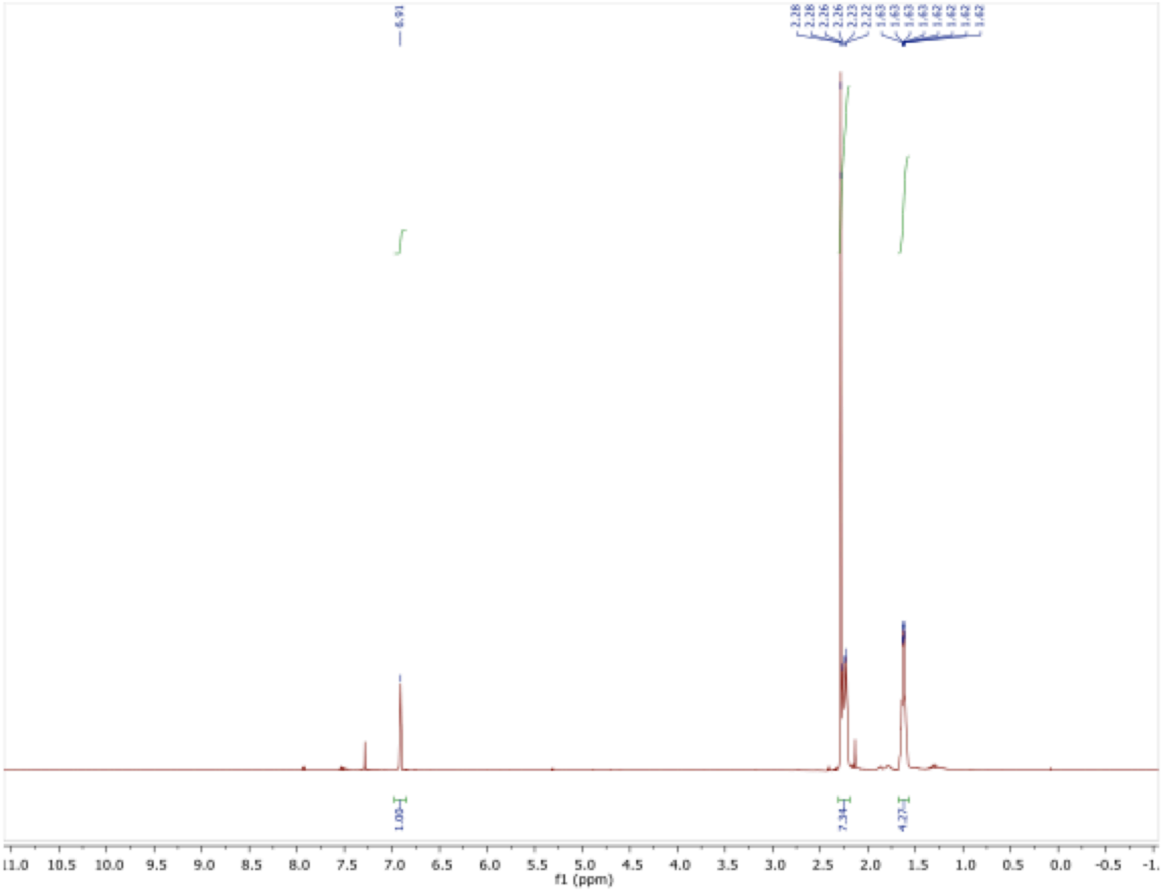

Figure S51. ^1H NMR of **3e** in $CDCl_3$.

Synthesis of 4-(cyclohex-1-en-1-yl)-3,3,4-trimethyl-1-phenylazetidin-2-one (4e):

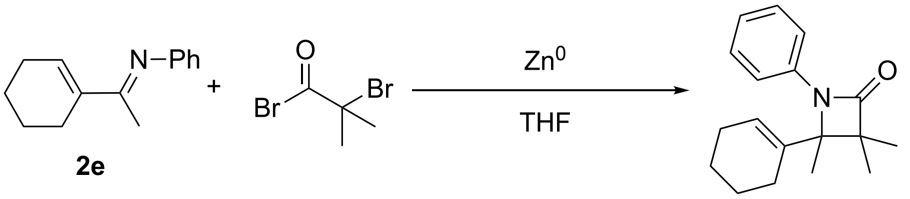

2e

4e

A 20 mL scintillation vial was charged with Zn (784 mg, 12.0 mmol, 50 equiv) and then placed under an argon atmosphere. A solution of imine **2e** (48 mg, 0.24 mmol, 1 equiv) in THF (1 mL) was added at room temperature, followed by *slow* addition of α-bromoisobutyryl bromide (0.17 mL, 1.4 mmol, 5.8 equiv) via syringe. After 1 h, the reaction mixture was quenched with water. The resulting solution was extracted with CH_2Cl_2. The combined organic phases were washed with brine, dried over Na_2SO_4, and concentrated in vacuo. Final purification by column chromatography (0-30% EtOAc in hexanes) afforded compound **4e** (28 mg, 53%) as an oil.

^{1}H NMR (500 MHz, $CDCl_3$, 25 °C, δ, ppm): 7.55 (d, *J* = 7.3 Hz, 2H, Ar-C-*H*), 7.30 (t, *J* = 7.3 2H, Ar-C-*H*), 7.06 (t, *J* = 7.5 Hz, 1H, Ar-C-*H*), 5.59 (s, 1H, C=C-*H*), 2.14 – 2.05 (m, 3H), 1.80 – 1.72 (m, 3H), 1.64 (s, 3H, C-CH_3), 1.56 – 1.52 (m, 2H), 1.34 (s, 3H, C-CH_3), 1.23 (s, 3H, C-CH_3).

^{13}C NMR (125 MHz, $CDCl_3$, 25 °C, δ, ppm): 172.1, 138.1, 128.7, 128.7, 123.8, 123.0, 118.1, 70.0, 57.1, 26.7, 24.8, 22.5, 22.0, 19.0, 18.9, 18.7.

MS (ESI): Calculated for $C_{18}H_{23}NONa^+$ $(M + Na)^+$ 292.1672, observed 292.1671.

IR (cm^{-1}, NaCl): 2929, 1748, 1600, 1497, 1360.

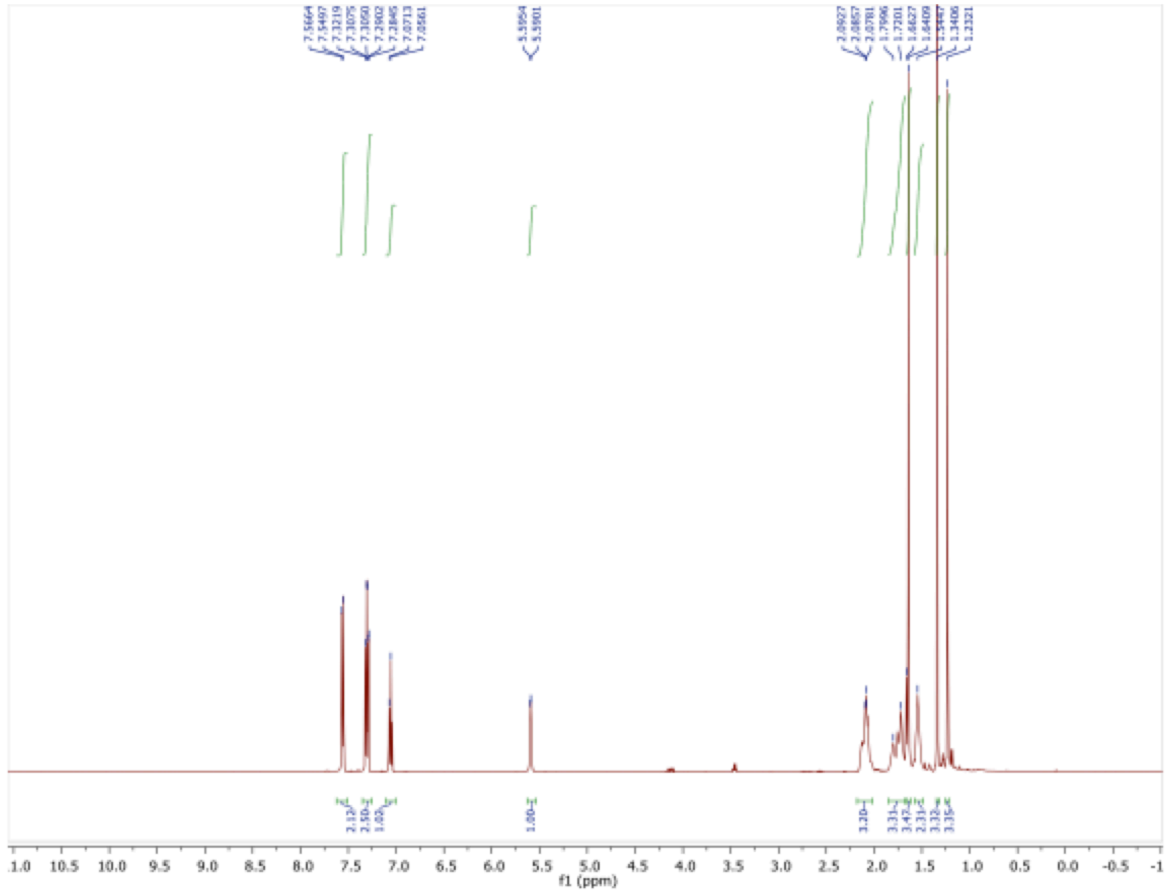

Figure S52. ^{1}H NMR of **4e** in $CDCl_3$.

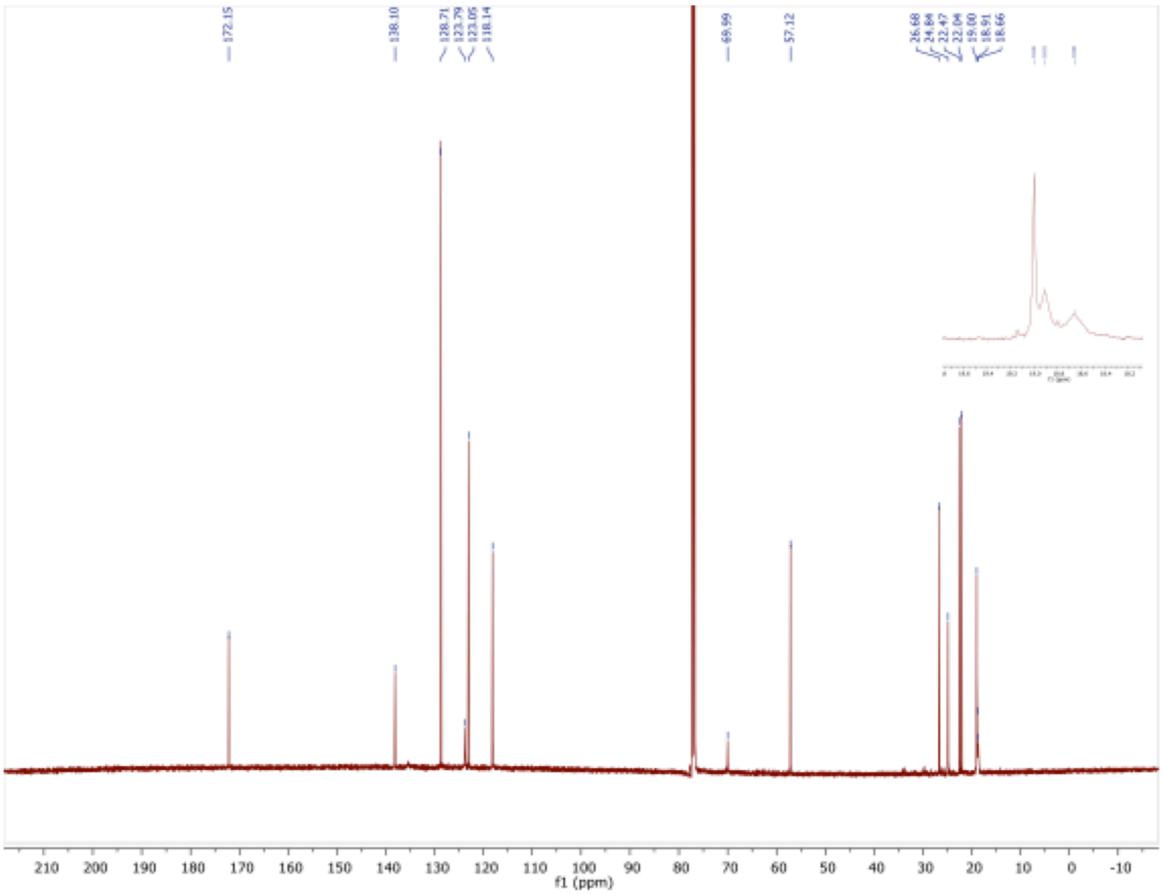

Figure S53. ^{13}C NMR of **4e** in $CDCl_3$.

Synthesis of *N*-(2-(cyclohex-1-en-1-yl)pent-4-en-2-yl)aniline (5e):

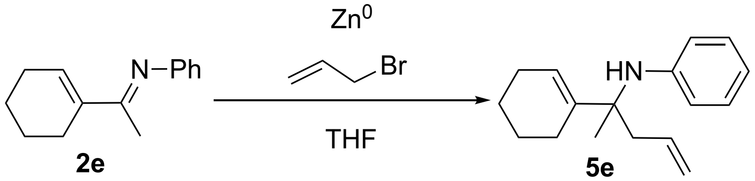

A 20 mL scintillation vial was charged with Zn (118 mg, 1.80 mmol, 9.5 equiv) and then placed under an argon atmosphere. THF (1 mL) and allyl bromide (50 µL, 0.58 mmol, 3.1 equiv) were sequentially added to the vial via syringe at room temperature. After 1 h, imine **2e** (38 mg, 0.19 mmol, 1 equiv) was added. After 16 h, the reaction was filtered through cotton and quenched by addition of water. The resulting solution was extracted with CH_2Cl_2. The combined organic phases were washed with brine, dried over Na_2SO_4, and concentrated in vacuo. Final purification by column chromatography (0-30% EtOAc in hexanes) afforded compound **5e** (32 mg, 68%) as an oil.

^{1}H NMR (400 MHz, $CDCl_3$, 25 °C, δ, ppm): 7.15 – 7.10 (m, 2H, Ar-C-*H*), 6.71 – 6.64 (m, 3H, Ar-C-*H*), 5.85 – 5.78 (m, 1H, C=C-*H*), 5.78 – 5.72 (m, 1H, C=C-*H*), 5.20 – 5.07 (m, 2H, C=C-*H*), 3.71 (s, 1H, N-*H*), 2.49 (dd, *J* = 13.4, 7.8 Hz, 1H, C=C-C*H$_2$*), 2.36 (dd, *J* = 13.5, 6.8 Hz, 1H, C=C-C*H$_2$*), 2.20 – 2.12 (m, 2H, C=C-C*H$_2$*), 2.12 – 2.06 (m, 2H, C=C-C*H$_2$*), 1.62 (m, 4H), 1.40 (s, 3H, C-C*H$_3$*).

^{13}C NMR (101 MHz, $CDCl_3$, 25 °C, δ, ppm): 146.9, 142.1, 134.1, 128.8, 122.1, 118.4, 116.8, 114.7, 58.3, 44.8, 25.6, 24.4, 23.8, 23.3, 22.4.

MS (ESI): Calculated for $C_{17}H_{24}N^+$ $(M + H)^+$ 242.1903, observed 242.1901.

IR (cm^{-1}, NaCl): 3410, 3051, 2928, 2836, 1602, 1503, 1320.

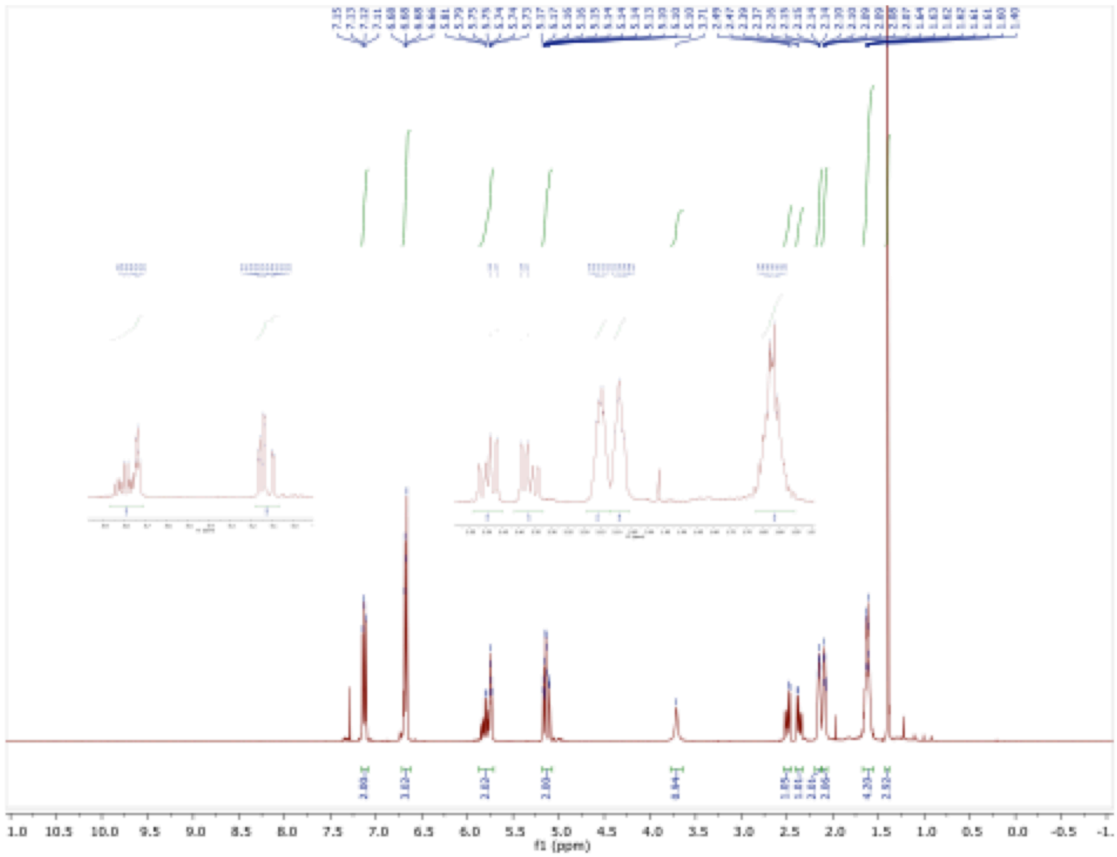

Figure S54. ^{1}H NMR of **5e** in $CDCl_3$.

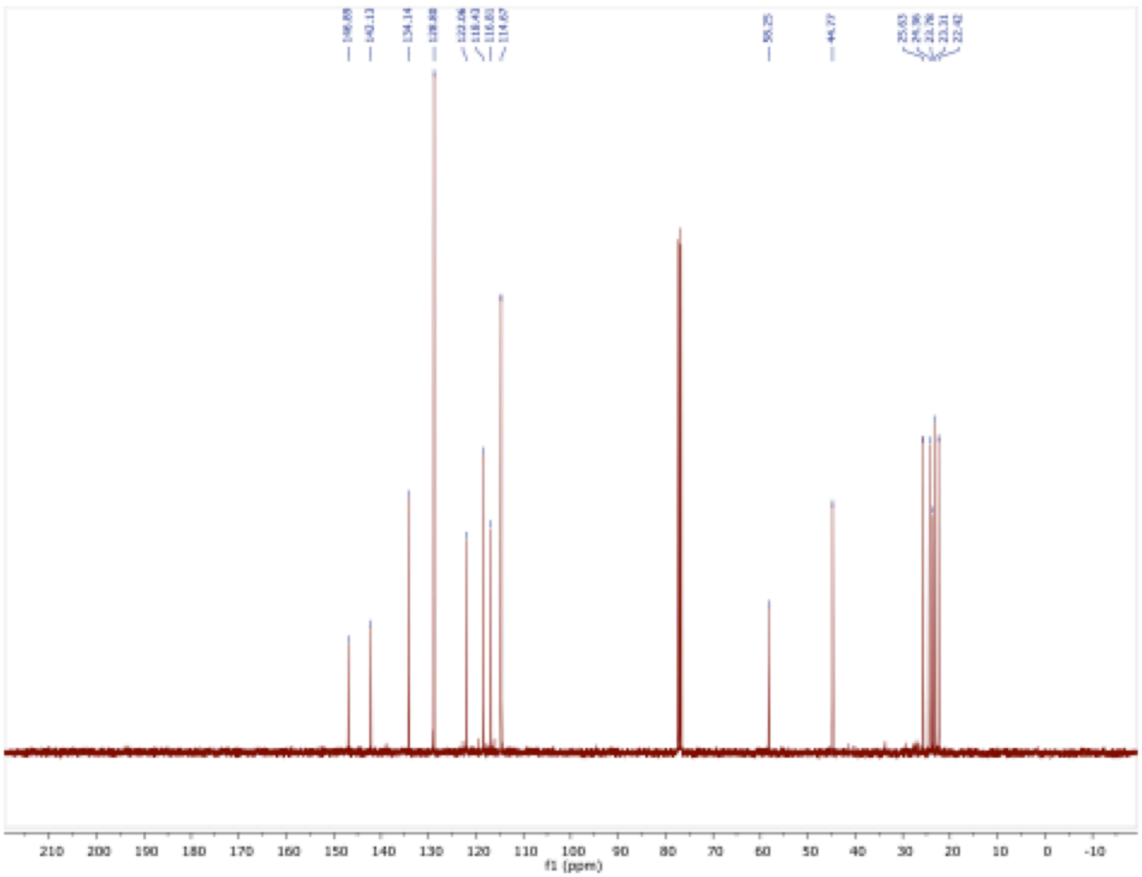

Figure S55. ^{13}C NMR of **5e** in $CDCl_3$.

Synthesis of *N*-(1-(cyclohex-1-en-1-yl)ethyl)aniline (6e):

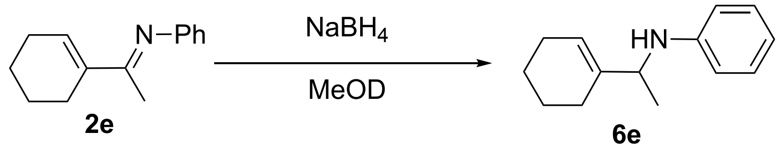

Compound **2e** (34 mg, 0.17 mmol, 1 equiv) was dissolved in MeOD (0.55 mL), and 1,3,5-$(OMe)_3C_6H_3$ (14 mg, 0.084 mmol) was added as an internal standard. After acquiring a ^{1}H NMR spectrum of the initial solution, $NaBH_4$ (14 mg, 0.37 mmol, 2.2 equiv) was added. Once the evolution of H_2 had stopped, a second ^{1}H NMR spectrum was obtained. The crude yield was determined by integration relative to 1,3,5-$(OMe)_3C_6H_3$ using the peak at 6.09 ppm from the standard and 5.65 ppm from the product. The product was generated in 91% yield. To obtain a pure compound, the crude mixture was basified using saturated $NaHCO_3$. The resulting solution was extracted with CH_2Cl_2. The combined organic phases were washed with brine, dried over Na_2SO_4, and concentrated in vacuo. Final purification by column chromatography (0-30% EtOAc in hexanes) afforded compound **6e** as an oil. The ^{1}H NMR spectrum of the pure compound is identical to that reported in the literature.[15] An image of the ^{1}H NMR spectrum is provided below.

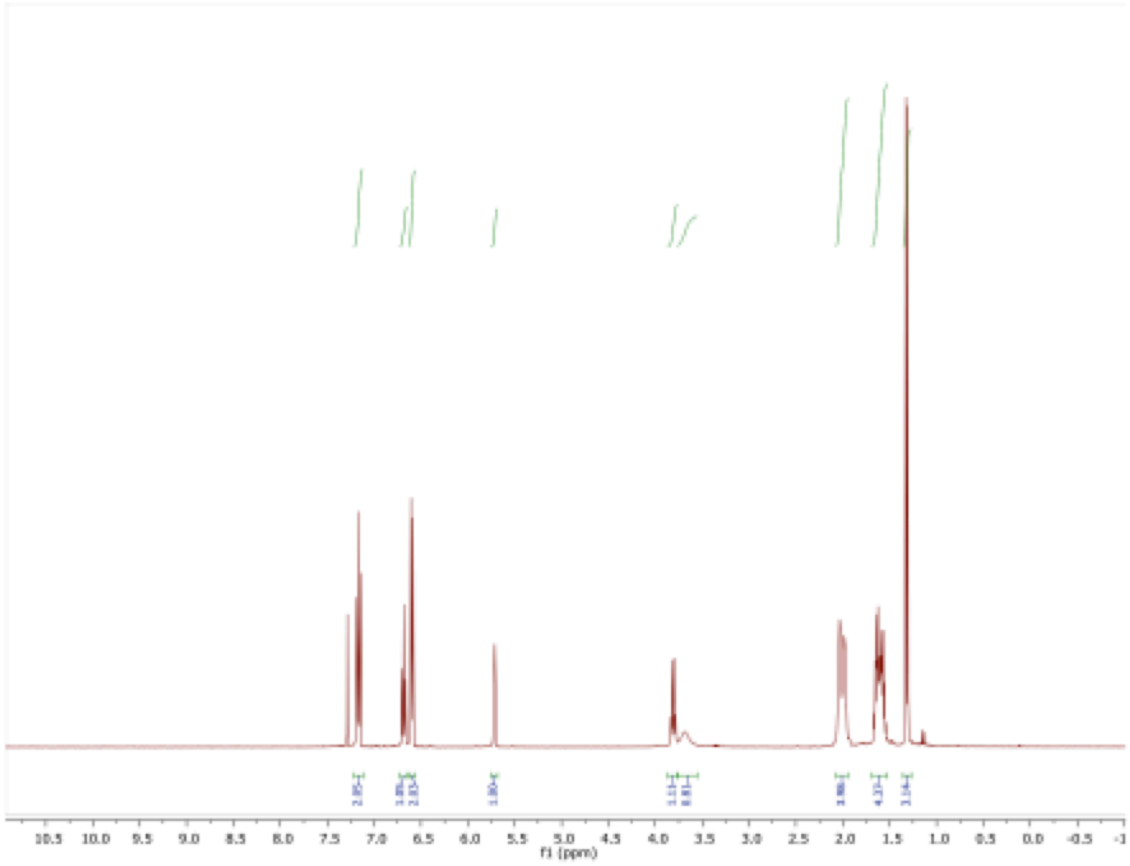

Figure S56. ^{1}H NMR of **6e** in $CDCl_3$.

Computational Details

Computational Protocol

Geometry optimizations were performed using Gaussian 16 program.[16] All geometry optimizations and frequency calculations were performed using the M06[17] functional, 6-311G(d,p) basis set,[18] the super fine grid setting, and the SMD solvation model.[19] The solvent was set to be the experimentally used solvent; therefore, $PhCF_3$ (ε = 9.18) for $py_3TiCl_2(N^tBu)$ and toluene for $Ti(NMe_2)_4$. All geometries were characterized by frequency analysis calculations to be local minima (without any imaginary frequency) or transition states (with only one imaginary frequency). Due to the breakdown of the harmonic oscillator model for low frequency vibrational modes, all non-imaginary vibrational frequencies below 50 cm^{-1} were replaced with values of 50 cm^{-1}. Zero-point vibrational energies and thermal contributions to electronic energy were calculated at 388.15 K and 1 atm for $py_3TiCl_2(N^tBu)$ and 383.15 K and 1 atm for $Ti(NMe_2)_4$. The NBO calculations utilized the NBO 5.G program,[20] and the d orbital occupation numbers were generated following the protocol described in ref 21.[21]

Ligand Effects

Our previous computational studies on [2+2+1] pyrrole synthesis[3] have shown that ligand coordination can play a significant role in the resulting barriers of the mechanism. Therefore, we considered $py_2TiCl_2(NPh)$, $pyTiCl_2(NPh)$, and $TiCl_2(NPh)$. However, during the optimization using $py_2TiCl_2(NPh)$, the pyridine would desorb; therefore, we only considered the no and 1 pyridine cases. Figure S57 compares the reaction mechanism for 1 pyridine and no pyridine. In general, we see a similar trend as we saw with the [2+2+1] pyrrole synthesis, which is that a 1 pyridine case is lower in energy. The principle exception are during the ligand rearrangement (**TS4**) which is not surprising as additional ligands are required to move to facilitate the 1,4-H migration. It is worth noting here that **TS5** for 1 pyridine has a lower free energy than **IM6**. The electronic energy for **TS5** is indeed higher than **IM6**; therefore, **TS5** is a true transition state. However, the free energy correction lowers the free energy of **TS5** more than **IM6**, leading to **TS5** being lower than **IM6**.

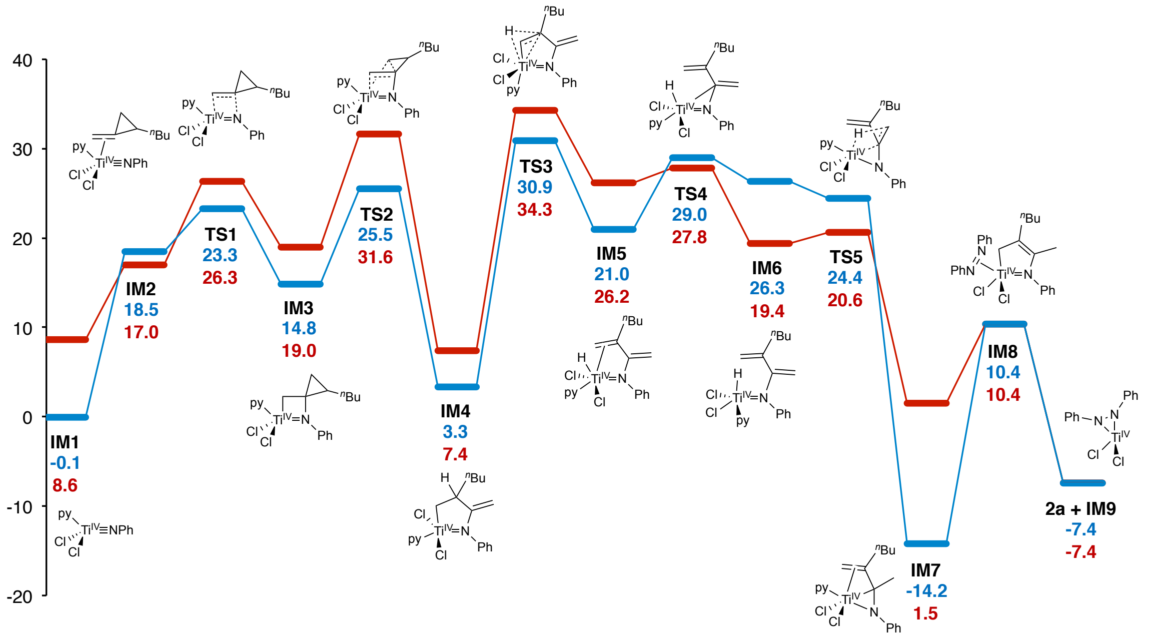

Figure S57. Comparison of no pyridine (red) verse 1 pyridine (blue) for α–methylene imine formation via ring-opening oxidative amination of MCPs. Calculations were performed at the M06/6-311(d,p)/SMD level of theory.

N-H reductive elimination

We considered multiple pathways for α–methylene imine formation via ring-opening oxidative amination of MCPs. One of the pathways considered was the N-H reductive elimination after β-H elimination to form a Ti hydride instead of ligand rearrangement and a 1,4-H migration to the α,β-unsaturated imine adduct. These two pathways are compared in Figure S58. Here, we see that the reaction barrier for N-H reductive elimination (**TS4-NH**) to be significantly higher (54.7 kcal/mol) compared to ligand rearrangement (27.8 kcal/mol) and a 1,4-H migration (20.6 kcal/mol) (**TS4** and **TS5**). Additionally, the N-H reductive elimination pathway requires moving through a tautomerization (**TS5-NH**), which also has a high barrier of 50.2 kcal/mol. Therefore, this pathway likely does not play a significant role in the formation of α–methylene imine.

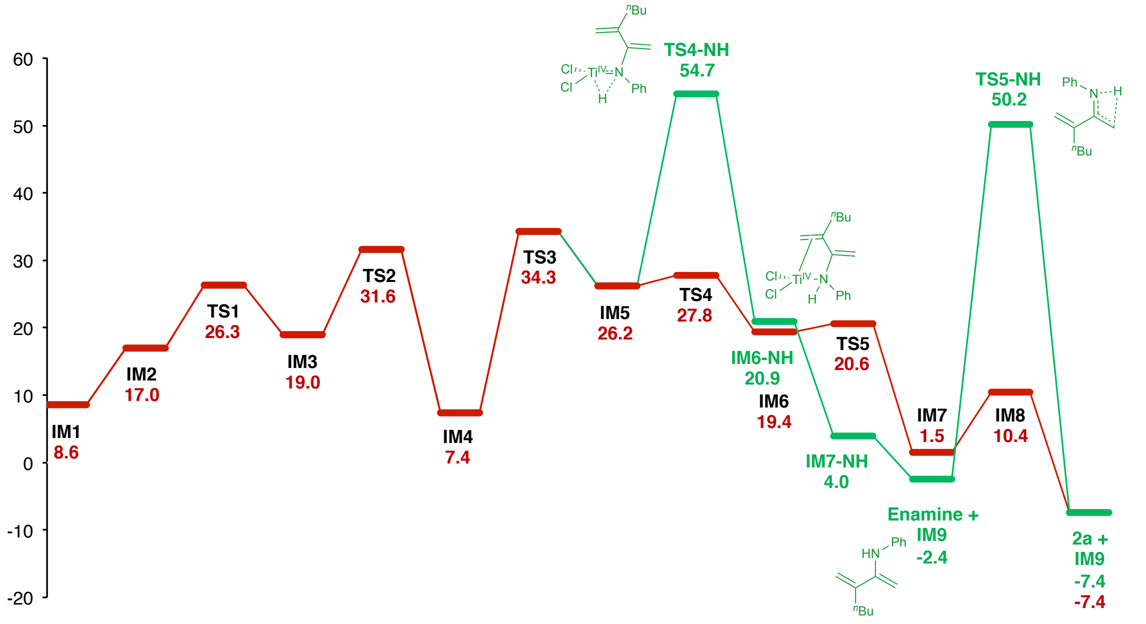

Figure S58. Comparison of N-H reductive elimination pathways to ligand rearrangement and 1,4-H migration mechanisms. Calculations were performed at the M06/6-311(d,p)/SMD level.

β-carbon elimination with no pyridine ligand

In addition to the 1 pyridine ligand considered for **β**-carbon elimination, we also considered the case where the pyridine ligand dissociated prior to **β**-carbon elimination. Generally, the barriers for the pyridine ligand remaining associated with the catalyst are lower in energy compared to the ligand dissociating. However, the same conclusions are found here. For **1a**, ring-opening of the substituted C-C bond to form **IM4'** is kinetically preferred (**TS2'** = 24.8 kcal/mol vs. **TS2** = 31.6 kcal/mol), but ultimately, **IM4** (7.4 kcal/mol) is significantly more stable than **IM4'** (13.0 kcal/mol) (Figure S59).[8] Since the subsequent β-H elimination is rate-determining, the C-C ring opening via β-C elimination proposed to be reversible, leading to thermodynamic control of the regioselectivity favoring formation and further reaction of **IM4**. In contrast, for **1b** ring-opening of the substituted benzylic C-C bond to form **IM4'** is both kinetically and thermodynamically favored, leading to the opposite regioselectivity compared to **1a** (Figure S60)

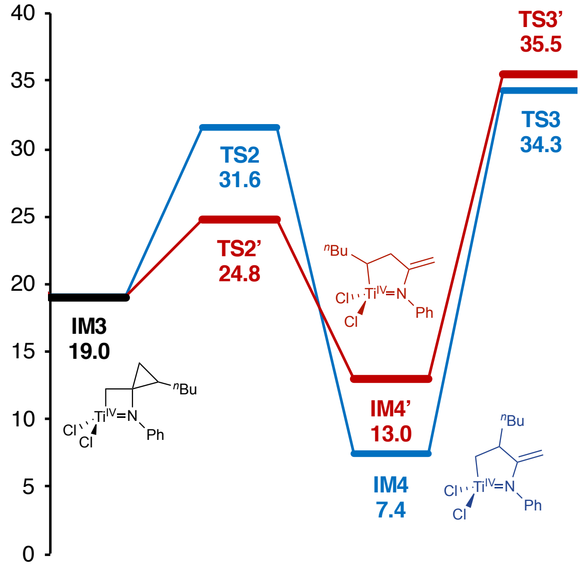

Figure S59. β-carbon elimination from **1a** thermodynamically favors formation of **IM4** over **IM4'**, and kinetic formation of **IM4'** is reversible (M06/6-311G(d,p), SMD $PhCF_3$, 388.15 K). $L_n = Cl_2$.

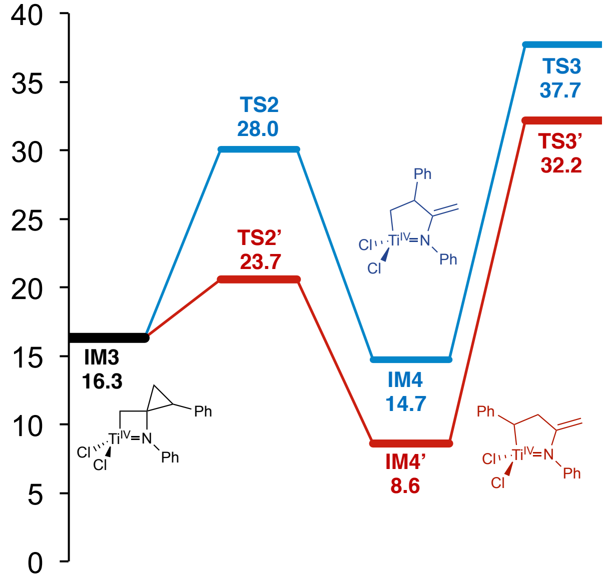

Figure S60. β-carbon elimination from **1b** both kinetically and thermodynamically favors formation of **IM4'** over **IM4** (M06/6-311G(d,p), SMD $PhCF_3$, 388.15 K). $L_n = Cl_2$.

Selectivity of $Ti(NMe_2)_4$

Eisen showed selectivity of the opposite regioselectivity of C-C ring-opening using **1b.**[22] Therefore, to better understand the differences in the regioselectivity, we computationally explored the key steps in the mechanism that dictate regioselectivity control. Eisen studied both $(Ph_2PNpy)_2Ti(NEt_2)_2$ and $Ti(NMe_2)_4$ and showed similarity reactivity of both these precatalysts with **1b** and $PhNH_2$. Here, we study the regioselectivity of $Ti(NMe_2)_4$ due to the large computational cost involved with the significantly larger $(Ph_2PNpy)_2Ti(NEt_2)_2$ catalysts.

Figure S59 shows the key steps of the mechanism using $Ti(NMe_2)_4$ as the catalyst. We begin our analysis after the formation of $Ti(NMe_2)_2NPh$ (**IM2**) and considered the [2+2] insertion, ring-opening of the C-C bond (**IM3**), and subsequent β-H elimination. In similarity with the $py_3TiCl_2(N^tBu)$ catalyst, we see that ring-opening of the substituted benzylic C-C bond is both kinetically and thermodynamically favorable. The ring-opening of substituted benzylic C-C is 5.0 kcal/mol compared to the unsubstituted benzyltic C-C bond of 7.0 kcal/mol. Additionally, the resulting intermediate of the ring-opening of substituted benzylic C-C bond is thermodynamically more stable by 6.6 kcal/mol.

The barrier resulting β-H elimination (**TS3**) is very large at 41.2 kcal/mol, indicating that the $Ti(NMe_2)_4$ catalyst can not proceed though the same mechanism as $py_3TiCl_2(N^tBu)$. It requires a protonolysis reaction step, which like occurs from was an additional $PhNH_2$. Therefore, a key difference between $Ti(NMe_2)_4$ and $py_3TiCl_2(N^tBu)$ is the ability to perform the rate determine step of β-H elimination.

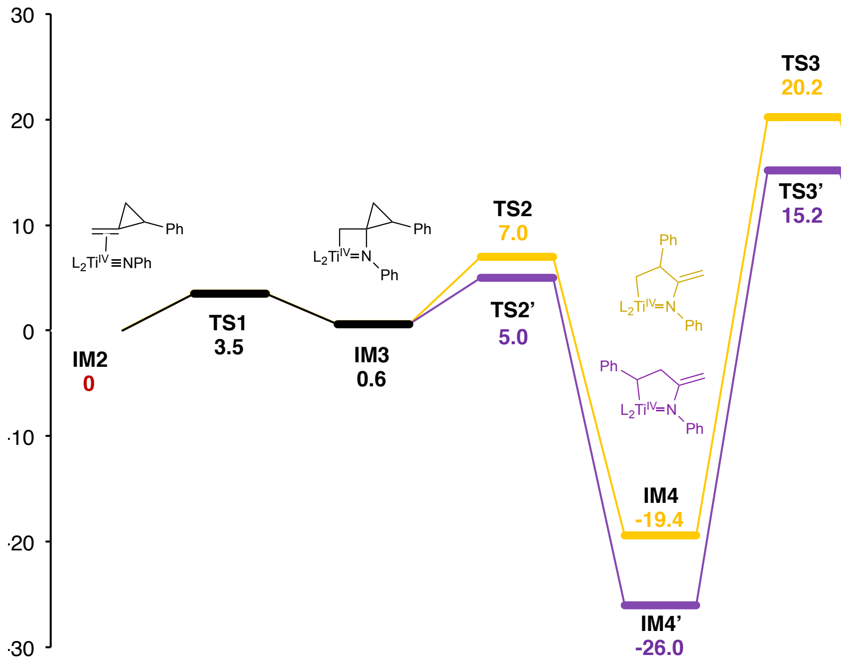

Figure S61. Comparison of β-carbon elimination using $Ti(NMe_2)_4$ as the precatalyst. Using $Ti(NMe_2)_2NPh$ (L= NMe_2) as the starting intermediate IM2 the substituted benzylic C-C ring opening is favored both kinetically and thermodynamically. Calculations were performed at the M06/6-311(d,p)/SMD level of theory.

NBO analysis

We performed natural bonding orbital analysis for both the $Ti(NMe_2)_4$ and $py_3TiCl_2(N^tBu)$ catalysts to see if there is any significant change to the formal oxidation state during the catalytic cycle or significant differences between the two catalysts. We previous benchmarked the correlation between the formal oxidation state of Ti with the occupation of the $3d_{z^2}$ orbital showing that occupation numbers closer to 0.4 have a +4 formal oxidation state and 0.6 have a +2 formal oxidation state.[3] In Table S2, S3, and S4 we show the NBO analysis for the geometries considered in Figure 4, 5 and S59. For Tables S2 and S3, we see no significant differences between either pathway for **1a** or **1b,** suggesting the selectivity is not influenced strongly by d orbital occupation of titanium. Table S4 shows that in general the $Ti(NMe_2)_4$ catalyst has slightly lower d orbital occupancy, which might suggest why the barrier for β-carbon elimination is lower. However, the oxidation state is still clearly +4, and therefore, we don't believe this gives any significant insight into the selectivity of this catalyst.

Table S2. Occupation Number of the $3d_{z^2}$ Orbital Compared with the Formal Oxidation State of the Ti Atom in the Complex for the $TiCl_2(NPh)$ catalyst (Figure 4).

Species	Occupation numbers ($3d_{z^2}$ orbital)	Formal Oxidation State
IM3	0.37	+4
TS2	0.44	+4
IM4	0.40	+4
TS3	0.44	+4
TS2'	0.45	+4
IM4'	0.41	+4
TS3'	0.45	+4

Table S3. Occupation Number of the $3d_{z^2}$ Orbital Compared with the Formal Oxidation State of the Ti Atom in the Complex for the $TiCl_2(NPh)$ catalyst (Figure 5).

Species	Occupation numbers ($3d_{z^2}$ orbital)	Formal Oxidation State
IM3	0.41	+4
TS2	0.41	+4
IM4	0.39	+4

TS3	0.45	+4
TS2’	0.43	+4
IM4’	0.41	+4
TS3’	0.44	+4

Table S4. Occupation Number of the $3d_{z^2}$ Orbital Compared with the Formal Oxidation State of the Ti Atom in the Complex for the $Ti(NMe_2)_4$ catalyst (Figure S59).

Species	Occupation numbers ($3d_{z^2}$ orbital)	Formal Oxidation State
IM2	0.27	+4
TS1	0.29	+4
IM3	0.34	+4
TS2	0.36	+4
IM4	0.34	+4
TS3	0.34	+4
TS2’	0.32	+4
IM4’	0.34	+4
TS3’	0.32	+4

Cartesian Coordinates in Å, Electron Energies and Free Energies with shifted frequency correction for the optimized structures.

I. Computational analysis of the catalytic reaction (Figure 2, Figure S57)

IM1 – 1py
Electronic energy -2304.456165 a.u.
Gibbs free energy -2304.333089 a.u.
26

Ti	2.110759858833	3.266127704031	0.257088724657
Cl	1.754557249333	3.196406912540	2.518948018122
Cl	3.694588610885	4.779535050569	-0.418081793765
N	2.619939528339	1.739456092897	-0.201378894331
C	3.137487177967	0.481491194417	-0.390323459514
C	3.137169485328	-0.109089418876	-1.662473743967
C	3.654820700136	-1.381848127832	-1.842701711034
C	3.674330779858	-0.236130718479	0.689268715675
C	4.192681542342	-1.505504672054	0.495546339410
C	4.184925613731	-2.086723422046	-0.768091762804
H	3.648353708691	-1.826952425969	-2.833802393185
H	2.731541067178	0.452659120805	-2.499714752333
H	4.592487433543	-3.082345475911	-0.914252395951
H	4.607925113289	-2.047983134219	1.340382950534
H	3.676908702603	0.225526934726	1.673124673518
N	0.291798831553	3.500814939019	-0.829466785287
C	-0.354596449158	4.674328877278	-0.729558081445
C	-1.546316459629	4.914043258167	-1.381413178368
C	-2.090558383710	3.904114903708	-2.161736665467
C	-1.425710064277	2.691008839140	-2.264372325820
C	-0.235855745117	2.523384457401	-1.585873522280
H	0.110360169841	5.431809215042	-0.101626791658
H	-2.035491139772	5.874692243887	-1.274559518611
H	-3.027671704649	4.061710570629	-2.685731610485
H	-1.820784830551	1.878697442785	-2.862816222265
H	0.323309203413	1.595159638345	-1.630233813346

IM2 – 1py
Electronic energy -2617.508800 a.u.
Gibbs free energy -2617.210311 a.u.
48

Ti	1.272500098460	1.305635473369	-0.672176033204
Cl	1.119788548081	1.717003778087	1.585363880007
Cl	2.965499964136	2.741381815541	-1.289729897770
N	2.126217845623	-0.145031403786	-0.728390070308
C	3.050401782702	-1.157318261720	-0.756665977923
C	3.944771040939	-1.276179287897	-1.831692030806
C	4.902899107874	-2.277223946979	-1.838654199150
C	3.141856833044	-2.073956566145	0.302305755388
C	4.098860314362	-3.075429738063	0.280192541661
C	4.983822299190	-3.184427200969	-0.787878930645
H	5.593369111817	-2.351361565034	-2.674394632613
H	3.876204398077	-0.562734759130	-2.648928313933
H	5.733663985946	-3.969799475745	-0.798590151931
H	4.158420022167	-3.776886520625	1.108138585625
H	2.456520926686	-1.973620194132	1.140778645668
C	-1.840438125353	0.043530401281	-1.056444886855
C	-1.420126847471	-1.163040967528	-0.726945976821
C	-0.880064804090	-2.039834005924	0.308050479847
C	-1.134380661676	-2.540494144985	-1.113025854358
C	0.038160715717	-2.883514991123	-1.997749539837
C	0.644740999542	-4.237127405899	-1.666161781140
H	-0.276667362075	-2.866508769126	-3.051421283435
H	0.804289494991	-2.101678978210	-1.889903345077
C	1.859562492919	-4.567025803912	-2.516190422615
H	0.934428609241	-4.250058116940	-0.603468826758
H	-0.117873603950	-5.022975018218	-1.784487282202
C	2.486900778820	-5.899532143080	-2.152959596352
H	2.784563003444	-5.917570989980	-1.096993021280
H	3.381289136444	-6.106109664663	-2.750807797180
H	1.783540019507	-6.726811992840	-2.309435141593
H	2.602475747990	-3.764816581710	-2.396688157966
H	1.573732777259	-4.564003632192	-3.578361208125
H	-1.535351577650	-2.380625959138	1.105850134800
H	0.159897946639	-1.889957751301	0.598993488787
H	-2.001403909825	-3.195075350827	-1.219317958548
H	-1.897985384527	0.840600645959	-0.317264129162
H	-2.191263217597	0.274190991642	-2.060824843002
N	-0.295382047753	2.877964821311	-1.332786729080
C	-1.008125998899	3.656795386894	-0.510925125843
C	-1.935567043523	4.571274522280	-0.976954573190
C	-2.133983470924	4.684183886132	-2.344326804560
C	-1.393435513335	3.883075144927	-3.201411555834
C	-0.485241219129	2.997562995224	-2.653964964771
H	-0.825702166254	3.534168089708	0.553712636330
H	-2.490632471640	5.181208486410	-0.273574163293
H	-2.856845477202	5.390811570686	-2.739360983081
H	-1.513494727979	3.941112137352	-4.276995485619
H	0.119327629237	2.353231041017	-3.290854472253

TS1 – 1py
Electronic energy -2617.504579 a.u.
Gibbs free energy -2617.202684 a.u.
48

Ti	0.751731495131	1.018914038883	-1.044170152476
Cl	0.623914555103	1.735864646749	1.235417986966
Cl	0.983204098994	1.122753846829	-3.396280808376
N	1.945476656619	-0.140049688670	-0.754095680881
C	2.997169444978	-0.947225473229	-0.410892105076
C	3.903901240740	-1.395693052691	-1.385482869303
C	4.939940232429	-2.245539458954	-1.037543565625
C	3.169932089411	-1.363960623133	0.919282993801
C	4.209523671307	-2.217207013474	1.254805569181
C	5.094796260782	-2.664967386765	0.280705507562
H	5.631754487878	-2.589602361189	-1.801752423624
H	3.769959156042	-1.066908007853	-2.413297021203
H	5.907042522127	-3.334348226735	0.547378202193
H	4.331318221780	-2.535113772480	2.286385655237
H	2.476663114998	-1.000392562538	1.673280903333
C	-1.331584726508	-0.045612012162	-0.965566320884
C	-0.756847609901	-1.245666270417	-0.779772799565
C	-0.316517657213	-2.273576475151	0.148559233253
C	-0.495139738506	-2.565700425540	-1.340670411343
C	0.680714921141	-2.909292776447	-2.223934118182
C	1.319556864970	-4.237678057023	-1.855865542213
H	0.335838166265	-2.933963066903	-3.267106240723
H	1.430191116761	-2.112402922312	-2.167769386469
C	2.409821798027	-4.656280142704	-2.827621556939
H	1.744971788787	-4.161683854919	-0.842532743586
H	0.549866042661	-5.023628396527	-1.804438520859
C	3.152566366622	-5.896013209173	-2.366620430248
H	3.653851909903	-5.716833269469	-1.407029656892
H	3.915975005904	-6.209334857886	-3.086303008613
H	2.464606137551	-6.738791821516	-2.224278884983
H	3.117592178801	-3.823153384194	-2.951764908671
H	1.965601578809	-4.827621866070	-3.818666432898
H	-1.040056753623	-2.653579112493	0.865572439955
H	0.708421584728	-2.225147349504	0.511926978617
H	-1.416775349212	-3.094818524544	-1.592873843240
H	-1.643088125535	0.557593152795	-0.111530635367
H	-1.752936595693	0.198923637274	-1.939829681463
N	-0.484827581783	3.041053726269	-1.414459242490
C	-0.214062854666	4.155730949375	-0.721837075342
C	-0.856609974840	5.361097030860	-0.943099808123
C	-1.839626020930	5.425080856772	-1.916683601794
C	-2.128998138701	4.279278984355	-2.641441604330
C	-1.425146639729	3.121427283387	-2.366718067389
H	0.544350169579	4.073623395265	0.050082987786
H	-0.587335676539	6.228707066243	-0.351053467162
H	-2.369934270479	6.352274272647	-2.110046812714
H	-2.885815304492	4.275748593948	-3.417888967558
H	-1.617558860473	2.220203941011	-2.941157061279

IM3 – 1py
Electronic energy -2617.518496 a.u.
Gibbs free energy -2617.216286 a.u.
48

Ti	1.072247000000	1.655620000000	-0.969214000000
Cl	1.209594000000	2.068132000000	1.260941000000
Cl	2.483114000000	2.909824000000	-2.270666000000
N	1.840068000000	-0.056823000000	-0.987234000000
C	3.120203000000	-0.545411000000	-0.664599000000
C	4.100241000000	-0.601963000000	-1.655964000000

C	5.358593000000	-1.106016000000	-1.365638000000
C	3.432312000000	-0.955260000000	0.632969000000
C	4.692752000000	-1.462949000000	0.915462000000
C	5.656382000000	-1.547895000000	-0.081861000000
H	6.110654000000	-1.157924000000	-2.147736000000
H	3.847976000000	-0.269088000000	-2.659428000000
H	6.641309000000	-1.945644000000	0.143608000000
H	4.925899000000	-1.787239000000	1.925670000000
H	2.680850000000	-0.871815000000	1.412884000000
C	-0.370779000000	0.220196000000	-1.481697000000
C	0.620319000000	-0.837527000000	-1.085384000000
C	0.242843000000	-1.917722000000	-0.117595000000
C	0.506688000000	-2.259133000000	-1.567144000000
C	1.662475000000	-3.164514000000	-1.901855000000
C	1.290780000000	-4.631659000000	-1.745222000000
H	2.007849000000	-2.978621000000	-2.930291000000
H	2.517648000000	-2.946411000000	-1.248736000000
C	2.440553000000	-5.576799000000	-2.049185000000
H	0.940866000000	-4.804579000000	-0.715237000000
H	0.436938000000	-4.869704000000	-2.398781000000
C	2.066535000000	-7.033488000000	-1.849322000000
H	1.759360000000	-7.221670000000	-0.813228000000
H	2.900428000000	-7.706412000000	-2.074573000000
H	1.227093000000	-7.317380000000	-2.495850000000
H	3.296009000000	-5.319106000000	-1.407593000000
H	2.778949000000	-5.415116000000	-3.082908000000
H	-0.774173000000	-1.918908000000	0.265832000000
H	1.000477000000	-2.261490000000	0.583380000000
H	-0.382586000000	-2.428884000000	-2.177489000000
H	-1.298171000000	0.247305000000	-0.902001000000
H	-0.561136000000	0.252448000000	-2.562015000000
N	-0.559645000000	3.223471000000	-1.205807000000
C	-0.315793000000	4.447573000000	-0.710228000000
C	-1.188806000000	5.505745000000	-0.867036000000
C	-2.367806000000	5.300378000000	-1.567577000000
C	-2.625408000000	4.041520000000	-2.084919000000
C	-1.700016000000	3.034140000000	-1.882474000000
H	0.615712000000	4.582010000000	-0.167377000000
H	-0.940871000000	6.472313000000	-0.444130000000
H	-3.075070000000	6.111391000000	-1.708189000000
H	-3.532149000000	3.831862000000	-2.640553000000
H	-1.875356000000	2.038449000000	-2.274739000000

TS2 – 1py
Electronic energy -2617.501906 a.u.
Gibbs free energy -2617.199249 a.u.
48

Ti	0.510147880136	-1.768926050188	0.137728198370
Cl	0.170892031176	-2.746134556877	-1.941105143093
Cl	2.811528961652	-1.704388353179	0.318718002951
N	0.200933736573	0.139453345781	0.045899199491
C	0.928005127944	1.311579177010	-0.086254685655
C	0.798853744158	2.381301729573	0.808393672152
C	1.540567203663	3.538535924790	0.622082255684
C	1.802055666570	1.439481025011	-1.174407199253
C	2.536409033136	2.598687853819	-1.347678929050
C	2.413878915440	3.656183774272	-0.451224749657
H	1.431272913207	4.358469552028	1.326466202233
H	0.115248963935	2.298641605263	1.649467317418
H	2.990535093065	4.564939691825	-0.592629373196
H	3.210701597808	2.680342447282	-2.195434399522
H	1.885840093947	0.616140458543	-1.879307712660
C	0.190405643554	-0.780713916344	2.175884569598
C	-0.594820549133	-0.159735200452	1.180480954557
C	-1.759169819379	-1.438554050421	0.386162797100
C	-2.065320936573	-0.126728790576	1.079320766427
C	-2.704921205106	0.982633442493	0.271430769671
C	-4.225749478303	0.926249925434	0.267181730752
H	-2.385993560931	1.948276517805	0.685995477273
H	-2.322507003118	0.946169842777	-0.758286193115
C	-4.822796885671	-0.313080412453	-0.382013515575
H	-4.592924125570	1.001015949600	1.302926780459
H	-4.609213977977	1.816146148638	-0.252166408726
C	-6.338657901931	-0.259958698625	-0.423729093201
H	-6.757675755993	-0.177205224933	0.586788113895
H	-6.767260448177	-1.154259095116	-0.887719362167
H	-6.686621867528	0.609716389136	-0.994428643421
H	-4.500053252790	-1.212031555622	0.161207429297

H	-4.423441127229	-0.415554529658	-1.401679204626
H	-2.054045720616	-2.330443011321	0.938281509864
H	-1.990727213818	-1.436617626088	-0.679043665664
H	-2.533242940115	-0.253223258615	2.060027558124
H	-0.286795887125	-1.211605261225	3.053943644205
H	1.187520003559	-0.371911719115	2.318219552448
N	0.321071777122	-3.736137047856	1.197683083104
C	-0.454825582404	-4.011138531257	2.256944662952
C	1.097142276887	-4.723691083570	0.718373684347
C	-0.486000270825	-5.252284423444	2.862089586259
C	1.126545751384	-5.989635979715	1.269661597622
C	0.319914065380	-6.262758637195	2.362396011717
H	1.774815587144	-6.743646521738	0.838909386354
H	0.319361030769	-7.247782314447	2.817674535074
H	-1.134756030265	-5.413957744541	3.714965446095
H	-1.073511385639	-3.205354051709	2.636087907941
H	1.718985828004	-4.489297154800	-0.140114124854

IM4 – 1py
Electronic energy -2617.538660 a.u.
Gibbs free energy -2617.234590 a.u.
48

Ti	1.357034913350	-1.045923352902	1.235383335938
Cl	2.829072684355	-1.871711509109	2.874062928897
Cl	-0.458783707289	-0.039333394758	0.155317712070
N	2.590192454262	-0.909860228804	-0.203522042507
C	3.569075500301	0.112323551480	-0.145870619822
C	4.904791809512	-0.189688496875	0.129242627905
C	5.837200816101	0.829946896169	0.223962377595
C	3.181558397577	1.441295672019	-0.313150847309
C	4.121316464714	2.459707948830	-0.206584903879
C	5.448558598908	2.156271571267	0.058397992166
H	6.875331903731	0.591057636844	0.435125163966
H	5.194397922911	-1.227704623426	0.268104158878
H	6.183702032101	2.951510199618	0.137073672612
H	3.812855783773	3.492360446663	-0.340171477749
H	2.142433825900	1.663192094564	-0.543495805975
C	3.392698135043	-1.834248242502	-2.288438556564
C	2.526215257537	-1.819795156457	-1.268003126068
C	0.979549051952	-2.881284215301	0.359857067887
C	1.318358369299	-2.728146304736	-1.128223600989
C	1.463065534845	-4.069157517127	-1.844107617477
C	0.239710383772	-4.961987879260	-1.723343239130
H	1.659249311919	-3.892061556485	-2.911421043511
H	2.346287239613	-4.594831184501	-1.448635856091
C	0.342415858375	-6.224014518242	-2.563650396495
H	0.083221463265	-5.245999469834	-0.672583711289
H	-0.658322491773	-4.396832551507	-2.021233701931
C	-0.859111328744	-7.134992096630	-2.395242651840
H	-0.966966450284	-7.455249164255	-1.351681134948
H	-0.780687389336	-8.036647559464	-3.011523992690
H	-1.785992248600	-6.620305214206	-2.676882852049
H	1.261023112431	-6.764099130197	-2.291313637219
H	0.456708526942	-5.947394353744	-3.621669773358
H	-0.060453003096	-3.187739104892	0.529184091136
H	1.652303665125	-3.605386163569	0.840227750540
H	0.478981310941	-2.190121310090	-1.599150535461
H	3.298592374979	-2.562127929650	-3.085162595394
H	4.205321331920	-1.119222048017	-2.362034597027
N	0.519720523276	0.021120171606	3.021062607677
C	0.995911314632	1.212159792859	3.392906080302
C	0.528166061607	1.870294932233	4.515025576883
C	-0.462980106226	1.266523062499	5.274299610225
C	-0.955872001992	0.029428861586	4.885573353978
C	-0.437081301073	-0.560504409125	3.748640697599
H	1.779267396006	1.641147167592	2.772053764526
H	0.940022429333	2.835386813641	4.786148279505
H	-0.847339522340	1.755611374021	6.163523482513
H	-1.729188115663	-0.475591915396	5.452412073430
H	-0.787615093893	-1.531547592427	3.400942910544

TS3 – 1py
Electronic energy -2617.493094 a.u.
Gibbs free energy -2617.190595 a.u.
48

Ti	0.297949395718	-1.576477329785	0.216964875268
Cl	-1.144548319668	-2.599045525617	1.751055135177
Cl	-1.333802251236	-0.410921014112	-0.999615526355
N	2.085671496989	-0.953854446480	-0.388968766471
C	2.797279517091	0.255130871734	-0.238196367414

C	4.037717301769	0.273559373825	0.405036948425
C	4.697665978634	1.473199095517	0.618573507548
C	2.241113733475	1.455405480653	-0.674705884089
C	2.903316479114	2.655805285379	-0.453354205831
C	4.131100384794	2.670662852732	0.194630783688
H	5.657014767675	1.475258649944	1.128262859838
H	4.469323564247	-0.663133219679	0.750250096998
H	4.646881871090	3.610382319711	0.367757137711
H	2.455923889904	3.585314581741	-0.793825152185
H	1.280779951735	1.435809739149	-1.183403249700
C	3.729726754179	-1.985301164416	-1.901252847706
C	2.676288607579	-2.010753510828	-1.075019810748
C	1.597210875959	-3.474665241995	0.529328560687
C	1.911130748422	-3.256292392346	-0.786308503897
C	1.682748757207	-4.255749394957	-1.875024527478
C	0.570281512706	-5.258329222761	-1.629019980442
H	1.505403112128	-3.719436330550	-2.815897622393
H	2.637125003745	-4.791009696837	-2.008511027771
C	0.324884929246	-6.148222887127	-2.836039949121
H	0.805557548797	-5.888089097109	-0.759472219737
H	-0.356105083373	-4.717323163697	-1.378635114334
C	-0.782309130527	-7.156039233088	-2.593832067609
H	-0.538462953637	-7.816044379883	-1.752539994395
H	-0.958452056705	-7.787328348078	-3.470680572847
H	-1.725830627411	-6.651889511256	-2.352437641398
H	1.256844341988	-6.669836663606	-3.097838222319
H	0.074845613402	-5.518925288937	-3.701787860506
H	1.085197566092	-4.376352311960	0.845504201799
H	2.092686373521	-2.891238620925	1.309332064440
H	0.274658661781	-2.284216591335	-1.328493155333
H	4.054602607692	-2.889664532201	-2.402833664805
H	4.286295346791	-1.073732433027	-2.089656889030
N	0.398492702451	0.195805209356	1.574210516294
C	1.321226377694	0.039183199827	2.532592388429
C	1.560514725874	0.989749190838	3.504471059891
C	0.808016966815	2.154827219654	3.488931064568
C	-0.161591396697	2.313121890562	2.511879950418
C	-0.337821436012	1.312312180072	1.573960376026
H	1.896281884083	-0.885364656051	2.505171703344
H	2.325275346146	0.816039367251	4.252403209932
H	0.972988077247	2.927115539636	4.233287319139
H	-0.778731653980	3.203004376338	2.467544833702
H	-1.082477864534	1.397939784729	0.788832230590

IM5 – 1py
Electronic energy -2617.506074 a.u.
Gibbs free energy -2617.206305 a.u.
48

Ti	1.272355000000	-1.546010000000	1.091454000000
Cl	2.217199000000	-1.774902000000	3.201095000000
Cl	-0.301458000000	-1.408897000000	-0.613266000000
N	2.859996000000	-1.447059000000	-0.000211000000
C	3.484343000000	-0.171090000000	-0.044288000000
C	4.497624000000	0.139977000000	0.859631000000
C	5.072824000000	1.403214000000	0.845431000000
C	3.052882000000	0.786563000000	-0.963548000000
C	3.632725000000	2.046279000000	-0.974027000000
C	4.640871000000	2.357078000000	-0.068094000000
H	5.862030000000	1.643644000000	1.551504000000
H	4.821477000000	-0.616546000000	1.569065000000
H	5.091589000000	3.345012000000	-0.075290000000
H	3.293556000000	2.790615000000	-1.688354000000
H	2.262988000000	0.527205000000	-1.664604000000
C	4.051039000000	-2.241624000000	-1.968870000000
C	3.168090000000	-2.390083000000	-0.971221000000
C	2.250096000000	-4.180090000000	0.400101000000
C	2.320297000000	-3.602490000000	-0.805721000000
C	1.542805000000	-4.047053000000	-2.005960000000
C	0.471086000000	-5.092838000000	-1.765233000000
H	1.085859000000	-3.152507000000	-2.455880000000
H	2.256629000000	-4.409836000000	-2.762334000000
C	-0.387557000000	-5.321353000000	-2.998373000000
H	0.926827000000	-6.043766000000	-1.452004000000
H	-0.175984000000	-4.772857000000	-0.934489000000
C	-1.456006000000	-6.374750000000	-2.776992000000
H	-1.009341000000	-7.343479000000	-2.522115000000
H	-2.077495000000	-6.519975000000	-3.666641000000
H	-2.120006000000	-6.092142000000	-1.951013000000
H	0.254548000000	-5.609074000000	-3.843528000000
H	-0.857159000000	-4.368376000000	-3.283893000000
H	1.612513000000	-5.036473000000	0.592930000000
H	2.899642000000	-3.868243000000	1.214103000000
H	0.415075000000	-2.918397000000	1.525941000000
H	4.198380000000	-3.041262000000	-2.686343000000
H	4.650253000000	-1.344278000000	-2.081167000000
N	0.332771000000	0.235555000000	1.897745000000
C	0.928669000000	1.434882000000	1.905860000000
C	0.347959000000	2.534414000000	2.507935000000
C	-0.882821000000	2.384107000000	3.127523000000
C	-1.495517000000	1.139424000000	3.122024000000
C	-0.857825000000	0.088153000000	2.494728000000
H	1.897694000000	1.500870000000	1.417696000000
H	0.862371000000	3.487902000000	2.491921000000
H	-1.360268000000	3.228623000000	3.613555000000
H	-2.455517000000	0.977938000000	3.597822000000
H	-1.298459000000	-0.905270000000	2.467825000000

TS4 – 1py
Electronic energy -2617.494559 a.u.
Gibbs free energy -2617.193693 a.u.
48

Ti	0.964892573838	0.043636851677	0.190163993512
Cl	1.560914680972	-0.154134477563	2.413909258248
Cl	1.233057701086	2.127046260739	-0.712170497763
N	2.657992211948	-0.603410569873	-0.411133079006
C	4.027268460351	-0.410880518356	-0.281685721934
C	4.951674299697	-1.123669160273	-1.058253210464
C	6.310473833567	-0.894756575366	-0.918112379446
C	4.509193585653	0.539595582096	0.629219255517
C	5.870341097797	0.759572110322	0.758583764913
C	6.781698504326	0.045214633385	-0.009740633555
H	7.009446226237	-1.458294814039	-1.529828240567
H	4.597825144426	-1.856646950895	-1.777551472008
H	7.847567157203	0.222576930837	0.094762379585
H	6.222273761200	1.502748417870	1.468420753264
H	3.806606143391	1.105216025904	1.232367865891
C	1.698301208446	-1.057302785188	-2.527363105885
C	2.137304704149	-1.537576580922	-1.349317358588
C	2.366899526891	-3.319899990825	0.297632703809
C	1.984304442902	-2.948867389055	-0.927573955441
C	1.369374778819	-3.891106261443	-1.922794652896
C	1.224903584195	-5.336343616017	-1.485801665974
H	0.376324454980	-3.501235081390	-2.199182097162
H	1.957568885879	-3.853812312758	-2.853181606070
C	0.585031425712	-6.199973968275	-2.560615928167
H	2.210228226641	-5.751511958458	-1.227178197017
H	0.618209027803	-5.393357039284	-0.570060067150
C	0.439215494985	-7.647297688992	-2.130688402185
H	1.415697843905	-8.089776765268	-1.899275475440
H	-0.026240634627	-8.260314565085	-2.909255917098
H	-0.179830022652	-7.729683169692	-1.229045539449
H	1.187439217697	-6.141890030183	-3.478589304836
H	-0.400477253228	-5.785801557276	-2.818027172471
H	2.267725239141	-4.343835914480	0.641198615257
H	2.800295554928	-2.611684610271	0.996773003453
H	-0.342274350512	-0.842461977172	-0.415271248255
H	1.200027803924	-1.681553268923	-3.260891797214
H	1.857013755292	-0.014366019186	-2.784078344364
N	-0.932335284477	0.967792042484	1.189722241738
C	-0.967829732896	2.212799569281	1.682771224176
C	-1.979863682517	0.169222099863	1.437429117670
C	-2.031782044634	2.701406290714	2.417825752758
C	-3.112255004350	1.869438241623	2.665444840868
C	-3.082047712242	0.577366556153	2.166427326894
H	-2.003616062627	3.719326322402	2.789541809349
H	-0.105085402366	2.840093863366	1.480291047467
H	-1.925144239475	-0.836433633727	1.032255329466
H	-3.899855114900	-0.114349931109	2.333578523954
H	-3.962894016483	2.222097382627	3.239948262614

IM6 – 1py
Electronic energy -2617.498259 a.u.
Gibbs free energy -2617.197808 a.u.
48

Ti	-0.044000191365	0.629355043679	-0.856007068834
Cl	-1.263739634728	-0.983134922165	-1.828987312204
Cl	-1.617560833010	2.373530427432	-0.803303843905
N	1.741919982508	-0.085980255355	-0.696923625229
C	2.824307243424	0.648903954352	-0.173006658246
C	3.780323488725	0.044277794382	0.651898479140

C	4.787213244918	0.802986304728	1.225211084523
C	2.925811944290	2.019026653262	-0.426384115147
C	3.935966778101	2.774054304405	0.154576054841
C	4.867538914351	2.171948962200	0.987476571360
H	5.518180895847	0.320580990467	1.867978730972
H	3.722258326581	-1.023596428382	0.844479653049
H	5.660332647302	2.759841211252	1.439671177326
H	3.998713963786	3.837650021714	-0.057291404656
H	2.208561472573	2.479904948861	-1.103346136444
C	2.704032037176	-1.195246072172	-2.578492998841
C	2.052545143069	-1.289884950483	-1.415525037089
C	1.378980617358	-2.675639393055	0.481659715077
C	1.615639274366	-2.577069927579	-0.828896790789
C	1.460881160905	-3.734448395835	-1.776594496650
C	0.785854114502	-4.972347468245	-1.216514363068
H	0.893931378470	-3.383238838211	-2.652318790649
H	2.450783441202	-4.011797106971	-2.171271474255
C	0.602866207883	-6.055856416645	-2.266570092642
H	1.369493993707	-5.380654453632	-0.378426860603
H	-0.197487075855	-4.699231583184	-0.803079072646
C	-0.076656040548	-7.292670744514	-1.710067631835
H	0.508600368216	-7.729984146693	-0.891916252719
H	-0.208029120008	-8.066263060246	-2.473589298511
H	-1.068661058361	-7.049735840232	-1.310078168229
H	1.583401270272	-6.324322048916	-2.685618119720
H	0.015564142642	-5.651833416262	-3.103504337137
H	1.066370465852	-3.606595148288	0.942723700062
H	1.509683027322	-1.824355161723	1.141908014136
H	0.449907246311	1.381390055156	-2.268741880161
H	2.982551317564	-2.069853941568	-3.155752050674
H	2.991136465117	-0.223241888815	-2.970165446256
N	0.156014322372	1.137589612746	1.287926733651
C	0.645938285585	2.273520023824	1.794995601875
C	0.913421575586	2.416951024558	3.143794598633
C	0.639128003992	1.357432546019	3.995105405605
C	0.091340335620	0.193994592386	3.474109648009
C	-0.129394581076	0.122386681269	2.112304672866
H	0.830454438554	3.081846895730	1.094061989759
H	1.327131248156	3.347922288495	3.513384545447
H	0.841846068796	1.440555307537	5.057988218588
H	-0.154889969149	-0.650710093813	4.106676107551
H	-0.542976348901	-0.772337941466	1.649312624667

TS5 – 1py

Electronic energy -2617.499550 a.u.

Gibbs free energy -2617.200865 a.u.

48

Ti	0.980926044312	2.094812572970	-0.122419215622
Cl	0.347017798874	1.383131985743	1.985560842969
Cl	1.924569674720	4.180231026518	-0.535306047914
N	2.753637054757	1.311358204819	-0.063211947248
C	4.011325472031	1.396670821606	0.517342356515
C	5.082600187321	0.628737948854	0.045581865918
C	6.338582192902	0.762374691590	0.612835569947
C	4.231635483110	2.302133816898	1.559715768873
C	5.493693969648	2.429574357491	2.118335877530
C	6.553180113621	1.660774664076	1.652525860128
H	7.161172934885	0.161312769966	0.235523160645
H	4.918462648877	-0.065072470049	-0.774611320506
H	7.540020828887	1.762415021173	2.093490480296
H	5.649224263269	3.135450646753	2.929186477975
H	3.400417847581	2.896094443775	1.928944179693
C	1.418636126503	0.550852027034	-1.788673729485
C	2.182445682006	0.213404618132	-0.663413184408
C	2.508145022624	-1.307670255708	1.231286999398
C	2.143446851354	-1.133316322156	-0.039967928239
C	1.714751393441	-2.264378315873	-0.936590276100
C	1.845018094728	-3.663082657557	-0.364775172430
H	0.665781064216	-2.104490669123	-1.231953529852
H	2.285820094789	-2.202693089081	-1.876291151219
C	1.406862152143	-4.732228483717	-1.352445526172
H	2.887232483946	-3.849679851345	-0.065413653396
H	1.242946829994	-3.752673961059	0.551343139192
C	1.529897329958	-6.132867340860	-0.783199925694
H	2.566815900984	-6.354404183336	-0.502840924102
H	1.210419480603	-6.895626677176	-1.500694477727
H	0.915327363949	-6.246849029438	0.118035153716
H	2.007940078249	-4.647533384406	-2.269238787236
H	0.366453667374	-4.541966704181	-1.653296645030
H	2.530314724689	-2.289941659775	1.691221064667
H	2.787243060361	-0.472146684198	1.864062345070
H	0.047337590218	1.473743702336	-1.412016853079
H	0.789612464506	-0.180830304120	-2.283147276514
H	1.806255743675	1.335948339972	-2.438712362739
N	-0.931040170586	3.366911272889	0.072407550628
C	-1.007898230785	4.320917055656	1.011532062141
C	-2.003422298177	3.166152618786	-0.703028699578
C	-2.134648887766	5.094871956381	1.206789235337
C	-3.240915385992	4.880742966284	0.397490598266
C	-3.172048551804	3.897712030460	-0.574827138513
H	-0.127448582921	4.467440260539	1.630994694641
H	-2.138531557518	5.851522715186	1.983128197963
H	-4.142130834066	5.471981839479	0.524861171035
H	-4.008658642546	3.691026025319	-1.232520442930
H	-1.917646572939	2.387881642474	-1.454958436807

IM7 – 1py

Electronic energy -2617.570641 a.u.

Gibbs free energy -2617.262547 a.u.

48

Ti	0.628412088174	-1.008497786838	0.233636490279
Cl	0.657362667786	0.724794561903	1.736238901912
Cl	-0.016926066120	-0.266720936486	-1.882198707272
N	2.526201078045	-0.948095608042	-0.017722446868
C	3.672614899820	-0.160331687725	-0.083509885623
C	4.946778700303	-0.737126584115	-0.047040942926
C	6.076854182448	0.063046627376	-0.096998126216
C	3.557120815964	1.229715581733	-0.176585116373
C	4.693615817941	2.021527242751	-0.220104381365
C	5.957682346750	1.445342687706	-0.181812212127
H	7.060268119088	-0.397201588346	-0.064038021297
H	5.039308666981	-1.816877949730	0.037210746625
H	6.845225637522	2.069516504125	-0.219611952895
H	4.589615165904	3.100188028306	-0.294241709780
H	2.568225703735	1.675288793652	-0.222796538932
C	2.952368743557	-2.339117732319	-2.046713567950
C	2.394387354616	-2.171931394371	-0.663701218569
C	0.973142319857	-2.832249651431	1.221681758013
C	1.586144953478	-3.125458462383	-0.046309894530
C	1.083761831388	-4.327131176909	-0.802524585145
C	-0.340580675388	-4.164725615419	-1.320997835681
H	1.750683343252	-4.565282516801	-1.638721457775
H	1.117566609968	-5.189414198092	-0.122227664746
C	-0.856302418985	-5.387371097888	-2.058301986607
H	-1.014672916665	-3.942906642203	-0.478123545606
H	-0.387735123852	-3.290744893634	-1.989608679155
C	-2.286768142566	-5.203798698918	-2.529208742554
H	-2.961011954206	-5.035889515094	-1.679998181611
H	-2.654766323799	-6.077481731628	-3.076669965552
H	-2.373031263629	-4.334711541351	-3.193459953205
H	-0.786996782227	-6.264613882679	-1.399120036729
H	-0.201114125278	-5.597383598621	-2.915643998959
H	0.257485613415	-3.556234244691	1.604033673333
H	1.612608135932	-2.382668647719	1.991377197198
H	2.189296234247	-2.701143026879	-2.742169815086
H	3.780540672511	-3.056971773760	-2.062285424603
H	3.322260122031	-1.384469358526	-2.428666630142
N	-1.613171769242	-1.324184388371	0.692919750561
C	-1.974607113011	-1.428360867044	1.981441763243
C	-3.260889537654	-1.733577775777	2.380941233946
C	-4.228018852900	-1.953250957250	1.411084324692
C	-3.865932166212	-1.850774863474	0.078557362901
C	-2.556469099681	-1.531716517137	-0.236213672475
H	-1.195157182209	-1.260107359026	2.719238933361
H	-3.492876038546	-1.799151441327	3.437788465734
H	-5.247229309699	-2.199278348117	1.691414928469
H	-4.583560555137	-2.010549307677	-0.718262689455
H	-2.245477407711	-1.435486659754	-1.272173942457

IM8 – 1py

Electronic energy -2941.843781 a.u.

Gibbs free energy -2941.450373 a.u.

61

Ti	1.287536066774	-1.451729787154	1.565160213219
Cl	-0.265272792601	-2.308432104167	3.135134851759
Cl	0.773127820767	0.807508556306	2.047773775823
N	2.415657661015	-1.200005684261	-0.040876676026
C	3.632790453630	-0.494605942056	0.140694538479
C	4.831594836167	-1.193154523085	0.320890058463
C	6.013223007545	-0.512923002509	0.569618555141

C	3.648495356258	0.898873551358	0.207569672323
C	4.833235720468	1.576471755433	0.458880400024
C	6.017323940770	0.875243811782	0.647759830863
H	6.934756499892	-1.069471253061	0.716643721476
H	4.820223546504	-2.281037341768	0.278888283515
H	6.941193296860	1.407741305987	0.852850203927
H	4.829572826456	2.661513780919	0.508505927400
H	2.719189855718	1.440876137126	0.064424294751
C	3.154691570508	-1.776340314038	-2.361852548163
C	2.232762887904	-1.962421112101	-1.202006845535
C	0.382411029705	-2.687392861237	0.089340097927
C	1.165917222105	-2.796700854351	-1.155612656412
C	0.775677089852	-3.769622114563	-2.226767826505
C	1.355843224538	-5.167144041021	-2.019127135947
H	-0.321351844300	-3.848236225229	-2.258311319657
H	1.084112542394	-3.409445333803	-3.218165048639
C	0.932396101746	-6.157111825885	-3.090125494777
H	2.456186891041	-5.099313350485	-1.997016869771
H	1.052024034454	-5.547565583163	-1.030847252053
C	1.521490243016	-7.537793619419	-2.870812382093
H	2.618125120286	-7.501373459091	-2.864322076456
H	1.218949734916	-8.242543864815	-3.652288821518
H	1.200828306843	-7.953867529992	-1.907648214272
H	1.234531974342	-5.772964138726	-4.075312142360
H	-0.165227866816	-6.217100087766	-3.111978500630
H	-0.371098333327	-1.863878230382	-0.007539482667
H	-0.170742800815	-3.588604547161	0.376912417547
H	3.293067882358	-0.711475411829	-2.586286930673
H	2.758977423004	-2.260442815044	-3.257485370892
H	4.152409004238	-2.191588708121	-2.176464139372
N	2.637624127179	-2.829941414092	2.293240228131
N	2.993213254405	-1.755641025690	2.821587460572
C	3.027431736099	-4.163108655007	2.475107956520
C	2.889712827513	-4.970670394858	1.346047210422
C	3.390033514467	-4.699612609026	3.712527810737
C	3.194214803521	-6.317227373104	1.434299371216
C	3.648955003030	-6.054976643520	3.793136420043
C	3.570593049501	-6.858502866956	2.657474546120
H	3.910342356955	-6.493572173836	4.750428500748
H	3.430086099821	-4.073659278269	4.597539679085
H	2.557921753027	-4.516585138745	0.415470268856
H	3.788730934511	-7.918980401615	2.734846519809
H	3.116958256741	-6.948045975042	0.554016009229
C	4.062262711227	-1.394859754490	3.640787193560
C	3.934777689178	-0.130485119393	4.225316644679
C	5.239026460416	-2.139433477668	3.794433647806
C	4.963088670559	0.370225068013	5.002596393748
C	6.128060720839	-0.368634476752	5.163927988672
C	6.266736183284	-1.612662879900	4.550210497740
H	3.022805267514	0.434340931397	4.053466108761
H	6.941477085050	0.028122565671	5.763182714524
H	4.862252592179	1.343672133976	5.470140887858
H	7.188832543031	-2.173112652763	4.663463937378
H	5.361782825740	-3.097263620962	3.302465895569

IM9
Electronic energy -2342.487851 a.u.
Gibbs free energy -2342.357914 a.u.
27

Ti	1.445901133547	-3.933034426012	2.229361180330
Cl	3.403360245075	-5.116176740168	2.546616812846
Cl	-0.250768727479	-3.152477906503	0.874645909404
N	1.473318016174	-2.886374550766	3.762714214193
N	0.535289050477	-3.920802052224	3.848082448618
C	2.398392395385	-2.686935093525	4.797861674709
C	3.465424413876	-1.820400377451	4.556510034742
C	4.398286018851	-1.584636161200	5.551561814462
C	4.271408213880	-2.198744249331	6.793111453351
C	2.261702784981	-3.301352403978	6.041461275451
C	3.202876697134	-3.053971949764	7.030116547325
H	3.548211933588	-1.341818104071	3.584221571394
H	5.231048595792	-0.915349634514	5.357177658328
H	5.002628836464	-2.009844878601	7.572795669082
H	3.097542392064	-3.536485147631	7.997421314285
H	1.427662157693	-3.972921636026	6.220684701269
C	-0.781039096096	-3.634837394829	4.240250524184
C	-1.736071091173	-4.642979681992	4.102796825887
C	-1.138692340308	-2.399347870619	4.777976053256
C	-3.044251760934	-4.407468810987	4.490135949828
C	-3.409295739378	-3.175016927047	5.021615769347
C	-2.452884197175	-2.177493955098	5.161701247263
H	-1.435015157644	-5.603743398161	3.693729391847
H	-4.435236419085	-2.994105776230	5.326694517427
H	-3.785785093455	-5.192817371599	4.377872983439
H	-2.730571569078	-1.212285905974	5.575183899110
H	-0.389022693176	-1.620977595697	4.881808558624

2a
Electronic energy -599.350944 a.u.
Gibbs free energy -599.120808 a.u.
34

N	4.760669000000	-1.566424000000	-2.647624000000
C	4.426871000000	-0.882786000000	-1.469273000000
C	5.449848000000	-0.458762000000	-0.619774000000
C	5.155284000000	0.272816000000	0.520780000000
C	3.108643000000	-0.528129000000	-1.173509000000
C	2.823414000000	0.219765000000	-0.040714000000
C	3.841684000000	0.617546000000	0.816930000000
H	5.960892000000	0.583170000000	1.180405000000
H	6.475626000000	-0.719624000000	-0.867133000000
H	3.614316000000	1.199604000000	1.704814000000
H	1.793704000000	0.491227000000	0.175132000000
H	2.312648000000	-0.834732000000	-1.847835000000
C	4.641542000000	-3.322115000000	-4.255187000000
C	4.327969000000	-2.735647000000	-2.915871000000
C	4.023184000000	-3.944040000000	-0.816536000000
C	3.523590000000	-3.595573000000	-1.998820000000
C	2.188415000000	-4.038899000000	-2.531636000000
C	1.251171000000	-4.669720000000	-1.520167000000
H	1.693526000000	-3.166809000000	-2.989519000000
H	2.351650000000	-4.738851000000	-3.366242000000
C	-0.096791000000	-5.026713000000	-2.124886000000
H	1.707313000000	-5.576601000000	-1.097281000000
H	1.103146000000	-3.978641000000	-0.676001000000
C	-1.035741000000	-5.659679000000	-1.115677000000
H	-0.609194000000	-6.583212000000	-0.705738000000
H	-2.004381000000	-5.910779000000	-1.559861000000
H	-1.222250000000	-4.982616000000	-0.273228000000
H	0.056619000000	-5.710158000000	-2.972516000000
H	-0.556060000000	-4.120548000000	-2.545383000000
H	3.486147000000	-4.592301000000	-0.130636000000
H	5.001808000000	-3.597940000000	-0.494956000000
H	5.047424000000	-4.335098000000	-4.153160000000
H	3.728117000000	-3.404851000000	-4.856529000000
H	5.353757000000	-2.687719000000	-4.786478000000

IM1 – 0py
Electronic energy -2056.252174 a.u.
Gibbs free energy -2056.207242 a.u.
15

Ti	1.718974801870	1.292675408667	-0.696346989681
Cl	1.538118513918	2.312796229677	1.370133696507
Cl	2.921998517628	2.843471493095	-1.895880086691
N	2.880420792759	0.133630503788	-0.436545436216
C	3.987332743609	-0.660193382397	-0.256276847436
C	4.897978177691	-0.843891005476	-1.306028290466
C	6.018400808889	-1.636980515463	-1.123049665972
C	4.224080431900	-1.287345290508	0.974069140942
C	5.348438049753	-2.078937660554	1.143691648614
C	6.248799242340	-2.258558691239	0.099281937090
H	6.719476874593	-1.770923278622	-1.941977783328
H	4.707952029919	-0.351356162052	-2.255817941697
H	7.128340985914	-2.879803976717	0.237780419742
H	5.525731246012	-2.559786247701	2.101495962930
H	3.516226783204	-1.134447424500	1.784260235662

IM1 – 0py
Electronic energy -2304.456165 a.u.
Gibbs free energy -2304.333089 a.u.
37

Ti	1.763575165965	1.354470241313	-0.685579858657
Cl	1.440921823758	1.862259905448	1.522490398705
Cl	2.789469022824	3.180420354742	-1.629989523358
N	2.952144087077	0.180095605085	-0.615832956740
C	4.044163343856	-0.616153180056	-0.364818505841
C	4.941707684718	-0.939323880106	-1.391878042850
C	6.022186065805	-1.767759737283	-1.136329055152
C	4.255410014961	-1.141265303111	0.918066238332
C	5.340067484085	-1.968010688431	1.161775100323
C	6.226194845312	-2.286790207086	0.138205024534

H	6.712217617118	-2.012484424465	-1.938814654121
H	4.768770257553	-0.531141668234	-2.384113515071
H	7.073985154906	-2.936280340407	0.333559340040
H	5.495600099489	-2.368288609129	2.159588791202
H	3.556832294425	-0.884754225084	1.710361248066
C	-0.334046744345	0.445510242074	-1.286743820844
C	-0.017625286899	-0.811738785834	-0.953616628021
C	-0.076191780558	-1.836657891012	0.075640080825
C	0.251656722881	-2.177271640026	-1.377749983662
C	1.620088501942	-2.713191219258	-1.708417157578
C	1.682000666188	-4.227456402688	-1.614689031918
H	1.920755870750	-2.383244367308	-2.713770423750
H	2.344441928460	-2.279809467488	-1.010419563929
C	3.088537696830	-4.764406482428	-1.818346734226
H	1.311529505029	-4.544204349052	-0.627171190884
H	1.001907119717	-4.676042844584	-2.354934753008
C	3.149153700316	-6.278790030410	-1.759527508409
H	2.786161922299	-6.649169248531	-0.792986290934
H	4.168849162596	-6.653212557185	-1.895671741593
H	2.522971289894	-6.729361689943	-2.539185116199
H	3.749104895872	-4.332755240669	-1.051455978924
H	3.476493451433	-4.409075752803	-2.784488893575
H	-1.044952477787	-2.072189983366	0.508600748519
H	0.767647728841	-1.899547798837	0.762776928889
H	-0.562917293918	-2.601460022940	-1.969491409735
H	-0.831386180111	1.100112308966	-0.566838999017
H	-0.312164361280	0.767219380126	-2.330493561436

IM2 – 0py
Electronic energy -2369.321220 a.u.
Gibbs free energy -2369.100611 a.u.
37

Ti	1.763575165965	1.354470241313	-0.685579858657
Cl	1.440921823758	1.862259905448	1.522490398705
Cl	2.789469022824	3.180420354742	-1.629989523358
N	2.952144087077	0.180095605085	-0.615832956740
C	4.044163343856	-0.616153180056	-0.364818505841
C	4.941707684718	-0.939323880106	-1.391878042850
C	6.022186065805	-1.767759737283	-1.136329055152
C	4.255410014961	-1.141265303111	0.918066238332
C	5.340067484085	-1.968010688431	1.161775100323
C	6.226194845312	-2.286790207086	0.138205024534
H	6.712217617118	-2.012484424465	-1.938814654121
H	4.768770257553	-0.531141668234	-2.384113515071
H	7.073985154906	-2.936280340407	0.333559340040
H	5.495600099489	-2.368288609129	2.159588791202
H	3.556832294425	-0.884754225084	1.710361248066
C	-0.334046744345	0.445510242074	-1.286743820844
C	-0.017625286899	-0.811738785834	-0.953616628021
C	-0.076191780558	-1.836657891012	0.075640080825
C	0.251656722881	-2.177271640026	-1.377749983662
C	1.620088501942	-2.713191219258	-1.708417157578
C	1.682000666188	-4.227456402688	-1.614689031918
H	1.920755870750	-2.383244367308	-2.713770423750
H	2.344441928460	-2.279809467488	-1.010419563929
C	3.088537696830	-4.764406482428	-1.818346734226
H	1.311529505029	-4.544204349052	-0.627171190884
H	1.001907119717	-4.676042844584	-2.354934753008
C	3.149153700316	-6.278790030410	-1.759527508409
H	2.786161922299	-6.649169248531	-0.792986290934
H	4.168849162596	-6.653212557185	-1.895671741593
H	2.522971289894	-6.729361689943	-2.539185116199
H	3.749104895872	-4.332755240669	-1.051455978924
H	3.476493451433	-4.409075752803	-2.784488893575
H	-1.044952477787	-2.072189983366	0.508600748519
H	0.767647728841	-1.899547798837	0.762776928889
H	-0.562917293918	-2.601460022940	-1.969491409735
H	-0.831386180111	1.100112308966	-0.566838999017
H	-0.312164361280	0.767219380126	-2.330493561436

TS1 – 0py
Electronic energy -2369.307860 a.u.
Gibbs free energy -2369.085860 a.u.
37

Ti	1.793566040971	1.103648390806	-1.450052008788
Cl	2.552038090759	3.113235017861	-0.747822524021
Cl	1.398939026870	0.801456148629	-3.673005821388
N	2.812818891953	-0.153818841232	-0.844626660526
C	3.824235445935	-0.696970372550	-0.071240978463
C	4.763837025603	-1.564065985345	-0.639785042320
C	5.790249137650	-2.079417466925	0.136705899196
C	3.928359076591	-0.363010463174	1.285451449746
C	4.949253299176	-0.893043728134	2.055100519013
C	5.882672267359	-1.751912865183	1.483846471328
H	6.521903915311	-2.743663564858	-0.313324425314
H	4.688354735649	-1.808665815764	-1.695484926133
H	6.684585169479	-2.163041277296	2.089298883030
H	5.022406727080	-0.631721074768	3.106518870782
H	3.192627842511	0.313331947688	1.714954508952
C	0.156709926839	0.377519896147	-0.333006782417
C	0.947126120146	-0.793347637310	-0.406454497945
C	1.152161350847	-2.008084068737	0.390041978075
C	0.677120750789	-2.096475261597	-1.053799327193
C	1.556283732558	-2.727218585972	-2.102244857033
C	1.744817423553	-4.223494196110	-1.920587453016
H	1.114273446008	-2.528308421686	-3.088775460949
H	2.533840909411	-2.233248225762	-2.107194302619
C	2.613067024043	-4.831524964328	-3.009332366597
H	2.204954773497	-4.416536319402	-0.938709638841
H	0.765882888983	-4.727047298640	-1.904197101297
C	2.818666579747	-6.323789176650	-2.833626817366
H	3.300238172255	-6.545173289008	-1.873381917149
H	3.447564881803	-6.745402088221	-3.624559122526
H	1.861045528999	-6.858195145096	-2.851398786262
H	3.586422254380	-4.318415359766	-3.017533079939
H	2.157777762593	-4.629192665168	-3.989509992611
H	0.453932267803	-2.170034305633	1.206401815681
H	2.162463765445	-2.366123913027	0.573276763147
H	-0.391822801928	-2.266680142778	-1.196473928732
H	0.090385049352	0.906484038563	0.620851684598
H	-0.713501500020	0.440197080428	-0.988612024100

IM3 – 0py
Electronic energy -2369.322975 a.u.
Gibbs free energy -2369.097514 a.u.

Ti	1.276851030351	1.395649961524	-0.831923266204
Cl	1.047143760399	1.853167374860	1.361818183599
Cl	2.575935227715	2.798412477171	-2.098600740207
N	2.071711319176	-0.242958951244	-0.837490962364
C	3.334221049530	-0.784646929890	-0.523446740310
C	4.303592295218	-0.863832184824	-1.521376473463
C	5.547296774593	-1.404939987565	-1.235682368625
C	3.630875681680	-1.208390563277	0.771307876848
C	4.876690597203	-1.753955161270	1.047008138257
C	5.833792437102	-1.860741843774	0.045695579756
H	6.296402340974	-1.473617860065	-2.018905996718
H	4.057394648819	-0.516896617631	-2.521398457253
H	6.807321038889	-2.287574395474	0.266769712771
H	5.103503400843	-2.089305281374	2.054710475383
H	2.883871653787	-1.105150913902	1.553167835456
C	-0.232329920365	0.091979898713	-1.314717301825
C	0.787449355190	-0.961515346098	-0.918484227868
C	0.412806772436	-2.055305105181	0.026650940994
C	0.641108652016	-2.366288477284	-1.438352372891
C	1.775924274134	-3.271498465855	-1.835323207777
C	1.366301901383	-4.736049864001	-1.788492330601
H	2.125694100627	-3.019322245743	-2.847853405543
H	2.634870012428	-3.125007444376	-1.168107687181
C	2.495960646530	-5.678600667253	-2.167496520510
H	1.012642317151	-4.979028753760	-0.774140150980
H	0.508127678237	-4.903007340819	-2.457783680570
C	2.089495652666	-7.138203230746	-2.093260561137
H	1.771232595944	-7.406964527585	-1.078581520321
H	2.911126482883	-7.806798588988	-2.370339785716
H	1.249403364155	-7.348399126288	-2.766432323522
H	3.354146488662	-5.495060730316	-1.504516905002
H	2.841912954345	-5.437415717750	-3.183054883162
H	-0.597236976356	-2.051010578628	0.427103993233
H	1.177460501691	-2.430767248541	0.702916860779
H	-0.262622344546	-2.500994031170	-2.034786509463
H	-1.128336222389	0.123592526338	-0.691868972477
H	-0.459594543102	0.093825942066	-2.385610245386

TS2 – 0py
Electronic energy -2369.301236 a.u.
Gibbs free energy -2369.077466 a.u.
37

Ti	0.723887609977	-1.382298020963	0.153202553976
Cl	0.481960198406	-2.599918614109	-1.811439260417
Cl	2.840938251134	-2.014109167263	0.841461739396

N	0.388650915630	0.476262083692	-0.039264164910
C	1.174193202452	1.618556678777	-0.190449549700
C	1.005465653016	2.746661926267	0.616448019841
C	1.807037801416	3.861626244827	0.418795138488
C	2.143382724378	1.631057973227	-1.199277996955
C	2.937250721447	2.748880898004	-1.383473204764
C	2.775566992580	3.870422316021	-0.576045950480
H	1.669165926456	4.733638414450	1.051451245324
H	0.246236163708	2.744641368743	1.394189406730
H	3.399168332022	4.746148001125	-0.725188977203
H	3.689728545620	2.745553038483	-2.166495137775
H	2.259300221423	0.753017900688	-1.831653687921
C	0.362342961447	-0.343935834844	2.144537907452
C	-0.413213861557	0.219767477104	1.112845457759
C	-1.487683530746	-1.182149026100	0.421743744930
C	-1.882092075471	0.166235386468	0.993655698197
C	-2.555069173456	1.148865161640	0.058515564096
C	-4.072609472241	1.041100255839	0.057789525774
H	-2.269020685058	2.164972559225	0.360444054407
H	-2.164114500522	1.007135012864	-0.958680118660
C	-4.623245542779	-0.286937018226	-0.438289376014
H	-4.448717043331	1.228937490111	1.075498476546
H	-4.479469776837	1.848540039799	-0.567210525890
C	-6.139454143965	-0.286438144918	-0.496642115284
H	-6.571293779356	-0.088423056017	0.492100637121
H	-6.536476630574	-1.245002379645	-0.845865682864
H	-6.506283864499	0.492750625764	-1.175830513043
H	-4.279556710501	-1.101742446978	0.214297928014
H	-4.210493670500	-0.500877479857	-1.435073544353
H	-1.594500878891	-2.026690052999	1.111901697676
H	-1.784948160186	-1.338708662701	-0.616088425336
H	-2.360899045693	0.114115351879	1.974704318037
H	-0.124544061203	-0.772485179298	3.018038507053
H	1.360140405988	0.065050129133	2.282023173627

IM4 – 0py
Electronic energy -2369.341905 a.u.
Gibbs free energy -2369.115967 a.u.
37

Ti	1.420830943033	-1.079014369398	1.218790473195
Cl	2.723115217851	-2.358691760003	2.601425989123
Cl	0.279386697575	0.928152486923	1.046407254655
N	2.448468397332	-0.956644918815	-0.354911196541
C	3.758176355953	-0.420650512115	-0.361752431534
C	4.786788682445	-1.108851153501	-1.012180947677
C	6.078567098041	-0.616167037319	-0.974433448486
C	4.045507204885	0.757007721130	0.326892034736
C	5.349145488691	1.236298964700	0.371818401627
C	6.365831606116	0.556470513315	-0.280375951043
H	6.872240032669	-1.153488777775	-1.485042298090
H	4.556965579094	-2.028711634562	-1.543023186546
H	7.382226579127	0.936883629445	-0.252462776992
H	5.563752662458	2.153984487693	0.911012270248
H	3.241476561960	1.305615767846	0.811763746183
C	1.998266843513	-0.635731559838	-2.714873397931
C	1.784730607268	-1.267558909935	-1.560160894579
C	0.276614015395	-2.347721356008	0.067690178124
C	0.814436813666	-2.406942218853	-1.363744061699
C	1.535328344260	-3.726890791634	-1.652858043730
C	0.616430889771	-4.934296140330	-1.712779616000
H	2.053121636985	-3.628601533774	-2.618390050692
H	2.319454688744	-3.881263678604	-0.892639842881
C	1.335784176363	-6.195580824969	-2.162470605775
H	0.149672176183	-5.113086926644	-0.732791933286
H	-0.210365275454	-4.722648265530	-2.409248950882
C	0.413696764743	-7.397318995162	-2.242980195175
H	-0.028843335458	-7.622649710095	-1.265165731841
H	0.940380899910	-8.294880978265	-2.583292544294
H	-0.411607729406	-7.212672117233	-2.941632297395
H	2.166718990688	-6.404701313794	-1.473395535596
H	1.795799656004	-6.013731713045	-3.144562781902
H	-0.732811996826	-1.917949954708	0.142548507882
H	0.280329287769	-3.316571911829	0.585984310139
H	-0.004176237656	-2.301687837503	-2.089976599420
H	1.438553356715	-0.926990229500	-3.598307964996
H	2.718160319591	0.170652559689	-2.811779880932

TS3 – 0py
Electronic energy -2369.292315 a.u.
Gibbs free energy -2369.073137 a.u.
37

Ti	-1.000383813753	0.389316825915	0.341769749896
Cl	-1.230770524387	0.629342445219	2.610987873047
Cl	-2.460410839225	1.943533504196	-0.530245949607
N	0.692434903999	0.715468195149	-0.447256809237
C	1.723139382534	1.632754749704	-0.183459417274
C	3.065019551920	1.244130450185	-0.166328542306
C	4.045739145828	2.162329324385	0.172850472757
C	1.384900558561	2.945834580543	0.149248421528
C	2.373542686016	3.854143660461	0.494218452920
C	3.707775565109	3.469406370026	0.503713787234
H	5.085701124905	1.849517584325	0.186535915089
H	3.332815718874	0.219828584116	-0.407559825021
H	4.481314061619	4.182958607115	0.769791628952
H	2.097066109692	4.871991836485	0.752255062144
H	0.340338751752	3.247362953974	0.129143497201
C	1.543525952815	-0.446436329668	-2.434444870032
C	0.795927682480	-0.376969777404	-1.330351165517
C	-0.085854958644	-1.696568091209	0.502696710172
C	-0.108731208975	-1.481139339341	-0.869483901187
C	-0.654829472323	-2.444471509631	-1.878004521049
C	-1.800321380173	-3.317886816912	-1.403826698867
H	-0.948745516124	-1.892520361742	-2.779897699434
H	0.192784581492	-3.085540586241	-2.170176049329
C	-2.348108060300	-4.191200053867	-2.520650370061
H	-1.474280570326	-3.957331895275	-0.571617385687
H	-2.606384688714	-2.678890693002	-1.009956295652
C	-3.486914561671	-5.078232531900	-2.056086922356
H	-3.161310614783	-5.751345021325	-1.253886841564
H	-3.878512281192	-5.697043397311	-2.869691912475
H	-4.318157222762	-4.478978424202	-1.665410783153
H	-1.534778799297	-4.808136626814	-2.928873163066
H	-2.686487396162	-3.549256782300	-3.346151602750
H	-0.685594466363	-2.487825016855	0.943688146128
H	0.746120909670	-1.315951889097	1.093392130175
H	-1.587401205118	-0.519618090651	-1.000642910610
H	1.537410432990	-1.337884549053	-3.050453429672
H	2.195436460036	0.368629112004	-2.728849781340

IM5 – 0py
Electronic energy -2369.306252 a.u.
Gibbs free energy -2369.085999 a.u.
37

Ti	1.678341126322	-1.681286342633	0.926674119454
Cl	2.435303610952	-1.354876488451	2.994810522666
Cl	0.208020716834	-0.071117355299	0.441370372498
N	3.129229440707	-1.443837353459	-0.309371629545
C	3.934929593111	-0.271631196516	-0.295485748263
C	5.150957612049	-0.285618960586	0.383773588890
C	5.942015275754	0.853004910620	0.406076562863
C	3.515596953088	0.881381808750	-0.953050213727
C	4.311565981655	2.017892178016	-0.926753101727
C	5.522906467844	2.006021993909	-0.247211572513
H	6.887876976689	0.841939091615	0.939222795411
H	5.460897948642	-1.194959918966	0.892863586612
H	6.141422743884	2.898159006176	-0.224515077917
H	3.981950582813	2.917654622056	-1.437463891530
H	2.567007483051	0.875720387485	-1.483468982013
C	4.030754889256	-2.268480646704	-2.424975116180
C	3.276829665451	-2.377373824743	-1.325051950152
C	2.423786625643	-4.043795943543	0.229186410807
C	2.401933552587	-3.541480746210	-1.022476282086
C	1.451484434015	-3.978564790628	-2.084517612409
C	0.549453036549	-5.151058173231	-1.757172155383
H	0.844091069730	-3.097788312099	-2.356379664455
H	2.038514582769	-4.191231814766	-2.991393583563
C	-0.380055750263	-5.495114646915	-2.909263391489
H	1.158457144085	-6.031030049154	-1.502682104885
H	-0.051421554268	-4.922481016410	-0.864452852806
C	-1.288161701373	-6.666784132282	-2.589157641436
H	-0.704645031462	-7.567447299712	-2.363091216029
H	-1.955867683116	-6.904950810615	-3.423263146420
H	-1.913330924864	-6.452374801163	-1.714017835549
H	0.219032569572	-5.718659726149	-3.803612431478
H	-0.983860192093	-4.611660984322	-3.161326038453
H	1.785458757189	-4.868174348481	0.524363868533
H	3.210992927230	-3.763347382860	0.931555424987
H	0.437670683394	-2.817310073824	1.111827165310
H	4.046228236269	-3.065605617493	-3.159554742040

H	4.656781150305	-1.400382241416	-2.602902435983

TS4 – 0py
Electronic energy -2369.304172 a.u.
Gibbs free energy -2369.083491 a.u.
37

Ti	0.890270469865	-0.802349483792	-0.172142149751
Cl	0.538576418754	-0.775223747259	2.047445595277
Cl	0.193747815778	1.161272619319	-0.970207450646
N	2.716866251160	-0.819009539737	-0.584277868904
C	3.903281523263	-0.136806176202	-0.313337191839
C	5.138078032268	-0.591626211911	-0.786997550476
C	6.298066678970	0.099502452401	-0.475514780999
C	3.859846262699	1.015589176170	0.478797344771
C	5.027036454692	1.695256541590	0.784883325635
C	6.252608410471	1.245277047069	0.308821661898
H	7.249633304895	-0.265029504027	-0.851638230984
H	5.183803058418	-1.486616889776	-1.400418267342
H	7.164606890614	1.784014867633	0.545857951671
H	4.976268412816	2.589743324430	1.398601870139
H	2.903629675523	1.376301836590	0.848645746398
C	2.723116046799	-1.511915879178	-2.887527503243
C	2.589995474852	-1.820770969555	-1.588064053847
C	2.201653752505	-3.402263185664	0.226078780493
C	2.102920918656	-3.126378923822	-1.086269921408
C	1.488638040782	-4.063055506285	-2.083051689836
C	0.745903529026	-5.255519622906	-1.511063602369
H	0.806314263704	-3.487945914530	-2.725332575285
H	2.288244335550	-4.416611631451	-2.753086825493
C	0.091622461863	-6.095629782302	-2.595378733398
H	1.429879530594	-5.889266488963	-0.928387607111
H	-0.025764562237	-4.904692505497	-0.808525376835
C	-0.637422193651	-7.300351578866	-2.031862907904
H	0.051249869713	-7.960085345536	-1.490280441267
H	-1.114915606515	-7.893430634416	-2.818403491097
H	-1.419401027334	-6.992710956766	-1.327104218551
H	0.858369105027	-6.423823213381	-3.311897983970
H	-0.607923552097	-5.467622419443	-3.165167542726
H	1.814038138270	-4.326655560603	0.639849734612
H	2.732716535402	-2.749658849817	0.911521839147
H	-0.174462213956	-1.736615860489	-1.100876923658
H	2.605236176429	-2.253329196585	-3.669774065631
H	3.005771316435	-0.505623286439	-3.183103895469

IM6 – 0py
Electronic energy -2369.317443 a.u.
Gibbs free energy -2369.096892 a.u.
37

Ti	0.753939954452	0.718983279644	-0.982645596071
Cl	-0.007787837195	0.810200268607	1.135284236946
Cl	1.336368680324	2.805293260117	-1.504941308219
N	2.394832260079	-0.265033271643	-1.075554439545
C	3.666026763449	-0.332682526919	-0.497567158002
C	4.617677242697	-1.236207655361	-0.977683186182
C	5.886268218587	-1.265118875741	-0.422094873087
C	4.008952515017	0.543707916570	0.533209885159
C	5.282645695932	0.508944406983	1.078277504820
C	6.225973946244	-0.395832765913	0.607802590745
H	6.618798620850	-1.970821757146	-0.802736368241
H	4.358431365995	-1.905491228652	-1.794123885284
H	7.222071691752	-0.421276369372	1.038345249296
H	5.537667863675	1.194217654520	1.881029721355
H	3.271029075770	1.248502976030	0.907548817662
C	0.980932535024	-0.879037722954	-2.781455696146
C	1.726956309683	-1.292705674529	-1.706997347025
C	1.680749668507	-2.753739651524	0.249497476664
C	1.567073888784	-2.626696615463	-1.072489411154
C	1.290825994233	-3.766596486869	-2.011074144138
C	1.247383156780	-5.148036956284	-1.388228192892
H	0.337828661734	-3.578728246935	-2.529021346300
H	2.052042815905	-3.745230313837	-2.807619255165
C	0.997194471756	-6.237061294295	-2.418545930568
H	2.193951608190	-5.352306977215	-0.865866973386
H	0.459568198185	-5.188955132033	-0.621553699465
C	0.944527161091	-7.620024223856	-1.797862857717
H	1.885651472991	-7.858987480325	-1.287895042393
H	0.767085945998	-8.397932210450	-2.547441682966
H	0.142016257178	-7.687271405337	-1.053352995329
H	1.786572449234	-6.200147437424	-3.182944938074
H	0.054936800628	-6.027702314384	-2.945067254275
H	-0.591901362263	0.481195255614	-1.977469011917
H	0.204920825132	-1.494367659178	-3.220655488761
H	1.300680612864	-0.012700551670	-3.359192366504
H	1.572055988724	-3.713087978196	0.743803005328
H	1.880150482014	-1.900784234584	0.891601960831

TS5 – 0py
Electronic energy -2369.314238 a.u.
Gibbs free energy -2369.094984 a.u.
37

Ti	0.741774310765	0.478441914154	-1.002707637516
Cl	-0.295532737989	0.291352671504	0.999429725438
Cl	1.135132528805	2.642764150914	-1.396667724258
N	2.465188156416	-0.299438996473	-1.018236434787
C	3.733014162426	-0.278361758451	-0.440001038950
C	4.762160578195	-1.075173415872	-0.947427026106
C	6.030350826343	-0.998306198234	-0.395620130258
C	3.991018545973	0.590255021856	0.621758483772
C	5.264809037247	0.661100355298	1.162986971142
C	6.289519802470	-0.131555961728	0.659680536351
H	6.826011274710	-1.617775369870	-0.799244236549
H	4.559576225140	-1.740882887273	-1.782078991319
H	7.286240798158	-0.072598253951	1.085589080743
H	5.457840643634	1.340481620608	1.987841696837
H	3.183342949049	1.201544661728	1.017177268478
C	1.134284576755	-0.914308866385	-2.816142901018
C	1.849827400341	-1.337682361204	-1.694665047521
C	1.923899749113	-2.847434224528	0.227631159906
C	1.665291656875	-2.671672215197	-1.070260942528
C	1.202682554609	-3.773395421498	-1.983188343922
C	1.187152019590	-5.171084269623	-1.394660578476
H	0.190414608137	-3.531950571532	-2.343400649517
H	1.833258020897	-3.765029710769	-2.885917542270
C	0.716056231815	-6.211670310476	-2.397464497014
H	2.193592404777	-5.437107270547	-1.038319090528
H	0.531788236138	-5.201441254405	-0.511930815256
C	0.709880829503	-7.613468876223	-1.818173742468
H	1.715364718978	-7.911290048555	-1.497000525805
H	0.360943546229	-8.354349604691	-2.544722676811
H	0.054088288875	-7.674323574323	-0.941174331614
H	1.362101940785	-6.177687620319	-3.286468660240
H	-0.292879360406	-5.946860315376	-2.745279218994
H	1.828322612379	-3.817265931572	0.703578894484
H	2.237718270008	-2.027503641598	0.866395311866
H	-0.196289488325	0.128428191502	-2.387572246004
H	0.458493391618	-1.583406485529	-3.337054655542
H	1.557618689967	-0.114823171361	-3.422344443746

IM7 – 0py
Electronic energy -2369.350678 a.u.
Gibbs free energy -2369.125347 a.u.
37

Ti	1.288758891871	-1.855522988739	0.579768183894
Cl	1.140865193351	-0.642987231403	2.477646853374
Cl	-0.413269737883	-1.252806382485	-0.817198395965
N	2.955562212164	-1.348979540956	-0.103862779280
C	3.918263898516	-0.337119924209	-0.158652749232
C	5.232561741495	-0.624560722578	-0.534259014535
C	6.184068856928	0.383083550654	-0.545378061531
C	3.571176330080	0.966677187709	0.198471324976
C	4.532370323567	1.964282452713	0.191603561358
C	5.840832268354	1.679068910795	-0.180573239479
H	7.204813622090	0.150825362297	-0.834499858979
H	5.503636093434	-1.643461599346	-0.797352223149
H	6.590209486632	2.464476465964	-0.186957768639
H	4.254772939609	2.975640739107	0.473022745476
H	2.542325382089	1.186604396200	0.469982493646
C	3.094276343729	-2.207060370729	-2.440977307049
C	2.883148261687	-2.435410428735	-0.973519871293
C	2.089260728047	-3.745043043551	0.952965076139
C	2.415958944255	-3.637443236406	-0.447423593326
C	1.956921569555	-4.738753162292	-1.368369511611
C	0.478245389801	-4.675919711927	-1.737441170799
H	2.557226655870	-4.731172143902	-2.286281516728
H	2.159451611144	-5.697526347210	-0.872897236307
C	0.048280509373	-5.821940948813	-2.636505717661
H	-0.131988390558	-4.681095099025	-0.820923882174
H	0.262651248444	-3.721352500777	-2.238628081667
C	-1.421202846038	-5.741808008230	-3.004929315433
H	-2.054134480742	-5.775874058798	-2.109907673694

H	-1.725018934453	-6.566463723386	-3.657711282449
H	-1.644901992658	-4.804069222616	-3.527844760589
H	0.259264511231	-6.777209232946	-2.134919517316
H	0.663805947369	-5.814107003949	-3.547682392213
H	1.620620409066	-4.667371175029	1.286918647070
H	2.820355597159	-3.334429796639	1.663070447783
H	3.967667371469	-2.755241292887	-2.811361915033
H	2.227535112441	-2.537480769059	-3.022229218571
H	3.247798931515	-1.145069398815	-2.646031279016

IM5-D
Electronic energy -4673.831012 a.u.
Gibbs free energy -4673.445730 a.u.
63

Ti	2.631522046014	-0.688424691487	2.400110043788
Cl	4.208111347228	-2.272317572023	2.956799980539
Cl	1.129842053610	-0.993378731511	4.119687027504
N	3.464457515829	-0.666951570485	0.337752110670
C	4.769109775735	-1.177241553737	0.042779763216
C	4.902425096884	-2.348799195230	-0.700429625748
C	6.157251967703	-2.900940464101	-0.903314923130
C	5.900503954386	-0.568163786699	0.575443189162
C	7.152194144182	-1.127340575829	0.368282951337
C	7.287748056055	-2.294445271376	-0.370916754519
H	6.248418764799	-3.819601334685	-1.475048381063
H	4.018389057286	-2.840541615133	-1.098417667006
H	8.268583959394	-2.731865314697	-0.528316776511
H	8.027521454278	-0.644734563198	0.792105060748
H	5.803928393257	0.335878313845	1.168022423956
C	2.586114504558	-0.222003106129	-1.828416457971
C	2.448225186386	-0.851573787436	-0.640476221883
C	1.256419207711	-1.856736133700	1.278519971606
C	1.172360825303	-1.511737354143	-0.198631465731
C	0.857230267932	-2.711893719814	-1.101504077156
C	-0.495510791500	-3.336761294960	-0.811200883625
H	0.885409684116	-2.392480032197	-2.153942855169
H	1.651532882526	-3.465588532983	-0.980740012434
C	-0.849933118902	-4.451939916545	-1.780678716210
H	-0.516648391634	-3.729879609556	0.215177621061
H	-1.270891810960	-2.555092753700	-0.853965098329
C	-2.207790447703	-5.063295969565	-1.490832031559
H	-2.242203783329	-5.485268074107	-0.479150776264
H	-2.453997942614	-5.865899773972	-2.193631808906
H	-3.000983599134	-4.308657211078	-1.557879381982
H	-0.072769331960	-5.228634453664	-1.737292724956
H	-0.829262235422	-4.059313316564	-2.807645445853
H	0.363574985724	-0.775975651743	-0.344697121136
H	1.775567960451	-0.203433475797	-2.549815227060
H	3.542539919838	0.205416555830	-2.142517316095
N	3.823887424611	0.592547778795	4.082830785023
C	3.824619924004	0.279744661064	5.383352333811
C	4.667805489157	1.546411452369	3.674947022341
C	5.530322383189	2.214460224848	4.525823310083
C	5.519882583315	1.885790857128	5.871795134993
C	4.648120452007	0.899649801497	6.306152693524
H	4.642983568153	1.795717057707	2.618918162949
H	6.179942223392	2.389057842473	6.571137424504
H	6.190328411603	2.978430726442	4.130017244612
H	4.601467741502	0.605206723701	7.348534288129
H	3.135218632569	-0.499723019137	5.693835535246
Ti	2.900504901947	1.318847208820	0.080377330211
H	1.730175251550	-2.846526742366	1.411991621615
H	0.262524924187	-1.914258890765	1.725889052261
Cl	4.649498013008	2.749695331210	0.249494875324
Cl	1.358544852549	2.554636163020	-1.029245755305
N	2.092693471358	1.129458482592	1.690561226785
C	1.197795617666	2.012262863138	2.331801390652
C	-0.172990303339	1.745543300587	2.301582666039
C	-1.063393645723	2.620891205289	2.900086006104
C	-0.602258011443	3.768176458079	3.535934591813
C	0.759309193826	4.036637522656	3.562570562229
C	1.659507941585	3.168497407911	2.961445134452
H	-0.525092439819	0.847361759241	1.801291795315
H	-2.127342421595	2.405248833767	2.869488391981
H	-1.303160035553	4.451573018422	4.005226327157
H	1.129139222655	4.933398044218	4.051018399243
H	2.724033075609	3.383369465461	2.974264055619

TS3-D
Electronic energy -4673.779586 a.u.
Gibbs free energy -4673.398697 a.u.
63

Ti	2.769154675403	-0.990308927261	1.856560943835
Cl	2.288949268907	-2.921910971940	3.082155786115
Cl	0.200246185352	-0.901885054314	1.445264571356
N	4.380697067608	-1.590213677637	1.015600256897
C	5.557850953967	-1.069535710831	1.624867211824
C	6.116286082215	-1.710720122254	2.729364845389
C	7.273663497677	-1.205037198984	3.302487020812
C	6.167691597818	0.060284608231	1.084677704205
C	7.320661640646	0.565773253141	1.666148389243
C	7.872682768740	-0.065850613003	2.776037323127
H	7.709696317947	-1.700313686182	4.164634915333
H	5.633865493978	-2.600228498271	3.125420843680
H	8.776551442494	0.329452989748	3.229494059946
H	7.790172264283	1.453334990406	1.252275936384
H	5.718702086648	0.523444739200	0.208955637631
C	5.768444304852	-2.759276099761	-0.599150860510
C	4.571160927711	-2.396835161372	-0.117195491690
C	2.532739311012	-3.728684123072	-0.246312276697
C	3.320076534260	-2.818568435800	-0.806984709789
C	3.074615316309	-2.179740296362	-2.143086210358
C	1.665813528064	-2.296644312737	-2.693587037365
H	3.356680568990	-1.118081884865	-2.075230916966
H	3.790260915309	-2.616821463555	-2.857667289767
C	1.474166654264	-1.499593796354	-3.973827319243
H	1.418425243071	-3.351935193238	-2.884449643550
H	0.945865822328	-1.954409031557	-1.932253641763
C	0.116326492999	-1.731532526972	-4.604148384294
H	0.079051624086	-2.697566025064	-5.121501358186
H	-0.131795747087	-0.952397440870	-5.332275635594
H	-0.673019716232	-1.736498774709	-3.841465852776
H	2.263891037773	-1.775242111598	-4.688109174299
H	1.614757800560	-0.429823693323	-3.763690392050
H	2.355690024094	-0.698327070117	0.140048193806
H	5.815956870362	-3.388217703187	-1.481892980427
H	6.707175737556	-2.473110682833	-0.138245967056
N	3.216328890214	-0.109829267391	3.875472895723
C	2.292237179311	-0.204747864142	4.848776482519
C	4.293788509388	0.656016325008	4.113056917195
C	4.512522674939	1.294063566526	5.318400954641
C	3.563895070130	1.176156811648	6.320621223650
C	2.427936954981	0.422160182780	6.072045638305
H	5.010033645913	0.753616567816	3.306021142127
H	3.702687211756	1.669087589054	7.277317627010
H	5.413993438433	1.882231454659	5.449743863554
H	1.649607217232	0.304158587826	6.816910497613
H	1.420837977577	-0.816590404426	4.639864160496
Ti	1.347044281552	0.837050737461	0.177395899753
H	2.785035369704	-4.199258975383	0.698165559521
H	1.611419230995	-4.053880878060	-0.722390621966
Cl	2.461796237804	1.572917429318	-1.672364205754
Cl	-0.235948885292	2.460964273887	0.411377431153
N	2.498059304792	1.071265297536	1.503670611572
C	2.906380098682	2.276541591923	2.095086134403
C	2.155947973012	2.839642294318	3.132120430598
C	2.565887270986	4.020876044158	3.727283058155
C	3.721204660034	4.662471517720	3.293459223053
C	4.458852527351	4.117117124206	2.250770950831
C	4.055653086581	2.934167636429	1.648925444319
H	1.251409358724	2.332576285641	3.460055854975
H	1.976718463748	4.446733344621	4.534434324455
H	4.038705970001	5.589955567163	3.760088216281
H	5.354485274045	4.618637909995	1.895640749409
H	4.630206113446	2.507828957005	0.832981039205

IM6-D
Electronic energy -4673.791693 a.u.
Gibbs free energy -4673.408536 a.u.
63

Ti	3.094424876126	-0.975161018055	2.134971173819
Cl	4.851690679631	-2.362675565121	3.003549695156
Cl	1.427909778542	-1.676815213371	3.521924897122
N	3.982423016456	-1.011091769676	0.170481504182
C	5.398678470555	-1.175865520056	0.084867147449
C	5.970682975396	-2.336056135926	-0.428602292113
C	7.347041063779	-2.500000407386	-0.394167925840
C	6.218732667763	-0.184311439314	0.617222460031
C	7.592473656174	-0.355866903243	0.653698664108
C	8.163688681191	-1.516933974412	0.149739424783

H	7.783766422658	-3.411916629169	-0.790307235831
H	5.338128048078	-3.119988764052	-0.834858968806
H	9.240092052052	-1.653832115653	0.177252539132
H	8.218808462980	0.424690334453	1.074449449513
H	5.774593825836	0.730891486315	1.003491710516
C	3.275360114754	-1.645656225450	-2.085644345584
C	3.132215593459	-1.689760269023	-0.753458675975
C	2.271312397196	-3.175168379822	0.944034463023
C	1.995496772700	-2.395204291148	-0.115862257411
C	0.639357607920	-2.252118990960	-0.732149458157
C	-0.547589507795	-2.566269662003	0.159700653087
H	0.540474635123	-1.235924124476	-1.136454926920
H	0.619187437436	-2.909754296826	-1.616743701372
C	-1.864883686813	-2.293556695435	-0.547916670190
H	-0.521011250432	-3.615232707158	0.486027698765
H	-0.491492352011	-1.955258658642	1.073263245417
C	-3.063744445450	-2.532323836713	0.348928130492
H	-3.089172442687	-3.568216645439	0.708030801727
H	-4.007021138545	-2.338066853339	-0.171675585361
H	-3.028414857157	-1.879559230207	1.229871284635
H	-1.932005763567	-2.924035521439	-1.446325890634
H	-1.871251051715	-1.253820120302	-0.907605109494
H	1.736467884770	0.917542961532	-1.385426674823
H	2.537760033332	-2.121668456705	-2.723095368670
H	4.138030037817	-1.200851091169	-2.564599893342
N	3.830952612079	0.420696211791	3.678765055508
C	3.858188407049	0.077890130965	4.977609316703
C	4.261657476444	1.643819318414	3.336561257587
C	4.731428511623	2.556894437459	4.260160718302
C	4.740726673896	2.204379347036	5.599809931981
C	4.293750190941	0.942362769865	5.961111507409
H	4.199770414401	1.909026831237	2.286621206211
H	5.089907854346	2.902956356993	6.353060692906
H	5.066814510674	3.530774264679	3.923557799715
H	4.282028741109	0.619258212673	6.995347431915
H	3.514638245014	-0.919056745709	5.229415800748
Ti	3.060678034120	0.872215198802	-0.358554504596
H	3.298295825889	-3.410590241965	1.214198824482
H	1.497029848623	-3.739023250033	1.451004770800
Cl	4.595879293965	1.328852587758	-2.036251881275
Cl	2.927052401743	3.070257447474	0.202217759685
N	2.085906806509	0.365376895136	1.324892513813
C	0.893683944883	1.010592287225	1.699381983240
C	-0.225033966370	0.966851721379	0.858551170406
C	-1.416431402483	1.571815072637	1.227873723798
C	-1.515595118632	2.252160833580	2.433873073642
C	-0.407959937842	2.312147454006	3.269848936414
C	0.781421370120	1.695206084372	2.915743459587
H	-0.149821052190	0.444016999544	-0.089613492065
H	-2.271164518327	1.515105387070	0.559217265589
H	-2.446391162725	2.731966921678	2.719954687216
H	-0.468832992823	2.841445234160	4.216455736647
H	1.631958292415	1.747288961165	3.587507291197

TS4-D

Electronic energy -4673.785896 a.u.

Gibbs free energy -4673.405479 a.u.

63

Ti	3.075781262363	-1.317526597659	2.168559575954
Cl	5.068932153521	-2.196519860776	2.935707499584
Cl	1.552264979900	-2.389200943894	3.460194729406
N	3.941761757170	-1.211236641404	0.238053505404
C	5.343360201765	-1.188387431356	-0.005736250499
C	5.995363501504	-2.358897158020	-0.408697618238
C	7.375105118345	-2.367836699834	-0.580744585370
C	6.089267070653	-0.025831887798	0.204660774834
C	7.471929354463	-0.045008574125	0.043692545048
C	8.121457188757	-1.214156354614	-0.345939032111
H	7.873815161115	-3.289826034132	-0.893346512443
H	5.409165951585	-3.270050981070	-0.571527921609
H	9.207010454377	-1.224811827351	-0.476014649959
H	8.045274257604	0.871796166900	0.208894624950
H	5.568225412349	0.903488241556	0.459692953106
C	3.237663838923	-1.378559813818	-2.044360015924
C	3.083300436496	-1.774866793675	-0.728925249991
C	2.491940252742	-3.539378061625	0.805372227458
C	2.112578816578	-2.763065555626	-0.238194512680
C	0.762093351769	-2.920667111456	-0.877365065618
C	-0.361401892019	-2.386508455169	0.003890328752
H	0.728087949329	-2.386221271119	-1.841477313780
H	0.588607769033	-3.989504788119	-1.098827856901
C	-1.719564806096	-2.402667268462	-0.671172549376
H	-0.394139714549	-2.948295248508	0.958473717180
H	-0.120118060437	-1.344965257758	0.275669354971
C	-2.770007371057	-1.693921832250	0.161136084054
H	-2.860824666509	-2.146529110535	1.164189846800
H	-3.765546597800	-1.723203275670	-0.307996974088
H	-2.497877510684	-0.633072839799	0.302211063291
H	-2.023913595140	-3.445560395265	-0.883217428269
H	-1.632180887123	-1.906843580306	-1.656702040123
H	2.462549280280	-1.627645624414	-2.776707722621
H	4.219182845527	-1.138322133258	-2.455242174021
H	3.800460856675	0.373547610904	-1.466194185344
N	3.488639357000	0.165529635256	3.751171341942
C	2.657938653072	0.432581814343	4.765767528948
C	4.554989190797	0.953999185941	3.557302329690
C	4.829497180018	2.043871356602	4.369917957302
C	3.973287960337	2.318192965705	5.433607594204
C	2.873770896604	1.494186926125	5.637745279086
H	5.220006780718	0.689114517531	2.727296723825
H	4.164793794018	3.166511892495	6.096382647943
H	5.707946165883	2.660115100252	4.168553509028
H	2.172733900293	1.667406025593	6.456793116731
H	1.796693131193	-0.233466908087	4.877547692222
Ti	2.520697097980	0.413393784396	-0.354528153083
H	3.519057462655	-3.552581560674	1.193754279049
H	1.807581805263	-4.286850941075	1.217547306499
Cl	3.380113380427	2.501486900068	-0.245636449990
Cl	0.619238629144	0.575933122437	-1.531250842485
N	2.033200639284	0.081647609135	1.534554261858
C	1.050402013404	0.900981807388	2.112925244620
C	-0.207311312615	0.373122116932	2.457480306912
C	-1.157812157651	1.167097429329	3.087286879434
C	-0.878205357188	2.498682173961	3.395829097346
C	0.363913565845	3.030214944638	3.057587913654
C	1.318594215695	2.246709475007	2.416227904617
H	-0.415843612887	-0.678694490796	2.247997782310
H	-2.130185343043	0.735376222047	3.344321430991
H	-1.628983162274	3.119499299049	3.892810439540
H	0.595388072548	4.074600685373	3.288167082744
H	2.288372932071	2.667826300536	2.138650623238

IM7-D

Electronic energy -4673.861225 a.u.

Gibbs free energy -4673.474634 a.u.

63

Ti	3.096677280130	-1.287755105822	2.211750681637
Cl	5.135234541564	-2.118634201116	2.920686900544
Cl	1.681877117013	-2.274732754462	3.734718915265
N	3.931987158053	-0.986185160467	0.110927984218
C	5.339725241628	-1.094927356274	-0.090426280557
C	5.869260264044	-2.293429699405	-0.570246150373
C	7.239439225532	-2.447531338722	-0.703322959948
C	6.202970932259	-0.058565975323	0.253913540571
C	7.574457642602	-0.221943446279	0.126042364100
C	8.099589389604	-1.413810432345	-0.353569626472
H	7.638117865595	-3.387549574321	-1.072964051479
H	5.202352287405	-3.116299673913	-0.812154504726
H	9.173380571509	-1.538455488207	-0.453018506312
H	8.236082204233	0.593736122335	0.401882342739
H	5.803123808023	0.878959443465	0.624567574994
C	3.333426778355	-1.450895327494	-2.329697054159
C	3.023191960353	-1.548559827033	-0.857278952237
C	2.436817293509	-3.031402755674	0.961959547800
C	2.125850363068	-2.490548873763	-0.301687900025
C	0.830375351316	-2.826364290802	-0.959791328118
C	-0.360148868849	-2.398529076703	-0.098443340189
H	0.753097722400	-2.339481651579	-1.937255762913
H	0.790003508789	-3.912251084578	-1.125054218965
C	-1.688389411015	-2.536867781940	-0.819444188816
H	-0.381374126256	-2.978377301450	0.835316161504
H	-0.222411813828	-1.348631616612	0.191313149691
C	-2.828400603256	-1.962369349484	0.000219000036
H	-2.897712673558	-2.451063088365	0.980167285279
H	-3.794610242839	-2.081699397080	-0.500281334193
H	-2.674523464697	-0.889730048250	0.176232492163
H	-1.875735392820	-3.595063077798	-1.050626213934
H	-1.629223313614	-2.015997176769	-1.785930902786
H	2.427121101179	-1.586592119974	-2.924314244714
H	4.061054749458	-2.204479472771	-2.651174125096

H	3.752015565967	-0.467644349410	-2.583797127468
N	3.469777242714	0.243306671721	3.882866686135
C	2.744478919486	0.472295714402	4.981412208826
C	4.474618655269	1.082574964068	3.599844307606
C	4.790541973081	2.168640376093	4.392082973304
C	4.037069548768	2.399429421913	5.534216722181
C	2.999161044285	1.533645215447	5.833207854869
H	5.053266427663	0.869892348754	2.703908103131
H	4.259107867638	3.242312969858	6.180708935013
H	5.615513383156	2.814599836467	4.114920229473
H	2.381886596077	1.671251645685	6.713560577277
H	1.933723234349	-0.221162704836	5.181063809640
Ti	2.552856336798	0.442329712501	-0.161261458084
H	3.486117149138	-3.336327810321	1.064751531468
H	1.737854864010	-3.746588341431	1.384057441779
Cl	3.820626262429	2.327061012781	-0.408699640841
Cl	0.694153217672	0.784716430497	-1.466158355672
N	1.990626838920	0.183511387473	1.577412235414
C	0.998997626422	0.956276620515	2.214023623896
C	-0.223884730694	0.388593547402	2.582382664633
C	-1.184217155583	1.159244312028	3.217697181660
C	-0.942683490838	2.499804202898	3.495927451385
C	0.266589657044	3.069926560539	3.121467425911
C	1.232325673971	2.308360521641	2.479887563338
H	-0.408122843856	-0.660682283803	2.372014715145
H	-2.130584422034	0.705972969599	3.498640925655
H	-1.697424102643	3.098046688501	3.996979721022
H	0.463282846477	4.118008301566	3.327282659642
H	2.182671367423	2.751102016429	2.192902739134

IM8-D

Electronic energy -4074.434592 a.u.

Gibbs free energy -4074.315544 a.u.

63

Ti	3.574036630947	0.525563332864	1.251521665924
Cl	5.793425822606	0.839068909937	0.671194660945
Cl	2.779880568095	-1.294702786394	0.126218095551
Ti	2.427872724850	2.584454361844	-0.177289124046
Cl	3.163382831979	4.713793122155	0.147440427537
Cl	0.338066375393	2.347431318844	-1.040570249784
N	2.256979988045	1.830940793941	1.513736284115
C	1.403473795312	2.024734988357	2.606847517867
C	0.320219462425	1.161728708439	2.808493094045
C	-0.517522575355	1.335647920870	3.899129101504
C	-0.291349108009	2.368497494060	4.801305278289
C	0.780591720937	3.230736085885	4.603067459829
C	1.623810024176	3.065145743538	3.515113730647
H	0.148096651977	0.362530344146	2.090972204753
H	-1.355204197044	0.659522640619	4.044209846668
H	-0.949956404892	2.502840445023	5.653844779781
H	0.961986801874	4.042184902658	5.302114566375
H	2.463570323040	3.735533557921	3.352701023205
N	3.564473959675	1.920615217125	-1.915125080912
C	3.135304148193	0.906815793648	-2.687439352558
C	4.692943338350	2.560012028357	-2.269193387953
C	3.815151824273	0.497399819891	-3.815832599479
C	5.426485280138	2.205250694301	-3.383549337770
C	4.984061928472	1.154188870097	-4.170971396659
H	3.427989293464	-0.328321722579	-4.401136383349
H	2.221847362013	0.408985655400	-2.376961948362
H	5.019900012213	3.371886838659	-1.626659314768
H	5.541926482882	0.850618515448	-5.050708927806
H	6.331974933969	2.750856404945	-3.621752633590

II. β-carbon elimination from 1a (Figure 4)

IM3

Electronic energy -2617.518496 a.u.

Gibbs free energy -2617.216286 a.u.

48

Ti	1.072247000000	1.655620000000	-0.969214000000
Cl	1.209594000000	2.068132000000	1.260941000000
Cl	2.483114000000	2.909824000000	-2.270666000000
N	1.840068000000	-0.056823000000	-0.987234000000
C	3.120203000000	-0.545411000000	-0.664599000000
C	4.100241000000	-0.601963000000	-1.655964000000
C	5.358593000000	-1.106016000000	-1.365638000000
C	3.432312000000	-0.955260000000	0.632969000000
C	4.692752000000	-1.462949000000	0.915462000000
C	5.656382000000	-1.547895000000	-0.081861000000
H	6.110654000000	-1.157924000000	-2.147736000000
H	3.847976000000	-0.269088000000	-2.659428000000
H	6.641309000000	-1.945644000000	0.143608000000
H	4.925899000000	-1.787239000000	1.925670000000
H	2.680850000000	-0.871815000000	1.412884000000
C	-0.370779000000	0.220196000000	-1.481697000000
C	0.620319000000	-0.837527000000	-1.085384000000
C	0.242843000000	-1.917722000000	-0.117595000000
C	0.506688000000	-2.259133000000	-1.567144000000
C	1.662475000000	-3.164514000000	-1.901855000000
C	1.290780000000	-4.631659000000	-1.745222000000
H	2.007849000000	-2.978621000000	-2.930291000000
H	2.517648000000	-2.946411000000	-1.248736000000
C	2.440553000000	-5.576799000000	-2.049185000000
H	0.940866000000	-4.804579000000	-0.715237000000
H	0.436938000000	-4.869704000000	-2.398781000000
C	2.066535000000	-7.033488000000	-1.849322000000
H	1.759360000000	-7.221670000000	-0.813228000000
H	2.900428000000	-7.706412000000	-2.074573000000
H	1.227093000000	-7.317380000000	-2.495850000000
H	3.296009000000	-5.319106000000	-1.407593000000
H	2.778949000000	-5.415116000000	-3.082908000000
H	-0.774173000000	-1.918908000000	0.265832000000
H	1.000477000000	-2.261490000000	0.583380000000
H	-0.382586000000	-2.428884000000	-2.177489000000
H	-1.298171000000	0.247305000000	-0.902001000000
H	-0.561136000000	0.252448000000	-2.562015000000
N	-0.559645000000	3.223471000000	-1.205807000000
C	-0.315793000000	4.447573000000	-0.710228000000
C	-1.188806000000	5.505745000000	-0.867036000000
C	-2.367806000000	5.300378000000	-1.567577000000
C	-2.625408000000	4.041520000000	-2.084919000000
C	-1.700016000000	3.034140000000	-1.882474000000
H	0.615712000000	4.582010000000	-0.167377000000
H	-0.940871000000	6.472313000000	-0.444130000000
H	-3.075070000000	6.111391000000	-1.708189000000
H	-3.532149000000	3.831862000000	-2.640553000000
H	-1.875356000000	2.038449000000	-2.274739000000

TS2

Electronic energy -2617.501906 a.u.

Gibbs free energy -2617.199249 a.u.

48

Ti	0.510147880136	-1.768926050188	0.137728198370
Cl	0.170892031176	-2.746134556877	-1.941105143093
Cl	2.811528961652	-1.704388353179	0.318718002951
N	0.200933736573	0.139453345781	0.045899199491
C	0.928005127944	1.311579177010	-0.086254685655
C	0.798853744158	2.381301729573	0.808393672152
C	1.540567203663	3.538535924790	0.622082255684
C	1.802055666570	1.439481025011	-1.174407199253
C	2.536409033136	2.598687853819	-1.347678929050
C	2.413878915440	3.656183774272	-0.451224749657
H	1.431272913207	4.358469552028	1.326466202233
H	0.115248963935	2.298641605263	1.649467317418
H	2.990535093065	4.564939691825	-0.592629373196
H	3.210701597808	2.680342447282	-2.195434399522
H	1.885840093947	0.616140458543	-1.879307712660
C	0.190405643554	-0.780713916344	2.175884569598
C	-0.594820549133	-0.159735200452	1.180480954557
C	-1.759169819379	-1.438554050421	0.386162797100
C	-2.065320936573	-0.126728790576	1.079320766427
C	-2.704921205106	0.982633442493	0.271430769671
C	-4.225749478303	0.926249925434	0.267181730752
H	-2.385993560931	1.948276517805	0.685995477273
H	-2.322507003118	0.946169842777	-0.758286193115
C	-4.822796885671	-0.313080412453	-0.382013515575
H	-4.592924125570	1.001015949600	1.302926780459
H	-4.609213977977	1.816146148638	-0.252166408726
C	-6.338657901931	-0.259958698625	-0.423729093201
H	-6.757675755993	-0.177205224933	0.586788113895
H	-6.767260448177	-1.154259095116	-0.887719362167
H	-6.686621867528	0.609716389136	-0.994428643421
H	-4.500053252790	-1.212031555622	0.161207429297
H	-4.423441127229	-0.415554529658	-1.401679204626
H	-2.054045720616	-2.330443011321	0.938281509864
H	-1.990727213818	-1.436617626088	-0.679043665664
H	-2.533242940115	-0.253223258615	2.060027558124

H	-0.286795887125	-1.211605261225	3.053943644205
H	1.187520003559	-0.371911719115	2.318219552448
N	0.321071777122	-3.736137047856	1.197683083104
C	-0.454825582404	-4.011138531257	2.256944662952
C	1.097142276887	-4.723691083570	0.718373684347
C	-0.486000270825	-5.252284423444	2.862089586259
C	1.126545751384	-5.989635979715	1.269661597622
C	0.319914065380	-6.262758637195	2.362396011717
H	1.774815587144	-6.743646521738	0.838909386354
H	0.319361030769	-7.247782314447	2.817674535074
H	-1.134756030265	-5.413957744541	3.714965446095
H	-1.073511385639	-3.205354051709	2.636087907941
H	1.718985828004	-4.489297154800	-0.140114124854

IM4
Electronic energy -2617.538660 a.u.
Gibbs free energy -2617.234590 a.u.
48

Ti	1.357034913350	-1.045923352902	1.235383335938
Cl	2.829072684355	-1.871711509109	2.874062928897
Cl	-0.458783707289	-0.039333394758	0.155317712070
N	2.590192454262	-0.909860228804	-0.203522042507
C	3.569075500301	0.112323551480	-0.145870619822
C	4.904791809512	-0.189688496875	0.129242627905
C	5.837200816101	0.829946896169	0.223962377595
C	3.181558397577	1.441295672019	-0.313150847309
C	4.121316464714	2.459707948830	-0.206584903879
C	5.448558598908	2.156271571267	0.058397992166
H	6.875331903731	0.591057636844	0.435125163966
H	5.194397922911	-1.227704623426	0.268104158878
H	6.183702032101	2.951510199618	0.137073672612
H	3.812855783773	3.492360446663	-0.340171477749
H	2.142433825900	1.663192094564	-0.543495805975
C	3.392698135043	-1.834248242502	-2.288438556564
C	2.526215257537	-1.819795156457	-1.268003126068
C	0.979549051952	-2.881284215301	0.359857067887
C	1.318358369299	-2.728146304736	-1.128223600989
C	1.463065534845	-4.069157517127	-1.844107617477
C	0.239710383772	-4.961987879260	-1.723343239130
H	1.659249311919	-3.892061556485	-2.911421043511
H	2.346287239613	-4.594831184501	-1.448635856091
C	0.342415858375	-6.224014518242	-2.563650396495
H	0.083221463265	-5.245999469834	-0.672583711289
H	-0.658322491773	-4.396832551507	-2.021233701931
C	-0.859111328744	-7.134992096630	-2.395242651840
H	-0.966966450284	-7.455249164255	-1.351681134948
H	-0.780687389336	-8.036647559464	-3.011523992690
H	-1.785992248600	-6.620305214206	-2.676882852049
H	1.261023112431	-6.764099130197	-2.291313637219
H	0.456708526942	-5.947394353744	-3.621669773358
H	-0.060453003096	-3.187739104892	0.529184091136
H	1.652303665125	-3.605386163569	0.840227750540
H	0.478981310941	-2.190121310090	-1.599150535461
H	3.298592374979	-2.562127929650	-3.085162595394
H	4.205321331920	-1.119222048017	-2.362034597027
N	0.519720523276	0.021120171606	3.021062607677
C	0.995911314632	1.212159792859	3.392906080302
C	0.528166061607	1.870294932233	4.515025576883
C	-0.462980106226	1.266523062499	5.274299610225
C	-0.955872001992	0.029428861586	4.885573353978
C	-0.437081301073	-0.560504409125	3.748640697599
H	1.779267396006	1.641147167592	2.772053764526
H	0.940022429333	2.835386813641	4.786148279505
H	-0.847339522340	1.755611374021	6.163523482513
H	-1.729188115663	-0.475591915396	5.452412073430
H	-0.787615093893	-1.531547592427	3.400942910544

TS3
Electronic energy -2617.493094 a.u.
Gibbs free energy -2617.190595 a.u.
48

Ti	0.297949395718	-1.576477329785	0.216964875268
Cl	-1.144548319668	-2.599045525617	1.751055135177
Cl	-1.333802251236	-0.410921014112	-0.999615526355
N	2.085671496989	-0.953854446480	-0.388968766471
C	2.797279517091	0.255130871734	-0.238196367414
C	4.037717301769	0.273559373825	0.405036948425
C	4.697665978634	1.473199095517	0.618573507548
C	2.241113733475	1.455405480653	-0.674705884089
C	2.903316479114	2.655805285379	-0.453354205831
C	4.131100384794	2.670662852732	0.194630783688
H	5.657014767675	1.475258649944	1.128262859838
H	4.469323564247	-0.663133219679	0.750250096998
H	4.646881871090	3.610382319711	0.367757137711
H	2.455923889904	3.585314581741	-0.793825152185
H	1.280779951735	1.435809739149	-1.183403249700
C	3.729726754179	-1.985301164416	-1.901252847706
C	2.676288607579	-2.010753510828	-1.075019810748
C	1.597210875959	-3.474665241995	0.529328560687
C	1.911130748422	-3.256292392346	-0.786308503897
C	1.682748757207	-4.255749394957	-1.875024527478
C	0.570281512706	-5.258329222761	-1.629019980442
H	1.505403112128	-3.719436330550	-2.815897622393
H	2.637125003745	-4.791009696837	-2.008511027771
C	0.324884929246	-6.148222887127	-2.836039949121
H	0.805557548797	-5.888089097109	-0.759472219737
H	-0.356105083373	-4.717323163697	-1.378635114334
C	-0.782309130527	-7.156039233088	-2.593832067609
H	-0.538462953637	-7.816044379883	-1.752539994395
H	-0.958452056705	-7.787328348078	-3.470680572847
H	-1.725830627411	-6.651889511256	-2.352437641398
H	1.256844341988	-6.669836663606	-3.097838222319
H	0.074845613402	-5.518925288937	-3.701787860506
H	1.085197566092	-4.376352311960	0.845504201799
H	2.092686373521	-2.891238620925	1.309332064440
H	0.274658661781	-2.284216591335	-1.328493155333
H	4.054602607692	-2.889664532201	-2.402833664805
H	4.286295346791	-1.073732433027	-2.089656889030
N	0.398492702451	0.195805209356	1.574210516294
C	1.321226377694	0.039183199827	2.532592388429
C	1.560514725874	0.989749190838	3.504471059891
C	0.808016966815	2.154827219654	3.488931064568
C	-0.161591396697	2.313121890562	2.511879950418
C	-0.337821436012	1.312312180072	1.573960376026
H	1.896281884083	-0.885364656051	2.505171703344
H	2.325275346146	0.816039367251	4.252403209932
H	0.972988077247	2.927115539636	4.233287319139
H	-0.778731653980	3.203004376338	2.467544833702
H	-1.082477864534	1.397939784729	0.788832230590

IM3’
Electronic energy -2617.519348 a.u.
Gibbs free energy -2617.216355 a.u.
48

Ti	1.877300025373	1.600520967742	-0.617328316487
Cl	2.285231093389	2.110063940613	1.553697097086
Cl	3.358497507088	2.544879687476	-2.099835850264
N	2.362904578945	-0.216802991954	-0.603304493463
C	3.570019130235	-0.874276448229	-0.303518727826
C	4.559067811203	-0.970305975867	-1.283369026951
C	5.748575796593	-1.629455756271	-1.014194461849
C	3.809389539284	-1.408217164013	0.964836086113
C	5.000288840931	-2.071185992771	1.225413594984
C	5.970093416190	-2.191027919570	0.237604500905
H	6.506091039735	-1.708605265189	-1.788737344942
H	4.367028841721	-0.541474543551	-2.263340676087
H	6.900687685143	-2.710190228457	0.446359458564
H	5.175301876443	-2.489685298389	2.212480920841
H	3.058101989633	-1.294696188655	1.741011577795
C	0.183575248697	0.391036520629	-0.900068971434
C	1.045505145323	-0.817924322966	-0.669350525581
C	0.587340835605	-1.958445949481	0.189237169001
C	0.725812837163	-2.141965409490	-1.305159741482
C	1.750343168302	-3.109756944077	-1.834152634461
C	1.204649923257	-4.528093636381	-1.903536537680
H	2.087918711033	-2.796008334044	-2.834103989212
H	2.643970650053	-3.104139677635	-1.195245929056
C	2.212269104220	-5.533676894788	-2.433590432798
H	0.875351861172	-4.836297259789	-0.898567793012
H	0.303072614150	-4.545609119679	-2.535844033765
C	1.659467037767	-6.945812069760	-2.477716836735
H	1.362235436972	-7.283345144902	-1.477243575149
H	2.391964389555	-7.661820857463	-2.864654709552
H	0.771062921058	-7.001075038008	-3.118798683926
H	3.114480378768	-5.504420246089	-1.805371011815
H	2.534357709281	-5.226341900880	-3.439163621999
H	-0.399923249622	-1.881728575196	0.637231973268
H	1.327702821265	-2.480152718793	0.791405742715
H	-0.208815676084	-2.131102389913	-1.869433447354
H	-0.589205284356	0.569596162848	-0.144271978889

H	-0.219894787070	0.463648235843	-1.918219405212
N	0.492216276126	3.384338000272	-0.840522496168
C	-0.756560342184	3.343488772769	-1.323086522450
C	0.994478562150	4.580851139155	-0.492550374040
C	-1.537991583702	4.474584953965	-1.472112931090
C	-1.013791156806	5.703517904326	-1.107111783065
C	0.278967275923	5.755821316656	-0.608121058374
H	2.009569057392	4.595146782898	-0.105811070667
H	0.735160293229	6.692464450871	-0.309662168709
H	-1.603058418553	6.609017428387	-1.209693200967
H	-2.542984025043	4.382435574608	-1.867403327979
H	-1.142496906948	2.388248423188	-1.597890430285

TS2'
Electronic energy -2617.501347 a.u.
Gibbs free energy -2617.199130 a.u.
48

Ti	-0.870568605848	-1.589896634499	0.179436774549
Cl	-2.080626531605	-2.099448763739	2.074772560053
Cl	-2.334610820574	-2.214266462482	-1.515893280704
N	0.876670340985	-1.317474431437	1.014761244688
C	1.701730846985	-0.873296785811	2.030432222401
C	1.124139905318	-0.359800615113	3.201941150321
C	1.921322367749	0.061898155452	4.252479025719
C	3.100075421537	-0.962462832984	1.956684016015
C	3.885166488484	-0.532910161849	3.014811315873
C	3.307062827043	-0.013729312035	4.166787647375
H	1.452653226153	0.452456593286	5.151392805129
H	0.041087108071	-0.312594710329	3.277812337483
H	3.928640579278	0.319298721855	4.991985535190
H	4.965997806521	-0.613943016131	2.938738581165
H	3.567789794928	-1.387686469119	1.073958868022
C	0.628302040516	-2.994253720847	-0.508683846553
C	1.288068601942	-1.747634988740	-0.251307051991
C	2.088228376443	-0.993047801333	-1.226628914915
C	0.681025284557	-0.630920620263	-1.632873655275
C	0.445886325354	0.839280256415	-1.857266843852
C	1.191583951168	1.335462145353	-3.092585221194
H	-0.622984977994	1.056416477342	-1.983752803961
H	0.789112481712	1.416989581806	-0.987269013237
C	0.954236646116	2.813566796044	-3.356997215034
H	2.270727889151	1.153312808605	-2.977610747619
H	0.874352362981	0.750961680366	-3.969705424192
C	1.684055545521	3.309545933900	-4.590940785031
H	2.767022916155	3.166497882672	-4.492646628351
H	1.505467500893	4.374563142048	-4.771849367496
H	1.361200198087	2.762556746208	-5.485038763400
H	1.270013922159	3.392124045469	-2.476490623300
H	-0.125608927037	2.991474655369	-3.464633694778
H	2.689172158954	-1.598618540757	-1.903771850067
H	2.639741407673	-0.147266097536	-0.813822801388
H	0.661470623734	-3.707135926881	0.317158767266
H	0.620774306290	-3.407512510160	-1.514905405199
H	0.294586944830	-1.277252654953	-2.421849341871
N	-1.518038309986	0.517248094753	0.307708948605
C	-0.799842301632	1.445448825949	0.958662737908
C	-2.731255379005	0.868687267647	-0.151979944507
C	-1.246410873599	2.739072488983	1.149194264986
C	-3.244315620413	2.142569677696	-0.005738819656
C	-2.488046468605	3.100133774276	0.651861452767
H	-2.863196168638	4.110420266287	0.778779761402
H	-0.619076389954	3.446807216347	1.678653344519
H	0.173814252923	1.140652342881	1.326799925574
H	-4.225084460188	2.370776089439	-0.406538525050
H	-3.301440615133	0.098800390548	-0.662151518413

IM4'
Electronic energy -2617.524444 a.u.
Gibbs free energy -2617.220015 a.u.
48

Ti	0.977071876490	-0.083139541026	-2.139056975038
Cl	-1.136008716386	0.656820697040	-2.712816261541
Cl	1.692162731431	-0.620870908261	-4.268508056600
N	1.934657065053	-0.063594864012	-0.431137258653
C	3.247676848330	-0.422372153110	-0.121639684932
C	4.268114149719	-0.207628591278	-1.055032011936
C	5.581450887351	-0.521431460169	-0.743973801648
C	3.583359728935	-0.938501866530	1.135688693247
C	4.899442365217	-1.252734838748	1.435391037970
C	5.906763958779	-1.048730980812	0.499878811446

H	6.358288034641	-0.354887843855	-1.484650250217
H	4.020726491357	0.200241747616	-2.032365503079
H	6.936447542177	-1.295180258834	0.739330914392
H	5.140838531031	-1.656046560608	2.414835295806
H	2.802289659705	-1.083428948489	1.877844545840
C	-0.318095161957	-0.087079606700	0.185019274535
C	0.830799863861	-0.766057310758	0.057944810173
C	0.839497781225	-2.242954764979	-0.225102834143
C	0.874394891539	-2.217082971013	-1.757026650859
C	1.763853811108	-3.269982861369	-2.379772153152
C	1.339618477016	-4.698555046911	-2.055272068457
H	1.780808177567	-3.150645768241	-3.470205590220
H	2.801920103432	-3.117689250206	-2.039124022260
C	2.221973810949	-5.741869014477	-2.721305364090
H	1.347242748969	-4.861400816710	-0.966519011973
H	0.295837127592	-4.844332450244	-2.376669393884
C	1.784915259645	-7.162256340499	-2.416146445383
H	1.809121107475	-7.359618308783	-1.337389648932
H	2.428178830536	-7.903041104722	-2.902737279015
H	0.757952748759	-7.339690077549	-2.758398702877
H	3.262607400720	-5.592694858377	-2.398227294663
H	2.216808720837	-5.575536072450	-3.808307356317
H	-0.039052245700	-2.739218748252	0.202615899758
H	1.735277304591	-2.726900608020	0.179170488979
H	-0.306869873431	1.017888660928	0.235188989164
H	-1.271402773651	-0.574190689904	0.298249949110
H	-0.159369105461	-2.297977506960	-2.150290927396
N	1.970261653816	1.864074139529	-2.509870731680
C	2.182313735632	2.744729981676	-1.437212855743
C	2.104782976453	2.520475769758	-3.723006263728
C	2.535708669264	4.077158947834	-1.535959942944
C	2.456850611939	3.844587132161	-3.905680921729
C	2.677542034726	4.641090868718	-2.793590084131
H	2.954930733055	5.684407454520	-2.905174598399
H	2.697662592525	4.655130543326	-0.633279920368
H	2.067179855322	2.284668608327	-0.470490844609
H	2.551629706910	4.237220382135	-4.911573205853
H	1.921457271005	1.873118259289	-4.574352724971

TS3'
Electronic energy -2617.488047 a.u.
Gibbs free energy -2617.188497 a.u.
48

Ti	4.484983358873	-2.191056591573	-1.838427183014
Cl	4.469691100680	-2.512271288722	-4.143271733340
Cl	6.750784735333	-2.615976780026	-1.437447726942
N	3.650667928641	-1.318829546352	-0.280797406686
C	3.891539803867	-0.115469848287	0.425098331019
C	3.050060695304	0.981003883462	0.255104209846
C	3.313885379292	2.169874662075	0.921326540295
C	4.981459879725	-0.021186606127	1.288960877079
C	5.237810204882	1.167010818922	1.957343807596
C	4.409301209975	2.266682639152	1.771061478973
H	2.660620260111	3.025441848773	0.775589875809
H	2.196020812677	0.892288256709	-0.412538199819
H	4.615127348140	3.198190203185	2.289864415624
H	6.091290042680	1.234920968421	2.625747723503
H	5.623200926774	-0.889223142826	1.418879168302
C	2.226188114368	-2.260244305891	1.489515829825
C	2.805951378672	-2.262148139227	0.282122271650
C	2.623366118423	-3.364980457194	-0.690766211867
C	2.251784937810	-3.091986308458	-1.977291682169
C	1.857302538112	-4.175712822121	-2.928645040099
C	0.400886481678	-4.587855557445	-2.726260475458
H	2.001704885239	-3.845136101380	-3.963583524687
H	2.511124551001	-5.048356773639	-2.782381485832
C	-0.044718871938	-5.665024466086	-3.699983899082
H	0.262013955306	-4.942148175621	-1.693592602472
H	-0.246099462592	-3.703926293606	-2.834255861040
C	-1.494626229425	-6.062992503851	-3.498547075276
H	-1.661288502120	-6.445755505207	-2.484339885869
H	-1.805965148209	-6.841998871818	-4.201964537773
H	-2.161780840668	-5.203735392839	-3.638457941082
H	0.605566958272	-6.544213818475	-3.586618617037
H	0.106313150151	-5.306591489026	-4.728173522264
H	2.625979376051	-4.390093564279	-0.324788467253
H	4.519370889285	-3.581773993437	-0.841542489101
H	2.345587775520	-1.435256748313	2.183794039447
H	1.610566795258	-3.101387629013	1.790137880195
H	1.962871793353	-2.064040062922	-2.223158691693

N	5.10346457403	-0.074252295184	-2.26892428387
C	6.19805943468	0.56621645484	-1.84614556853
C	6.41391883261	1.90984098912	-2.09612110606
C	5.46578695170	2.61829336966	-2.81549924813
C	4.32905492014	1.95762287269	-3.25849563795
C	4.18569561023	0.61743973260	-2.96010537637
H	6.92103371563	-0.02540595611	-1.29381051967
H	7.31659123251	2.38396068534	-1.72829560288
H	5.60977186465	3.67228630602	-3.03915421376
H	3.55979971489	2.46828555883	-3.82592903745
H	3.30137882837	0.07142178518	-3.28220149371

III. β-carbon elimination from 1b (Figure 5)

IM3
Electronic energy -2691.283955 a.u.
Gibbs free energy -2691.011719 a.u.
48

Ti	1.89381789884	1.54443555241	-0.61386880055
Cl	2.30932203936	2.09009848029	1.54357597149
Cl	3.38994505923	2.45379726769	-2.09636411804
N	2.35159770644	-0.28339308371	-0.58286185129
C	3.54457589069	-0.94853185199	-0.21514765072
C	4.54511587011	-1.11216025010	-1.17382836395
C	5.73147508526	-1.74952071810	-0.84505802607
C	3.76779270792	-1.39745170922	1.08876560290
C	4.95477967412	-2.04073430306	1.40920414508
C	5.93783995518	-2.22432212067	0.44467391153
H	6.49785435143	-1.88045194092	-1.60373518552
H	4.36858024652	-0.74765495052	-2.18213635679
H	6.86660988829	-2.72567434570	0.70005764242
H	5.11728166905	-2.38996661017	2.42496491277
H	3.00998944112	-1.22753580229	1.84821279363
C	0.18109999093	0.34953742353	-0.88994809413
C	1.02805427751	-0.85872405733	-0.60120404653
C	0.58749653768	-1.98978522294	0.27127359849
C	0.70619404156	-2.21085134355	-1.21331802237
H	-0.40522829940	-1.93068830100	0.70891754647
H	1.33495944088	-2.46441178937	0.90015159675
H	-0.23227578000	-2.18815596091	-1.76782845010
H	-0.59659215781	0.55413809713	-0.12601512044
H	-0.21237904761	0.39925567544	-1.89129830438
N	0.52574978799	3.33184807840	-0.86325687490
C	-0.72056979251	3.29461302513	-1.35291121848
C	1.04314789990	4.53137237405	-0.54823894243
C	-1.48400023330	4.43182105330	-1.54246163026
C	-0.94375546111	5.66379748813	-1.21289699030
C	0.34592294482	5.71237451297	-0.70563959112
H	2.05587126055	4.54332007126	-0.15522132020
H	0.81443267647	6.65107912252	-0.43391037313
H	-1.51801592957	6.57440293442	-1.34964986466
H	-2.48736396980	4.34198982527	-1.94236148445
H	-1.11927780680	2.31746851923	-1.60105051549
C	1.73875162199	-3.08670809264	-1.82355532934
C	2.41428242826	-4.07838286367	-1.11561047346
C	2.03318594213	-2.92205473184	-3.17955656895
C	3.38763607515	-4.87321222944	-1.73942439898
H	2.19378659716	-4.23493549842	-0.06292809675
C	2.98259979010	-3.71488491157	-3.80573914932
H	1.50625110064	-2.15494776172	-3.74409379249
C	3.65761269680	-4.69381963246	-3.08511817811
H	3.88477048218	-5.63984116379	-1.16896399561
H	3.19778381335	-3.56975118753	-4.86074781502
H	4.40240065820	-5.31628271649	-3.57236180906

TS2
Electronic energy -2691.268362 a.u.
Gibbs free energy -2690.996270 a.u.
48

Cl	0.61538761266	0.37668735017	0.68803811045
Cl	-1.93838673924	1.07725632815	-1.79132671734
N	-1.76104865039	2.74800343169	1.06060035852
C	-1.55052480138	4.08141473090	0.73214421306
C	-1.94029592484	5.11075806043	1.59683826505
C	-1.89628617538	6.43109400680	1.26030944095
C	-0.87249950086	4.40226218776	-0.44753947601
C	-0.83344937600	5.72791657125	-0.77357339757
C	-1.05045438617	6.74997872631	0.07040894054
H	-2.01377512222	7.22016595399	1.93631151309
H	-2.43723623791	4.86419886445	2.53094166483
H	-0.86395357048	7.78706865964	-0.19055143571
H	-0.11532142833	5.96375621216	-1.69867857877
H	-0.54847422102	3.60208988122	-1.10787146301
C	-2.17999015262	1.14606676365	2.62224345558
C	-2.77203824056	2.20231809242	1.64817216389
C	-3.96911269570	1.29239262543	0.74731236505
C	-4.23454605054	2.44587719681	1.69682885185
H	-4.17958965866	1.52134454276	-0.29424475883
H	-4.33980692883	0.33846086443	1.11270260233
H	-4.76101212093	2.15427104407	2.60656942752
H	-2.83958576361	0.43789546724	3.12241175260
H	-1.27012702888	1.42642899484	3.15020057954
C	-4.76775835630	3.73331029288	1.15350542434
C	-4.60849404082	4.12910884703	-0.17351429305
C	-5.44846943723	4.58499805763	2.02321830227
C	-5.93851543252	5.80793989372	1.58817313355
H	-5.58967002861	4.28220393855	3.05855995290
C	-5.09315051899	5.35306716585	-0.61069491397
H	-4.08077491339	3.49195143587	-0.87976124065
C	-5.75808305154	6.19859832875	0.26775163944
H	-6.46588590340	6.45610656836	2.28217981374
H	-4.94934135651	5.64724048172	-1.64637269163
H	-6.13877084934	7.15542026857	-0.07707188315
Ti	-1.61732971037	0.90291428088	0.50356137154
N	-2.22055542163	-1.22928760695	0.69852586062
C	-3.04588571967	-1.80420898790	-0.19068007250
C	-1.70467448272	-1.99603007557	1.67253654611
C	-3.38018568898	-3.14354640000	-0.13952802001
H	-3.44964394573	-1.16313676868	-0.96840154767
C	-1.99235365943	-3.34144989140	1.79187401416
H	-1.03861522186	-1.50637041077	2.37571083047
C	-2.84484181041	-3.92832121814	0.86944646975
H	-4.04982821389	-3.55643621877	-0.88491084379
H	-1.54795051685	-3.91384145351	2.59767057123
H	-3.08810719993	-4.98380214486	0.93809125088

IM4
Electronic energy -2691.303483 a.u.
Gibbs free energy -2691.029425 a.u.
48

Ti	0.31375146933	1.51434960767	0.60120854435
Cl	1.25094509304	1.51520055483	2.68329052164
Cl	0.35920240023	3.59888019458	-0.28968812213
N	1.64998839768	0.62267288551	-0.45877453352
C	2.95993181943	1.19279594570	-0.49214355559
C	3.92213961203	0.76790800117	0.42056636516
C	5.20320395791	1.29812025311	0.37606164858
C	3.29018412612	2.14536471970	-1.45311528858
C	4.57259716224	2.67260120843	-1.49432746019
C	5.53013472534	2.25259207443	-0.57911100412
H	5.94941193979	0.96419935100	1.09093038474
H	3.65436607906	0.01770485587	1.15967880554
H	6.53231373384	2.66956360330	-0.61059024780
H	4.82515985243	3.41579499189	-2.24483573851
H	2.53384269332	2.46249474387	-2.16584658400
C	2.28326596767	-1.00942999876	-2.14450716272
C	1.41505264857	-0.49333349085	-1.26536447451
C	-0.81417186063	0.05381084841	-0.40974264307
C	0.05622611084	-1.07099926027	-0.95325065659
H	2.00942479914	-1.88098639132	-2.72906177564
H	3.27228156782	-0.59111221585	-2.29854980824
C	-0.58735862895	-1.83904141742	-2.08079940715
H	0.21425975473	-1.77997075145	-0.12464987895
C	-0.90022548840	-1.20970001169	-3.28744799648
C	-1.51427368848	-1.90881665968	-4.31434760764
C	-1.83059168283	-3.25424998849	-4.15359637274
C	-1.52443261561	-3.89084660299	-2.96020631503
C	-0.90580997579	-3.18572143763	-1.93331014146
H	-0.65115748921	-0.15916022195	-3.42281137698
H	-2.31274753663	-3.80157683750	-4.95833683820
H	-1.74782240536	-1.40364939544	-5.24746739591
H	-0.66392571473	-3.68888025260	-0.99936426897
H	-1.76468877070	-4.94177755004	-2.82557734434
H	-1.16335582836	0.72883460618	-1.21735464314
H	-1.69671145088	-0.32814001031	0.11468972026
N	-1.73622382765	1.87573032304	1.44432043816
C	-2.11478619244	1.19702749541	2.53920243296
C	-2.63218160722	2.68504743985	0.86129292664
C	-3.37561826368	1.31117446315	3.08980024933
C	-4.29467013648	2.15620824372	2.48613552897

C	-3.914499754303	2.851651606115	1.350219543941
H	-4.597734699326	3.520775355799	0.840256259490
H	-2.305464295298	3.217567814016	-0.026002134972
H	-1.376422668785	0.535024740373	2.982409359047
H	-5.293919117697	2.267679775127	2.894332052215
H	-3.625792013432	0.742086791879	3.977395995413

TS3
Electronic energy -2691.253820 a.u.
Gibbs free energy -2690.984726 a.u.
48

Ti	2.011086015636	1.009469302145	-0.416406375551
Cl	1.761767072775	2.570288932184	1.297014240295
Cl	4.262989440439	0.906921945043	-0.814239749625
N	1.217579994335	1.828944675738	-1.985635448770
C	1.372900050109	3.159099251979	-2.430950447604
C	2.631995907695	3.626776297215	-2.801019023504
C	2.797665828826	4.951991881152	-3.181083972290
C	0.283312762362	4.034728002185	-2.438408800096
C	0.456393280912	5.353313591884	-2.824140848648
C	1.713702110591	5.817955262669	-3.196985135023
H	3.782937614988	5.306575477385	-3.469087232622
H	3.474992458445	2.941748220463	-2.795201714811
H	1.845885379408	6.853755627022	-3.494443175102
H	-0.394972942181	6.027849041893	-2.824445247794
H	-0.694165915695	3.670631268329	-2.132097866064
C	-0.401302191193	1.064861661690	-3.667811275136
C	0.236445100744	0.983425425632	-2.497560415929
C	-0.247699437013	0.418216026809	-0.200657181753
C	-0.011698350777	-0.074997308599	-1.471020923005
H	-0.518844233499	-0.248525126413	0.611267802048
H	-0.515360202164	1.466817229132	-0.085867003681
H	-1.127915423526	0.310437885363	-3.947479841055
H	-0.219788284651	1.881246759885	-4.358906323513
C	-0.264663364236	-1.482622745206	-1.854547711549
H	1.821349772012	-0.381264824629	-1.412761328829
C	-1.207977848862	-2.236689100658	-1.157540437184
C	-1.461083037828	-3.554987807355	-1.513728895350
C	-0.776381395486	-4.133268159063	-2.571747352147
C	0.162057230878	-3.387237552240	-3.278318669675
C	0.414472874349	-2.073811497937	-2.924087784069
H	-1.764878374740	-1.783709399215	-0.342912882036
H	-0.973009352182	-5.164305128485	-2.850264719451
H	-2.201687391718	-4.128083244031	-0.964215971354
H	1.160963442147	-1.497977830728	-3.464799583301
H	0.703028135483	-3.838038971204	-4.105844182248
N	2.169191704583	-0.762668147588	0.947370870721
C	2.670387183970	-1.926849294045	0.501318982109
C	1.773893098371	-0.700524431873	2.229821480553
C	2.778506573016	-3.049730763778	1.299177579602
C	2.385305274208	-2.978637420185	2.619587538982
C	1.858015897737	-1.776589412878	3.092058856205
H	2.990412780905	-1.949489678004	-0.535260781510
H	3.185916250810	-3.962999361705	0.881452687862
H	2.439036261268	-3.841140766538	3.271393016934
H	1.524210789299	-1.665827880971	4.117263824446
H	1.372923458951	0.248782096631	2.569751441513

IM3'
Electronic energy -2691.283955 a.u.
Gibbs free energy -2691.011719 a.u.
48

Ti	1.893817698845	1.544435552417	-0.613868800552
Cl	2.309322039362	2.090098480291	1.543575971495
Cl	3.389945059230	2.453797267690	-2.096364118042
N	2.351597706446	-0.283393083716	-0.562861851297
C	3.544575890694	-0.948531851996	-0.215147650729
C	4.545115870113	-1.112160250101	-1.173828363952
C	5.731475085261	-1.749520718101	-0.845068026074
C	3.767792707924	-1.397451709225	1.088765602906
C	4.954779674127	-2.040734303063	1.409204146085
C	5.937839956160	-2.224322120679	0.444673611537
H	6.497854351438	-1.880451940927	-1.603735185529
H	4.368580246528	-0.747654950529	-2.182188356795
H	6.866809888291	-2.725674345703	0.700057642427
H	5.117281669059	-2.388956610172	2.424964912779
H	3.009989441120	-1.227535802294	1.848212793636
C	0.181099990934	0.349537423530	-0.869948094131
C	1.028054277515	-0.858724057333	-0.601204046534
C	0.587496537688	-1.889785222942	0.271273598495

C	0.706194041560	-2.210851343555	-1.213318022370
H	-0.405228299402	-1.930688301001	0.708917546477
H	1.334959440881	-2.464411789370	0.900151596758
H	-0.232275780005	-2.188156960918	-1.767828450105
H	-0.596592157813	0.554138097135	-0.126015120442
H	-0.212379047615	0.399255675449	-1.891298304387
N	0.525749787991	3.331848078407	-0.863256674903
C	-0.720569792514	3.294613025135	-1.352911218483
C	1.043147899906	4.531372374059	-0.548238942430
C	-1.484000233300	4.431821053301	-1.542461630268
C	-0.943755461118	5.663797488131	-1.212896990363
C	0.345922944821	5.712374512978	-0.705639591125
H	2.055871260552	4.543320071268	-0.155221320207
H	0.814432676474	6.651079122529	-0.433910373132
H	-1.518015929571	6.574402934421	-1.349649864664
H	-2.487363969808	4.341989825279	-1.942361484456
H	-1.119277606806	2.317468519232	-1.601050515493
C	1.738751621999	-3.086708092647	-1.823555329346
C	2.414282428265	-4.078382863672	-1.115810473468
C	2.033185942130	-2.922054731847	-3.179556508951
C	3.367636075159	-4.873212229442	-1.739424398983
H	2.193786597165	-4.234935498427	-0.062928096757
C	2.982599790104	-3.714884911676	-3.805739149321
H	1.508251100647	-2.154947761720	-3.744093792494
C	3.657612666803	-4.693818632468	-3.085118176118
H	3.884770482183	-5.639841163795	-1.168983995610
H	3.197783613353	-3.569751167536	-4.860747815022
H	4.402400658206	-5.316282716498	-3.572361809081

TS2'
Electronic energy -2691.270595 a.u.
Gibbs free energy -2690.998925 a.u.
48

Ti	-0.889516549359	-1.510073068855	0.210835185521
Cl	-2.018645350066	-2.082356133876	2.105935896205
Cl	-2.349072183918	-2.142659802510	-1.490027912731
N	0.884947432421	-1.239424041074	0.966810085604
C	1.737689180214	-0.836443797908	1.979185432582
C	1.194966758943	-0.247533394453	3.131334907826
C	2.017058594113	0.144771081506	4.173765071470
C	3.125339053914	-1.030690975930	1.915197008645
C	3.936353318038	-0.628951691883	2.965063232221
C	3.393782104290	-0.034967895771	4.097727142451
H	1.575441576885	0.595272914227	5.058151548032
H	0.118311878139	-0.117147702809	3.200521205558
H	4.035261737558	0.276072418877	4.916230687667
H	5.008449093039	-0.791830694997	2.897838518258
H	3.563919257804	-1.516973047887	1.049424004132
C	0.530450673835	-2.957568878547	-0.500805740795
C	1.270307947186	-1.747754088091	-0.280940580137
C	2.152328783945	-1.112334046109	-1.274846259181
C	0.824603031364	-0.585586391356	-1.713965280951
H	2.647780492048	-1.815817635329	-1.942383035941
H	2.837208803096	-0.361698596819	-0.887277015681
H	0.328749978131	-1.184154726213	-2.474729046293
H	0.552659960985	-3.658620434768	0.337248604704
H	0.492989553951	-3.392311753592	-1.497247626079
N	-1.595627388934	0.573502107593	0.267273117272
C	-0.896099190275	1.524919619054	0.906096206098
C	-2.862004352345	0.856959825649	-0.089163267791
C	-1.421527417602	2.768650537850	1.201769457755
C	-3.455578827336	2.075647983002	0.170350834865
C	-2.724481582254	3.053021386526	0.828368503215
H	0.119573465430	1.275245219259	1.190807238612
H	-0.805478049100	3.497435188341	1.716140476330
H	-3.165223589181	4.019983930226	1.048250719473
H	-4.479144874079	2.246343675952	-0.142365636245
H	-3.411358782909	0.072326196826	-0.599238494397
C	0.596471215224	0.855934156696	-1.813383572742
C	1.415598258475	1.815079251341	-1.205608015562
C	-0.464727190943	1.314978244315	-2.609715320073
C	1.190845854859	3.171720238404	-1.397645754593
H	2.243904002197	1.508669765300	-0.572225466902
C	-0.684162068427	2.666814546621	-2.803518036859
H	-1.114347571987	0.585291857755	-3.089158704208
C	0.143919128991	3.607551572325	-2.197035878647
H	1.845698692918	3.894077325285	-0.917975936782
H	-1.506500078275	2.992093815722	-3.434890185695
H	-0.025818980924	4.668938938146	-2.351298314214

IM4’
Electronic energy -2691.312411 a.u.
Gibbs free energy -2691.036858 a.u.
48
Ti -0.241553140260 2.958012006075 0.103426434868
Cl 1.115974169380 1.496961550208 1.215358128745
Cl 0.563118660485 5.051596696906 0.523137778053
N 0.380689680545 2.708825384390 -1.705691522112
C 1.770997375979 2.496587024403 -1.949468899061
C 2.259230260018 1.198475321523 -2.088011514122
C 3.608060759693 0.987513023444 -2.322884092556
C 2.638641035949 3.578288919111 -2.053739700255
C 3.990947977273 3.361528848509 -2.287081741303
C 4.477906058243 2.068807244995 -2.420067136193
H 3.985153453504 -0.025740413990 -2.426348100244
H 1.569460257050 0.362831036319 -1.997689255861
H 5.535974559131 1.900887454834 -2.597725201883
H 4.668100128800 4.208839690620 -2.382650908221
H 2.245696642492 4.585812252957 -1.949808508577
C -0.114580043804 2.488770837968 -4.077931407423
C -0.476408240190 2.699953452130 -2.805329727049
C -1.880514733281 2.990626783067 -2.368064689877
C -2.106953173071 2.553011154424 -0.927386819635
H -2.603495752754 2.543194770851 -3.065308208053
H -2.026975903321 4.078989664301 -2.420902865963
H -0.871219149825 2.493623814954 -4.855840734353
H 0.915813558826 2.326687994080 -4.375790694482
C -2.403484650516 1.123013887554 -0.677028963839
H -2.865779068976 3.182285728353 -0.450030614937
C -3.279188827297 0.752326713165 0.355097421674
C -3.530001585024 -0.578727090319 0.645744183948
C -2.915037941472 -1.584480514473 -0.091527884798
C -2.057186289726 -1.239192610007 -1.127864222576
C -1.801615542441 0.093333805500 -1.417030102282
H -3.779260813873 1.532018664393 0.927062132018
H -3.111094598496 -2.628460417242 0.133820043027
H -4.217227940252 -0.834149321538 1.447615825854
H -1.132372824698 0.339059574679 -2.237717233116
H -1.580543537043 -2.015915227045 -1.719487929091
N -1.631209862879 3.161053511216 1.872450249332
C -1.721723414047 2.228431213464 2.831754756184
C -2.429176453499 4.237376887025 1.961306067600
C -2.601704404658 2.332539334580 3.893816434344
C -3.334419946051 4.414532639122 2.987875178613
C -3.425391843055 3.442439273252 3.974172789039
H -1.070521671370 1.365643836868 2.739156901911
H -2.633210998580 1.546621058530 4.639593331768
H -4.128675400306 3.551493869493 4.793461741285
H -3.954552336924 5.303074296885 3.008264494405
H -2.337264489479 4.979376374469 1.173143885193

TS3’
Electronic energy -2691.312411 a.u.
Gibbs free energy -2691.036858 a.u.
48
Ti 0.924886889113 0.160404216949 -0.729678815783
Cl 2.893869086949 -0.647207432623 -1.497634727925
Cl 1.262711674925 0.653339213209 1.525267290010
N 0.857656389260 1.864551300177 -1.637302792309
C 1.923610318512 2.766195977418 -1.864704479018
C 2.525619838339 2.859078236208 -3.120607682776
C 3.595858538751 3.718049541895 -3.310951917781
C 2.415185061881 3.522566999931 -0.804458936733
C 3.490006773719 4.379041692690 -1.002284893837
C 4.080307153780 4.481633004350 -2.254054100727
H 4.062687140211 3.785407758991 -4.289290423978
H 2.155377420642 2.242222116274 -3.935121401392
H 4.922044620248 5.150265720473 -2.406959598594
H 3.867388447675 4.967513499910 -0.171252246023
H 1.943812771264 3.433655123081 0.170533315442
C -0.744934367204 2.814331357446 -3.251613498032
C -0.350627547171 1.946611140984 -2.315392816850
C -1.207328216723 0.845671181664 -1.804335819253
C -1.455051832859 0.790537295094 -0.450760462007
H 0.093876294274 -0.378568511613 -2.154040104756
H -1.812196753436 0.291657900538 -2.517529585984
H -1.734540343171 2.718457037894 -3.685525090701
H -0.113384029273 3.628976513621 -3.588301934494
C -2.422267175286 -0.091870689800 0.199716785358
H -1.087431219892 1.618036019421 0.156219128036
C -2.748178073409 0.136274164630 1.538130636402
C -3.669668584680 -0.665278399936 2.194292286388
C -4.282888849591 -1.715124223732 1.522643526353
C -3.965552894887 -1.955258823428 0.189752843139
C -3.045099922728 -1.155358231788 -0.467019811724
H -2.267553529175 0.957704191926 2.064984510106
H -5.004681875377 -2.344743150360 2.034216971418
H -3.911312873859 -0.468270646249 3.234623234934
H -2.801468802581 -1.369038887300 -1.504714399530
H -4.438316052607 -2.776464744316 -0.341532102290
N 0.150417291815 -1.892579718700 -0.235227200658
C 0.091483037342 -2.832319406698 -1.195540083211
C -0.213892927338 -2.249144436433 1.007835112431
C -0.329737255906 -4.125377643653 -0.955983065310
C -0.706557546591 -4.480982762372 0.330059299234
C -0.644211110098 -3.522935582140 1.328116514118
H 0.394055350779 -2.525270097613 -2.191340150019
H -0.355088086801 -4.838125543701 -1.772187491803
H -1.041540491150 -5.489583793630 0.549901551296
H -0.927497546955 -3.747282955622 2.350141330553
H -0.157898390751 -1.481405523069 1.771181018256

IV. N-H reductive elimination pathway (Figure S58)

TS4-NH
Electronic energy -2369.26021 a.u.
Gibbs free energy -2369.040651 a.u.
37
Ti 0.741953319617 -1.023635368617 -0.628502051597
Cl -1.063149527898 -1.685913119831 -1.846465442577
Cl 0.045594546733 0.359720136479 1.009517255302
N 2.743939571298 -0.878843724908 -0.444820866362
C 3.938629555591 -0.173984785885 -0.267417250173
C 5.150046663101 -0.660410366232 -0.764218902610
C 6.324046691683 0.034509832976 -0.519969450053
C 3.920886495850 1.013920904406 0.467590940435
C 5.100662276050 1.701366243699 0.698806459462
C 6.308663844561 1.217069222468 0.209417221586
H 7.261357042931 -0.353110465308 -0.908469356528
H 5.164715915463 -1.583934109401 -1.336208340760
H 7.231254731985 1.758184458142 0.394362152793
H 5.076250383486 2.625248163831 1.269186771973
H 2.974097555002 1.386235828860 0.849581549739
C 2.315535689979 -1.342513372626 -2.709596295309
C 2.536639922367 -1.857455005703 -1.438555198920
C 1.441929432709 -3.101563684200 0.345195191406
C 1.887861119226 -3.065210197417 -0.950909871963
C 1.522461957402 -4.121505074028 -1.954908712535
C 0.694080890117 -5.285643522007 -1.447258884203
H 1.005533871528 -3.662194801199 -2.807320834600
H 2.472761983861 -4.496080301996 -2.366120886244
C 0.401395102781 -6.291292720355 -2.548563025893
H 1.214131627814 -5.790550118464 -0.620126026033
H -0.258759744307 -4.918464358541 -1.038152692432
C -0.425116431836 -7.462970019148 -2.054661174559
H 0.094758132205 -8.002656250705 -1.253882736552
H -0.635601793498 -8.179374602847 -2.855100883662
H -1.386883831673 -7.123541703349 -1.651592107637
H 1.349422687879 -6.652722556378 -2.972027482639
H -0.124432215604 -5.782261060036 -3.369045940964
H 0.786194077998 -3.901768981436 0.669562002495
H 1.875557089715 -2.501228392146 1.137552752671
H 1.910714362599 0.109689761016 -1.191203464493
H 1.938197246594 -1.952631260703 -3.523715261731
H 2.806239756689 -0.416234628411 -2.991529156833

IM6-NH
Electronic energy -2369.320381 a.u.
Gibbs free energy -2369.094472 a.u.
37
Ti 0.929921223448 -1.425755925666 -0.098531299488
Cl -0.416805903493 -2.145626753859 1.660317676683
Cl -0.691785097414 -1.246143878848 -1.780345081441
N 2.573305301018 -0.465739793974 -1.239801456941
C 3.785396100666 -0.019246117299 -0.597320100707
C 4.972251287118 -0.732546091990 -0.694400428006
C 6.104779114367 -0.264402574310 -0.039612849129
C 3.732420113638 1.159178282773 0.140574443887
C 4.869783181697 1.622641514157 0.782313042049
C 6.059771240283 0.909621451000 0.697848636243

H	7.031708017708	-0.824460993701	-0.116488266675
H	5.015262578763	-1.642561560567	-1.284151661681
H	6.949655554165	1.271382216434	1.203180552887
H	4.825068383638	2.545082588064	1.353038810789
H	2.797344983356	1.711182966945	0.200716390848
C	2.871938955854	-1.591188123853	-3.366998491388
C	2.595267075893	-1.679693496891	-2.064999413447
C	2.386538031384	-2.876099949719	0.105110049546
C	2.045557419509	-2.793975402235	-1.301099655826
C	1.506286891512	-3.955036201012	-2.091987259457
C	0.732357253525	-4.996794426407	-1.303672911268
H	0.860825127070	-3.559986914465	-2.891319370618
H	2.344225881767	-4.456050986205	-2.607865657074
C	0.104471720364	-6.046106623304	-2.206401325030
H	1.392429608510	-5.494938745914	-0.578374264093
H	-0.059454580029	-4.510516245088	-0.712498309113
C	-0.619222573885	-7.129012638813	-1.429330997580
H	0.069118389949	-7.662830354927	-0.762761239069
H	-1.079727930812	-7.869329104975	-2.091801840926
H	-1.413788414162	-6.701508133886	-0.805847669328
H	0.886610096693	-6.496475898430	-2.835252356070
H	-0.593189078701	-5.551977404143	-2.897877410962
H	2.076266288505	-3.779028450404	0.627385019392
H	3.315910580556	-2.454007881636	0.494835333180
H	2.189521491603	0.305997247538	-1.790695991977
H	2.819977483181	-2.459721459489	-4.013948740245
H	3.152174202756	-0.641004134901	-3.812835907965

IM7-NH
Electronic energy -2941.852449 a.u.
Gibbs free energy -2941.460421 a.u.
63

Ti	-0.243267550436	-0.277860982973	0.262754635969
Cl	-0.319460525935	-0.178640326625	2.502228801407
Cl	-1.685582025465	-1.990483225948	-0.313845089497
N	2.801426386036	0.243178284419	-1.878769700329
C	4.155662830243	0.025059945030	-1.612635489542
C	4.746651665582	-1.236954819610	-1.696212963006
C	6.092562764029	-1.395740284855	-1.394998787404
C	4.945227073424	1.119511136328	-1.245206004268
C	6.289808990115	0.951101332851	-0.962527584357
C	6.873710888578	-0.309433838555	-1.026640288545
H	6.535934841854	-2.384963463713	-1.465207560161
H	4.163560296291	-2.096476772876	-2.015613619734
H	7.926773762832	-0.440599118236	-0.799017906618
H	6.886785997928	1.814696438416	-0.683443962452
H	4.487332521935	2.104510258134	-1.187174789576
C	1.070402803494	-0.647824673028	-3.260235828640
C	1.880176496796	-0.757908681173	-2.197757836815
C	1.854866740048	-1.576505549506	0.121409371003
C	1.679747264393	-1.838129366271	-1.194951968692
C	1.264867446573	-3.178055732140	-1.711921847862
C	1.214421837991	-4.316654666459	-0.712814609269
H	0.284217101306	-3.078966754789	-2.200178233550
H	1.958330061984	-3.429747374503	-2.529074570333
C	0.742895015480	-5.606857617122	-1.362975009294
H	2.209880069052	-4.467219419915	-0.268783874404
H	0.536183435042	-4.068841418113	0.115572704898
C	0.733841243461	-6.774387961051	-0.395600522309
H	1.738664537483	-6.966182682625	-0.000162514907
H	0.383810654386	-7.695472003807	-0.872465640495
H	0.077092191849	-6.572234421447	0.458955898384
H	1.387224568400	-5.837938947790	-2.223558853107
H	-0.266414026450	-5.450222930868	-1.771242705010
H	1.743825268409	-2.349378074855	0.877231387372
H	2.307494611886	-0.633933104398	0.438911680429
H	2.579372258955	1.161086959102	-2.247920234561
H	0.252512420562	-1.336789210366	-3.435972735115
H	1.184972016437	0.179953253493	-3.954174430242
N	-1.334148470225	1.100544056491	-0.423474838053
N	-0.014749188965	1.524355945416	-0.221123882654
C	-1.933936775294	1.355882792222	-1.663704882309
C	0.241159843586	2.683926540251	0.516086438023
C	-3.184867641011	0.787856294263	-1.922217676153
C	-1.343747033870	2.190297964215	-2.615384942244
C	-3.821175354031	1.037047608468	-3.125806159289
C	-1.993384615234	2.432519880518	-3.817400948047
C	-3.230313832509	1.858680538088	-4.081103532374
H	-1.528545060207	3.083142475657	-4.552873166760
H	-3.735518297948	2.054736185488	-5.021796312119
H	-4.791337423027	0.588653617108	-3.319503534050
H	-3.641295436442	0.154827216773	-1.167060505347
H	-0.377405413932	2.639967172803	-2.405733024212
C	-0.757347741175	3.605769872481	0.831663607936
C	1.557690543528	2.917323294338	0.923824319289
C	1.865739432456	4.055003217504	1.649598221613
C	0.872044257504	4.974391546669	1.971129077996
C	-0.434348344300	4.741667001314	1.559995766934
H	-1.778659777846	3.417670589167	0.514608279989
H	-1.216272432849	5.453534673872	1.808866964725
H	2.325566072848	2.191741076481	0.663137856964
H	2.889734616778	4.226033648199	1.968868506309
H	1.115816137617	5.866352608054	2.539805044466

Enamine
Electronic energy -599.344718 a.u.
Gibbs free energy -599.112851 a.u.
34

N	5.145430930558	-1.214953233153	-2.465703852196
C	4.812590356070	-0.249751415341	-1.513569586839
C	5.842500887797	0.509553375742	-0.946382153512
C	5.564374628693	1.478445568061	0.002458497238
C	3.497819480317	-0.005686020139	-1.107556999783
C	3.232762833748	0.957949377400	-0.144432001733
C	4.257269327493	1.706324923843	0.419267591703
H	6.380134714212	2.057660637259	0.426284414123
H	6.866774151636	0.323875531957	-1.262122600688
H	4.041119373368	2.461241370609	1.168699560998
H	2.203797886690	1.131649999732	0.158630651197
H	2.678667832075	-0.560696322589	-1.555118951619
C	4.089899701681	-2.050961429384	-4.426545256744
C	4.212620319287	-2.061364488839	-3.093677766281
C	4.008189097889	-3.396158661947	-1.058671152491
C	3.478794942244	-3.013314338712	-2.221629883514
C	2.148013750635	-3.500487808925	-2.723562404360
C	1.345914734085	-4.372081292012	-1.776130426139
H	1.546751499848	-2.621411345453	-3.006144709045
H	2.298774824477	-4.050334736070	-3.665172615758
C	0.002578785800	-4.775370080035	-2.362136601766
H	1.914541554414	-5.279353577347	-1.524031241318
H	1.182288182200	-3.840185889987	-0.826778807141
C	-0.804432779067	-5.646825526048	-1.418604179000
H	-0.264249416942	-6.570077071597	-1.176032728784
H	-1.770089605569	-5.931355100612	-1.849062656156
H	-1.002754881337	-5.125714516745	-0.474073908577
H	0.166246357171	-5.305026874227	-3.311792542634
H	-0.568380311698	-3.870285452121	-2.614927176818
H	3.506955670406	-4.098437673760	-0.401070800349
H	4.972845118489	-3.020455372456	-0.731984459461
H	5.936831971644	-0.962941441916	-3.045747462037
H	3.475715942916	-2.771541665950	-4.953600813079
H	4.651702138771	-1.340979449237	-5.027016977438

TS5-NH
Electronic energy -599.252744 a.u.
Gibbs free energy -599.028957 a.u.
34

N	5.009861629713	-1.054733824557	-2.620798200438
C	4.731979404849	-0.162862642198	-1.559566550290
C	5.805207921805	0.365481172780	-0.844209951536
C	5.581452395071	1.270822882602	0.181934396350
C	3.434071767665	0.249312746732	-1.254564778171
C	3.215257490458	1.151854655537	-0.225293378997
C	4.286402533039	1.663383177288	0.498090458826
H	6.423031024749	1.672686567859	0.738533827495
H	6.811789895964	0.048189608445	-1.102989277255
H	4.112356760138	2.374354280519	1.300072683396
H	2.201959627909	1.466001173699	0.007974280714
H	2.600309412645	-0.133647025147	-1.838546860937
C	4.406107598588	-2.141456429373	-4.434155453168
C	4.236352651977	-2.068142057703	-3.016854576261
C	4.011445743059	-3.317770930081	-0.959223617188
C	3.474663674498	-2.957338538434	-2.126386225419
C	2.154262842800	-3.441223845562	-2.652113481010
C	1.366131130501	-4.360483713417	-1.741025677007
H	1.542679269628	-2.559597891172	-2.905090640512
H	2.332220131316	-3.938628891071	-3.618229997512
C	0.038611384174	-4.780111306968	-2.350703082425
H	1.959851447264	-5.258232964350	-1.512394629180
H	1.181613384599	-3.863144422094	-0.776782718322

C	-0.752599138624	-5.700795489284	-1.441298755227
H	-0.189998737784	-6.616326844121	-1.221697568438
H	-1.706326711110	-5.997593761508	-1.889539756309
H	-0.973193989611	-5.214013350587	-0.483509774558
H	0.224151943777	-5.275683953608	-3.314465234851
H	-0.553486243376	-3.882644256648	-2.580671871141
H	3.512778642116	-4.000372188462	-0.278099012280
H	4.989032947381	-2.952567748147	-0.657503818071
H	5.355213789463	-1.133902091187	-3.874182908300
H	4.315005298640	-3.096072632842	-4.956845669023
H	3.895590076720	-1.324766466944	-4.954016182955

V. β-carbon elimination from 1a (Figure S59)

IM3
Electronic energy -2369.322975 a.u.
Gibbs free energy -2369.097514 a.u.

Ti	1.276851030351	1.395649961524	-0.831923266204
Cl	1.047143760399	1.853167374860	1.361818183599
Cl	2.575935227715	2.798412477171	-2.098600740207
N	2.071711319176	-0.242958951244	-0.837490962364
C	3.334221049530	-0.784646929890	-0.523446740310
C	4.303592295218	-0.863832184824	-1.521376473463
C	5.547296774593	-1.404939987565	-1.235682368625
C	3.630875681680	-1.208390563277	0.771307876848
C	4.876690597203	-1.753955161270	1.047008138257
C	5.833792437102	-1.860741843774	0.045695579756
H	6.296402340974	-1.473617860065	-2.018905996718
H	4.057394648819	-0.516896617631	-2.521398457253
H	6.807321038889	-2.287574395474	0.266769712771
H	5.103503400843	-2.089305281374	2.054710475383
H	2.883871653787	-1.105150913902	1.553167835456
C	-0.232329920365	0.091979898713	-1.314717301825
C	0.787449355190	-0.961515346098	-0.918484227868
C	0.412806772436	-2.055305105181	0.026650940994
C	0.641108652016	-2.366288477284	-1.438352372891
C	1.775924274134	-3.271498465855	-1.835323207777
C	1.366301901383	-4.736049864001	-1.788492330601
H	2.125694100627	-3.019322245743	-2.847853405543
H	2.634870012428	-3.125007444376	-1.168107687181
C	2.495960646530	-5.678600667253	-2.167496520510
H	1.012642317151	-4.979028753760	-0.774140150980
H	0.508127678237	-4.903007340819	-2.457783680570
C	2.089495652666	-7.138203230746	-2.093260561137
H	1.771232595944	-7.406964527585	-1.078581520321
H	2.911126482883	-7.806798588988	-2.370339785716
H	1.249403364155	-7.348399126288	-2.766432323522
H	3.354146488662	-5.495060730316	-1.504516905002
H	2.841912954345	-5.437415717750	-3.183054883162
H	-0.597236976356	-2.051010578628	0.427103993233
H	1.177460501691	-2.430767248541	0.702916860779
H	-0.262622344546	-2.500994031170	-2.034786509463
H	-1.128336222389	0.123592526338	-0.691868972477
H	-0.459594543102	0.093825942066	-2.385610245386

TS2
Electronic energy -2369.301236 a.u.
Gibbs free energy -2369.077466 a.u.
37

Ti	0.723887609977	-1.382298020963	0.153202553976
Cl	0.481960198406	-2.599918614109	-1.811439260417
Cl	2.840938251134	-2.014109167263	0.841461739396
N	0.388650915630	0.476262083692	-0.039264164910
C	1.174193202452	1.618556678777	-0.190449549700
C	1.005465653016	2.746661926267	0.616448019841
C	1.807037801416	3.861626244827	0.418795138488
C	2.143382724378	1.631057973227	-1.199277996955
C	2.937250721447	2.748880898004	-1.383473204764
C	2.775566992580	3.870422316021	-0.576045950480
H	1.669165926456	4.733638414450	1.051451245324
H	0.246236163708	2.744641368743	1.394189406730
H	3.399168332022	4.746148001125	-0.725188977203
H	3.689728545620	2.745553038483	-2.166495137775
H	2.259300221423	0.753017900688	-1.831653687921
C	0.362342961447	-0.343935834844	2.144537907452
C	-0.413213861557	0.219767477104	1.112845457759
C	-1.487683530746	-1.182149026100	0.421743744930
C	-1.882092075471	0.166235386468	0.993655698197
C	-2.555069173456	1.148865161640	0.058515564096
C	-4.072609472241	1.041100255839	0.057789525774
H	-2.269020685058	2.164972559225	0.360444054407
H	-2.164114500522	1.007135012864	-0.958680118660
C	-4.623245542779	-0.286937018226	-0.438289376014
H	-4.448717043331	1.228937490111	1.075498476546
H	-4.479469776837	1.848540039799	-0.567210525890
C	-6.139454143965	-0.286438144918	-0.496642115284
H	-6.571293779356	-0.088423056017	0.492100637121
H	-6.536476630574	-1.245002379645	-0.845865682864
H	-6.506283864499	0.492750625764	-1.175830513043
H	-4.279556710501	-1.101742446978	0.214297928014
H	-4.210493670500	-0.500877479857	-1.435073544353
H	-1.594500878891	-2.026690052999	1.111901697676
H	-1.784948160186	-1.338708662701	-0.616088425336
H	-2.360899045693	0.114115351879	1.974704318037
H	-0.124544061203	-0.772485179298	3.018038507053
H	1.360140405988	0.065050129133	2.282023173627

IM4
Electronic energy -2369.341905 a.u.
Gibbs free energy -2369.115967 a.u.
37

Ti	1.420830943033	-1.079014369398	1.218790473195
Cl	2.723115217851	-2.358691760003	2.601425989123
Cl	0.279386697575	0.928152486923	1.046407254655
N	2.448468397332	-0.956644918815	-0.354911196541
C	3.758176355953	-0.420650512115	-0.361752431534
C	4.786788682445	-1.108851153501	-1.012180947677
C	6.078567098041	-0.616167037319	-0.974433448486
C	4.045507204885	0.757007721130	0.326892034736
C	5.349145488691	1.236298964700	0.371818401627
C	6.365831606116	0.556470513315	-0.280375951043
H	6.872240032669	-1.153488777775	-1.485042298090
H	4.556965579094	-2.028711634562	-1.543023186546
H	7.382226579127	0.936883629445	-0.252462776992
H	5.563752662458	2.153984487693	0.911012270248
H	3.241476561960	1.305615767846	0.811763746183
C	1.998266843513	-0.635731559838	-2.714873397931
C	1.784730607268	-1.267558909935	-1.560160894579
C	0.276614015395	-2.347721356008	0.067690178124
C	0.814436813666	-2.406942218853	-1.363744061699
C	1.535328344260	-3.726890791634	-1.652858043730
C	0.616430889771	-4.934296140330	-1.712779616000
H	2.053121636985	-3.628601533774	-2.618390050692
H	2.319454688744	-3.881263678604	-0.892639842881
C	1.335784176363	-6.195580824969	-2.162470605775
H	0.149672176183	-5.113086926644	-0.732791933286
H	-0.210365275454	-4.722648265530	-2.409248950882
C	0.413696764743	-7.397318995162	-2.242980195175
H	-0.028843335458	-7.622649710095	-1.265165731841
H	0.940380899910	-8.294880978265	-2.583292544294
H	-0.411607729406	-7.212672117233	-2.941632297395
H	2.166718990688	-6.404701313794	-1.473395535596
H	1.795799656004	-6.013731713045	-3.144562781902
H	-0.732811996826	-1.917949954708	0.142548507882
H	0.280329287769	-3.316571911829	0.585984310139
H	-0.004176237656	-2.301687837503	-2.089976599420
H	1.438553356715	-0.926990229500	-3.598307964996
H	2.718160319591	0.170652559689	-2.811779880932

TS3
Electronic energy -2369.292315 a.u.
Gibbs free energy -2369.073137 a.u.
37

Ti	-1.000383813753	0.389316825915	0.341769749896
Cl	-1.230770524387	0.629342445219	2.610987873047
Cl	-2.460410839225	1.943533504196	-0.530245949607
N	0.692434903999	0.715468195149	-0.447256809237
C	1.723139382534	1.632754749704	-0.183459417274
C	3.065019551920	1.244130450185	-0.166328542306
C	4.045739145828	2.162329324385	0.172850472757
C	1.384900558561	2.945834580543	0.149248421528
C	2.373542686016	3.854143660461	0.494218452920
C	3.707775565109	3.469406370026	0.503713787234
H	5.085701124905	1.849517584325	0.186535915089
H	3.332815718874	0.219828584116	-0.407559825021
H	4.481314061619	4.182958607115	0.769791628952
H	2.097066109692	4.871991836485	0.752255062144
H	0.340338751752	3.247362953974	0.129143497201

C	1.543525952815	-0.446436329668	-2.434444870032
C	0.795927682480	-0.376969777404	-1.330351165517
C	-0.085854958644	-1.696568091209	0.502696710172
C	-0.108731208975	-1.481139339341	-0.869483901187
C	-0.654829472323	-2.444471509631	-1.878004521049
C	-1.800321380173	-3.317886816912	-1.403826698867
H	-0.948745516124	-1.892520361742	-2.779897699434
H	0.192784581492	-3.085540586241	-2.170176049329
C	-2.348108060300	-4.191200053867	-2.520650370061
H	-1.474280570326	-3.957331895275	-0.571617385687
H	-2.606384688714	-2.678890693002	-1.009956295652
C	-3.486914561671	-5.078232531900	-2.056086922356
H	-3.161310614783	-5.751345021325	-1.253886841564
H	-3.878512281192	-5.697043397311	-2.869691912475
H	-4.318157222762	-4.478978424202	-1.665410783153
H	-1.534778799297	-4.808136626814	-2.928873163066
H	-2.686487396162	-3.549256782300	-3.346151602750
H	-0.685594466363	-2.487825016855	0.943688146128
H	0.746120909670	-1.315951889097	1.093392130175
H	-1.587401205118	-0.519618090651	-1.000642910610
H	1.537410432990	-1.337884549053	-3.050453429672
H	2.195436460036	0.368629112004	-2.728849781340

TS2'

Electronic energy -2369.301236 a.u.

Gibbs free energy -2369.077466 a.u.

37

Ti	1.162677101588	-0.203281084360	-2.158324155285
Cl	1.193535589791	2.137193059081	-2.484213064777
Cl	0.518985442739	-0.494051412060	-4.402196702650
N	2.134252174665	-0.563061858080	-0.448676206705
C	3.229962932832	-1.347117816847	-0.083556303919
C	4.309341616196	-1.437217971932	-0.969305247067
C	5.417068528406	-2.201846004187	-0.647457813956
C	3.311141634831	-1.985811648506	1.156363678048
C	4.428502630182	-2.744373437158	1.472721965896
C	5.479753192026	-2.867224851259	0.572674318837
H	6.241974271767	-2.272467397017	-1.350366140922
H	4.263440070455	-0.898335958521	-1.914010242313
H	6.351502615578	-3.462026690680	0.826581213784
H	4.480786465000	-3.238400432982	2.438465046629
H	2.501018355921	-1.877234002739	1.872248769835
C	0.084780511141	0.426486745835	-0.175563634525
C	0.845715876748	-0.714247304230	0.007170353309
C	0.193966020484	-2.064793375841	-0.062217937587
C	0.143015179347	-2.042510011517	-1.579733261784
C	1.173625586606	-2.795274392303	-2.358508228568
C	1.815610992783	-4.016502091389	-1.711579047114
H	0.756688668233	-3.061538300139	-3.336270672978
H	2.040042622494	-2.121529825471	-2.657855632812
C	2.716805378167	-4.774847954334	-2.670331516182
H	2.397855320746	-3.729355730852	-0.825956258982
H	1.014210840268	-4.680039954387	-1.354442056502
C	3.371549907501	-5.971610534510	-2.007341232095
H	3.997643054538	-5.659313138320	-1.162311840676
H	4.005981259798	-6.528805959321	-2.703852444604
H	2.617268009844	-6.666093426842	-1.617477570812
H	3.487032471193	-4.092831211271	-3.060185417762
H	2.129740250925	-5.100459682703	-3.540542360404
H	-0.792709607610	-2.053538720918	0.402597371474
H	0.786051069693	-2.874882789392	0.364940299630
H	0.542913136661	1.394389165693	-0.023778443782
H	-1.000485435860	0.361986525323	-0.149927569963
H	-0.862152735675	-2.003994525864	-2.003657012712

IM4'

Electronic energy -2369.341905 a.u.

Gibbs free energy -2369.115967 a.u.

37

Ti	1.357531704377	-0.222036469467	-2.098412313192
Cl	-0.464492011802	0.989694266001	-2.779658028718
Cl	2.876066740162	-0.249599735197	-3.746821635736
N	2.321259180621	-0.271916316724	-0.454510657387
C	3.583330355449	-0.558730952718	0.071188841491
C	4.731419138682	-0.234851615003	-0.658949426190
C	5.987246139247	-0.483813696530	-0.131988431860
C	3.727347282191	-1.113273129574	1.347924770197
C	4.989827261386	-1.364912347750	1.862018111966
C	6.127044764971	-1.055464795897	1.127427243799
H	6.867696748486	-0.224932210539	-0.712856536225
H	4.630113705558	0.221544714867	-1.638682042348
H	7.113925237336	-1.248786859431	1.535884064851
H	5.083153335537	-1.799324449462	2.853173603348
H	2.843890009795	-1.337633509654	1.939886180042
C	0.096190455064	0.016082837279	0.278082014457
C	1.130615341632	-0.823636108874	0.085333082194
C	0.952486221004	-2.286906389482	-0.212282012128
C	0.859119660786	-2.235098295297	-1.750047897286
C	1.643321183588	-3.297478006378	-2.495057720802
C	1.149563178342	-4.712589197832	-2.215378595856
H	1.597182726316	-3.113781257981	-3.577456818293
H	2.707140578970	-3.223459615854	-2.215864444790
C	1.943724869430	-5.769559136252	-2.965453231585
H	1.198703381273	-4.919675809962	-1.135458453543
H	0.086209042845	-4.785465293701	-2.492037542358
C	1.449835408180	-7.177274970026	-2.690526157625
H	1.519809826070	-7.418240547228	-1.622783383095
H	2.029246456103	-7.927962337803	-3.238013716059
H	0.399136195821	-7.290592150334	-2.984135594552
H	3.004824797790	-5.684614379233	-2.689972768692
H	1.893635011737	-5.559518859117	-4.043569000423
H	0.068689111180	-2.708397823689	0.278782524728
H	1.831314330118	-2.865290356375	0.094872911467
H	0.252453697537	1.089698645157	0.317322717719
H	-0.907786599534	-0.364650402304	0.444689546238
H	-0.201814466248	-2.218783437635	-2.059679203755

TS3'

Electronic energy -2369.292315 a.u.

Gibbs free energy -2369.073137 a.u.

37

Ti	3.960303593967	-2.098706086682	-1.876445876881
Cl	3.488857163157	-1.151639039367	-3.909112313073
Cl	6.220599183693	-1.973259320148	-1.598254950704
N	3.326161121298	-1.346181867286	-0.251973049829
C	3.766383143085	-0.163506262909	0.375542794490
C	3.565322833443	1.045089723779	-0.289913320392
C	4.054172950353	2.226203568187	0.248586762195
C	4.477536608644	-0.182322318477	1.577059327778
C	4.955601779078	1.004171342166	2.110223275655
C	4.745272710642	2.210823283335	1.452209319936
H	3.891442691136	3.162661643383	-0.276348847086
H	3.012327452089	1.044975100768	-1.225776775812
H	5.126278232850	3.135334853377	1.874886402312
H	5.509709256977	0.984242654323	3.044009831953
H	4.672232556827	-1.127564978131	2.074033427361
C	2.285218164355	-2.552581083644	1.642339824850
C	2.679615403140	-2.417444424360	0.374609492798
C	2.458450180578	-3.466339543626	-0.664027197892
C	1.852601245926	-3.074313474874	-1.841979587373
C	1.545623530786	-4.057100302888	-2.925665064783
C	0.300712858242	-4.887871044151	-2.615112894011
H	1.406019633824	-3.533350820596	-3.879594730053
H	2.405284532227	-4.734661127134	-3.059135938506
C	-0.022597237263	-5.894125720566	-3.705413688304
H	0.444179913327	-5.412775392779	-1.658857010307
H	-0.555474243308	-4.213066631202	-2.468220493097
C	-1.259005608862	-6.712891554107	-3.385656655138
H	-1.130789225673	-7.271341210981	-2.450434740418
H	-1.484967937500	-7.436638224991	-4.175327614859
H	-2.137784888302	-6.068031913082	-3.264712135370
H	0.840935820804	-6.559158937929	-3.850014418391
H	-0.160089679910	-5.362891800509	-4.657878849037
H	2.543669645456	-4.512496971245	-0.376253401836
H	4.117328135461	-3.657159784917	-1.155466246897
H	2.416689024080	-1.770838139061	2.381038464243
H	1.784097455423	-3.465364713610	1.945744927864
H	1.381429999950	-2.090880480060	-1.872092051386

VI. β-carbon elimination from 1b (Figure S60)

IM3

Electronic energy -2443.088799 a.u.

Gibbs free energy -2442.894014 a.u.

35

Ti	-0.211798911133	4.385716518366	0.131868453655
Cl	-0.801053670433	6.575875818949	0.677781429279
Cl	-0.925196676731	3.251508880182	1.937349219854

N	0.126791225306	3.000722207926	-1.118481089916
C	1.223823092561	2.189739522138	-1.457394617212
C	1.853561038874	2.289424859136	-2.701662302428
C	2.925773661858	1.463810113260	-3.006681485714
C	1.719093584808	1.286293174236	-0.514275531417
C	2.795210818719	0.468944268361	-0.824167958944
C	3.395723036365	0.546034959952	-2.074861501743
H	3.403459095928	1.543839495663	-3.978973368972
H	1.500864843041	3.022060056559	-3.421432403217
H	4.232496008571	-0.100951476209	-2.320089865580
H	3.159067387391	-0.241433629336	-0.087383118834
H	1.225269176703	1.212256990017	0.451046352710
C	-1.796185601618	4.229409011243	-1.185893444790
C	-1.150836987587	3.023553924281	-1.795914975223
C	-1.394809132838	2.571985139221	-3.201090358325
C	-1.965672862850	1.774394519309	-2.058381809883
H	-0.569670577268	2.123388400116	-3.745843822975
H	-2.071417278938	3.169156379425	-3.805908897929
H	-2.787099876714	4.095428738093	-0.746605232661
H	-1.754987609582	5.124462590444	-1.818410121043
C	-1.479298714302	0.416844554051	-1.702020897877
H	-3.026152378837	1.927262730937	-1.857535305283
C	-1.738474579365	-0.064842006332	-0.416606342460
C	-1.320487808918	-1.329231269874	-0.031438205662
C	-0.634475509309	-2.139588199712	-0.929408067673
C	-0.377275375678	-1.674600257248	-2.211830398400
C	-0.798225868335	-0.407249970911	-2.594722095286
H	-2.277361454878	0.568782527540	0.285625372462
H	-0.308370906559	-3.131779184866	-0.631437639067
H	-1.532220333314	-1.685910404434	0.972655248881
H	-0.597417204168	-0.057574025387	-3.604117216874
H	0.150536349229	-2.303584955097	-2.923308001455

TS2
Electronic energy -2443.067867 a.u.
Gibbs free energy -2442.871940 a.u.
35

Ti	0.956974907655	-1.756490127252	0.509241696108
Cl	0.933820446176	-2.918615045635	-1.517418852119
Cl	2.561957549346	-3.051445993543	1.558591402782
N	0.733467335945	0.112952613258	1.068690582646
C	0.856807504639	1.291719626079	0.307066539715
C	0.481285567833	2.526270951750	0.836402328202
C	0.655985365939	3.680227582571	0.089709389287
C	1.444165607023	1.237596573025	-0.957308730269
C	1.617364070490	2.396525265878	-1.698914863560
C	1.217685297332	3.621450490328	-1.181188221042
H	0.350626187130	4.636194797672	0.505434352396
H	0.053843506253	2.568426483387	1.833986689002
H	1.351471548201	4.528928873184	-1.761718346381
H	2.065805783445	2.339226517709	-2.686201439036
H	1.759233221249	0.276685441149	-1.361180854774
C	-0.118656812098	-1.593271332795	2.340992800818
C	-0.459675379502	-0.459007606719	1.499986250913
C	-1.257241714951	-1.247057367575	0.081560464275
C	-1.849087038786	-0.216055785672	1.009561566083
H	-1.675911182206	-2.251362093660	0.146935722833
H	-1.025573494784	-0.870092595448	-0.915116396706
H	-2.550758072264	-0.643373891582	1.727144802279
H	-0.917680595533	-2.296543368604	2.571149948493
H	0.588613542827	-1.385045713510	3.140461729029
C	-2.336206466290	1.107672170383	0.508539792829
C	-2.827845335895	2.015242498423	1.448073683628
C	-2.357429238137	1.459366506536	-0.836574164689
C	-3.302425766044	3.256498264039	1.055515313288
H	-2.827938985773	1.741313013514	2.501230700252
C	-2.834701767592	2.702503612331	-1.232723016348
H	-2.000240617897	0.765460499166	-1.592030451794
C	-3.303130945656	3.606253330650	-0.290065507069
H	-3.676937036575	3.952834972372	1.800154073776
H	-2.841245292968	2.962413345257	-2.287259948984
H	-3.675060698534	4.577564493331	-0.602354235862

IM4
Electronic energy -2443.092668 a.u.
Gibbs free energy -2442.896576 a.u.
35

Ti	-0.435598320389	4.184065703139	0.164308221324
Cl	-1.825050188151	3.275383472237	1.748778685566
Cl	1.209432064033	5.739966903453	-0.082405211104

N	0.064637423135	2.844785377392	-0.975693025499
C	1.213368345392	2.123289302555	-1.345131490476
C	1.901898301350	2.428937146861	-2.520582509551
C	3.025721521979	1.696654760826	-2.870952542721
C	1.689309403248	1.119356724298	-0.504019952486
C	2.818725299247	0.397076014619	-0.859303414149
C	3.482338275700	0.673952491911	-2.048121030770
H	3.554287857080	1.934408848268	-3.789485300050
H	1.555381342008	3.247104646733	-3.145300125209
H	4.360964977871	0.101257921938	-2.328846193116
H	3.177035391584	-0.393582872285	-0.206810773347
H	1.145717415200	0.899575659286	0.411314795516
C	-1.745386465327	4.331380537721	-1.400084879954
C	-1.142405660140	3.005842290013	-1.799834028930
C	-1.239753459818	2.514890168093	-3.209746848293
C	-2.004827519142	1.809671349137	-2.120960443235
H	-0.388798285770	1.991211754830	-3.632795719669
H	-1.782060536416	3.149153792919	-3.904244415626
H	-2.809495148080	4.319964161196	-1.160603477998
H	-1.470198509617	5.145714515933	-2.077931718290
C	-1.607126939027	0.445315280677	-1.675328674126
H	-3.073662927644	2.017187791437	-2.065693702777
C	-1.906045056759	0.042363089031	-0.372321819863
C	-1.547404376968	-1.218144879746	0.082412046306
C	-0.883582985346	-2.101442719630	-0.761325644453
C	-0.590413499174	-1.715804353326	-2.061981297190
C	-0.950151242125	-0.452613179090	-2.513957871170
H	-2.422897094975	0.733324370989	0.290632110523
H	-0.600469012268	-3.088292232133	-0.406858363205
H	-1.784888672205	-1.512797951745	1.100715956890
H	-0.716294324876	-0.163697023125	-3.535453768675
H	-0.079147393608	-2.401348864411	-2.731917574196

TS3
Electronic energy -2443.050602 a.u.
Gibbs free energy -2442.859858 a.u.
35

Ti	1.755585277362	0.953263125254	-0.387196225838
Cl	1.628769531307	1.817578138561	1.730519808745
Cl	4.008461624247	0.544790597525	-0.654253533529
N	1.142462104291	1.873060020297	-1.934650366631
C	1.250826712893	3.211685912797	-2.348402471844
C	2.448674131676	3.887864129942	-2.108249051044
C	2.572553448933	5.227882376016	-2.440724868931
C	0.178985077606	3.899003829649	-2.923166654878
C	0.316892255413	5.236010202616	-3.260556070362
C	1.510903684133	5.906944329155	-3.023625516558
H	3.510032424905	5.741247710603	-2.249273894149
H	3.284835177180	3.349580381146	-1.668528646040
H	1.611610309043	6.954225041457	-3.290875576475
H	-0.522137522585	5.760936320640	-3.707864951667
H	-0.762447642506	3.386261532030	-3.094655827340
C	0.016347742753	0.855091516407	-3.869400155257
C	0.368281097079	0.893809691607	-2.581245999197
C	-0.402007912398	0.300971895033	-0.347241848698
C	-0.015994956683	-0.170316560935	-1.597356856555
H	-0.703793688858	-0.402126360304	0.424688626607
H	-0.735399650518	1.333363374330	-0.243177095040
H	-0.575177547187	0.029812317999	-4.247781302725
H	0.285694205456	1.647906076298	-4.558002402227
C	-0.273847118747	-1.554432728009	-2.054592481550
H	1.705561430554	-0.548049389908	-1.212640925172
C	-1.446269129287	-2.189636479727	-1.648612273186
C	-1.738505271026	-3.472806377113	-2.091769325118
C	-0.861231014347	-4.130601475188	-2.940507544056
C	0.309473816473	-3.501325973946	-3.353072513061
C	0.599525591618	-2.220785498945	-2.917638532497
H	-2.140788912235	-1.668154814331	-0.996346289264
H	-1.086106812780	-5.136323264546	-3.282612578237
H	-2.656155333451	-3.957040008964	-1.772460597197
H	1.521620737445	-1.734137238204	-3.225645507082
H	1.001378132244	-4.016304349246	-4.012282553950

TS2’
Electronic energy -2443.080374 a.u.
Gibbs free energy -2442.882194 a.u.
35

Ti	1.101788105307	0.112835966857	-1.873383252451
Cl	1.774654919599	2.249803057632	-1.983419215616
Cl	1.044027088008	-0.268033308580	-4.074912799901

N	1.664913790676	-0.392068134879	-0.012390344195
C	2.636515452379	-1.252430649466	0.486249543433
C	3.850353072682	-1.357190862481	-0.212202249937
C	4.840908912957	-2.225215814941	0.225396984837
C	2.470505462498	-1.975892773390	1.676790608696
C	3.472815802981	-2.838056966463	2.111738240603
C	4.653252067674	-2.980766020590	1.384012195961
H	5.773534305278	-2.308172391120	-0.339563142633
H	3.999569787261	-0.748581946389	-1.113056585210
H	5.435083919415	-3.661384139197	1.731034364761
H	3.330922952759	-3.401440673655	3.038617851908
H	1.555076339923	-1.850346595293	2.263030691687
C	-0.372917875267	0.653975996845	-0.292645053154
C	0.312839456427	-0.514792060557	0.111828668871
C	-0.375598593957	-1.841777427624	-0.009527564437
C	-0.225074306098	-1.839205119114	-1.512817491204
H	-1.413502357152	-1.806644905857	0.351579085355
H	0.148686984157	-2.671706522491	0.482876615326
H	0.027227100607	1.619714809079	0.023859286060
H	-1.450726733083	0.588009275745	-0.482703170258
H	-1.128428144685	-1.510413888917	-2.052542718733
C	0.534972502810	-2.901040676812	-2.176118823783
C	1.580135100296	-3.611379741417	-1.553990256094
C	0.219294588929	-3.253042479446	-3.507658561131
C	0.909562206413	-4.251238791258	-4.174037317112
C	2.284847455163	-4.603046049329	-2.232683244400
C	1.957827765569	-4.928921588347	-3.544376144698
H	1.857053615121	-3.398217511517	-0.518288193970
H	-0.591648286316	-2.721959187344	-4.015264814190
H	0.631291834847	-4.505965518493	-5.200781474784
H	2.509742913964	-5.709007049057	-4.076067832044
H	3.096902792857	-5.126440312133	-1.718673887563

IM4'

Electronic energy -2443.104392 a.u.

Gibbs free energy -2442.906319 a.u.

35

Ti	0.167233724398	1.139529145667	-0.107931626749
Cl	1.153966010105	-0.681035912980	0.815051023072
Cl	0.287983072946	2.687610760303	1.507446038743
N	0.741878347171	2.136196020889	-1.647939228695
C	1.961971815205	2.687284703528	-2.040892215730
C	2.184291561196	3.381275335546	-3.233038094306
C	3.459003518915	3.848268140539	-3.532224461310
C	3.040039182753	2.479218360151	-1.169175425462
C	4.300760112200	2.955484009783	-1.473872730518
C	4.521288376582	3.646519892361	-2.660091029188
H	3.612670165183	4.380566120297	-4.466185564445
H	1.379307272494	3.537038044782	-3.942584275822
H	5.509903974684	4.023297686173	-2.900955306943
H	5.117998624643	2.780138459188	-0.779760502958
H	2.862799333443	1.932061504707	-0.241332672420
C	-0.955370304838	3.497034488376	-2.804905108326
C	-0.512799480482	2.357852160172	-2.267423891155
C	-1.419341255323	1.153429993389	-2.081607963057
C	-1.818734533290	0.999236392232	-0.599171186474
H	-0.882357957407	0.272094280810	-2.402724396122
H	-2.317958820518	1.226100212833	-2.705577257222
H	-1.965554283067	3.529719356122	-3.202753004815
H	-0.361304800172	4.401138160283	-2.869257194919
C	-2.467711169502	-0.255558656506	-0.147968124743
H	-2.307151447022	1.910811224699	-0.230881220805
C	-3.005550439630	-0.315433178468	1.149283161852
C	-3.577177945140	-1.479989644163	1.630588332853
C	-3.632362917541	-2.615584317178	0.828245959160
C	-3.110696202976	-2.568885416073	-0.455853867222
C	-2.530465720086	-1.406135388314	-0.939186978659
H	-2.959956606021	0.570948149099	1.779493758308
H	-4.082672918634	-3.530489200062	1.201354151230
H	-3.982739983557	-1.503083969862	2.638110080573
H	-2.137098319456	-1.394464216862	-1.951016919012
H	-3.157179987256	-3.447212701461	-1.092692258713

TS3'

Electronic energy -2443.05884 a.u.

Gibbs free energy -2442.868779 a.u.

35

Ti	0.438766923556	0.572062078496	-0.605140384308
Cl	2.057484670212	-0.960469465667	-0.631817385681
Cl	0.469405666600	1.072408953587	1.647708493370
N	0.669497657219	2.154995599775	-1.609995959533
C	1.886901321079	2.789897824035	-1.931549607650
C	2.232980174407	3.160841208437	-3.232058256561
C	3.479275974069	3.712332255995	-3.481418763045
C	2.808087929062	2.974452878539	-0.897397508362
C	4.053773682501	3.516967291854	-1.162065945138
C	4.393058503560	3.895872858488	-2.453023394189
H	3.741456713133	3.992681588821	-4.496947882817
H	1.549208123437	2.971327910577	-4.051296489736
H	5.365099961872	4.332288215095	-2.657678978022
H	4.757643046191	3.656537903271	-0.348091438585
H	2.521455674550	2.726312170813	0.122200903458
C	-0.851940084093	3.282347232597	-3.198151931829
C	-0.544134330664	2.349541383962	-2.291413133061
C	-1.542193424866	1.340475025363	-1.875593789117
C	-1.748618891221	1.014383299122	-0.538742755217
H	-0.241949198838	-0.130355184322	-2.079706634249
H	-2.140633687665	0.893256140605	-2.670508301486
H	-1.839107785977	3.274400671390	-3.646178161005
H	-0.173957555643	4.078042654401	-3.477773025045
C	-2.489538538836	-0.150168581538	-0.071704619608
H	-1.499174595888	1.740722747073	0.233744086531
C	-2.823329975604	-0.252344645579	1.286943949606
C	-3.502520866243	-1.357115732016	1.763206752169
C	-3.850245161932	-2.389833880354	0.899541759023
C	-3.502776066453	-2.315838415848	-0.441325109410
C	-2.818302573150	-1.212625284426	-0.924806781919
H	-2.543251572956	0.552593016834	1.961060030376
H	-4.378936946923	-3.259359084306	1.276713818722
H	-3.758876957964	-1.418644085683	2.815837608604
H	-2.521204033589	-1.185811587957	-1.969046667532
H	-3.753733772941	-3.129784961436	-1.113794498750

VII. $Ti(NMe_2)_4$ Catalyst (Figure S61)

IM2

Electronic energy -1791.590705 a.u.

Gibbs free energy -1791.251709 a.u.

51

Ti	1.285158596017	3.778387029558	-1.617275717813
N	2.748868234390	2.866753741014	-1.377400978144
C	3.828032577372	2.043389478156	-1.449744385443
C	4.090963859607	1.265749508927	-2.598107840124
C	5.162865123451	0.390335771141	-2.641897304074
C	4.702400594624	1.891131202343	-0.353595335354
C	5.774558974335	1.018328567871	-0.408376091237
C	6.012793134726	0.259688486802	-1.549546661040
H	5.341978234356	-0.194141886421	-3.540657106269
H	3.431337907372	1.376418845321	-3.455961013487
H	6.854304133753	-0.425138747474	-1.587465339547
H	6.433945358824	0.924173898547	0.450421387345
H	4.513985321852	2.485931729733	0.536950611090
C	-0.566920184454	2.321135645052	-1.444526810519
C	0.353991185221	1.522350542401	-0.878522770327
C	0.866209231575	0.974654941490	0.364655282054
C	1.066786431056	0.249457982484	-0.975620028965
H	0.136619096107	0.572087384988	1.066258630604
H	1.759859351229	1.405831374824	0.807276570083
H	-1.148464620303	3.011003138501	-0.831891914697
H	-0.882923446383	2.170530555199	-2.475355014068
C	0.346877185666	-1.000576624095	-1.324171240501
H	2.094900365272	0.288458707126	-1.333690824500
C	-1.002975806944	-1.192005265864	-1.028739939115
C	-1.635320854266	-2.380249517516	-1.359396468379
C	-0.932566780852	-3.394773138127	-1.996499441350
C	0.408485017081	-3.211200874070	-2.300670792511
C	1.042085903094	-2.023043615712	-1.967422888577
H	-1.568462592897	-0.401716589489	-0.540267158644
H	-1.430508139596	-4.323832803342	-2.256601835061
H	-2.686468746411	-2.514574573101	-1.121433765706
H	2.094280868650	-1.879325178521	-2.203789421574
H	0.966453319086	-3.997721452889	-2.800136408124
N	1.061317234559	4.967326679903	-0.148576849857
N	1.001386190928	4.457486460850	-3.404383466301
C	1.791048351934	5.143671869747	1.077651288024
C	-0.120872308883	5.792584421670	-0.212941630403
C	0.668909078250	5.785536580293	-3.846603312020
C	1.287521356007	3.581436689607	-4.514346340526

H 1.177166428583 4.881513708722 1.957733232127
H 2.116019513193 6.189349931317 1.216374422147
H 2.680680029834 4.508459112304 1.081380471848
H -0.814935703452 5.589232524536 0.621800102058
H 0.116656545108 6.869965425043 -0.186035682671
H -0.673435927651 5.608855513752 -1.147202756680
H -0.249367670573 5.798472749137 -4.460646077345
H 0.511674561863 6.459151236303 -2.996291227539
H 1.471099853850 6.223805153543 -4.466019177662
H 0.433239238985 3.519087213478 -5.212067558895
H 1.495287534875 2.560174026507 -4.167499935682
H 2.161716859981 3.915392438431 -5.100353486651

TS1

Electronic energy -1791.58513 a.u.

Gibbs free energy -1791.246138 a.u.

51

Ti 1.366251280400 2.795122312815 -2.681547016552
N 2.349116005040 2.369142078393 -1.262104841020
C 2.929409094830 2.401613959814 -0.020572235042
C 4.270648161024 2.015519838848 0.138213444575
C 4.879333440242 2.053688929435 1.382036019829
C 2.220216634361 2.805294952026 1.124686009113
C 2.830821932345 2.829544354437 2.366385129852
C 4.163722961352 2.457407914641 2.502317623193
H 5.920494973246 1.759493180264 1.480617501579
H 4.821350584601 1.698710102566 -0.743928411373
H 4.641117308771 2.481683639389 3.477043247735
H 2.264228786373 3.143726035676 3.238561956099
H 1.175943132219 3.088613940662 1.014128898510
C -0.144851662529 1.301893261220 -2.213918265498
C 0.932699920782 0.826495079778 -1.455525277688
C 1.118706260962 0.007260101080 -0.252791182568
C 1.745463657653 -0.402502805353 -1.573230371219
H 0.225654381998 -0.511863169039 0.088243759888
H 1.799496159389 0.311832828720 0.536918824416
H -1.013866738775 1.715811857512 -1.702737869732
H -0.332141239955 0.824557977866 -3.178256031655
C 1.239782963091 -1.515285458181 -2.419457522481
H 2.822004127456 -0.235259780678 -1.612967229529
C 0.571390122193 -2.613116821402 -1.889225703065
C 0.106237880583 -3.624805627224 -2.719947980842
C 0.302281857951 -3.548520479365 -4.090803291651
C 0.967385733088 -2.454213218583 -4.630491858520
C 1.431116401928 -1.446344301300 -3.800249268066
H 0.421250441154 -2.682059626716 -0.814915305110
H -0.062043285086 -4.339699410785 -4.739170594709
H -0.410221443153 -4.478967301361 -2.291716939567
H 1.943247547909 -0.579287977878 -4.214461525961
H 1.123027707628 -2.386469414859 -5.703366785496
N 2.261499653462 2.255212720339 -4.271752616476
N 0.568521895526 4.538675067055 -2.729500383724
C 3.590495102982 1.766667848416 -4.522494320894
H 4.150944292951 2.434481501659 -5.198702750374
H 3.580710427224 0.769973262617 -4.999685955512
H 4.149802787955 1.688529990646 -3.585582086142
C 1.461062731776 2.344449130617 -5.469706149240
H 0.456586825718 2.734061240577 -5.242608106588
H 1.323375319770 1.359887808337 -5.950681786645
H 1.907119660877 3.017823131970 -6.221669062803
C 0.497235231567 5.484563261729 -3.812274889073
H 0.967120029772 5.086293226105 -4.718352975375
H 1.012098786521 6.428872361170 -3.563370570653
H -0.546361046429 5.746471788974 -4.061172935767
C 0.006898328539 5.075950190968 -1.513855978810
H -1.062154078126 5.324819766850 -1.633501526163
H 0.521804857075 5.997103952301 -1.190245688716
H 0.082864103768 4.353876797253 -0.690589124487

IM3

Electronic energy -1791.612431 a.u.

Gibbs free energy -1791.267285 a.u.

51

Ti 1.450883722067 3.707431274760 -1.414253466418
N 2.469679040366 2.205278813577 -0.745540557631
C 3.516918422219 1.682927671945 -1.503427520971
C 3.414847226245 0.552858627756 -2.328748834235
C 4.493930014357 0.147709876888 -3.101430720402
C 4.737839796032 2.375889808608 -1.483016372878
C 5.802946532335 1.968347201693 -2.265414470932
C 5.691770136390 0.848492669128 -3.082243909527
H 4.387863649781 -0.726134281035 -3.738855084294
H 2.477193175847 0.008790369759 -2.384719432910
H 6.528914124565 0.526317283310 -3.693347499492
H 6.733855308199 2.527839936052 -2.233634570220
H 4.824561447307 3.242811847297 -0.832194977204
C 0.261936520962 2.010803781044 -1.768842365692
C 1.177056229550 1.484191209535 -0.695463999122
C 0.648517097952 1.206720883074 0.676469911071
C 1.195183190710 0.064186443766 -0.122323848876
H -0.427873220337 1.275075213558 0.815275509345
H 1.246213281187 1.539182230706 1.519697123927
H -0.797353419498 2.042876139413 -1.508997722863
H 0.422602822394 1.548373420217 -2.750187784931
C 0.377605623437 -1.018934828012 -0.708222616574
H 2.193599740201 -0.251924412130 0.174337221419
C -1.003826725646 -0.925627947469 -0.883576147729
C -1.719386177502 -1.964872210440 -1.459398890769
C -1.072215950112 -3.121466824585 -1.871687973276
C 0.300546010033 -3.230633691089 -1.699813192370
C 1.014738826136 -2.190190145133 -1.125474607555
H -1.532484261925 -0.033798708728 -0.560783351984
H -1.635372715081 -3.934009467307 -2.320636035554
H -2.794355060683 -1.870728793101 -1.584176050545
H 2.090431340025 -2.281739308365 -0.990160701794
H 0.819429649594 -4.132265560414 -2.012298725355
N 0.523805270301 4.709013725902 -0.112860183271
N 1.865773585656 4.616238035829 -3.024572032120
C 0.710998444632 4.745997321697 1.314543985598
C -0.688554769901 5.358805766615 -0.551414073524
C 1.528281867247 5.969622825807 -3.392755236274
C 2.558936960948 3.925295830924 -4.090090844414
H -0.129293838243 4.271024632883 1.848610260955
H 0.787678251836 5.781902031319 1.683339748002
H 1.630524505096 4.222629116510 1.596841127005
H -1.577878622601 4.919262739971 -0.069866845750
H -0.685343660671 6.438663135348 -0.327966260013
H -0.819054192871 5.242913339538 -1.636715505731
H 0.846559164590 5.998981463633 -4.259409555920
H 1.046191763689 6.496939325924 -2.562841043797
H 2.427677773594 6.543999564308 -3.669397234699
H 1.947360241253 3.889932320745 -5.006484730281
H 2.798743098652 2.891024592723 -3.813748225915
H 3.509638759685 4.422995706047 -4.343331683508

TS2

Electronic energy -1791.579842 a.u.

Gibbs free energy -1791.239185 a.u.

51

Ti 0.111892824558 2.435167542049 -1.218315300102
N 1.970361464755 1.358774297063 -1.344062946931
C 2.756303514821 0.977433306148 -0.263902648032
C 3.535581735828 -0.188732752121 -0.247927640216
C 4.338750809082 -0.482014100225 0.843670267999
C 2.840278204944 1.845883535526 0.834336666651
C 3.645832197439 1.545475645456 1.921627575092
C 4.395820277785 0.375383727560 1.937177482224
H 4.935495171728 -1.390290035485 0.836709038453
H 3.520571846771 -0.851431392424 -1.110024491141
H 5.028403528490 0.139048891993 2.787279964115
H 3.690638311822 2.232701303274 2.762098079186
H 2.266434750721 2.772622124474 0.797497871682
C 0.390567257014 1.265056471005 -3.019770628359
C 1.064285592114 0.543819901319 -1.947001874507
C -0.395684667093 0.213678008281 -0.699035405656
C 0.556711420537 -0.738332327128 -1.387934499747
H -1.433756160681 0.165102441414 -1.034203147784
H -0.261103350673 0.286970773446 0.385075625440
H -0.451497355357 0.757756406628 -3.492798204450
H 1.071448134066 1.765224973709 -3.708704755741
C 0.037152106264 -1.767397244722 -2.344858986094
H 1.267962472338 -1.189518314945 -0.690548245446
C -1.202164136135 -2.373559392188 -2.162227979953
C -1.644257353383 -3.361477569454 -3.034220773731
C -0.852669118458 -3.753632109386 -4.104428045458
C 0.387110792030 -3.154295393123 -4.296125363247
C 0.826349277694 -2.170700641825 -3.422892269561
H -1.826592755479 -2.071988934951 -1.324888748733
H -1.199869540531 -4.521967131585 -4.789049033866
H -2.614062154311 -3.825134969631 -2.876451118361

H	1.795595139630	-1.699938781630	-3.577885220920
H	1.012816833627	-3.453500484406	-5.132275944242
N	1.020194659423	4.126869050317	-1.565019720788
N	-1.432748265364	2.979644358583	-0.241620839555
C	2.343783841792	4.321327158413	-2.111621548365
H	3.026763303628	4.773185452980	-1.368642473985
H	2.323109051883	5.008328214072	-2.975333119180
H	2.778878142076	3.372094304049	-2.433015091984
C	0.493584705353	5.408208459133	-1.137526770613
H	1.126373037000	5.863878020673	-0.354003930267
H	-0.520490952044	5.307597401417	-0.742158846932
H	0.464301891172	6.130128483605	-1.972099588002
C	-1.406223837194	3.262748679523	1.164997393128
H	-0.368834231410	3.360616883233	1.516798063075
H	-1.888528717836	2.466464703247	1.759943577783
H	-1.921446481869	4.208360707107	1.406344753120
C	-2.749380041111	2.784235469609	-0.784664083787
H	-2.691061973316	2.520950607266	-1.850755163554
H	-3.365360873909	3.695506695990	-0.707373544640
H	-3.294740330228	1.972147576662	-0.270988364021

IM4

Electronic energy -1791.604287 a.u.

Gibbs free energy -1791.282619 a.u.

51

Ti	0.218797955703	3.927146260612	0.043258762819
N	-0.627802791236	2.219526463152	0.464013876216
C	0.068991036722	1.196087914881	1.109687615104
C	0.966386737860	1.531605512324	2.130303803883
C	1.794839919992	0.572114877546	2.694522975487
C	0.002898700284	-0.140220799964	0.691884296711
C	0.818280840258	-1.093401476963	1.274840124898
C	1.722729894770	-0.747099326354	2.274854256180
H	2.487545217594	0.858295026484	3.480678231250
H	0.999879307009	2.561484106867	2.484016370864
H	2.360526532956	-1.502296880515	2.723142065523
H	0.757562406530	-2.123595914228	0.935008055267
H	-0.683869679731	-0.412392379553	-0.104979505751
C	-2.900529978903	1.368611452934	0.533924862846
C	-1.922306074122	2.054709337961	-0.061984990913
C	-1.430852464985	4.157162831376	-1.290238788439
C	-2.078999073459	2.769561187241	-1.393562945069
H	-2.748738317713	0.859597497799	1.481232359536
H	-3.880292764538	1.294285185894	0.073670897475
C	-1.482490638804	1.884887976203	-2.476373665786
H	-3.156838592591	2.848590999198	-1.606160591589
C	-2.172042177616	0.735615732571	-2.867119958094
C	-1.654016119697	-0.122979453176	-3.822672222189
C	-0.423646589817	0.148584871163	-4.409873522946
C	0.270656680481	1.287120662984	-4.034733733188
C	-0.257857620216	2.150651313878	-3.081070723835
H	-3.130929372086	0.511922938135	-2.403956601465
H	-0.012923502404	-0.523538289654	-5.157477230982
H	-2.211977162489	-1.008725114715	-4.112972953263
H	0.300154445195	3.038618922867	-2.794774339635
H	1.231408571449	1.513402685718	-4.489561681612
N	-0.189805591580	5.293248106644	1.278005047459
N	1.907406349521	3.988535396032	-0.824168661392
C	-1.093891899615	5.153654403435	2.394359473975
H	-2.104236776836	5.516005535030	2.135027488100
H	-0.752920848291	5.731990496752	3.267884005443
H	-1.186038024002	4.105719067349	2.698606318540
C	-0.071591952399	6.657085844451	0.822435666758
H	0.492987590522	7.288227433125	1.526515894089
H	-1.065984388900	7.114722576956	0.683392941140
H	0.437392801539	6.706269943871	-0.150186633599
C	2.770523461953	5.118122972397	-1.067115289479
H	3.017796060678	5.210812051628	-2.137159285542
H	3.722959425763	5.024105630837	-0.520047584388
H	2.303487674484	6.056665292335	-0.752835182587
C	2.556243457358	2.742234464907	-1.178092856458
H	3.477928588054	2.589327076226	-0.593271709693
H	2.834769547376	2.724147233055	-2.244908858230
H	1.902931170893	1.879504644372	-0.998193925674
H	-2.112490124326	4.821300209159	-0.735647031677
H	-1.262681848589	4.605687498775	-2.276504916092

TS3

Electronic energy -1791.556102 a.u.

Gibbs free energy -1791.219518 a.u.

51

Ti	1.678722484393	0.952007155207	-0.134169541998
N	0.983395654116	1.872223275301	-1.860262970268
C	1.087057114441	3.199073753338	-2.284348429282
C	2.346898020145	3.802722644238	-2.347022241067
C	2.479880737223	5.146552890059	-2.658953497883
C	-0.035577940040	3.986058198396	-2.569152125466
C	0.103020829620	5.327901267355	-2.887987931523
C	1.359030700509	5.920192080927	-2.931546804882
H	3.470785572765	5.591220029484	-2.698404122744
H	3.221915982538	3.192084075467	-2.140948906554
H	1.462780331332	6.972181602720	-3.178753863025
H	-0.783594870181	5.921111198119	-3.095509661880
H	-1.023650513248	3.535629264509	-2.518615044215
C	-0.362837271744	0.991131888277	-3.732473712221
C	0.187308820457	0.953811949720	-2.506390315860
C	-0.396365562730	0.137000528772	-0.285125941945
C	-0.038638459278	-0.204772277489	-1.589223441791
H	-0.657505053091	-0.635350926930	0.432602738810
H	-0.812734797810	1.137284523635	-0.126201440881
H	-0.944072851567	0.154510373860	-4.101430322635
H	-0.257701024129	1.857870887554	-4.376114474190
C	-0.162673647046	-1.580117200417	-2.113554512102
H	1.850797625038	-0.299213185950	-1.375851867787
C	-1.137526662744	-2.437286918072	-1.603810050547
C	-1.281896032320	-3.722969831614	-2.106027210763
C	-0.452142701831	-4.168367963802	-3.122888838512
C	0.521545243431	-3.321182453774	-3.641129402403
C	0.664108144868	-2.038965863911	-3.143052626166
H	-1.806037871273	-2.084330316068	-0.824367346933
H	-0.561357820729	-5.175291733199	-3.514290979835
H	-2.050055732865	-4.375817464252	-1.703078821083
H	1.438022729992	-1.382435801069	-3.530323365533
H	1.177560533839	-3.667337699800	-4.433890977264
N	2.493861453018	2.477552313010	0.645806302532
N	2.419759654002	-0.432835714999	1.013069400850
C	1.580497977027	3.407995383234	1.250266467845
C	3.870376619410	2.871897256997	0.713144889891
C	2.919052601979	-0.171052848229	2.346522456412
C	2.425340189575	-1.858094126425	0.776741499079
H	0.546866953224	3.026424945333	1.193006533208
H	1.804483098954	3.576450651077	2.317867569296
H	1.582712077433	4.388636462126	0.743156274052
H	4.205228512467	2.996944034218	1.757957770010
H	4.055003980590	3.829166064697	0.195931395074
H	4.508552308553	2.107658091734	0.251750425418
H	3.960545039967	-0.519230352884	2.466886988775
H	2.888822132874	0.896642415149	2.581630973023
H	2.323584960205	-0.701284602627	3.111147547972
H	3.432701175351	-2.288261294543	0.915856202144
H	1.759599388702	-2.388043054306	1.482445334962
H	2.098450164586	-2.097583574152	-0.239029980115

TS2'

Electronic energy -1791.579843 a.u.

Gibbs free energy -1791.246138 a.u.

51

Ti	0.979628586325	0.126911708842	-1.894020728480
N	1.629276564227	-0.192850046909	-0.054800449214
C	2.615483288100	-0.990710772414	0.529997846515
C	3.794840890758	-1.310229026678	-0.154191917927
C	4.764889918535	-2.098307275238	0.442410718216
C	2.449533463214	-1.488581681679	1.829027277031
C	3.418515090194	-2.289773718756	2.411643105439
C	4.585843691426	-2.598669167186	1.726338036139
H	5.666778376411	-2.340139590766	-0.113369597339
H	3.908156297599	-0.977995459651	-1.180848764870
H	5.345381766269	-3.223515587314	2.185576654028
H	3.261526573808	-2.669861845161	3.417370905458
H	1.546803568379	-1.234717966752	2.379725967558
C	-0.579852074384	0.545131137945	0.415165074735
C	0.271598627984	-0.477618198591	0.231650773114
C	-0.203046988823	-1.833012695169	-0.240742586581
C	-0.049916246727	-1.787703604389	-1.779236212723
H	-1.241336287593	-2.010932805558	0.063726274387
H	0.406843801488	-2.632641753753	0.192395091907
H	-0.215037452623	1.545350424193	0.626931186402
H	-1.654582689110	0.390626466890	0.390773629919
H	-1.023035115991	-1.580107665393	-2.248976445353

C	0.648541236697	-2.881243950965	-2.481525263545
C	1.731229090116	-3.573204115052	-1.921668046873
C	0.297770773270	-3.204605773177	-3.801100655264
C	1.000170782875	-4.150452203174	-4.529447934147
C	2.434444959796	-4.519661798846	-2.650925048293
C	2.079766553726	-4.814595251140	-3.960722169549
H	2.049034123563	-3.352138363626	-0.905267266550
H	-0.550366671707	-2.696228340108	-4.258971561529
H	0.698482461129	-4.375821684316	-5.548936817594
H	2.633689345460	-5.555248301154	-4.529332261053
H	3.274689476035	-5.029583317882	-2.186831851734
N	-0.106404247875	1.565160379812	-2.558932911087
N	2.582035554314	0.223256444014	-2.901091630698
C	0.052798446373	2.894383023838	-2.035936388775
H	-0.896399565383	3.319684334860	-1.662504125807
H	0.445096356497	3.587667606406	-2.800567796814
H	0.764353699040	2.905350607332	-1.199013176429
C	-0.921462158766	1.512593391258	-3.742542908531
H	-0.506631718978	2.119904563885	-4.566189707368
H	-1.942444259490	1.885260773951	-3.549422248421
H	-1.025549774273	0.483478680744	-4.109981595972
C	3.739009107854	1.059686098817	-2.737093506823
H	3.880567963070	1.319924724510	-1.681936049595
H	3.643204271239	1.999965772362	-3.308780714589
H	4.660149086138	0.562292816885	-3.084417192676
C	2.272684024927	-0.068666592468	-4.279292497458
H	2.961463870812	-0.813253617380	-4.710792760229
H	2.287285032558	0.837624316203	-4.908989465610
H	1.265658531514	-0.511121102100	-4.371772285350

IM4’

Electronic energy -1791.638728 a.u.
Gibbs free energy -1791.293145 a.u.
51

Ti	-0.866290896891	1.123552785297	0.321163871786
N	-0.110057386521	2.363270637510	-1.011170151776
C	1.280378771482	2.624634350825	-0.930347066439
C	2.185020951492	1.967985749925	-1.768004602138
C	3.545336361241	2.198383419909	-1.647354192902
C	1.767224130003	3.512421845916	0.025487052958
C	3.132877186359	3.739634936020	0.146100701836
C	4.025894160573	3.086168609938	-0.689722257054
H	4.239336800003	1.679038913097	-2.302486500574
H	1.798706302897	1.271718837805	-2.509265761018
H	5.092976382214	3.264541934776	-0.597470998907
H	3.498746565520	4.434805702625	0.896464017246
H	1.057082936296	4.022169992249	0.672563605618
C	-0.273964775164	3.540649502291	-3.142185016361
C	-0.792333401973	2.814310094085	-2.140092139090
C	-2.253333488776	2.416408476434	-2.123535446430
C	-2.534527460357	1.367542198956	-1.086872010780
H	-2.563750264442	2.123398897069	-3.137567488516
H	-2.845022657650	3.308871870614	-1.883345739827
H	-0.906165806774	3.824988279847	-3.977597255996
H	0.753730390408	3.885892520724	-3.150998971431
C	-2.263335501612	-0.020789097149	-1.275867932137
H	-3.348622707059	1.578143712902	-0.388932493107
C	-2.386725851063	-0.882552647714	-0.154734028817
C	-1.995610937928	-2.224203525850	-0.217606572047
C	-1.438530528824	-2.732039933548	-1.370172492411
C	-1.251812617724	-1.882112601587	-2.469853102424
C	-1.620234786780	-0.558143436319	-2.423137974451
H	-2.930217898446	-0.523580553599	0.723217680869
H	-1.138229260665	-3.773102256325	-1.427053067540
H	-2.145160615119	-2.863300221377	0.647837348647
H	-1.457228352735	0.082956971660	-3.285579797561
H	-0.800320936274	-2.275642212272	-3.376731527827
N	-1.506353828522	2.041622122390	1.844058065922
N	0.688968430859	0.078753693277	0.724072885765
C	-1.956583534614	3.409042402654	1.908095189012
H	-1.412678645730	3.979239675159	2.680178414241
H	-3.030255453633	3.470993203569	2.154603852423
H	-1.803008704541	3.913041274265	0.948430531726
C	-1.690494279727	1.325554388203	3.083874035514
H	-1.352664399242	0.284604819379	2.989973002347
H	-2.750248621612	1.305146588643	3.388187526417
H	-1.119692280493	1.782049361118	3.910395187738
C	1.484402956550	0.445567446322	1.876164577112
H	1.009522142669	1.246065785886	2.453496056981
H	2.477528661894	0.813319205449	1.565464553173
H	1.647506534485	-0.412251163146	2.551255976690
C	1.354322346500	-0.926523145405	-0.061079299646
H	1.478431560956	-1.870513592875	0.497739060805
H	2.362781868625	-0.589332599200	-0.357935035836
H	0.795150439865	-1.151683220422	-0.974254271785

TS3’

Electronic energy -1791.533676 a.u.
Gibbs free energy -1791.153854 a.u.
51

Ti	-0.975995856300	3.433740278927	-0.169431440646
N	0.281557780321	2.275749213307	-1.270980475817
C	1.667077018909	2.330508702754	-1.163054525311
C	2.523540234817	2.628704246038	-2.229507434440
C	3.895887269149	2.655139048413	-2.038803829497
C	2.236980610568	2.021256464336	0.079098145430
C	3.607622008771	2.054979682136	0.260700509032
C	4.449409743200	2.382003525608	-0.795132582126
H	4.543128783433	2.889763502188	-2.879120531137
H	2.104581870649	2.828005577461	-3.210954174279
H	5.524764138457	2.409549754850	-0.653654847804
H	4.024554620278	1.816567749821	1.234826376591
H	1.576420781980	1.741747838953	0.897798208027
C	-0.175404860517	4.328825802473	-2.516088248293
C	-0.447129189839	3.025558687495	-2.164457122248
C	-1.925497372094	2.661721651410	-2.289236580130
C	-2.354116526760	1.316954646826	-2.542566355284
H	-2.335707087708	3.306561027621	-3.070422525377
H	-2.468316434074	3.281691075825	-1.338347079325
C	-2.171439056116	0.049133364077	-1.915582449631
H	-3.057592681519	1.264803423654	-3.373224950471
C	-1.399201048548	-0.237438396807	-0.767388742220
C	-1.321957100485	-1.520826063015	-0.259764348406
C	-2.000385634374	-2.574345516323	-0.860252744176
C	-2.770141322335	-2.322080022735	-1.991593265033
C	-2.854499458954	-1.044876975827	-2.502306159763
H	-0.849561007805	0.559349574395	-0.288969988792
H	-1.931577531066	-3.578121520836	-0.453265730031
H	-0.713991391351	-1.702803816673	0.622572837633
H	-3.462947767921	-0.860739245301	-3.384666982907
H	-3.309435737146	-3.131166713548	-2.475326459410
H	-0.898876668054	4.909294127590	-3.076852531524
H	0.823243845508	4.745120590555	-2.431025013695
N	-0.242628511002	4.870657063268	0.831281450715
N	-2.166748793947	2.480445596277	0.951548943755
C	1.058027377553	5.467277487071	0.618884414112
H	0.976490296420	6.536755766365	0.365860657880
H	1.606439498457	4.967772875301	-0.187485268182
H	1.678429863204	5.388258842405	1.526922359802
C	-0.915293469276	5.460923117717	1.968212188185
H	-1.898753139567	5.005741341556	2.118075620962
H	-1.061776098927	6.543477627488	1.828005423260
H	-0.328178597502	5.328721140803	2.893217920730
C	-1.448002954115	2.034471354569	2.128006112894
H	-1.941800212558	2.366365032912	3.056166881591
H	-0.429298149107	2.452944742131	2.147521011572
H	-1.359232746444	0.935588178669	2.163641841882
C	-3.488751959363	1.918516701632	0.869168861135
H	-4.103441848386	2.214770969067	1.734579475825
H	-3.455845496403	0.815442032038	0.844709078010
H	-3.997780032116	2.258708845083	-0.037465933068

References

1 A. J. Blake, P. E. Collier, S. C. Dunn, W.-S. Li, P. Mountford and O. V Shishkin, *J. Chem. Soc., Dalt. Trans.*, 1997, 1549–1558.

2 Z. W. Davis-Gilbert, L. J. Yao and I. A. Tonks, *J. Am. Chem. Soc.*, 2016, **138**, 14570–14573.

3 Z. W. Davis-Gilbert, X. Wen, J. D. Goodpaster and I. A. Tonks, *J. Am. Chem. Soc.*, 2018, **140**, 7267–7281.

4 K. Kitatani, T. Hiyama and H. Nozaki, *Bull. Chem. Soc. Jpn.*, 1997, **50**, 3288–3294.

5 J. E. Baldwin and R. E. Peavy, *J. Org. Chem.*, 1971, **36**, 1441–1443.

6 L. Fitjer and U. Quabeck, *Synthesis*, 1987, 299–300.

7 G. M. Whitesides and W. J. Ehmann, *J. Am Chem. Soc.*, 1970, **92**, 5625–5640.

8 G. Fournet, G. Balme and J. Gore, *Tetrahedron*, 1988, **44**, 5809–5820.

9 N. Hayashi, Y. Hirokawa, I. Shibata, M. Yasuda and A. Baba, *J. Am. Chem. Soc.*, 2008, **130**, 2912–2913.

10 E. P. Siqueira Filho, J. A. R. Rodrigues and P. J. S. Moran, *Tetrahedron Asymmetry*, 2001, **12**, 847–852.

11 C. Cao, Y. Li, Y. Shi and A. L. Odom, *Chem. Commun.*, 2004, 2002–2003.

12 H.-J. Knölker, N. Foitzik, H. Goesmann, R. Graf, P. G. Jones and G. Wanzl, *Chem. Eur. J.*, 1997, **3**, 538–551.

13 F. Huet, M. Pellet and J. M. Conia, *Tetrahedron Lett.*, 1977, **18**, 3505–3508.

14 H. B. Kwon, B. H. McKee and J. K. Stille, *J. Org. Chem.*, 1990, **55**, 3114–3118.

15 B. T. Cho and S. K. Kang, *Tetrahedron*, 2005, **61**, 5725–5734.

16 M. J. Frisch, G. W. Trucks, H. B. Schlegel, G. E. Scuseria, M. A. Robb, J. R. Cheeseman, G. Scalmani, V. Barone, G. A. Petersson, H. Nakatsuji, X. Li, M. Caricato, A. V. Marenich, J. Bloino, B. G. Janesko, R. Gomperts, B. Mennucci, H. P. Hratchian, J. V. Ortiz, A. F. Izmaylov, J. L. Sonnenberg, D. Williams-Young, F. Ding, F. Lipparini, F. Egidi, J. Goings, B. Peng, A. Petrone, T. Henderson, D. Ranasinghe, V. G. Zakrzewski, J. Gao, N. Rega, G. Zheng, W. Liang, M. Hada, M. Ehara, K. Toyota, R. Fukuda, J. Hasegawa, M. Ishida, T. Nakajima, Y. Honda, O. Kitao, H. Nakai, T. Vreven, K. Throssell, J. A. Montgomery, J. E. Peralta, F. Ogliaro, M. J. Bearpark, J. J. Heyd, E. N. Brothers, K. N. Kudin, V. N. Staroverov, T. A. Keith, R. Kobayashi, J. Normand, K. Raghavachari, A. P. Rendell, J. C. Burant, S. S. Iyengar, J. Tomasi, M. Cossi, J. M. Millam, M. Klene, C. Adamo, R. Cammi, J. W. Ochterski, R. L. Martin, K. Morokuma, O. Farkas, J. B. Foresman and D. J. Fox, 2016.

17 Y. Zhao and D. G. Truhlar, *Theor. Chem. Acc.*, 2008, **120**, 215–241.

18 R. Krishnan, J. S. Binkley, R. Seeger and J. A. Pople, *J. Chem. Phys.*, 1980, **72**, 650–654.

19 A. V Marenich, C. J. Cramer and D. G. Truhlar, *J. Phys. Chem. B.*, 2009, **113**, 6378–6396.

20 E. D. Glendening, J. K. Badenhoop, A. E. Reed, J. E. Carpenter, J. A. Bohmann, C. M. Morales and F. Weinhold, 2001.

21 A. J. Webster, C. M. Mueller, N. P. Foegen, P. H.-L. Sit, E. D. Speetzen, D. W. Cunningham and J. S. D'Acchioli, *Polyhedron*, 2016, **114**, 128–132.

22 E. Smolensky, M. Kapon and M. S. Eisen, *Organometallics*, 2007, **26**, 4510–4527.
